# Knowledge localization is associated with higher performance of domestic large language models in a Chinese radiation oncology examination

**DOI:** 10.3389/fonc.2026.1808714

**Published:** 2026-06-17

**Authors:** Yuchen Zhou, Shuyu Lin, Xinhai Wang, Ke Hu

**Affiliations:** 1 Department of Radiation Oncology, Peking Union Medical College Hospital, Chinese Academy of Medical Sciences and Peking Union Medical College, Beijing, China; 2 Tsinghua Medicine, School of Medicine, Tsinghua University, Beijing, China; 3 Department of Radiation Oncology, National Cancer Center/National Clinical Research Center For Cancer/Cancer Hospital, Chinese Academy of Medical Sciences and Peking Union Medical College, Beijing, China

**Keywords:** clinical reasoning, knowledge localization, large language models, medical education, radiation oncology

## Abstract

**Introduction:**

The rapid evolution of large language models has demonstrated human-level proficiency in general English-language medical examinations, yet their applicability in highly specialized, non-English clinical domains remains insufficiently explored.

**Methods:**

To bridge this gap, this study benchmarked leading domestic (Qwen 3 Max, DeepSeek V3.2) and international (GPT-5, Gemini 2.5 Flash) models against a board-certified Chinese radiation oncologist, using the National Intermediate Professional Title Examination for Radiation Oncology in China. Statistical comparisons were conducted using mixed-effects logistic regression and McNemar's test for paired data.

**Results:**

Domestic models demonstrated strong performance, with Qwen achieving an accuracy of 86.30%, which was higher than that of the single physician reference participant (adjusted P = 0.020). However, because only one human participant was included, this comparison should be interpreted as an illustrative reference rather than evidence of general superiority over radiation oncologists. In contrast, international models exhibited a marked performance decline in this specific context. Detailed cognitive stratification revealed a distinct reasoning-recall dissociation in international models, which maintained relatively robust clinical reasoning capabilities but failed significantly in localized knowledge retrieval. Notably, translating the examination into English did not bridge this performance gap for international models, while revealing a significant language penalty for certain domestic architectures (e.g., DeepSeek, P = 0.013). Granular error analysis confirmed that the majority of failures in international models like GPT-5 stemmed from discrepancies between Western and Chinese clinical guidelines rather than intrinsic reasoning deficits or hallucinations.

**Discussion:**

These findings suggest that, within this examination-based Chinese radiation oncology benchmark, alignment with regional clinical standards may be a major contributor to model performance. However, differences in model architecture, training data, post-training, interface configuration, and potential test-set contamination may also contribute.

## 1 Introduction

The emergence of large language models (LLMs) has marked a new era in medical informatics and education (1, 2). Built on transformer architectures and trained on vast datasets that include biomedical literature, models like ChatGPT and Claude have evolved from simple conversational agents into sophisticated reasoning engines capable of processing complex clinical scenarios (3). An expanding body of research has benchmarked these models against human standards, demonstrating that LLMs can perform at—and in some cases exceed—the level of medical students and residents. Notably, GPT-4 has been reported to pass the United States Medical Licensing Examination (USMLE) without any domain-specific fine-tuning (4).

Despite these promising results, the ability of LLMs to generalize to highly specialized clinical fields remains uncertain. Recent evaluations in medical oncology have revealed a distinct performance stratification. Although GPT-4 substantially outperforms earlier models such as GPT-3.5 with passing rates exceeding 80%, it continues to struggle with complex, case-based reasoning compared to factual recall questions (5–7). A similar pattern has been observed in radiology, where models show strong performance in lower-order recognition tasks but exhibit notable weaknesses in higher-order reasoning and problems involving spatial or physical logic (8). Even in disciplines such as ophthalmology, where text-based reasoning is relatively robust, LLMs still encounter challenges when confronted with multimodal or unstructured clinical inputs (9).

Radiation oncology represents an even more demanding test of domain specialization. Unlike disciplines primarily focused on pharmacotherapy, radiation oncology requires the integration of multidisciplinary expertise, including clinical management, radiobiological principles, and physical dosimetry. Whether general-purpose LLMs possess sufficient domain depth to recommend stage-appropriate treatments or delineate target volumes based solely on textual information remains largely unverified.

Beyond specialization, language and cultural contexts introduce additional sources of variability. Most existing medical LLM benchmarks, such as MedExpQA and PubMedQA, are primarily English-based, limiting their ability to evaluate model performance across languages and cultural contexts. Alonso et al. demonstrated that multilingual medical question-answering benchmarks remain scarce, and cross-lingual evaluations often reveal marked performance degradation when English-trained models are applied to other languages (10). Similarly, Huang et al. showed that even advanced foundation models perform considerably worse on Chinese-language tasks, underscoring the need for language-specific and culturally adapted evaluation frameworks (11). In China, this gap is further amplified by differences in epidemiology and clinical practice. For instance, malignancies such as nasopharyngeal carcinoma and esophageal squamous cell carcinoma occur far more frequently, prompting the development of domestic guidelines (e.g., Chinese Society of Clinical Oncology, CSCO) that diverge from Western standards (e.g., National Comprehensive Cancer Network, NCCN) in therapeutic sequencing and drug availability (12–14). Furthermore, Chinese medical communication presents a unique hybrid linguistic structure. Clinical narratives are primarily written in Chinese but frequently incorporate key English technical terms—such as TNM staging (e.g., cT4N2M0), molecular markers (e.g., HER-2, AFP), chemotherapy regimens (e.g., R-CHOP, TP), and imaging modalities (e.g., MRI, PET-CT) (15). Thus, models must not only interpret mixed-language input but also reason according to Chinese clinical guidelines that may differ from those represented in their pre-training data. Consequently, the reliability of globally trained LLMs within this localized clinical environment remains uncertain.

While our research group previously conducted a preliminary comparative analysis of GPT-4o and ERNIE Bot in this domain (16), the rapid evolution of model architectures and the unresolved impact of language on diagnostic reasoning underscore the need for a broader, updated evaluation. This study addresses these gaps by benchmarking a wider range of state-of-the-art models and examining how language presentation affects their performance on domain-specific clinical tasks. Specifically, we compare model accuracy on the original Chinese examination and a professionally translated English version to distinguish the effects of linguistic processing from those of underlying clinical reasoning.

To achieve this, we employed the National Intermediate Professional Title Examination for Radiation Oncology (NIPTE-RO) as an evaluation platform. Administered by the National Health Commission of China, the NIPTE-RO is a high-stakes, standardized examination required for physicians seeking promotion from resident to attending level. It serves as a rigorous benchmark of professional competence, assessing not only foundational medical knowledge but also advanced clinical reasoning within the context of Chinese epidemiology and domestic treatment guidelines. The examination consists of text-based clinical cases written in Chinese but containing essential English medical terminology (e.g., TNM staging, drug names). By evaluating LLMs against both the original Chinese text and a curated English translation, this study aims to determine whether global and domestic models can function as reliable decision-support tools in Chinese radiation oncology, and to what extent their performance is constrained by the language barrier itself.

## 2 Materials and methods

### 2.1 Study resources

The dataset used in this study was derived from a validated question bank established in prior research (16). While the original question bank encompassed the entire scope of the NIPTE-RO, this study specifically focused on the ‘Specialized Knowledge’ section, which evaluates theoretical principles, clinical guidelines, and treatment planning in radiation oncology rather than general medical fundamentals. The final dataset comprised 562 representative questions, stratified into eight clinical chapters. This distribution was designed to closely mirror the official examination syllabus and weighting guidelines, ensuring a highly representative evaluation of the discipline. The question bank used in this study consists of publicly available mock examination questions designed for NIPTE-RO preparation. The complete set of evaluated questions, including correct answer keys and detailed explanations, is provided in **Supplementary Material S1**. Three question formats were included in the question bank: standard multiple-choice questions (Type A1/A2), consisting of single questions with five options, questions with shared clinical vignettes (Type A3/A4), which link multiple questions to a single clinical case, and questions with shared options (Type B1), in which several questions share a common set of answer choices. The overall composition of the dataset and the distribution of question types are presented in **Table 1**.

**Table 1:** Composition of the study dataset for the NIPTE-RO.

Category	Count (n)	Percentage (%)
Question Type		
Standard Multiple-Choice Questions (Type A1/A2)	388	69.0
Questions with Shared Clinical Vignette (Type A3/A4)	102	18.1
Questions with Shared Options (Type B1)	72	12.8
Total (Question Type)	562	100.0
Clinical Chapter		
Head and Neck Tumors	178	31.7
Lung Cancer and Pleural Mesothelioma	55	9.8
Mediastinal Tumors	11	2.0
Digestive System Tumors	94	16.7
Breast Cancer	19	3.4
Tumors of the Urinary and Male Reproductive Systems	70	12.5
Malignant Lymphoma	54	9.6
Bone and Soft Tissue Tumors	81	14.4
Total (Clinical Chapter)	562	100.0

Percentages may not sum to exactly 100% due to rounding.

### 2.2 Dataset translation and validation

To investigate how LLMs perform on Chinese-language versus English-translated clinical questions, the original Chinese question bank was translated into English. The translation was carried out by a Chinese senior radiation oncologist with 15 years of clinical experience and advanced proficiency in academic medical English. The translation focused on conveying the precise clinical meaning rather than performing a literal, word-by-word conversion. Medical terminology, staging systems, and details specific to Chinese clinical guidelines (e.g., CSCO) were carefully preserved to ensure equivalence with the original content. After the initial translation, a second senior radiation oncologist independently reviewed the content to verify clinical accuracy and alignment with the original examination standards. The complete translated English version is provided in **Supplementary Material S2**.

### 2.3 Large language models

Four representative LLMs were selected for this study that reflect diverse architectural philosophies, access paradigms, and linguistic optimization strategies. These include both open-weight and closed-source systems spanning sparse, dense, and mixture-of-experts frameworks (**Table 2**).

**Table 2:** Overview of the four LLMs evaluated in this study.

Model	Developer	Access type	Architecture & scale	Context window
Qwen 3 Max	Alibaba Cloud (China)	Proprietary	Dense Transformer (Foundation Model)	128k Tokens
DeepSeek V3.2	DeepSeek-AI (China)	Open-weight	Mixture-of-Experts (MoE)	128k Tokens
Gemini 2.5 Flash	Google DeepMind (UK/USA)	Closed-source (API)	Multimodal Transformer	>1M Tokens
GPT-5	OpenAI (USA)	Closed-source (Standard Tier)	Proprietary (Next-Gen Transformer)	128k Tokens

Qwen 3 Max (Alibaba Cloud, China) represents the flagship foundation model of the Qwen series, employing a dense Transformer backbone. It is designed as a general-purpose large-scale model with enhanced reasoning and language alignment in Chinese and English. Its architecture emphasizes balanced performance across knowledge retrieval, reasoning, and text generation tasks (17).

DeepSeek V3.2 (DeepSeek-AI, China) adopts a Mixture-of-Experts (MoE) framework with open-weight availability. The model integrates expert routing for efficiency while maintaining high representational capacity, illustrating the trade-off between compute scalability and inference throughput. DeepSeek’s open-weight release supports transparency and broader community benchmarking (18).

Gemini 2.5 Flash (Google DeepMind, United Kingdom/United States) is a multimodal Transformer designed for ultra-fast inference and extended context processing. It natively supports text, vision, and audio inputs within a unified architecture and features latency-optimized routing. Gemini 2.5 Flash exemplifies the trend toward multimodal generalist models capable of integrating perception and reasoning (19).

GPT-5 (OpenAI, Standard Tier, United States) represents the latest generation of OpenAI’s proprietary Transformer family. It builds upon the GPT-4 architecture with expanded reasoning depth, multilingual comprehension, and adaptive response control. GPT-5 serves as a widely adopted benchmark for generalist AI systems, combining large-scale dense Transformer design with extensive instruction tuning and safety-layer refinement (20).

For clarity and consistency, these four models are hereafter referred to by their abbreviated names: Qwen, DeepSeek, Gemini, and GPT.

### 2.4 Model access and configuration

All data collection and experiments were conducted between November 15, 2025, and November 30, 2025. We evaluated the models via their official web-based interfaces to reflect typical end-user clinical conditions. However, to ensure rigorous reproducibility during this timeframe, we recorded the exact model name, official interface URL, account tier (e.g., Plus/Pro subscriptions), access date and time, and visible version information for each evaluated LLM.

Crucially, to assess intrinsic clinical knowledge, all external features including web browsing, memory, plug-ins, code execution, and retrieval-augmented generation tools were strictly disabled. Prompts were entered manually, and each question was submitted in a single run within a completely new session to prevent context leakage or memory carryover. A standardized prompt template was uniformly applied across all models. To provide full transparency, the extracted final answers, and the original, unmodified model outputs for all incorrect responses are thoroughly documented in **Supplementary Material S3**.

### 2.5 Prompt design

To evaluate the models’ intrinsic clinical reasoning within the Chinese medical context, a zero-shot prompting strategy was employed. Three standardized prompts were developed to align with the structural characteristics of the examination question types (Type A1/A2, Type A3/A4, and Type B1).

To ensure contextual consistency across tasks, each prompt included a predefined professional persona ‘a radiation oncologist preparing for the Chinese Intermediate Professional Title Examination’. The prompts were designed to instruct models to provide answers aligned with standard Chinese clinical practice and nationally endorsed guidelines. While this standardized approach provides a consistent benchmark for evaluating performance within a specific clinical framework, we recognize that model scores may reflect a combination of intrinsic localized knowledge and the ability to comply with complex clinical instructions. The complete text of the standardized prompts for each question type is available in **Supplementary Material S4**.

### 2.6 Human benchmark evaluation

To establish a baseline for human performance, a physician representative of typical NIPTE-RO candidates completed the full examination (N = 562). The participant had undergone the standard Chinese medical training pathway (‘5 + 3+3’ model) and completed the required residency in radiation oncology, meeting all eligibility criteria for the intermediate professional title assessment. The test was administered under controlled, closed-book conditions consistent with official examination procedures. This score serves as a benchmark for competent physician-level performance in radiation oncology.

### 2.7 Statistical analysis

Statistical analyses were performed using SPSS software (version 30.0.0). The primary outcome measure was accuracy, calculated as the percentage of correct responses relative to the total number of questions. The 95% confidence intervals (95% CIs) for accuracy estimates were calculated using the Wilson score interval method.

Because each model evaluated the exact same set of questions, the resulting data were paired. Consequently, pairwise comparisons between models (e.g., Qwen vs. GPT, or model vs. human), as well as intra-model comparisons across language versions (Chinese vs. English), were reanalyzed using McNemar’s test for paired nominal data. To control for the increased risk of Type I errors, P-values from multiple pairwise comparisons were adjusted using the False Discovery Rate (FDR) method (Benjamini-Hochberg procedure).

To assess the group-level effect of model origin (Domestic vs. International), we conducted a mixed-effects logistic regression analysis. Correctness was modeled as the binary outcome, model origin and presentation language were specified as fixed effects, and the specific question ID was included as a random intercept to account for item-level variability.

All statistical tests were two-sided, and an (adjusted) P-value of <0.05 was considered statistically significant. Data visualization was conducted using GraphPad Prism (version 10.4.0).

## 3 Results

### 3.1 Overall performance and human baseline evaluation

**Figure 1:**
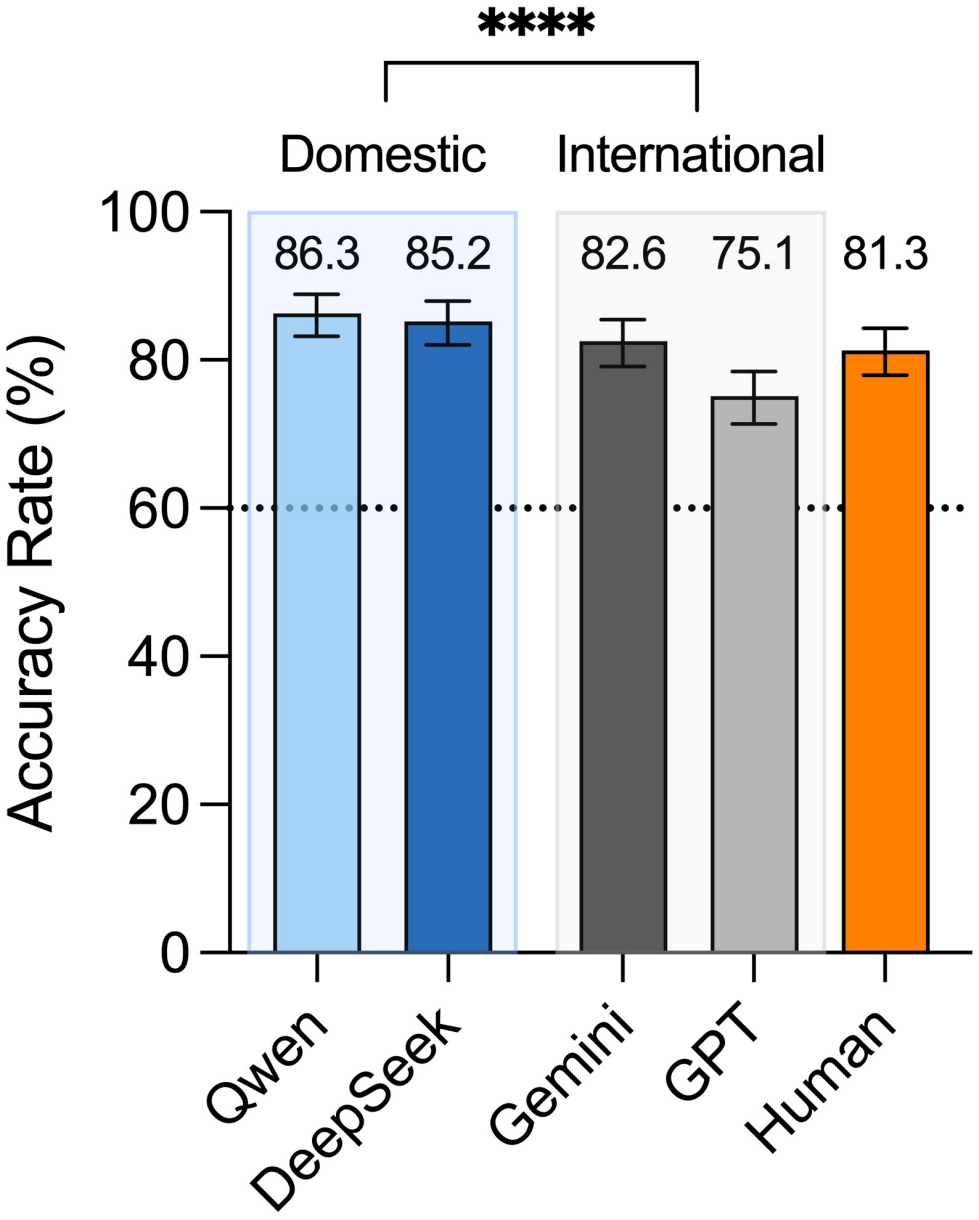
Performance comparison between domestic and international LLMs on the NIPTE-RO question bank. The overall accuracy across the complete dataset (N = 562). Models are grouped by development origin: domestic models (Qwen, DeepSeek) are shown in blue, international models (Gemini, GPT) in gray, and the human radiation oncologist benchmark in orange. The dashed line indicates the official 60% passing threshold for the NIPTE-RO examination, which corresponds to the fixed national qualifying standard established by the Ministry of Human Resources and Social Security of the People’s Republic of China for health professional technical qualification examinations. Error bars indicate 95% Wilson confidence intervals. Statistical brackets indicate the group-level comparison between pooled domestic and pooled international models; ****P<0.0001 calculated via mixed-effects logistic regression.

To examine how model origin influences diagnostic performance in Chinese medical contexts, we compared two groups of LLMs—domestic (Qwen, DeepSeek) and international (Gemini, GPT)—using the NIPTE-RO dataset (N = 562). As shown in **Figure 1**, the domestic models demonstrated consistently high accuracy. Qwen achieved an accuracy of 86.30% (485/562) and DeepSeek achieved 85.23% (479/562). Notably, the difference between these two domestic models was not statistically significant (adjusted P = 0.602).

In contrast, the international group displayed greater variability and overall lower accuracy. Gemini achieved a competitive accuracy of 82.56% (464/562), whereas GPT performed significantly lower at 75.09% (422/562; adjusted P<0.0001 in comparison to Qwen, and adjusted P<0.0001 in comparison to DeepSeek). When aggregated by development origin, the domestic group significantly outperformed the international group (85.77% vs. 78.83%; P<0.0001, mixed-effects logistic regression).

Model performance was benchmarked against a board-certified radiation oncologist to provide a clinical reference (**Table 3**). The human specialist achieved a baseline accuracy of 81.32% (457/562). Notably, Qwen was the only model to score higher than the single human benchmark (adjusted P = 0.020), indicating that a domestic model can achieve high diagnostic accuracy that is highly competitive with a board-certified specialist. However, given the N = 1 limitation, this result serves as an initial comparative reference rather than definitive evidence that the model can generally exceed professional clinical accuracy. DeepSeek (adjusted P = 0.061) and Gemini (adjusted P = 0.602) performed comparably to the human, whereas GPT remained significantly below the baseline (adjusted P = 0.007).

**Table 3:** Comparative accuracy of LLMs versus the human specialist.

Model	Accuracy	Difference (model-human)	Adjusted p-value
Qwen 3 Max	86.30% (485/562)	+4.98%	0.020
DeepSeek V3.2	85.23% (479/562)	+3.91%	0.061
Gemini 2.5 Flash	82.56% (464/562)	+1.24%	0.602
GPT-5	75.09% (422/562)	-6.23%	0.007

The human radiation oncologist achieved a baseline accuracy of 81.32% (457/562). Individual model performance was compared against this human benchmark using McNemar’s test for paired data. P-values were adjusted for multiple comparisons using the False Discovery Rate (FDR) method.

### 3.2 Performance stratification by question type

**Figure 2:**
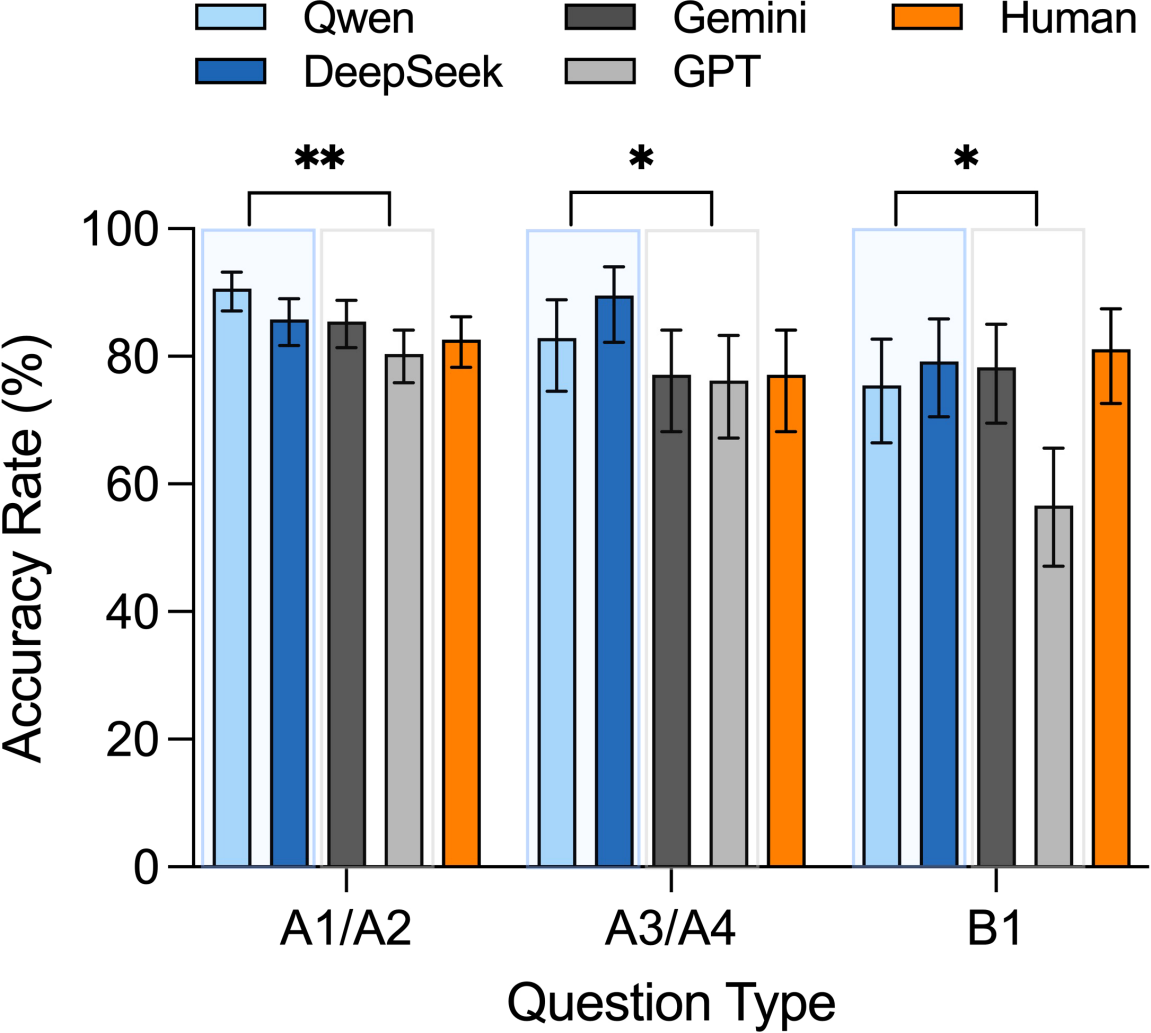
Accuracy of LLMs and human participant across question type. Accuracy for Qwen, DeepSeek, Gemini, GPT and human participant across three question types: A1/A2 (single best choice), A3/A4 (case-based shared stem), and B1 (standard matching). Error bars indicate 95% Wilson confidence intervals. Statistical brackets indicate group-level comparisons between pooled domestic and pooled international models within each question type; **P<0.01, *P<0.05 calculated via mixed-effects logistic regression.

To determine whether the performance gap persisted across different cognitive task types, we evaluated model accuracy across three standard question formats: A1/A2 (knowledge recall), A3/A4 (case analysis), and B1 (matching) (**Figure 2**). Across all categories, domestic models (Qwen and DeepSeek) consistently outperformed international models (Gemini and GPT), demonstrating superior adaptability across distinct cognitive demands.

In the A1/A2 category, which primarily assesses factual recall, Qwen achieved the highest accuracy (>90%), followed closely by DeepSeek. At the group level, domestic models significantly exceeded the accuracy of the international models (P<0.01). In A3/A4 case-based analysis questions, which usually require multi-step reasoning, the domestic models again achieved superior performance (P<0.05). In the B1 matching-type questions, Qwen and DeepSeek maintained significant advantages over Gemini and GPT (P<0.05). In contrast, the human benchmark remained relatively stable across question types (~80%), whereas the LLMs displayed greater variability. Notably, GPT showed a pronounced drop in B1 questions, reflecting reduced stability in this format.

### 3.3 Cognitive complexity analysis: knowledge recall vs. clinical reasoning

**Figure 3:**
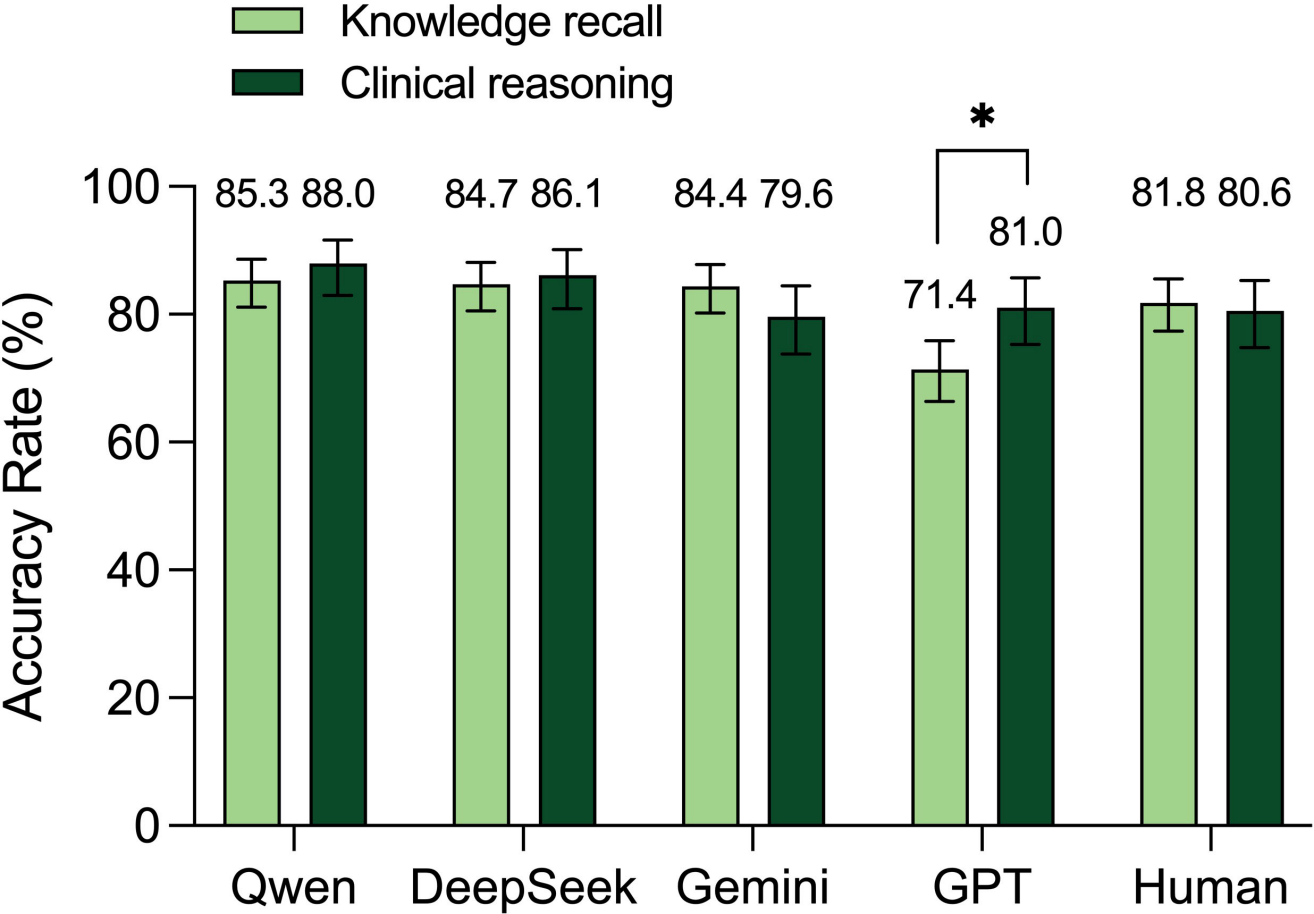
Accuracy of LLMs and human participant on knowledge recall and clinical reasoning tasks. Accuracy across two cognitive domains: knowledge recall (N = 346) and clinical reasoning (N = 216). Error bars indicate 95% Wilson confidence intervals. Statistical brackets indicate intra-model comparisons between independent cognitive domains; *P<0.05 calculated via Pearson’s chi-square test.

Building on the previous findings, we next examined how models handle distinct cognitive processes. Each question was independently classified into two domains by two senior radiation oncologists: (1) knowledge recall, defined as the direct retrieval of specific clinical facts, doses, or guideline clauses; and (2) clinical reasoning, defined as the synthesis of patient-specific data to determine a diagnosis or treatment plan. Inter-rater agreement for this classification was high (Cohen’s kappa = 0.821), with any discrepancies resolved through consensus discussion. The final dataset comprised 346 knowledge recall and 216 clinical reasoning items (**Figure 3**).

Domestic models demonstrated dual-domain competency. Qwen and DeepSeek maintained balanced high accuracy in recall and reasoning tasks. Specifically, Qwen achieved 85.26% on recall and 87.96% on reasoning, with no statistically significant difference between categories (P = 0.44).

In contrast, international models revealed a reasoning-recall dissociation. GPT showed a paradoxical pattern, performing significantly worse on knowledge recall (71.39%) but better on clinical reasoning (81.02%; P=0.014). Conversely, Gemini exhibited the opposite trend, with slightly higher proficiency in recall (84.39%) than reasoning (79.63%), though the difference was not statistically significant (P = 0.18).

Importantly, when isolating reasoning performance, the performance gap between Qwen (87.96%) and GPT (81.02%) narrowed compared to the knowledge recall gap, though it remained statistically significant (P = 0.014, McNemar’s test). This narrowed gap suggests that the observed disadvantage of GPT is primarily driven by informational misalignment (knowledge gap) rather than a profound inherent reasoning limitation.

### 3.4 Domain-specific performance across clinical chapters

**Figure 4:**
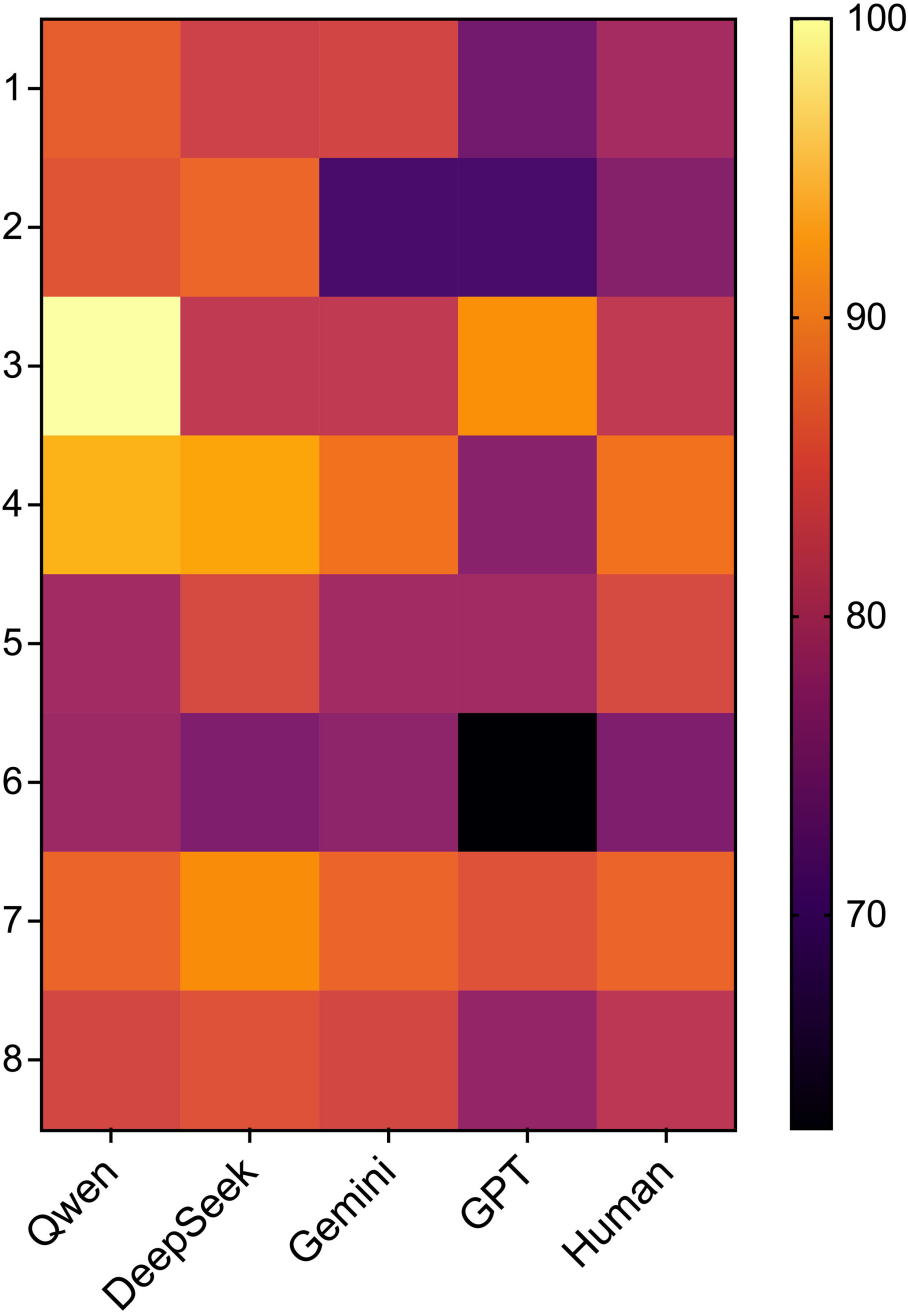
Heatmap of accuracy across the eight clinical chapters. The Y-axis represents individual chapters: (1) Head and Neck Tumors; (2) Lung Cancer and Pleural Mesothelioma; (3) Mediastinal Tumors; (4) Digestive System Tumors; (5) Breast Cancer; (6) Tumors of the Urinary and Male Reproductive Systems; (7) Malignant Lymphoma; and (8) Bone and Soft Tissue Tumors. The X-axis displays the four evaluated LLMs and the human specialist benchmark. The color scale indicates accuracy ranging from 60% (dark purple/black) to 100% (bright yellow).

To assess whether model localization advantages extend across clinical subspecialties, we analyzed accuracy within eight anatomical chapters (**Figure 4**).

Domestic models showed strong performance in several anatomical domains. In Chapter 3 (Mediastinal Tumors, n=11), Qwen achieved a perfect score (11/11, 100%), which was higher than the human specialist (9/11, 81.82%). However, due to the small sample size of this chapter, this result should be interpreted as a descriptive trend rather than a definitive measure of superiority. Similarly, in Chapter 4 (Digestive System Tumors, n=94), both Qwen (88/94, 93.62%) and DeepSeek (87/94, 92.55%) performed better than the human benchmark (83/94, 88.30%).

However, the advantage of LLMs was not universal. Chapter 6 (Tumors of the Urinary and Male Reproductive Systems, n=70) was the most challenging for both human and models. The human specialist correctly answered 53 questions (75.71%), matching DeepSeek (53/70, 75.71%) and slightly below Qwen (55/70, 78.57%). In contrast, GPT showed a marked drop in this domain, with only 44 correct answers (62.86%).

Overall, performance stability differed between the human reference and models. The human specialist maintained a narrow accuracy range (75.71%–88.30%) across all chapters, whereas international models showed greater variability. GPT, for example, ranged from 62.86% in Chapter 6 to 90.91% in Chapter 3. Although results in several chapters are subject to small-sample noise, these findings suggest that while top-performing LLMs can achieve high accuracy in specific knowledge-based tasks, their performance across subspecialties exhibits greater variability compared to the reference baseline.

### 3.5 Impact of language presentation on LLM accuracy

**Figure 5:**
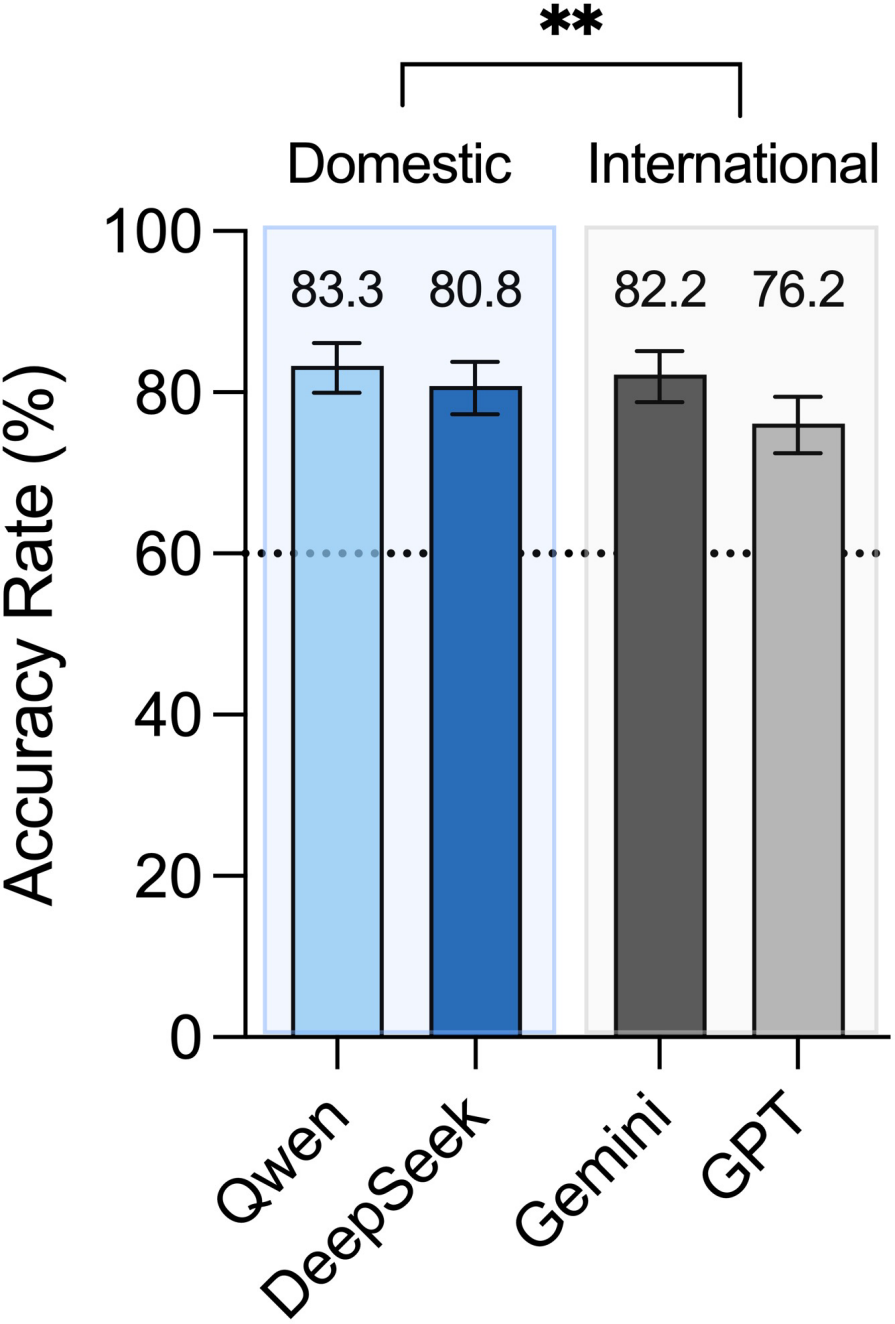
Comparison of domestic and international LLM performance on the English-translated NIPTE-RO dataset. Accuracy rates (%) for Qwen, DeepSeek, Gemini, and GPT with 95% Wilson confidence intervals on the English-translated question bank. Domestic models (Qwen, DeepSeek) are represented in blue, and international models (Gemini, GPT) in gray. Statistical brackets indicate the group-level comparison between pooled domestic and pooled international models; **P<0.01 calculated via mixed-effects logistic regression.

Given the observed performance differences in the Chinese setting, we next examined whether these disparities were primarily associated with language proficiency rather than knowledge localization. To separate the effects of language proficiency from knowledge localization, model performance was tested using a professionally translated English version of the NIPTE-RO dataset (**Figure 5**; **Table 4**).

**Table 4:** Comparison of LLM accuracy between Chinese and English-translated versions of the NIPTE-RO dataset.

Model	Chinese accuracy (N = 562)	English accuracy (N = 562)	Performance gap	P-value
Qwen	86.30% (485)	83.27% (468)	-3.03%	0.076
DeepSeek	85.23% (479)	80.78% (454)	-4.45%	0.013
Gemini	82.56% (464)	82.21% (462)	-0.35%	0.923
GPT	75.09% (422)	76.16% (428)	+1.07%	0.655

Table legend: Statistical comparisons were performed using McNemar’s test for paired data. Positive gaps indicate better performance in English.

Statistical analysis using McNemar’s test for paired data revealed varying degrees of language sensitivity among the domestic models. Qwen demonstrated resilience to language switching, maintaining a high accuracy of 83.27% (468/562) in the English version, with a non-significant decline from its Chinese performance (Δ=−3.03%, P = 0.076). However, DeepSeek exhibited a statistically significant decrease in accuracy when tested in English (85.23% to 80.78%, Δ=−4.45%, P = 0.013).

Conversely, international models did not benefit from a native-language advantage. Gemini displayed near-identical performance across languages (82.56% vs. 82.21%; P = 0.923), and GPT showed only a non-significant improvement in its native English context (75.09% to 76.16%; Δ=+1.07%, P = 0.655). Even in English, GPT remained significantly below Qwen’s Chinese performance (76.16% vs. 86.30%, P<0.0001), confirming that the primary performance disparities stem from medical data alignment rather than linguistic proficiency.

Collectively, these findings indicate that knowledge localization plays a more critical role than language fluency in achieving diagnostic accuracy in Chinese clinical contexts, although language alignment remains a factor for certain domestic architectures.

### 3.6 Mechanistic analysis of failure modes of GPT-5

**Figure 6:**
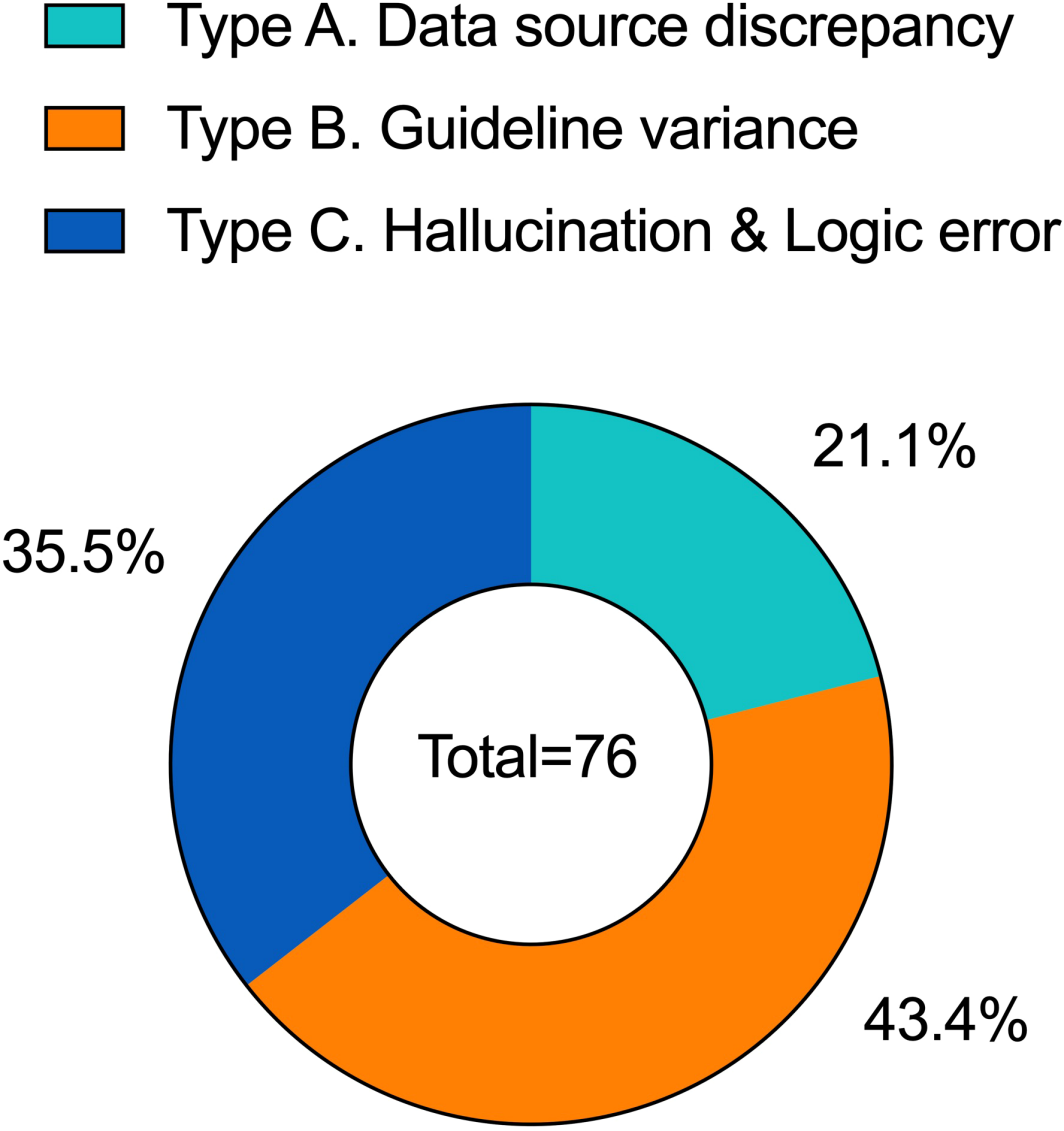
Distribution of error types identified in GPT-generated responses. The donut chart illustrates the distribution of identified error mechanisms (N = 76). Type A (Data Source Discrepancy, 21.1%) denotes errors arising from the citation of non-local epidemiological data. Type B (Guideline Variance, 43.4%) refers to conflicts between international protocols and Chinese national guidelines. Type C (Hallucination & Logic Error, 35.5%) represents intrinsic cognitive failures.

To further verify whether knowledge misalignment—rather than reasoning deficiency and language fluency—predominantly explains the observed performance differences, we conducted a granular error analysis. GPT was specifically selected for this analysis because it exhibited the lowest overall accuracy and the most pronounced reasoning-recall dissociation, thus providing the most distinct theoretical window into the failure modes of international models in this localized context. Out of the 140 total incorrect responses generated by GPT in the original Chinese question bank, 64 were excluded based on predefined criteria: specifically, cases where the model either failed to provide an explanatory rationale or generated ambiguous outputs that precluded mechanistic classification. The remaining 76 cases, representing the entirety of evaluable incorrect responses with clear and traceable reasoning steps, were subjected to detailed qualitative analysis (**Figure 6**; **Table 5**).

**Table 5:** Classification and definition of GPT error categories.

Error category	Count	Percentage	Definition & implication
Type A: Data source discrepancy	16	21.1%	Data mismatch: The model cites correct Western epidemiological data (e.g., US incidence rates from ACS/SEER) that is factually incorrect within the context of Chinese epidemiology.
Type B: Guideline variance	33	43.4%	Conflict in standards: The model follows international (e.g., NCCN) or general evidence-based protocols that differ from the specific dosage, fractionation, or surgical timing required by Chinese national guidelines (CSCO) or textbooks.
Type C: Hallucination & Logic error	27	35.5%	Cognitive failure: Fundamental errors in anatomy, pathology classification, or logical failures in interpreting the clinical vignette (e.g., contradictory reasoning).
Total	76	100%	

Errors were grouped into three categories based on predefined operational definitions: Type A (Data Source Discrepancy), Type B (Guideline Variance), and Type C (Hallucination & Logic Error). The classification process was independently performed by two analysts (senior radiation oncologists) to minimize subjective bias. Inter-rater agreement for the error categorization was strong (Cohen’s kappa = 0.856). Any classification discrepancies were resolved through consensus discussion. The final denominator for this analysis was established as N = 76, and all percentages in **Figure 6** and **Table 5** have been synchronized to this total. Detailed final error annotations for all 76 evaluable cases are provided in **Supplementary Material S5**.

As shown in **Figure 6**, most errors were ‘extrinsic’ (Type A and B), which indicate mismatches in medical knowledge rather than reasoning failures. Specifically, 64.5% of errors (49/76) were due to conflicts between international and Chinese medical standards. The largest group, Type B (43.4%, 33/76), involved answers that were valid under international protocols (e.g., NCCN guidelines or Western dosing standards) but inconsistent with Chinese national guidelines (CSCO) or textbooks. Type A (21.1%, 16/76) included instances where the model cited accurate Western epidemiological data, such as U.S. cancer incidence rates from ACS/SEER, that contradicted Chinese-specific statistics required by the exam.

In contrast, ‘intrinsic’ reasoning failures were less common. Type C errors (35.5%, 27/76) involved factual hallucinations or logical inconsistencies, such as anatomical misinterpretation or flawed reasoning in clinical cases.

**Table 6** provides representative examples of these discrepancies. For example, in Question 5 (Chapter 1), GPT reported that head and neck tumors account for ‘<5%’ of all malignancies, reflecting U.S. epidemiological data rather than the correct Chinese proportion.

**Table 6:** Examples of GPT failure modes.

Error category	Question ID	Clinical domain	Specific conflict: GPT response vs. standard answer (CN)
Type A: Data source discrepancy	Ch 1, Q5	Epidemiology (Head & Neck)	GPT (US Data): Cites American Cancer Society (ACS) statistics, stating head & neck tumors account for <5% of malignancies. Standard (CN Data): 5%-10%, reflecting higher incidence rates (e.g., nasopharyngeal carcinoma) in the Chinese population.
Type B: Guideline variance	Ch 6, Q62	Radiotherapy dose (Seminoma)	GPT (Intl. Protocol): Recommends 40–45 Gy for postoperative radiotherapy, referencing salvage or high-risk international protocols. Standard (CSCO/Textbook): 20–30 Gy, adhering to the standard prophylactic dose for Stage I seminoma in China.
Type C: Hallucination & Logic error	Ch 2, Q10	Pathology (Lung cancer)	GPT (Hallucination): Incorrectly identifies ‘Oat cell type’ as the most common pathological subtype of small cell lung cancer. Standard (Fact): Intermediate cell type (or generic SCLC), as ‘oat cell’ is a historical term and not the primary modern classification.

This analysis suggests that GPT’s response pattern resembled that of a model aligned with international rather than Chinese guideline sources, lacking the localized medical knowledge necessary for the Chinese context. Its limitations are likely to stem from insufficient alignment with regional guidelines and data sources, but not from a deficit in reasoning ability.

## 4 Discussion

This study provides a comprehensive evaluation of LLMs in the domain of Chinese radiation oncology. The results highlight a clear performance gap based on the model’s origin (**Figure 1**). Domestic models (Qwen and DeepSeek) showed stronger alignment with the local domain, with accuracies outperforming international models (Gemini, GPT) and, in the case of Qwen, scoring higher than the single human specialist baseline employed in this study. In contrast, despite their well-documented performance in English-language benchmarks such as the USMLE (4), international models showed a notable decline in this localized context. Beyond overall accuracy, model performance also varied across question types and cognitive categories (**Figures 2**, **3**), clinical chapters (**Figure 4**), and language settings (**Figure 5**). Collectively, these results suggest that the observed differences arise mainly from knowledge alignment—the ability to adhere to region-specific medical standards—rather than from deficits in reasoning or language proficiency, although testing language can still influence the efficiency of knowledge retrieval for certain domestic architectures.

The strong performance of Qwen and DeepSeek underscores the central role of training data composition and local adaptation in specialized medical AI. In this context, the developmental origin of the models serves as a proxy for their underlying training corpora—domestic models inherently benefit from massive pre-training on Chinese clinical literature, guidelines, and localized medical discourse. Both models maintained balanced accuracy in factual recall and clinical reasoning tasks (**Figure 3**), indicating effective integration of local medical knowledge. This finding aligns with recent studies showing that domain-adapted pretraining and fine-tuning improve factual accuracy and reliability in clinical tasks (21). Exposure to Chinese clinical guidelines (e.g., CSCO) and examination materials likely contributed to this advantage, allowing the models to act as local experts who reason according to domestic clinical standards and regional disease patterns, such as the high incidence of nasopharyngeal carcinoma and China-specific radiotherapy fractionation schemes.

A key finding of this study is the reasoning-recall dissociation observed in GPT (**Figure 3**). Although GPT demonstrated relatively strong reasoning ability (81.0%), its performance in knowledge recall was markedly lower (71.4%). This pattern challenges the assumption that LLMs underperform in non-English clinical settings because of suboptimal reasoning or hallucinations. Further error analysis (**Figure 6**) indicates that 64.5% of GPT’s errors were extrinsic, arising from discrepancies between international and Chinese medical standards rather than from intrinsic logical failures. In practice, GPT frequently produced answers that were clinically valid under international frameworks such as NCCN guidelines but inconsistent with Chinese national standards (CSCO). This pattern suggests that GPT may function as a competent ‘international consultant’, that is capable of solid reasoning but insufficiently aligned with local clinical practices. Therefore, the observed performance gap may primarily reflect a lack of knowledge localization rather than a profound failure of medical reasoning. This finding is consistent with recent work emphasizing that domain-specific data alignment, not model scale, is the key factor for clinical reliability in LLMs in specialized domains (2, 3, 22).

These findings have several implications for the future development and deployment of medical AI systems within specialized regional contexts. Because reasoning capability appears largely preserved, the observed performance gap in international models may be more effectively addressed through enhanced knowledge integration rather than extensive retraining. Recent studies suggest that augmenting general-purpose LLMs with domain-specific or region-specific medical corpora can significantly enhance factual accuracy and contextual reliability (3, 11). Incorporating localized clinical guidelines such as CSCO standards or domestic staging systems via retrieval-augmented generation (RAG) or continual fine-tuning could substantially reduce the observed knowledge misalignment. This approach aligns with emerging evidence that grounding model outputs in trusted, structured medical sources improves safety and clinical consistency across languages and health systems (2, 16). Collectively, these observations highlight that effective clinical adaptation depends not only on reasoning strength but also on the contextual calibration of medical knowledge to local standards and data ecosystems.

At the same time, the results reveal the limitations of current global benchmarks, such as MedQA and PubMedQA, which primarily reflect Western clinical practices. MedQA consists of English-language questions derived from U.S. medical licensing exam formats while PubMedQA is constructed from English PubMed abstracts (23, 24). Models that perform well on these datasets may still generate unsafe or contextually inappropriate outputs in other regions where disease prevalence, treatment accessibility, and guideline frameworks differ. Future evaluation frameworks should therefore adopt region-specific benchmarks, based on local guidelines and epidemiological realities, to ensure both fairness and clinical safety in real-world implementation.

The observed consistency of the human specialist across question types (**Figure 2**) and clinical chapters (**Figure 4**) further emphasizes the complementary roles of clinicians and LLMs. While top-performing models can reach or exceed human-level accuracy in knowledge-dense domains, human experts remain more stable across diverse and evolving contexts. This aligns with prior studies showing that AI systems are most effective when used to support, rather than replace, clinical expertise (1, 2). Human judgment remains essential for contextual reasoning and error detection. Such evidence supports the positioning of LLMs as clinical decision-support tools rather than independent agents. Furthermore, future evaluations should assess not only answer accuracy but also confidence calibration and uncertainty quantification, because overconfident incorrect responses may be particularly unsafe in clinical decision-support settings. Relevant board-examination work includes Madrid et al., who evaluated plug-in-augmented ChatGPT on the German Medical Board Examination and assessed the models’ ability to quantify uncertainty (25).

Several limitations should be noted. First, the evaluation relied on text-based multiple-choice questions. Although standardized, it cannot fully capture the multimodal reasoning required in real-world radiotherapy practice (e.g., image interpretation, treatment planning). Second, the human benchmark consisted of a single board-certified specialist. Given the substantial performance variability among individual clinicians, this N = 1 comparator serves only as a preliminary reference rather than a robust clinical standard. Therefore, our results should not be overinterpreted as models achieving general ‘superhuman’ proficiency. Expanding to a diverse panel of experts would provide a more representative baseline in future studies. Third, the distribution of questions across clinical chapters is uneven. Consequently, performance estimates in smaller subspecialty chapters (e.g., Mediastinal Tumors, n=11; Breast Cancer, n=19) are subject to statistical noise, and claims regarding subspecialty-specific superiority should be interpreted with caution.

Fourth, the error taxonomy was manually annotated. Despite predefined operational criteria and cross-validation among investigators, subjective judgment remains a potential source of bias.

Fifth, test-set contamination is an inherent concern in LLM evaluation. To address this, we searched for exact and near-exact matches of our question bank in publicly available online sources and found no direct matches. Furthermore, an evaluation of the models’ outputs revealed no verbatim reproduction of our standard answer explanations, suggesting that the performance of the models was primarily based on clinical reasoning rather than simple memorization. Nevertheless, because the pre-training datasets of these models are not fully transparent, the theoretical potential for hidden contamination cannot be absolutely eliminated and remains a limitation. Future research should prioritize sensitivity analyses utilizing unpublished, newly authored clinical questions.

Sixth, our evaluation utilized a prespecified, guideline-specific prompt. Without a formal prompt sensitivity analysis (e.g., comparing neutral versus persona-based prompts), we cannot definitively disentangle the models’ intrinsic knowledge from their instruction-following proficiency. Consequently, the observed performance may partially reflect varying degrees of sensitivity to the provided clinical instructions.

Seventh, our evaluation relied on a single-run testing protocol for each clinical question. Due to the inherent stochasticity of LLMs operating at default web-interface temperature settings, a model’s output may exhibit variability across multiple iterations. While single-run manual prompting reflects a realistic zero-shot use case by a clinician, future studies should employ API-based programmatic testing with multiple iterations to quantitatively assess the response stability and confidence calibration of these models.

Furthermore, the dataset itself, which derived from a national standardized examination, primarily measures theoretical and guideline-based knowledge. Although well-validated, it does not fully reflect the dynamic decision-making and patient-specific considerations encountered in daily clinical oncology practice.

Finally, because the pre-training datasets of proprietary models like GPT-5 and Gemini remain undisclosed, we cannot definitively isolate the effect of knowledge localization from other unobserved variables in the training process. Therefore, our conclusions regarding the mechanisms of the performance gap remain hypotheses supported by our error analysis rather than established causal facts.

In conclusion, domestic large language models have reached professional-level proficiency in Chinese radiation oncology, a performance advantage that we hypothesize is strongly associated with superior knowledge localization. For international models, the principal barrier appears to be the ability to internalize and apply region-specific clinical norms. The future of AI in specialized medical disciplines will depend not merely on scaling model size, but on aligning its embedded knowledge with local medical realities. Bridging this knowledge alignment gap represents a critical step toward developing AI systems that are clinically trustworthy across diverse regional practices.

## Data Availability

The original contributions presented in the study are included in the article/**Supplementary Material**. Further inquiries can be directed to the corresponding author.
