# Supplementary material for: Knowledge localization is associated with higher performance of domestic large language models in a Chinese radiation oncology examination
Source: Front Oncol. 2026 Jun 17;16:1808714. doi: 10.3389/fonc.2026.1808714 (PMC13318762; doi:10.3389/fonc.2026.1808714)
Supplement: Supplementary file 1 [file Table1.docx]

**第1章 头颈部肿瘤（N=178）**

1. 在我国，男性头颈部恶性肿瘤发病首位是（ ）。
   A. 甲状腺癌
   B. 鼻咽癌
   C. 口腔癌
   D. 喉癌
   E. 上颌窦癌
   参考答案：B
   【解析】我国是鼻咽癌的高发地区，男性多于女性。在我国，男性头颈部恶性肿瘤发病首位是鼻咽癌，其次是喉癌、口腔癌。
2. 在我国，女性头颈部恶性肿瘤发病首位是（ ）。
   A. 甲状腺癌
   B. 鼻咽癌
   C. 口腔癌
   D. 喉癌
   E. 上颌窦癌
   参考答案：A
   【解析】在我国，女性头颈部恶性肿瘤发病首位是甲状腺癌，其次是鼻咽癌、口腔癌。
3. 通常所指的头颈部肿瘤除外的是（ ）。
   A. 甲状腺癌
   B. 鼻咽癌
   C. 脑胶质瘤
   D. 口腔癌
   E. 外耳道鳞癌
   参考答案：C
   【解析】头颈部肿瘤包括自颅底到锁骨上、颈椎以前这一解剖范围内的肿瘤，通常不包括颅内、颈椎肿瘤和眼内肿瘤。脑胶质瘤有自己独特的生物学行为和治疗模式，不属于通常所说的头颈部肿瘤的范畴。
4. 在我国，头颈部恶性肿瘤发病人数占全身恶性肿瘤的百分比大约是（ ）。
   A. 15%～20%
   B. 1%～5%
   C. 10%～15%
   D. 5%～10%
   E. 20%以上
   参考答案：D
   【解析】在我国，头颈部恶性肿瘤占全身恶性肿瘤的比例接近10%，所以正确答案是D。
5. 在美国，头颈部恶性肿瘤发病人数占全身恶性肿瘤的百分比大约是（ ）。
   A. 15%～20%
   B. 5%以下
   C. 10%～15%
   D. 5%～10%
   E. 20%以上
   参考答案：D
   【解析】美国国家肿瘤数据库资料统计，从1985～1994年，有头颈肿瘤患者301350人，占全身恶性肿瘤的6.6%，所以答案是D。
6. 头颈部恶性肿瘤最常见的病理类型是（ ）。
   A. 淋巴瘤
   B. 鳞状细胞癌
   C. 未分化癌
   D. 腺癌
   E. 软组织肉瘤
   参考答案：B
   【解析】没有试题分析
7. 头颈部恶性肿瘤若先行术前放疗，放疗结束后再行手术的最佳时机是（ ）。
   A. 放疗结束后4～6周
   B. 放疗结束后1周以内
   C. 放疗结束后2～4周
   D. 放疗结束后1～2周
   E. 放疗结束后6～8周
   参考答案：C
   【解析】放疗结束后2～4周是再行手术的最佳时机。如果间隔时间过短，放疗急性反应未过；间隔时间过长，会导致组织纤维化形成，这些均会导致手术难度增大和并发症增加。
8. 以下头颈部恶性肿瘤中治疗效果最差的是（ ）。
   A. 鼻咽癌
   B. 甲状腺癌
   C. 喉癌
   D. 腮腺癌
   E. 下咽癌
   参考答案：E
   【解析】头颈部肿瘤治疗中甲状腺癌、腮腺癌、喉癌等疗效好，而下咽癌、颈段食管癌等最差。下咽癌容易发生淋巴转移，局部病变广泛，早期症状不典型，远处转移几率高，所以治疗效果差。正确答案是E。
9. 头颈部恶性肿瘤总的治愈率在（ ）。
   A. 40%～70%
   B. 10%～20%
   C. 30%～40%
   D. 20%～30%
   E. 70%～100%
   参考答案：A
   【解析】没有试题分析
10. 下列有关头颈部肿瘤治疗及预后的说法，正确的是（ ）。
    A. 同步放化疗不能用于头颈部肿瘤的术前治疗
    B. 头颈部肿瘤大多病变表浅，有利于早期发现和诊断，所以治愈率较高
    C. 颈部淋巴结转移并不影响患者的生存率
    D. 下咽癌治疗效果较好，腮腺癌、喉癌治疗效果较差
    E. 头颈部肿瘤术前放疗剂量在60～70Gy
    参考答案：B
    【解析】头颈部肿瘤大多由于病变表浅，有利于早期发现和诊断，所以治愈率较高，可达40％～70％。
11. 下列化疗药物不属于头颈部肿瘤化疗常用药物的是（ ）。
    A. 多西紫杉醇
    B. 顺铂
    C. 紫杉醇
    D. 氟尿嘧啶
    E. 伊立替康
    参考答案：E
    【解析】头颈部肿瘤常用的化疗药物有顺铂、氟尿嘧啶、紫杉醇类，而伊立替康对头颈部肿瘤有效率低，不是常用药物，所以正确答案是E。
12. 下列对同步放化疗在头颈部肿瘤治疗中的应用，错误的描述是（ ）。
    A. 同步放化疗不良反应严重，患者不能耐受
    B. 同步放化疗的应用，可以提高头颈部肿瘤治疗的局部控制率
    C. 目前常用于头颈部肿瘤同步放化疗的药物有顺铂、氟尿嘧啶、紫杉醇类等
    D. 化疗药物有放疗增敏作用
    E. 手术前也可以进行同步放化疗
    参考答案：A
    【解析】同步放化疗是头颈部肿瘤治疗中的一个重要进展，目前在鼻咽癌经临床试验证实可提高肿瘤局部控制率。同步放化疗化疗药物有提高放疗疗效的增敏作用，但同步放化疗的不良反应比单纯放疗要重，但是可控的，经过处理患者是可以耐受的。
13. 下列属于鼻腔及副鼻窦恶性肿瘤最佳治疗方式的是（ ）。
    A. 放疗结合化疗
    B. 单纯手术
    C. 放疗结合手术
    D. 单纯放疗
    E. 手术结合化疗
    参考答案：C
    【解析】鼻腔及副鼻窦恶性肿瘤治疗方式主要以放射治疗与手术结合的综合治疗为主，疗效明显优于单纯放疗或单纯手术。
14. 关于鼻腔及副鼻窦恶性肿瘤的治疗，下列说法不正确的是（ ）。
    A. 恶性黑色素瘤应采取以放疗、手术、化疗及生物治疗联合的综合治疗模式
    B. 上颌窦未分化癌只需行单纯放疗
    C. 临床A期的嗅神经母细胞瘤可单纯手术或单纯放疗
    D. 若上颌窦顶壁和眶内软组织受侵可保留眶内容物，术后补充放疗
    E. 有颈部淋巴结转移时应行根治性颈淋巴结清扫术
    参考答案：D
    【解析】上颌窦未分化癌，只需给予单纯放疗。手术中探查上颌窦顶壁，如果顶壁结构可以随上颌骨切除，而与眶内软组织没有明显粘连，可以保留眶内容物。如果肿瘤明显侵犯顶壁和眶内软组织，应切除眶内容物。对临床A期的嗅神经母细胞瘤可单纯放疗或单纯手术。恶性黑色素瘤应以放疗联合手术、化疗及生物治疗的综合治疗模式为宜。当出现颈部淋巴结转移时，应行根治性颈部淋巴结清扫。
15. 颌骨恶性肿瘤最常见的病理类型是（ ）。
    A. 纤维肉瘤
    B. 骨肉瘤
    C. 恶性纤维组织细胞瘤
    D. 软骨肉瘤
    E. 淋巴瘤
    参考答案：B
    【解析】颌骨恶性肿瘤中常见的原发性颌骨恶性肿瘤为骨肉瘤，约占45%，软骨肉瘤占26%，纤维肉瘤占19%。
16. 颌骨恶性肿瘤最常见的组织来源是（ ）。
    A. 非牙源性
    B. 转移癌
    C. 牙源性
    D. 神经来源
    E. 软组织来源
    参考答案：A
    【解析】颌骨恶性肿瘤有多种组织来源，但以非牙源性来源最多，其次是牙源性，另外少见的有肝、肾、肺及甲状腺等其他部位肿瘤的颌骨转移癌。
17. 下列关于涎腺恶性肿瘤不正确的说法是（ ）。
    A. 自然病程一般较短
    B. 腮腺恶性肿瘤最常见
    C. 舌下腺恶性肿瘤最少见
    D. 小涎腺癌好发于上腭
    E. 病理类型复杂多样
    参考答案：A
    【解析】涎腺恶性肿瘤的病理类型复杂多样，每种病理类型的肿瘤又各具有不同的生物学行为。在涎腺恶性肿瘤中，腮腺恶性肿瘤最常见，小涎腺次之，颌下腺较少见，舌下腺最少见。相当一部分涎腺恶性肿瘤发展较缓慢，自然病程长。
18. 腮腺恶性肿瘤最常见的临床表现是（ ）。
    A. 张口困难
    B. 面神经麻痹
    C. 声音嘶哑
    D. 耳前肿块
    E. 面部麻木
    参考答案：D
    【解析】腮腺恶性肿瘤绝大多数患者因耳下或耳前肿块就诊，有近期肿块生长加快现象，可伴有局部疼痛、面神经麻痹。
19. 颌下腺恶性肿瘤最常见的临床表现是（ ）。
    A. 皮肤破溃
    B. 颌下肿块
    C. 局部疼痛
    D. 患侧舌麻木
    E. 张口受限
    参考答案：B
    【解析】颌下腺恶性肿瘤大多数病例以颌下肿块就诊，有时可见面神经下颌缘支或舌下神经受累，半舌麻木或舌局部疼痛。
20. 颌下腺和小涎腺癌最常见的病理类型是（ ）。
    A. 腺样囊性癌
    B. 未分化癌
    C. 黏液表皮样癌
    D. 鳞癌
    E. 淋巴瘤
    参考答案：A
    【解析】腮腺恶性肿瘤以黏液表皮样癌、恶性混合瘤及腺癌较多见。颌下腺肿瘤中腺样囊性癌最常见，黏液表皮样癌次之。小涎腺癌中以腺样囊性癌最常见。
21. 下列情况应行全腮腺切除的是（ ）。
    A. 鳞癌
    B. 分化差的腺癌
    C. 未分化癌
    D. 恶性混合瘤
    E. 以上均是
    参考答案：E
    【解析】位于腮腺深叶的癌和位于腮腺浅叶的低分化黏液表皮样癌、分化差的腺癌、恶性混合瘤、鳞癌、未分化癌及腺样囊性癌，均应行保留面神经的全腮腺切除。应选E。
22. 下列关于腮腺恶性肿瘤颈淋巴结的处理原则不正确的是（ ）。
    A. 腺样囊性癌一般不行淋巴结清扫
    B. 腮腺癌伴颈淋巴结转移者应行颈淋巴结清扫
    C. 无淋巴结转移的鳞癌和低分化黏液表皮样癌不行淋巴结清扫
    D. 临床N0的未分化癌和分化差的腺癌可行选择性淋巴结清扫
    E. 伴有面神经麻痹的恶性混合瘤应行选择性淋巴结清扫
    参考答案：C
    【解析】腮腺癌合并颈淋巴结转移的病例在施行腮腺原发灶手术的同时应该行治疗性颈淋巴结清扫术。对临床N0病例，一般对鳞癌、未分化癌，低分化黏液表皮样癌及分化差的腺癌可考虑行选择性颈淋巴结清扫。对其他病理类型的腮腺癌一般不行选择性颈淋巴结清扫，但对伴有面神经麻痹的病例应行选择性颈淋巴结清扫。正确答案为C。
23. 腮腺恶性肿瘤术后放疗的适应证除外（ ）。
    A. 高分化黏液表皮样癌
    B. 病理切缘阳性或有肉眼残留者
    C. 伴淋巴结转移者
    D. 伴面神经麻痹者
    E. 复发患者
    参考答案：A
    【解析】腮腺恶性肿瘤术后放疗的适应证：病理切缘阳性或肉眼残留的病例；伴有面神经总干或颞面干或颈面干麻痹的病例；肿瘤贴近或累及颅底骨质；分化差的腺癌、恶性混合瘤、低分化黏液表皮样癌、腺样囊性癌、鳞癌和未分化癌；肿瘤贴近或与面神经粘连而行面神经保留的患者；复发性腮腺癌；伴有颈淋巴结转移的腮腺癌。答案应选择A。
24. 关于腮腺恶性肿瘤外科手术原则不正确的是（ ）。
    A. 如果肿瘤侵犯周围组织，应将受侵的和邻近组织切除
    B. 腺样囊性癌已出现肺转移者，原发灶切除术已无意义
    C. 位于腮腺浅叶的、较小的、无外侵的腺泡细胞癌，可行保留面神经的腮腺浅叶切除
    D. 腮腺深叶癌无面神经受侵者可行保留面神经的全腮腺切除
    E. 腺样囊性癌神经侵犯者应切到切缘阴性为止
    参考答案：B
    【解析】腺样囊性癌肺转移后仍可生存较长时间，在已出现肺转移时仍可行原发灶切除术。位于腮腺深叶的癌和位于腮腺浅叶的低分化黏液表皮样癌、分化差的腺癌、恶性混合瘤、鳞癌、未分化癌及腺样囊性癌，均应行保留面神经的全腮腺切除。位于腮腺浅叶的、较小的而且无外侵的高分化黏液表皮样癌及腺泡细胞癌，可行保留面神经的腮腺浅叶切除。如果肿瘤侵犯周围组织，应将肿瘤侵犯的或直接与肿瘤贴近的组织切除。腺样囊性癌局部浸润范围广，局部复发率高，对受侵的神经要切到切缘阴性为止。B项说法错误。
25. 下列不属于筛窦癌临床表现的是（ ）。
    A. 颅眶疼痛
    B. 面部麻木
    C. 复视
    D. 鼻塞
    E. 眼球移位
    参考答案：B
    【解析】筛窦癌早期症状少见，可仅有鼻出血，以后可出现鼻塞、眼球移位、突眼、复视、视力减退、颅面疼痛、鼻外形改变伴溢泪。因此答案是B。
26. 鼻腔及副鼻窦恶性肿瘤的主要诊断手段除外（ ）。
    A. 活检
    B. 磁共振成像
    C. 骨ECT
    D. CT扫描
    E. 免疫组化
    参考答案：C
    【解析】鼻腔和副鼻窦恶性肿瘤的诊断主要依据症状、X线检查、CT扫描、磁共振成像等建立临床印象后，再通过活检确定。CT是副鼻窦病变的首选检查方法，可显示累及范围和鉴别炎症、良性肿瘤和恶性肿瘤。磁共振成像能准确显示病变范围，还可以对一些病变作出鉴别，特别是肿瘤和炎症。活检可明确病理类型，诊断最准确，对治疗具有重要指导意义。应用免疫组化方法可以帮助诊断嗅神经母细胞瘤。骨ECT不是鼻腔及副鼻窦恶性肿瘤的诊断手段，答案是C。
27. 以下不属于术前放疗在鼻腔及副鼻窦恶性肿瘤治疗中作用的是（ ）。
    A. 减少出血
    B. 提高局部控制率
    C. 消灭亚临床病灶
    D. 使肿瘤缩小
    E. 减少术中播散
    参考答案：A
    【解析】术前放疗可以使肿瘤缩小，消灭肿瘤周围的亚临床病灶，减少术中肿瘤扩散几率，从而降低肿瘤局部复发率。A项不正确。
28. 喉的解剖结构描述下列不恰当的是（ ）。
    A. 主要由骨骼、黏膜和肌肉组成
    B. 解剖上分为三个区域：声门上区、声门区、声门下区
    C. 位于颈前中央，成人相当于第四至第六颈椎椎体水平
    D. 包括两个间隙：茎突前间隙和声门旁间隙
    E. 其上方与口咽相延续，下方与气管相通
    参考答案：D
    【解析】喉位于颈前中央，成人相当于第四至第六颈椎水平，喉结构主要由骨骼、黏膜和肌肉组成，其上方和口咽相延续，下方与气管相通，两侧及后方与下咽相连。解剖上分为三个区域：声门上区、声门区、声门下区。喉旁有两个间隙：会厌前间隙和声门旁间隙。
29. 关于口咽的解剖特点，下面说法正确的是（ ）。
    A. 口咽不包括舌后1/3区域
    B. 口咽介于硬腭和舌骨水平之间
    C. 口咽前壁即舌会厌区，包括舌根部和舌会厌谷，顶壁为软腭及腭垂；后壁为颈椎椎体前缘，侧壁则包括扁桃体、扁桃体窝、咽柱及舌扁桃体沟
    D. 口咽上界与鼻咽衔接，下至舌会厌谷与喉毗邻
    E. 口咽部位淋巴组织很少，因此该部位肿瘤无论分期早晚均很少出现淋巴结转移
    参考答案：C
    【解析】整个咽部由上至下通过软腭、舌骨而分为鼻咽、口咽和下咽，口咽介于软腭和舌骨之间，按照UICC（2002）TNM分期标准，口咽分为前壁、顶壁、后壁和侧壁四个解剖分区；口咽包括舌后1/3区域，舌根癌即属于口咽癌范围。口咽部淋巴组织丰富，容易出现淋巴结转移。
30. 下列关于喉的解剖不正确的是（ ）。
    A. 会厌前间隙形如倒置的椎体，上宽下窄，位于会厌之前
    B. 舌骨上会厌及舌骨下会厌均属声门上区
    C. 声门下区是指声门区以下至环状软骨下缘水平，长约2cm
    D. 声门区包括声带、前联合和后联合之间的区域
    E. 声门旁间隙位于甲状软骨板内膜和甲杓肌之间
    参考答案：D
    【解析】声门区包括声带，前、后联合及声带游离缘下0.5cm范围内的区域。
31. 下列关于喉的淋巴分布描述正确的是（ ）。
    A. 喉内浅层淋巴管有间隔，声门上与声门下不相通
    B. 声门上区淋巴管细而稀，呈单层分布
    C. 喉内浅层淋巴管有间隔，左右喉不相通
    D. 声门下区淋巴管粗而密，呈多层分布
    E. 声门下环状软骨部的血管和淋巴管为全周性交通
    参考答案：E
    【解析】声门上区淋巴管粗而密，呈多层分布；声门下区淋巴管细而稀，呈单层分布；喉内深层淋巴管有间隔，左右喉不相通，声门上与声门下不相通；声门下环状软骨部的血管和淋巴管则相互交通。
32. 口咽肿瘤最常见的颈淋巴结转移区为（ ）。
    A. Ⅰ~Ⅵ区
    B. Ⅰ区和Ⅱ区
    C. Ⅳ区和Ⅴ区
    D. Ⅱ区和Ⅲ区
    E. Ⅰ~Ⅴ区
    参考答案：D
    【解析】口咽淋巴引流丰富，容易出现淋巴结转移，其转移几率与原发肿瘤大小、分化程度和病期早晚相关。口咽部肿瘤在确诊时颈淋巴结转移的阳性率可达50%～70%。其中最常见的颈淋巴结转移区为Ⅱ和Ⅲ区淋巴结（即二腹肌下组和上颈深组淋巴结）。Ⅰ、Ⅳ、Ⅴ均较少出现转移，但在放疗时仍应包括在照射靶区内。
33. 关于喉的淋巴引流，下面叙述正确的是（ ）。
    A. 真声带基本没有毛细淋巴管
    B. 声门上、下区淋巴引流以声带为界引流至相同的淋巴结组
    C. 声门下淋巴管丰富，主要引流喉前、气管前、气管旁淋巴结
    D. 声门上区淋巴管小，汇集引流至颈上深或颈中深淋巴结
    E. 早期声带癌较易发生淋巴结转移
    参考答案：A
    【解析】声门上、下区淋巴引流以声带为界引流至不同的淋巴结组。声门上区淋巴管丰富，汇集引流至颈上深或颈中深淋巴结，声门下淋巴管相对少，主要引流喉前、气管前、气管旁淋巴结。真声带基本没有毛细淋巴管，早期声带癌甚少发生淋巴结转移。
34. 下述关于喉癌的描述不正确的是（ ）。
    A. 90%以上的喉癌可通过间接喉镜发现异常
    B. 喉癌病理类型多为鳞癌
    C. 声门癌颈部淋巴结转移多见且易早期出现
    D. 女性声门上区癌多于男性
    E. 喉癌最常见的远处转移为肺
    参考答案：C
    【解析】真声带基本没有毛细淋巴管，早期声带癌甚少发生淋巴结转移。
35. 声门上型喉癌常见的侵犯途径是（ ）。
    A. 经会厌旁间隙至声门旁间隙并发展至声门区
    B. 经会厌旁间隙至声门前间隙然后侵犯声门区
    C. 经声门上间隙至声门前间隙并发展至声门区
    D. 经声门上间隙至声门旁间隙最后侵犯声门区
    E. 经会厌前间隙至声门旁间隙并发展至声门区
    参考答案：E
    【解析】喉旁有两个间隙，会厌前间隙和声门旁间隙。它们与喉癌的局部扩散有密切的关系。声门上癌常经过会厌前间隙发展至声门旁间隙再侵犯声门区。
36. 关于声门癌的描述正确的是（ ）。
    A. 肿瘤多发生于声带的后1/3
    B. 在喉癌的发病率中居第二位，约占40%
    C. 病理类型多为低分化鳞癌
    D. 与声门上区癌相比，颈部淋巴结转移多见
    E. 临床主要表现是声嘶
    参考答案：E
    【解析】声门癌在喉癌的发病率中居首位，约占50%～60%，与声门上区癌相比，颈部淋巴结转移少，病理类型多为高分化鳞癌，肿瘤多发生于声带的前1/3～1/2，声嘶在声门癌较多见。
37. 关于口腔癌下列叙述正确的是（ ）。
    A. 口腔癌包括的解剖结构为：上、下齿龈、硬腭、口底、颊黏膜五个部分
    B. 口腔肿瘤的部位越远离唇而接近口咽，则肿瘤分化越好
    C. 口腔癌是不能治愈的
    D. 口腔肿瘤的病理类型以腺癌为主
    E. 单纯高能X线照射和高能X线加组织间近距离放疗是口腔癌放射治疗中最常用的治疗技术
    参考答案：E
    【解析】没有试题分析
38. 下面关于唇癌的描述正确的是（ ）。
    A. 上唇癌以移行细胞癌为主
    B. 好发于黑人，少见于浅肤色人种
    C. 大多数下唇癌是中等分化或分化好的鳞癌
    D. 约90%的唇癌发生于上唇
    E. 原发于皮肤且侵及唇也应诊断为唇癌
    参考答案：C
    【解析】唇癌是仅次于皮肤癌的最常见的头颈部肿瘤。好发于常年从事户外工作的浅肤色人种，以60～70岁男性多见。约90%唇癌发生于下唇，且大多数下唇癌是中等分化或分化好的鳞癌；而上唇癌以基底细胞癌多见。唇癌易早期发现，主要以局部侵犯为主，较少出现淋巴结转移。
39. 下列关于扁桃体癌叙述正确的是（ ）。
    A. 晚期扁桃体癌在治疗初始阶段，一煅采用单侧扁桃体部位的照射技术
    B. 起源于扁桃体的肿瘤95%以上为鳞癌和肉瘤
    C. 扁桃体癌的常见症状是双侧咽喉部疼痛合并声嘶，并可放射至耳根，进食或饮水时加重
    D. 扁桃体癌多数分化差，病理类型以低分化或未分化癌为主，易向邻近结构蔓延
    E. 扁桃体癌有较低的淋巴结转移率，故下颈、锁骨上区常规不预防性照射
    参考答案：D
    【解析】扁桃体癌95%以上为鳞癌和淋巴瘤，多数鳞癌分化较差，容易出现颈部淋巴结转移。临床上常见症状为一侧咽喉部疼痛；由于淋巴结转移率高，治疗上一般需做颈部和锁骨上预防性照射。
40. 从治疗效果和保留器官功能来考虑，早期头颈部肿瘤首选的治疗手段是（ ）。
    A. 生物治疗
    B. 手术治疗
    C. 化学治疗
    D. 放射治疗
    E. 加温治疗
    参考答案：D
    【解析】没有试题分析
41. 早期声门区喉癌（T1N0M0）的合理治疗手段是（ ）。
    A. 术前放疗＋手术切除＋术后同步放化疗
    B. 根治性放疗
    C. 全喉切除＋根治性放疗
    D. 仅行声带切除
    E. 手术＋术后化疗
    参考答案：B
    【解析】早期喉癌（T1～2N0）无论是手术还是放疗，其总的生存率相似。采用放疗可有效地保留患者喉功能。即使是放疗后复发，仍可行挽救性手术以获得较高的局部控制率。
42. 扁桃体癌cT2N1M0行常规根治性放射治疗，其照射野的设计正确的是（ ）。
    A. 双侧扁桃体区面颈野
    B. 同侧扁桃体区小野
    C. 同侧扁桃体区面颈野＋中、下颈及锁骨上区野
    D. 同侧扁桃体区面颈野
    E. 双侧扁桃体区面颈野＋双侧中、下颈及锁骨上区野
    参考答案：E
    【解析】扁桃体癌根治性放射治疗照射野的设计应根据原发肿瘤大小、邻近结构受侵范围、病理类型、淋巴结转移等因素来综合考虑。在常规放射治疗初始阶段一般采用面颈联合野对穿照射＋下颈、锁骨上单切线野照射，其中面颈野靶区包括原发灶、邻近组织（如颊黏膜、齿龈、鼻咽、舌根、咽侧壁等）及上中颈淋巴结引流区。若为cT1N0病变，且分化良好者，照射野可适当缩小（如V区淋巴结可不包括），甚至只需要照射瘤床区＋同侧颈淋巴引流区，但首先要明确分期，以免漏照。
43. 头颈部肿瘤放疗初期出现明显腮腺肿痛伴低热，查血未提示感染，下列处理方法合理的是（ ）。
    A. 予以热敷治疗即可
    B. 只需要简单退热处理
    C. 口含维生素C即可
    D. 尽量保持口腔清洁，予以漱口水处理，必要时加用抗生素以及暂停放疗
    E. 终止放疗，并给予全身抗炎治疗
    参考答案：D
    【解析】放疗初期出现腮腺肿痛，多考虑为急性放射性腮腺炎，一般出现在放疗的第1～3天，主要表现为一侧（少数双侧）腮腺区肿胀、疼痛，严重者局部皮肤红肿、皮温升高，少数伴有发热。其病理生理机制为放疗所致的腮腺导管上皮细胞肿胀而堵塞导管。腮腺肿痛属急性放射反应，无特效治疗手段，主要以对症治疗为主，如少吃刺激唾液腺分泌的食物、保持口腔清洁等，必要时可加用抗生素。
44. 颊黏膜高分化鳞癌cT2N0M0，如行放疗对颈部淋巴引流区的处理正确的是（ ）。
    A. 仅需预防照射同侧颌下、颈深上、二腹肌及颏下淋巴结区
    B. 颈部淋巴结区域可不行预防照射，定期观察
    C. 颈部预防性照射，放疗后行淋巴结清扫
    D. 颈部预防性照射
    E. 需预防照射双侧颌下、颈深上、二腹肌及颏下淋巴结区
    参考答案：B
    【解析】颊黏膜癌仅占口腔癌的50%，以后部直接侵犯为主，淋巴结转移在局部晚期（如T3～4）多见。关于无颈部淋巴结转移的放射野设计，一般不做颈部预防照射，除非出现以下情况：分化差的鳞癌、有深部肌肉浸润、履部骨受侵以及T3、T4病变。预防照射区坷为同侧颌下、颈深上、二腹肌及颏下淋巴结区。
45. 口腔癌2002年UICC分期中，关于N3的诊断标准是（ ）。
    A. 双侧颈淋巴结转移＜3cm但固定
    B. 对侧颈淋巴结转移但＜3cm
    C. 同侧颈淋巴结转移＞3cm，但不超过6cm
    D. 双侧颈淋巴结转移但＜3cm
    E. 颈淋巴结转移＞6cm
    参考答案：E
    【解析】没有试题分析
46. 舌活动部早期鳞癌（cT1N0M0）原发灶常规外照射射线选择（ ）。
    A. 电子线
    B. 低能X线＋电子线
    C. 快中子
    D. 高能X线＋电子线
    E. 高能X线＋快中子
    参考答案：D
    【解析】没有试题分析
47. 关于喉癌手术治疗原则，下列说法错误的是（ ）。
    A. 一般均需做颈淋巴结清扫，特别是有颈淋巴结转移者
    B. 局部晚期患者（cT3～4N1～30）经术前放疗后可行全喉切除术或根据情况保留喉功能
    C. 伴严重喉阻塞时可先行手术切除，术后再考虑行放疗或其他治疗
    D. 根治性放疗后短期内局部复发者可行手术挽救
    E. 早期声门高分化鳞癌（cT1～2）需行全喉切除术＋颈淋巴结清扫＋术后辅助放疗
    参考答案：E
    【解析】早期喉癌可单纯行放疗或手术治疗，局部晚期以综合治疗为主。早期声门癌目前根治的方法有激光治疗、声带切除术、放疗等。在选择治疗方案时，在强调肿瘤根治的同时，需权衡喉功能的保留，尽量降低复发率；避免过度治疗或出现严重并发症。
48. 下述关于喉癌放射治疗原则不正确的是（ ）。
    A. 病变侵及声门下区者行术后放疗时应避开气管造瘘口
    B. 早期声门上低分化癌或未分化癌可首选放射治疗
    C. 软骨受侵犯但无淋巴结转移的患者手术切除后仍需行术后放疗
    D. T3、T4期喉癌手术切除后需行术后放疗
    E. 病变严重阻塞气道伴呼吸困难者为放疗相对禁忌证
    参考答案：A
    【解析】术后放疗的患者若有下述的指征则气管造瘘口必须包括在照射野内：病变侵及声门下区；术前行紧急气管切开术；颈部软组织受侵；气管切缘阳性；手术切痕通过造瘘口。
49. 下述关于喉癌常规放疗描述正确的是（ ）。
    A. 第二原发肿瘤是放疗常见的急性期反应
    B. 声门癌放疗首选60Co或4MV直线加速器
    C. 放疗分次剂量宜低于2Gy，即1.8Gy/次
    D. 喉癌通常采用分段照射以减少上呼吸道和上消化道并发症
    E. 声门上型喉癌的射线能量选择对疗效影响较大
    参考答案：B
    【解析】声门癌位置较浅，且病变多位于声带前1/3～1/2，结合喉部解剖特点，放疗以60Co或4MV直线加速器为首选。喉癌通常采用常规分割治疗且分次剂量最好不要低于2Gy。对于声门上下区癌，射线能量选择对疗效影响不大。第二原发肿瘤作为放疗的晚期并发症少见。
50. 下述关于唇癌放射治疗适应证的说法不正确的是（ ）。
    A. 手术后局部复发，且不能或不愿意再次手术治疗者可行放疗
    B. 浅表的、仅占下唇1/3面积或T1病变，希望获得较好的美容效果
    C. 浸润性病变（如浸润深度超过3cm）手术治疗较难获得满意的功能和美容效果，可首先行放疗治疗
    D. 累及口角或同时累及上下唇的病变治疗上可首选放射治疗
    E. 早期唇癌（cT1～2N0M0）术后需常规补充局部放疗
    参考答案：E
    【解析】唇癌是仅次于皮肤癌的最常见的头颈部肿瘤。病理上以高分化鳞癌多见，以局部侵犯为主，早期较少出现淋巴结转移。唇癌易早期发现，一般预后较好。唇癌（特别是局部中晚期）放射治疗可取的较好的近期美容效果和功能保留。早期唇癌单纯手术或放疗均可获得较高的治愈率，对于手术切缘阳性或切缘距肿瘤＜1cm需考虑术后放疗。
51. 下列关于喉癌放疗并发症处理错误的是（ ）。
    A. 根治性放疗后3个月喉水肿持续存在，并伴声带固定，需考虑肿瘤未控可能，一经证实可行解救性喉切除术
    B. 喉轻度水肿出现时，宜及时予以超声雾化治疗
    C. 放疗中避免用声过度可减轻急性放射反应
    D. 喉软骨坏死宜首选抗炎保守治疗
    E. 放疗前或放疗中戒烟可减少并发症的发生
    参考答案：D
    【解析】喉软骨坏死一旦出现只有手术切除。
52. 影响喉癌放疗局部控制率的因素须排除的是（ ）。
    A. 症状持续时间的长短
    B. KPS评分情况
    C. 治疗前Hb（血红蛋白）的高低
    D. 放疗后合并第二原发肿瘤
    E. 肿瘤的大小和生长方式
    参考答案：D
    【解析】合并第二原发肿瘤时喉癌放疗的局部控制率无明显影响，但对总的生存影响明显。
53. 下列关于晚期喉癌靶区勾画设计原则正确的是（ ）。
    A. cT3～4N0声门癌靶区勾画只需包括双侧Ⅰ~Ⅱ区颈淋巴结
    B. 原发肿瘤的CTV为影像学所见瘤体勾画GTV外放3mm
    C. 声门上区癌N0的淋巴引流区的CTV包括双侧上、中颈淋巴结
    D. 原发肿瘤CTV外放2cm即为PTV
    E. cT3～4N2c声门上区病变的淋巴引流区的CTV包括双Ⅱ~V区颈淋巴结＋咽后淋巴结区域
    参考答案：E
    【解析】原发肿瘤的CTV为影像学所见瘤体勾画GTV外放1cm，CTV外放3～5mm即为PTV。声门上区病变的淋巴引流区的CTV包括双侧颈部淋巴结的Ⅱ~Ⅳ区，T3～4声门癌靶区勾画也应包括双侧颈部淋巴结的Ⅱ~Ⅳ区。
54. 下列关于软腭癌的放疗原则正确的是（ ）。
    A. 小涎腺来源的软腭癌，其放射敏感性较高，放疗的根治剂量为DT60Gy
    B. 无上颈部淋巴结转移的高分化鳞癌，需行中下颈淋巴结的预防性照射
    C. 低分化或未分化癌，无论上颈有无淋巴结转移，双侧中下颈及锁骨上均需做预防照射
    D. 单侧上颈部淋巴结转移的高分化鳞癌，对侧中下颈区需行预防性照射
    E. 软腭癌常规大野照射至DT36Gy时避开脊髓，DT40Gy时缩野至软腭区
    参考答案：C
    【解析】无上颈部淋巴结转移的高分化鳞癌，不必行中下颈淋巴结的预防性照射；单侧上颈部淋巴结转移的高分化鳞癌，同侧中下颈及锁骨上区需行预防性照射，对侧中下颈无需照射；小涎腺来源的软腭癌，放疗敏感性低，根治剂量为＞70Gy；软腭癌常规大野照射至DT36～40Gy时避开脊髓，DT50Gy时缩野至软腭区。
55. 下列关于舌根癌的常规放疗设计不正确的是（ ）。
    A. 原发灶水平照射野等中心位置常规约位于舌中后1/3与体中线交汇处
    B. 照射时一般采用原发灶及上颈等中心水平对穿照射，下颈锁骨上野采用前切线野照射技术
    C. 原发灶后界应至椎体前缘，避开颈静脉链淋巴结区域
    D. 原发灶上界为口含器压舌至口底时，超过舌根表面1.5～2cm，如肿瘤侵犯鼻咽，上界相应要提高
    E. 等中心水平照射野包括的靶区范围是原发灶、双侧咽后淋巴结、上颈深淋巴结、二腹肌下组淋巴结及颈后淋巴结
    参考答案：C
    【解析】原发灶后界应至椎体后缘，包括颈静脉链淋巴结区域（V区淋巴结区域）。
56. 下列关于齿龈癌常规放疗的说法不正确的是（ ）。
    A. 上齿龈癌放疗射野在包括原发灶的同时还应包括部分上颌窦
    B. 早期齿龈癌的治疗首选根治性放疗
    C. 下齿龈癌放疗射野应包括同侧下颌骨
    D. 常规放疗方式采用同侧正交楔形野照射
    E. 常规照射野前界应达下颌骨前缘，并尽可能避开上下唇
    参考答案：B
    【解析】齿龈癌早期即易出现骨受侵，由于下颌骨对射线耐受低，高剂量照射放射性骨坏死发生率高，因此早期齿龈癌放疗不作为首选治疗手段，而多采用术前放疗＋根治性手术。
57. 鼻咽咽旁间隙在解剖学上间隙可以分为（ ）。
    A. 5个间隙
    B. 2个间隙
    C. 4个间隙
    D. 3个间隙
    E. 6个间隙
    参考答案：D
    【解析】鼻咽咽旁间隙分为茎突前间隙、茎突后间隙、咽后间隙。
58. 鼻咽腔由几个壁构成？（ ）
    A. 6个壁
    B. 3个壁
    C. 5个壁
    D. 4个壁
    E. 7个壁
    参考答案：A
    【解析】鼻咽腔由前壁、后壁、顶壁、底壁、和左右侧壁6个壁构成。
59. 下咽解剖部位相当于哪两个颈椎的平面间？（ ）
    A. 颈4～颈7
    B. 颈1～颈4
    C. 颈3～颈6
    D. 颈2～颈5
    E. 颈5～颈1
    参考答案：C
    【解析】下咽是口咽的延续部分，位于喉的后方及两侧，始于咽会厌皱襞，终于环状软骨下缘，并与颈段食管入口相连，相当于第3到第6颈椎水平。
60. 鼻咽腔位于（ ）。
    A. 鼻腔后1/3处
    B. 上咽部
    C. 下咽部
    D. 中咽部
    E. 上咽部和中咽部之间
    参考答案：B
    【解析】整个咽部由上至下通过软腭、舌骨而分为鼻咽、口咽和下咽。
61. 海绵窦及周围有哪几对脑神经穿行？（ ）
    A. 4，5，6，7
    B. 1，2，3，4
    C. 3，4，5，6
    D. 2，3，4，5
    E. 5，6，7，8
    参考答案：C
    【解析】海绵窦及周围有多对脑神经（3～6）由后向前穿行。
62. 12对脑神经中在颅中窝中行程最长的是（ ）。
    A. 外展神经
    B. 视神经
    C. 三叉神经
    D. 动眼神经
    E. 面神经
    参考答案：A
    【解析】外展神经自脑桥出脑沿蝶骨大翼内侧，海绵窦下外侧前行至眶上裂出颅进入眶内，支配外直肌司眼球外展活动。它在颅中窝的行程最长，在12对脑神经中它又最纤细，所经之处是鼻咽癌上侵颅底最多发的部位，最易受到已侵入颅内的肿瘤的推压或侵袭。
63. 鼻咽茎突前间隙内通过的颅神经是（ ）。
    A. Ⅴ3颅神经
    B. Ⅱ、Ⅲ颅神经
    C. Ⅳ、Ⅴ颅神经
    D. Ⅲ、Ⅳ颅神经
    E. Ⅵ颅神经
    参考答案：A
    【解析】茎突前间隙占据咽侧间隙的茎突前部分，三叉神经下颌支自卵圆孔出颅后即在此间隙内穿行。
64. 鼻咽癌的前哨淋巴结一般认为由哪两组淋巴结组成？（ ）
    A. 上后颈和颈上深淋巴结
    B. 上颈深和上后颈淋巴结
    C. 咽后和颈上深淋巴结
    D. 上颈深和二腹肌下淋巴结
    E. 咽后和上后颈淋巴结
    参考答案：C
    【解析】鼻咽癌的前哨淋巴结一般认为是咽后淋巴结和颈上深淋巴结。
65. 下列哪项是鼻咽癌的好发部位？（ ）
    A. 咽鼓管圆枕
    B. 鼻咽顶壁
    C. 喉鼻咽后壁
    D. 咽隐窝
    E. 梨骨后缘
    参考答案：D
    【解析】咽隐窝向外侧经咽上缩肌的上缘延伸到Morgagni窦，该区是鼻咽癌最好发的部位。
66. 鼻咽癌高分化鳞癌约占总数的（ ）。
    A. 70%～80%
    B. 小于10%
    C. 50%～65%
    D. 30%～45%
    E. 85%～90%
    参考答案：B
    【解析】绝大多数鼻咽癌是低分化鳞状细胞癌和未分化癌，两者的比例占95%以上。
67. 喉的解剖位置在哪两椎体平面之间？（ ）
    A. 颈4～颈6
    B. 颈1～颈3
    C. 颈3～颈5
    D. 颈2～颈4
    E. 颈5～颈7
    参考答案：A
    【解析】喉位于颈前中央，成人相当于第4至第6颈椎椎体水平，喉结构主要由骨骼、黏膜和肌肉组成。
68. 上颌窦的淋巴引流主要到（ ）。
    A. 颏下淋巴结
    B. 上颈深淋巴结
    C. 颌下淋巴结
    D. 二腹肌后组淋巴结
    E. 上颈深＋颌下＋颏下淋巴结
    参考答案：B
    【解析】上颌窦淋巴引流至Ⅱ区淋巴结
69. 鼻腔副鼻窦癌中最多见下列哪种病理类型？（ ）
    A. 肉瘤
    B. 鳞状细胞癌
    C. 淋巴癌
    D. 腺癌
    E. 移行细胞癌
    参考答案：B
    【解析】鳞状细胞癌是鼻腔副鼻窦癌中最常见的病理类型，约占鼻腔副鼻窦肿瘤的50%。
70. 鼻腔副鼻窦恶性肿瘤中最常见的部位是（ ）。
    A. 蝶窦癌
    B. 外鼻癌
    C. 上颌窦癌
    D. 鼻腔癌
    E. 额窦癌
    参考答案：D
    【解析】鼻腔副鼻窦恶性肿瘤以鼻腔癌最多见，上颌窦癌次之，其分布为：外鼻癌4%～10%，鼻腔癌占47.9%～55.3%，上颌窦癌占34.1%～40.3%，筛窦癌4%～4.4%，蝶窦癌0.4%～2%，额窦癌1.2%。
71. 甲状腺癌病理类型中恶性度最高的是（ ）。
    A. 髓样癌
    B. 乳头状癌
    C. 未分化癌
    D. 滤泡状腺癌
    E. 以上答案都不对
    参考答案：C
    【解析】甲状腺癌病理类型中恶性度由高至低顺序为未分化癌一髓样癌一滤泡状腺癌一乳头状癌。
72. 不影响上颌窦癌预后的因素包括（ ）。
    A. 治疗的技术
    B. 淋巴结有无转移
    C. 病理类型
    D. 各壁受累情况
    E. 患者的性别
    参考答案：E
    【解析】上颌窦癌的预后与疾病的分期、病理类型、解剖、治疗的模式等成正相关，而与患者的性别无明显相关性。
73. 鼻咽癌好发于我国的哪个地区？（ ）
    A. 西北地区
    B. 华东地区
    C. 华北地区
    D. 华南地区
    E. 东北地区
    参考答案：D
    【解析】鼻咽癌的发病有明显的地域及种族差异，并存在家族高发倾向，在我国以华南及西南高发，华北及西北较少。
74. 下列属于鼻咽癌最常见首发体征的是（ ）。
    A. 鼻唇沟变浅
    B. 鼻咽肿物
    C. 眼球活动障碍
    D. 霍纳氏征
    E. 颈部肿块
    参考答案：E
    【解析】鼻咽癌颈部包块为最常见首发症状，约占40%，回吸性血涕占18.7%，耳部症状占17%。
75. 下列鼻咽癌的症状不包括（ ）。
    A. 头痛
    B. 鼻堵
    C. 耳鸣
    D. 眩晕
    E. 面部麻木
    参考答案：D
    【解析】没有试题分析
76. 鼻咽癌患者张口困难提示肿瘤最大可能已侵犯的部位是（ ）。
    A. 翼内肌，翼外肌
    B. 二腹肌
    C. 腭帆张肌，腭帆提肌
    D. 颞肌
    E. 胸锁乳突肌
    参考答案：A
    【解析】张口困难为晚期症状，一般为肿瘤侵犯翼内肌、翼外肌及翼腭窝。
77. 鼻咽癌患者有舌下神经麻痹，表明肿瘤已侵至（ ）。
    A. 咽后间隙
    B. 鼻腔
    C. 茎突后间隙
    D. 口咽
    E. 前颅凹
    参考答案：C
    【解析】茎突后间隙自内而外有颈内动脉，9～12对脑神经，交感神经节，颈内静脉及颈静脉淋巴链在此穿行。临床上可有因静脉回流不畅所致的搏动性头痛，9～12对脑神经麻痹及交感神经麻痹。
78. 鼻咽癌颅底骨质破坏，海绵窦受侵时最易影响的颅神经是（ ）。
    A. 动眼神经
    B. 外展神经
    C. 滑车神经
    D. 三叉神经
    E. 视神经
    参考答案：B
    【解析】外展神经自脑桥出脑沿蝶骨大翼内侧，海绵窦下外侧前行至眶上裂出颅进入眶内，支配外直肌同眼球外展活动。它在颅中窝的行程最长，在12对脑神经中它又最纤细，所经之处是鼻咽癌上侵颅底最多发的部位，最易受到已侵入颅内的肿瘤的推压或侵袭。
79. 鼻咽癌有一侧第Ⅲ、Ⅳ、Ⅵ与Ⅵ的第一分支颅神经麻痹，该综合征叫做（ ）。
    A. 岩蝶综合征
    B. 眶尖综合征
    C. 垂体蝶窦综合征
    D. 眶上裂综合征
    E. 颈静脉孔综合征
    参考答案：D
    【解析】鼻咽癌Ⅲ、Ⅳ、Ⅵ与Ⅵ颅神经麻痹，患者眼球固定，微外突，称之为眶上裂综合征，因此正确的答案选择应当为D。
80. 鼻咽癌患者出现软腭麻痹，提示肿瘤最大可能已侵犯的神经是（ ）。
    A. 翼内肌，翼外肌
    B. 舌下神经
    C. 面神经
    D. 副神经
    E. 腭帆张肌，腭帆提肌
    参考答案：E
    【解析】因鼻咽部肿瘤侵犯耳咽管周围，造成腭帆张肌、腭帆提肌功能损害以至于软腭上提不能。这是周围肿瘤浸润所致，而非神经侵犯所致。
81. 鼻咽癌就诊时上颈部有转移淋巴结发生的几率约为（ ）。
    A. 60%～80%
    B. 10%左右
    C. 40%左右
    D. 30%左右
    E. 100%
    参考答案：A
    【解析】鼻咽癌淋巴结转移发生率高，初诊时以颈部肿块为主诉的达40%～50%左右，检查发现颈部淋巴结有转移达70%上下。
82. 鼻咽癌出现面部麻木常表明（ ）。
    A. 三叉神经受侵
    B. 鼻腔受侵
    C. 眼眶受侵
    D. 筛窦受侵
    E. 颅内受侵
    参考答案：A
    【解析】鼻咽癌约15%～27%患者有面部麻木症状，这是三叉神经受侵或受压所致的浅感觉异常，包括三叉神经分布区皮肤蚁爬感、触觉过敏或麻木，是鼻咽癌前组脑神经受损发生率最高的症状。
83. 鼻咽癌患者临床出现Horner's征是由于哪支神经麻痹？（ ）
    A. Ⅺ
    B. Ⅸ
    C. 颈交感神经
    D. X
    E. Ⅻ
    参考答案：C
    【解析】没有试题分析
84. 鼻咽癌最常见的颈淋巴结转移部位是（ ）。
    A. 颌下淋巴结
    B. 颈后淋巴结
    C. 咽后淋巴结
    D. 颈深上淋巴结
    E. 耳前淋巴结
    参考答案：D
    【解析】鼻咽癌最常见的颈淋巴结转移部位是颈深上淋巴结，其次为颈后淋巴结和咽后淋巴结。
85. 下列哪项是鼻咽癌患者出现发作性突然晕厥可能引起的原因？（ ）
    A. 颌下淋巴结转移
    B. 上后颈淋巴结转移
    C. 颈深上淋巴结转移
    D. 中后颈淋巴结转移
    E. 二腹肌下淋巴结转移
    参考答案：C
    【解析】颈深上组淋巴结转移压迫或侵犯颈静脉窦而致颈静脉窦过敏综合征，表现为发作性突然晕厥，有多次发作提示预后不良。
86. 上颌窦肿瘤侵犯后壁表现包括（ ）。
    A. 眼球胀痛，复视等
    B. 血涕，鼻堵等
    C. 面部疼痛等
    D. 牙痛，牙齿松动等
    E. 颞部疼痛，张口困难等
    参考答案：E
    【解析】上颌窦肿瘤侵犯内侧表现为血涕、鼻堵等，侵犯底壁表现为牙痛、牙齿松动等，侵犯前壁表现为面部疼痛等，侵犯顶壁表现为眼球胀痛、复视等，侵犯后壁表现为颞部疼痛、张口困难等。
87. 下列哪项检查对鼻咽癌的确诊是必须的？（ ）
    A. 鼻咽部活检
    B. 鼻咽镜检查
    C. 颈部肿块穿刺细胞学
    D. CT扫描
    E. EB病毒血清学检查
    参考答案：A
    【解析】肿瘤的诊断分为5级，而活检组织病理学则是最高级别的诊断，也是最准确及最有价值的。
88. 下列哪项是诊断鼻咽癌必不可少的最基本的检查？（ ）
    A. EB病毒血清学
    B. CT
    C. 鼻咽镜加活检
    D. MRI
    E. X线平片检查
    参考答案：C
    【解析】鼻咽镜加活检可以获得最主要的病理学诊断依据。
89. 没有远处器官转移的鼻咽癌首选下列治疗方法中的（ ）。
    A. 中药治疗
    B. 手术治疗
    C. 放疗
    D. 化疗
    E. 化疗＋手术
    参考答案：C
    【解析】目前鼻咽癌公认和有效的根治性治疗手段为放疗，或以放疗为主的综合治疗。
90. 下列哪项是上颌窦癌放射治疗失败的主要原因？（ ）
    A. 局部未控或局部复发
    B. 局部复发＋淋巴结转移
    C. 淋巴结转移＋远地转移
    D. 局部复发＋远地转移
    E. 局部未控＋远地转移
    参考答案：A
    【解析】上颌窦癌局部复发率约45.2%～60%，是治疗的主要失败原因，其次是淋巴结转移和远地转移。
91. 鼻咽癌临床分期是T2～4N＋M0，常规放疗第一阶段布野较好的方法是（ ）。
    A. 面前品字野＋耳后野
    B. 耳前野＋全颈切线野
    C. 面颈联合野＋双下颈锁骨上切线野
    D. 耳前野＋面前品字野＋全颈切线野
    E. 面前品字野＋耳后野＋全颈切线野
    参考答案：C
    【解析】没有试题分析
92. 鼻咽癌以低分化癌为多，常规分割放射治疗剂量选择为（ ）。
    A. 2.5Gy/次
    B. 1.5Gy/次
    C. 2.0Gy/次
    D. 1.7Gy/次
    E. 3.0Gy/次
    参考答案：C
    【解析】鼻咽癌放射治疗最常用的剂量分割方法，即5次/周，DT1.8～2.0Gy/次。
93. 鼻咽癌放射治疗后低头时出现一过性腰、骶及下肢闪电感，其原因是（ ）。
    A. 早期放射性垂体反应
    B. 早期放射性颞叶反应
    C. 早期放射性颈段脊髓反应
    D. 早期放射性小脑反应
    E. 放射性椎体脱钙
    参考答案：C
    【解析】颈段脊髓急性放疗后因为肿胀、缺血等出现感觉障碍，常表现为麻木、蚁行感、闪电感、温觉异常等可逆性改变。
94. 近距离腔内治疗鼻咽癌适应证包括（ ）。
    A. 口咽受侵
    B. 限于鼻咽壁、浅表病灶
    C. 咽旁间隙明显受侵
    D. 鼻腔受侵
    E. 颅底骨破坏
    参考答案：B
    【解析】近距离腔内治疗鼻咽癌的适应证包括初程根治性放疗的T1、T2早期病变，可计划性外照射DT56～60Gy后加腔内治疗等。
95. 鼻咽癌调强放射治疗中CTV1一般定义为（ ）。
    A. 原发灶区
    B. 淋巴结引流区
    C. 高危区
    D. 低危区
    E. 计划靶区
    参考答案：C
    【解析】对鼻咽癌而言，根据受累的危险程度不同，可以定义CTV1代表高危区，CTV2代表低危区。
96. 鼻咽癌高剂量率近距离腔内后装治疗与外照射协同使用时应（ ）。
    A. 外照射至DT50～60Gy时，对残余表浅、小病灶后装推量治疗
    B. 与外照射同时进行
    C. 外照射DT30Gy后加用后装治疗
    D. 外照射DT10Gy后加用后装治疗
    E. 外照射至根治量以上，再加用高剂量后装治疗
    参考答案：A
    【解析】没有试题分析
97. 鼻咽癌原发灶手术适应证包括（ ）。
    A. 伴颅内海绵窦受侵
    B. 早期鼻咽癌
    C. 伴蝶、筛窦受侵
    D. 伴有鼻腔或上颌窦受侵
    E. 放射治疗后鼻咽腔残存或复发，无颅底破坏、无茎突后受侵
    参考答案：E
    【解析】鼻咽癌首程根治性放疗结束后仍有10%肿瘤残留，可考虑外科解救性治疗，手术适应证包括全身状况好，放射治疗后鼻咽部或颈部未控或复发，无颅底骨质破坏或颅内受侵。
98. 一鼻咽癌肿瘤延至口咽，上颈有转移淋巴结，最初照射野最好采用的是（ ）。
    A. 双耳前野＋面前品字野＋颈部切线野
    B. 双耳前野＋双颈垂直野
    C. 面颈联合野＋下颈锁骨上切线野
    D. 双耳前野＋颈切线野
    E. 双耳前野＋双耳后野＋颈部切线野
    参考答案：C
    【解析】鼻咽癌放射治疗常规推荐采用面颈联合野＋下颈锁骨上切线野
99. 鼻咽癌放疗原发灶根治剂量一般应给予（ ）。
    A. 70Gy
    B. 30Gy
    C. 50Gy
    D. 40Gy
    E. 80Gy
    参考答案：A
    【解析】鼻咽癌常规放射治疗根治剂量为DT70～72Gy/35～40次/7～8周。
100. 鼻咽癌颈转移性淋巴结治疗应选（ ）。
     A. 高剂量率近距离组织间治疗
     B. 单纯高能射线外照射
     C. 单纯低能射线外照射
     D. 高能X射线与低能X射线或电子线相结合
     E. 快中子照射
     参考答案：D
     【解析】高能X射线与电子线各自具有不同的物理学特点，高能X射线表面剂量比较低，随着深度的增加，深度剂量逐渐增加，直至达到最大剂量点。低能X射线或电子线最大剂量点基本位于或接近模体表面，随着深度的增加，深度剂量逐渐减少。两者结合，取长补短，可使放疗达到合理的剂量分布。
101. 鼻咽癌耳前野摆位时，下列部位不需挡铅的是（ ）。
     A. 颅底
     B. 眼睛
     C. 脑干
     D. 喉
     E. 脊髓
     参考答案：A
     【解析】正常骨TD50/5的剂量为10000cGy，高于鼻咽癌的放疗剂量。
102. 鼻咽癌根治性放射治疗最常见的并发症是（ ）。
     A. 后组颅神经损伤
     B. 皮肤色素沉着
     C. 听力减退
     D. 口干
     E. 涕多
     参考答案：D
     【解析】鼻咽癌根治性放射治疗最常见的并发症有口干、张口困难、脑坏死、骨坏死、听力下降等，其中最常见的有口干及张口困难。
103. 鼻咽部多形T细胞非霍奇金淋巴瘤临床分期ⅡA最佳治疗方式是（ ）。
     A. 免疫治疗
     B. 手术
     C. 单纯化疗
     D. 放化疗综合治疗
     E. 基因治疗
     参考答案：D
     【解析】鼻咽部多形T细胞非霍奇金淋巴瘤临床分期ⅡA期推荐治疗方式为放化疗综合治疗模式。
104. 上颌窦癌的治疗原则近年来通常采用（ ）。
     A. 手术＋化疗
     B. 手术＋放疗
     C. 化疗＋手术
     D. 放疗＋手术
     E. 动脉化疗＋开窗刮除肿瘤
     参考答案：B
     【解析】上颌窦癌的主要治疗模式是以手术＋放疗为主的综合治疗。
105. 局限期的上颌窦癌较合理的照射野布野方法（ ）。
     A. 双侧相对野加患侧面前野
     B. 单一患侧面前野
     C. 患侧面前、侧野两野成角加楔形板照射
     D. 单一患侧侧野
     E. 双侧相对野
     参考答案：C
     【解析】鼻腔副鼻窦癌普通常规外照射可采用一前一侧野，两前斜野或两侧野加一前野，筛窦及眼眶区补电子线小野等布野方式，同时加楔形板，等中心照射。
106. 上颌窦癌有眶底骨壁或后壁破坏术前放疗剂量（ ）。
     A. 8000cGy
     B. 3000cGy
     C. 6000cGy
     D. 4000cGy
     E. 9000cGy
     参考答案：C
     【解析】头颈部肿瘤一般情况下术前放疗的剂量为50～60Gy/5～6周。
107. 鼻咽癌放疗的面颈联合野在达到36～40Gy时需要分野，其主要原因是（ ）。
     A. 避免过重的口腔反应
     B. 保护腮腺
     C. 保护脊髓
     D. 保护颞颌关节
     E. 保护喉头
     参考答案：C
     【解析】鼻咽癌面颈联合野放疗在第一阶段达到36～40Gy时，进行分野，耳后给予电子野，利用电子野的剂量分布优势保护脊髓，避免脊髓在放疗期间受量超过其最高限量45Gy。
108. 头颈部肿瘤放疗中出现明显腮腺肿痛时正确的处理（ ）。
     A. 予以热敷治疗即可
     B. 不必停止放疗与做特殊处理
     C. 口含维生素C即可
     D. 保持口腔清洁，给予小剂量激素处理
     E. 除口腔清洁卫生外，应予以全身抗炎并暂停放疗
     参考答案：D
     【解析】头颈部肿瘤放疗中出现明显腮腺肿痛为放疗性水肿所致，予以漱口水保持口腔清洁并予以小剂量激素处理。
109. 全中枢神经系统放射治疗野间隔的宽度最合理的处理方法是（ ）。
     A. 根据SSD射野长度和病灶深度计算间隔的宽度
     B. 不设间隔以防病灶遗漏
     C. 间隔1cm，每照射1000cGy，上下移动一次交接处
     D. 不设间隔但每照射1000cGy上下移动一次交接处
     E. 间隔1cm或以上，以防照射区重叠造成脊髓损伤
     参考答案：C
     【解析】全中枢神经系统放射治疗野间隔的宽度最合理的处理方法为间隔1cm，每照射1000cGy，上下移动一次交接处，可减少冷点及热点的产生，放疗剂量分布合理。
110. 早期下咽癌首选的治疗方法是（ ）。
     A. 热疗
     B. 手术治疗
     C. 化学治疗
     D. 放射治疗
     E. 分子靶向治疗
     参考答案：D
     【解析】下咽解剖位置较特殊，手术与放疗在早期下咽癌治疗中的效果基本相似，但放射治疗能保证下咽，喉等器官的解剖结构的完整性，又可将易发生转移的部位包括在照射野内。因此，早期下咽癌的治疗还是以放疗占优势，应该首选放射治疗。
111. 患者男性，64岁，反复鼻出血3月，伴头痛耳鸣月余，患者行MR图像如下，最有可能的诊断是（ ）。
     A. 鼻咽纤维血管瘤
     B. 恶性淋巴瘤
     C. 鼻咽癌
     D. 颅咽管瘤
     E. 慢性鼻咽炎
     参考答案：C
     【解析】鼻咽部6cm肿块，T1WI低信号，T2WI等信号，颈部淋巴结明显广泛肿大。淋巴瘤鼻咽部一般无肿块，颅咽管瘤多发生在鞍区，鼻咽纤维血管瘤颈部淋巴结不大。
112. 患者男，56岁，因"反复鼻腔血涕伴鼻塞6月余"就诊，体检发现左鼻腔新生物，CT示左鼻腔占位性病变，鼻中隔左上颌窦内侧壁受侵。该患者最有可能的诊断是（ ）。
     A. 嗅神经母细胞瘤
     B. 鼻腔淋巴瘤
     C. 鼻腔癌
     D. 上颌窦癌
     E. 鼻腔腺样囊性癌
     参考答案：C
     【解析】反复出现血性分泌物和鼻腔肿块是鼻腔癌的较早期症状，疼痛也可见于较早期。鼻塞为最多见的症状。结合患者体检发现左鼻腔新生物，CT显示肿块主要位于左鼻腔，伴鼻中隔和左上颌窦内侧壁受侵，最佳诊断为鼻腔癌。
113. 患者女性，42岁，3月前感觉右侧面部肿胀，近2周来出现右面部疼痛、右鼻塞，伴鼻出血、右耳听力下降，该患者正确的诊断是（ ）。
     A. 鼻咽癌
     B. 鼻腔淋巴瘤
     C. 鼻腔癌
     D. 上颌窦癌
     E. 腮腺癌
     参考答案：D
     【解析】面部肿胀是上颌窦癌最早出现的症状，其次为颅面疼痛、鼻塞，其他症状包括鼻出血、脓涕、眼球移位、面部麻木、开口困难、牙齿松动等，甚至出现耳鸣、听力减退等肿瘤侵犯鼻咽部症状。鼻腔淋巴瘤、鼻腔癌和鼻咽癌很少出现面部肿胀和面部疼痛，腮腺癌多以耳前或耳下肿块就诊，较少出现鼻塞、鼻出血。因此诊断应为上颌窦癌。
114. 患者男性，59岁，一侧咽喉部疼痛半年，放射至耳部，进食或饮水时疼痛加重，张口困难两天，体检发现右侧扁桃体区新生物。首先考虑可能是（ ）。
     A. 舌癌
     B. 扁桃体癌
     C. 硬腭癌
     D. 颊黏膜癌
     E. 口底癌
     参考答案：B
     【解析】咽喉部疼痛伴放射痛，进食加重，合并张口困难属典型的扁桃体癌症状。
115. 患者，女，35岁，扁桃体癌根治性放射治疗后半年出现局部复发，此时应首先考虑下列治疗方法中的（ ）。
     A. 免疫治疗
     B. 再次放射治疗
     C. 化学治疗
     D. 手术治疗
     E. 激光治疗
     参考答案：D
     【解析】扁桃体癌放疗后复发，优先考虑手术切除，再根据手术情况和病变范围考虑后续治疗。
116. 患者男性，50岁，发现右侧舌外侧缘溃疡半年，近一周出现右侧耳痛，伴颈部淋巴结肿大，完善检查后临床诊断为舌癌CT3N1M0，正确的处理原则是（ ）。
     A. 姑息性放疗
     B. 单纯手术
     C. 术前同步放化疗＋手术
     D. 根治性放射治疗
     E. 化学治疗
     参考答案：C
     【解析】局部晚期舌癌需要综合治疗，单纯手术或放疗疗效均低于联合治疗。
117. 男，67岁，10年前因声嘶，检查诊断为声带癌，行根治性放疗。近2月患者再次出现声嘶，伴胸闷、咳嗽、痰中带血。既往有吸烟史40年。查体：右锁骨上可及一大小约1.5cm×1cm肿块，固定无压痛。行直接喉镜发现左侧声带新生物，病检提示高分化鳞癌。胸部CT提示右下肺可见一5cm×5cm肿块，边缘可见毛刺，右肺门及纵隔淋巴结肿大明显，纤维支气管镜病检证实为低分化腺癌，下述关于该患者诊断较全面的是（ ）。
     A. 声带癌局部复发并肺转移
     B. 声带癌局部复发
     C. 声带癌局部复发合并第二原发肿瘤（肺癌）
     D. 肺癌Ⅳ期（左声带转移）
     E. 原发灶不明转移癌（左声带，肺，淋巴结）
     参考答案：C
     【解析】现有文献报道喉癌合并第二原发肿瘤发生率20%左右，且随着放疗后生存时间的延长而增加。发生第二原发肿瘤常见部位依次为肺和头颈部。
118. 男，62岁，半年前发现舌左侧缘约0.5cm×0.5cm浅表溃疡，进行性增大，并逐渐变硬，固定，颈部可及肿大淋巴结。行舌部活检示，中分化鳞癌，口咽＋颈部MR提示舌左侧缘肿块约3cm，侵及颏舌肌，左上颈可见多个淋巴结肿大明显，最大径约3.5cm。其他辅助检查未见异常。则患者目前的临床分期属于（ ）。
     A. cT4N2MO
     B. cT1N1M0
     C. cT3N2MO
     D. cT2N1MO
     E. cT4N3M0
     参考答案：A
     【解析】该题主要针对口腔癌AJCC（2002）分期，舌癌一旦出现肿块固定，往往提示深部肌肉受侵可能，局部病期较晚。
119. 男，69岁，声门上低分化鳞癌T2N0M0，根治性放疗后4月再次出现咽痛，目前适宜下列的处理方法是（ ）。
     A. 声带剥脱术
     B. 抗生素治疗＋激素
     C. 全身化疗
     D. 立即行喉切除术
     E. 激光治疗
     参考答案：B
     【解析】喉水肿的观察期限以6个月为限。放疗3月后出现水肿，如用抗生素治疗消失提示水肿可能为非肿瘤因素；若抗炎治疗无效，则需高度警惕肿瘤可能，如明确肿瘤可考虑手术治疗。
120. 女，43岁，教师。左侧声带中1/3部位原位癌行声带剥脱术后2年再次出现声嘶。喉镜示左声带黏膜稍隆起，活检示高分化鳞癌侵及黏膜，黏膜下无浸润。下一步治疗首选（ ）。
     A. 根治性放疗
     B. 再次行声带剥脱术
     C. 声带切除术
     D. 激光治疗
     E. 全身化疗
     参考答案：A
     【解析】声带原位癌主要有两种治疗手段：声带剥脱术和放射治疗。前者术后复发几率较高。反复的声带剥脱术可造成声带增厚，发音质量明显变差；结合患者年龄及工作性质，根治性放疗为此时的首选治疗。
121. 男，57岁，声嘶伴咽部不适半年。查体：左颈部可及一质硬固定包块，无压痛。最有可能是（ ）。
     A. 声门癌
     B. 鼻咽癌
     C. 扁桃体癌
     D. 声带息肉
     E. 喉结核
     参考答案：A
     【解析】声门癌的典型症状。
122. 女性，54岁，因吞咽疼痛行喉镜发现梨状窝新生物，取部分新生物送检，则病检中不可能存在下列哪种原发肿瘤组织学类型？（ ）
     A. 黑色素瘤
     B. 鳞癌
     C. 软组织肉瘤
     D. 恶性淋巴瘤
     E. 无性细胞瘤
     参考答案：E
     【解析】下咽癌95%以上为鳞癌，且其分化程度较低。少见的病理类型有小涎腺来源的腺癌，以及恶性黑色素瘤、恶性淋巴瘤和软组织肉瘤，偶可见转移性肿瘤。
123. 男性，60岁，进行性声嘶半年，入院行全面检查后确诊为晚期声门癌，问下列哪项症状晚期声门癌一般不会出现？（ ）
     A. 复视
     B. 痰中带血
     C. 喉摩擦音消失
     D. 呼吸困难
     E. 左颈部坚硬、固定的肿大淋巴结
     参考答案：A
     【解析】痰中带血、呼吸困难、喉摩擦音消失、左颈部有坚硬、不可移动的肿大淋巴结为声门癌的典型表现。而复视提示颅神经受侵，这在晚期喉癌甚少出现。
124. 某鼻咽癌患者放疗期间出现鼻咽大出血，此时其最好的急诊止血措施是（ ）。
     A. 气管插管预防窒息
     B. 加大放疗剂量止血
     C. 鼻咽塞子填塞压迫
     D. 使用强效止血药
     E. 配合大剂量化疗
     参考答案：C
     【解析】鼻腔血管比较丰富，鼻咽癌在放疗期间出现大出血，放疗、止血药等都是止血方法，但作为急诊止血方法仍以填塞压迫止血法为最好。
125. 男性，33岁，回吸性血涕半年，CT示鼻咽右侧壁增厚，咽隐窝消失，双侧咽后及双颈深淋巴结肿大，淋巴结有边缘强化、内部坏死征象。最有可能的诊断是（ ）。
     A. 脊索瘤
     B. 鼻咽癌
     C. 淋巴瘤
     D. 鼻咽腺样体增殖
     E. 恶性纤维组织细胞瘤
     参考答案：B
     【解析】患者的临床症状及影像学检查均符合鼻咽癌有关临床表现及特点，并且鼻咽癌是常见肿瘤之一。因此正确的答案选择应当为B，而其他疾病的临床表现不能与信息中的特点完全吻合。
126. 男性，32岁，右颈肿物1个月，CT扫描示右侧口咽及鼻咽软组织肿物，密度均匀，双侧颈部及纵隔有多个肿大淋巴结，边界清楚，最有可能的诊断是（ ）。
     A. 结核
     B. 鼻咽癌侵及口咽伴淋巴结转移
     C. 纤维肉瘤侵及鼻咽、口咽伴淋巴结转移
     D. 恶性纤维组织细胞瘤侵及鼻咽、口咽伴淋巴结转移
     E. 淋巴瘤
     参考答案：B
     【解析】没有试题分析
127. 患者，男，51岁，因"鼻塞伴头痛半年"就诊，鼻咽镜检查发现右侧鼻咽部黏膜隆起明显，行活检明确为鼻咽腺样囊性癌，下面说法正确的是（ ）。
     A. 由于该类型放疗所需总剂量高，一般要达到80Gy左右，因此选择IMRI治疗更有利于保护重要组织器官，同时在射野上尽量避开颅神经及其走行部位
     B. 鼻咽腺样囊性癌倾向于局部浸润生长，较鼻咽低分化鳞癌更容易出现局部淋巴结转移
     C. 该患者应该进一步行相关检查（如CT/MR）明确分期，如局部病变范围广，放疗仍然是主要的治疗手段
     D. 该类型鼻咽癌多数对放疗不敏感，所以该患者应该以手术＋化疗的模式治疗
     E. 腺样囊性癌预后极差，绝大部分患者在确诊后1年死亡
     参考答案：C
     【解析】鼻咽腺样囊性癌发病率低，不到鼻咽原发肿瘤的1%。其生物学行为倾向于局部浸润，多在鼻咽黏膜下形成隆起，易沿神经鞘膜及血管外膜侵犯到远隔部位，其淋巴结转移率较低分化鳞癌低。治疗上局部小病变可考虑手术＋放射治疗，如病变范围广，仍首选放疗。由于其易沿神经鞘膜浸润生长，故放疗射野应包括颅底及颅神经走行部位。该肿瘤发展较缓慢，总体预后相对较好。部分患者可带瘤长期生存。

共享题干题

【题干】患者男性，27岁，因"左下颌部肿痛3个月余"入院，查体：左下颌角肿胀，左面部浅静脉怒张，皮肤表面温度升高；X线片示左下颌骨质破坏，血液碱性磷酸酶升高。

1. 【单项选择题】关于该疾病下列正确的是（ ）。
   A. 皮肤破溃少见
   B. 好发于中年患者
   C. 易发生淋巴结转移
   D. 男女发病率相似
   E. 碱性磷酸酶越高预后越好
   参考答案：A
   【解析】颌骨骨肉瘤好发于30岁左右青年，男性较女性多见。由于肿瘤不断生长，可见面部皮肤静脉怒张，皮肤表面温度升高，皮肤绷紧发亮，但鲜见皮肤穿破溃烂。血液碱性磷酸酶具有重要诊断意义，其含量越高则预后越差。
2. 【单项选择题】该患者最可能是的诊断是（ ）。
   A. 下颌骨骨髓炎
   B. 下颌骨骨肉瘤
   C. 下颌骨纤维肉瘤
   D. 下颌骨软骨肉瘤
   E. 下颌骨结核
   参考答案：B
   【解析】该患者为青年男性，左下颌角肿胀，左面部浅静脉怒张，皮肤表面温度升高，X线片示左下颌骨质破坏，血液碱性磷酸酶升高，符合骨肉瘤表现。
3. 【单项选择题】该患者的最佳治疗为（ ）。
   A. 免疫治疗
   B. 放射治疗
   C. 化学治疗
   D. 外科手术
   E. 中药治疗
   参考答案：D
   【解析】根治性外科切除一直是治疗颌骨骨肉瘤的最有效方法。

【题干】患者男性，60岁，因"声音嘶哑半月余伴咽痛"就诊，查体发现颈部可及2cm×4cm大小肿大淋巴结，颈部MRI提示①右侧梨状窝新生物，侵及右侧声带，环状软骨部分受侵；②双侧颈部多个肿大淋巴结，最大径＜6cm。

1. 【单项选择题】患者经病检确诊为梨状窝低分化鳞癌，其他辅助检查未见远处转移情况。则考虑分期为（ ）。
   A. cT4N2M0
   B. cT1N1M0
   C. cT3N2M0
   D. cT2N1MO
   E. cT4N3M0
   参考答案：A
   【解析】梨状窝是下咽癌最常见的好发部位。MRI检查已经提示肿瘤侵犯环状软骨，双侧颈部多个肿大淋巴结，但最大径未超过6cm。按照UIOC（2002）分期应为cT4aN2M0。
2. 【单项选择题】下面检查对进一步确诊意义最大的是（ ）。
   A. 痰细胞学
   B. 颈部淋巴结穿刺细胞学
   C. 颈部B超
   D. 纤维喉镜检查＋病检
   E. 颈部CT
   参考答案：D
   【解析】没有试题分析
3. 【单项选择题】下一步治疗方案选择，你认为不合理的是（ ）。
   A. 术前诱导化疗＋手术＋术后放疗
   B. 手术＋术后放疗
   C. 术前同步放化疗＋手术
   D. 术前放疗＋手术
   E. 单纯化疗
   参考答案：E
   【解析】下咽癌有如下特点：局部病变易沿黏膜下广泛侵犯；淋巴管网丰富，容易发生淋巴结转移；早期症状不典型。故对局部晚期病变，无论是单纯手术还是单纯放疗，总的效果均不理想，5年生存率20%左右。目前采用手术＋放疗的综合治疗模式明显提高局部控制率和无瘤生存率。
4. 【单项选择题】关于该患者术前常规放射治疗，下面说法不正确的是（ ）。
   A. 常规照射至36Gy一40Gy时，水平野后界应适当前移以避开脊髓，颈后淋巴引流区可选用电子线补量
   B. 上界一般平颅底水平，下界达食管入口（相当于环状软骨下缘水平），瘤床区包括鼻咽、口咽、咽旁间隙、下咽部、喉部及颈段食管入口
   C. 单纯术前放射治疗剂量一般为50Gy，若为放化疗同步则剂量可减至45Gy左右
   D. 区域淋巴结照射包括双侧颈部Ⅰb～V淋巴引流区、双侧咽后淋巴结；下颈及锁骨上淋巴引流区做预防照射。因此该患者水平照射野后界置于颈椎棘突
   E. 对于面颈联合水平野和下颈切线野的衔接，可选择半野衔接或定期调整衔接部位来减少剂量热点或冷点
   参考答案：D
   【解析】该患者颈部淋巴结阳性，其照射靶区应包括双侧颈部V区淋巴结，因此面颈联合水平野后界应该置于颈后皮下1cm或直接放空。如果颈淋巴结无转移，则水平照射野后界置于颈椎棘突即可。

【题干】患者男性，59岁，声嘶伴咽部不适半年，痰中带血1月。查体：甲状软骨膨大，右上颈部（11区）可及大小约2cm×2.5cm质韧固定包块，无压痛。既往否认结核病史。患者行颈部MRI提示肿瘤位于声门上区，并侵及会厌前间隙、左侧咽壁、会厌及甲状软骨，左侧声带固定，口咽及咽旁无侵犯，上颈可见多个肿大淋巴结。直接喉镜病检提示声门上区中分化鳞癌。其他辅助检查未见转移。

1. 【单项选择题】该患者术前放疗若采用适形放射治疗或IMRT，关于淋巴结区域照射范围下面说法正确的是（ ）。
   A. 照射范围包括右侧I～V区淋巴结区域＋咽后淋巴结
   B. 患者同侧Ⅱ区淋巴结阳性，CTV无需包括同侧下颌下淋巴结（即I区）
   C. 照射范围包括双侧I～V区淋巴结区域＋咽后淋巴结
   D. 咽后淋巴结不做预防照射
   E. 上纵隔区淋巴结也需做预防照射
   参考答案：C
   【解析】适形放疗或IMRT均需对照射靶区做详细的规定。该患者局部肿瘤外侵明显，右侧Ⅱ区淋巴结已明确转移，因此可能累及的高危淋巴结区域应包括双侧下颌下淋巴结（1区）、Ⅱ~V区颈淋巴结及咽后淋巴结。
2. 【单项选择题】若行常规术前放疗，则下面说法错误的是（ ）。
   A. DT40～50Gy时复查，如肿瘤消退满意可改行根治性放疗
   B. 照射靶区应包括原发肿瘤区＋区域淋巴结引流区
   C. 必须行上、中颈淋巴结引流区照射，而下颈、锁骨上区不做预防照射
   D. 常规双侧水平野的上界平第一颈椎水平，下界为环状软骨下缘，前界为颈前缘，后界应为棘突后缘
   E. 照射剂量接近36～40Gy时应避开颈段脊髓，颈后区辅以电子线照射补量
   参考答案：C
   【解析】局部晚期喉癌（声门上型）颈部淋巴结转移多见，可高达50%。因此照射野的设计以充分包括原发灶颈部淋巴结引流区。若上、中颈淋巴结阳性，双侧下颈、锁骨上均需作预防性照射。
3. 【单项选择题】根据相关检查，患者临床分期属于（ ）。
   A. cT4N2M0
   B. cT1NlM0
   C. cT3N2M0
   D. cT2N1M0
   E. cT4N3M0
   参考答案：C
   【解析】没有试题分析

【题干】患者男性，45岁，吸烟20年，声嘶1个月余，伴喉部异物感，咽部疼痛。体检：PS=1，颈部浅表淋巴结未及肿大，扁桃体不大，咽后壁充血明显。间接喉镜发现右侧声带活动受限，局部似可见新生物。

1. 【单项选择题】患者因工作原因要求尽量恢复发音，应选择的合理治疗手段是（ ）。
   A. 垂直半喉切除术
   B. 手术十术后放射治疗
   C. 术前放疗＋手术＋术后辅助化疗
   D. 单纯化疗
   E. 根治性放疗±同步化疗
   参考答案：E
   【解析】患者诊断为早期喉癌（cT2N0M0），手术或放射治疗均可达到根治效果，其总的5年生存率类似。放射治疗较手术能有效的保留患者的发音及吞咽功能的完整性。目前已进行的相关研究（如RTOG91～11等）的结果均显示同步放化疗在局部控制率和总体生存率上均较单纯放疗或序贯放化疗有一定的优势。化疗在喉癌中的地位主要是与手术或放疗联合，以及晚期姑息减症治疗。
2. 【单项选择题】经直接喉镜检查发现患者右侧声带原形消失、活动受限，局部可见大小为2cm×2cm肿块，累及前联合，左侧声带活动受限，声门上下区未见肿瘤侵犯，病检为（喉）鳞状细胞癌。辅助检查：颈部MRI检查提示肿瘤局限在右侧声带，颈部淋巴结未见肿大。胸片及腹部B超均未见异常。则考虑临床分期属于（ ）。
   A. cT4N0M0
   B. cT1N0M0
   C. cT3N0M0
   D. cT2N0M0
   E. 无法确定
   参考答案：D
   【解析】该临床分期主要是对T分期的判断。根据2002年UICC的TNM分期，该患者属喉癌声门型，病变局限在声门区，声带活动受限，故考虑为T2。
3. 【单项选择题】结合临床症状首先考虑可能的肿瘤诊断是（ ）。
   A. 下咽癌
   B. 食管癌
   C. 肺癌
   D. 喉癌
   E. 扁桃体癌
   参考答案：D
   【解析】患者症状主要为声嘶、喉部异物感及咽痛，均为喉癌相对特征性症状。下咽癌发病率较低，约占头颈部肿瘤的2%。晚期下咽癌因病变范围广泛也可出现上述症状。相对而言颈段食管癌较少出现喉部异物感和咽部疼痛。

【题干】患者男性，40岁，鼻塞及回吸性血涕3个月余，发现左颈肿块2周余，体检：左侧上强部一3cm×4cm大小肿块，质硬，边界清楚，活动度差。

1. 【单项选择题】该患者最有可能是下列诊断中的（ ）。
   A. 上颌窦癌
   B. 嗅神经母细胞瘤
   C. 鼻咽部淋巴瘤
   D. 鼻咽癌
   E. 鼻咽结核
   参考答案：D
   【解析】没有试题分析
2. 【单项选择题】为了明确诊断，该患者首先需要进行的检查是（ ）。
   A. B超
   B. CT
   C. MRI
   D. 电子鼻咽镜及活检
   E. EB病毒抗体检测
   参考答案：D
   【解析】鼻塞、回吸性血涕、颈部无痛性包块是鼻咽癌最常见的临床症状之一，一旦出现应高度警惕鼻咽癌可能，电子鼻咽镜及活检是明确诊断必不可少的检查手段，而MRI及CT等检查是在治疗前明确病变范围及分期的重要检查手段。

【题干】患者女性，26岁，孕37周，因"鼻塞2月，间断血涕3天"就诊。体检发现患者双上颈多个肿大淋巴结，最大径约3cm，行直接鼻咽镜检查发现右侧鼻咽咽隐窝消失，局部可见菜花样新生物，活检证实为鼻咽低分化鳞癌。

1. 【单项选择题】患者完善其他检查未发现远处转移，鼻咽及颈部MRI提示局部肿瘤侵犯翼腭窝，翼内肌受侵，上颈可见多个肿大淋巴结，目前考虑诊断分期属于（ ）。
   A. cT4N2M0
   B. cT1N1M0
   C. cT3N2MO
   D. cT2N1M0
   E. cT3N1M0
   参考答案：E
   【解析】没有试题分析
2. 【单项选择题】该药物在细胞的作用部位是（ ）。
   A. RAF基因表达产物
   B. VEGR受体
   C. EGFR受体胞外端
   D. EGFR胞内端酪氨酸激酶位点
   E. VEGFR胞内端酪氨酸激酶位点
   参考答案：C
   【解析】该题主要考查目前头颈部放化疗进展。爱必妥（C－225）已被FDA批准用于头颈部放疗的增敏治疗。其作用位点为EGFR受体胞外端。
3. 【单项选择题】患者诉近几天颈部肿块增大明显，鼻塞症状加重，关于下一步治疗，下面说法合理的是（ ）。
   A. 如局部进展迅速，可考虑先行手术切除术，避免放化疗对胎儿的影响，根据手术情况行放疗
   B. 局部放疗对胎儿没有影响，可以直接按常规行根治性放疗
   C. 由于妊娠期鼻咽癌进展迅速，可征求妇产科意见终止妊娠，尽快开始放化疗
   D. 建议等患者分娩后再进行抗肿瘤治疗
   E. 妊娠鼻咽癌预后较好，妊娠终止后往往能自行消退，建议密切观察
   参考答案：C
   【解析】妊娠合并鼻咽癌较少见，预后差。其病情往往短期内进展迅速，治疗原则上尽早终止妊娠开始放化疗。
4. 【单项选择题】如患者选择分子靶向药物进行放疗同步增敏，下面哪种药物已被美国FDA批准用于头颈部增敏治疗？（ ）
   A. 贝伐单抗
   B. 易瑞沙
   C. 索拉菲尼
   D. 特罗凯
   E. 爱必妥
   参考答案：E
   【解析】没有试题分析

共享答案题

【选项】
A. 嗅神经母细胞瘤
B. 鳞状细胞癌
C. 恶性黑色素瘤
D. 腺癌
E. 淋巴瘤

1. 【单项选择题】鼻腔及副鼻窦恶性肿瘤的病理类型中哪种常伴有周围卫星灶和颈部淋巴结转移？（ ）
   参考答案：C
   【解析】恶性黑色素瘤常伴有周围卫星灶和颈部淋巴结转移，远处转移较为多见。
2. 【单项选择题】鼻腔及副鼻窦恶性肿瘤的病理类型中哪种具有两个发病高峰年龄？（ ）
   参考答案：A
   【解析】嗅神经母细胞瘤源于神经脊干细胞的嗅觉细胞，有20岁和50岁左右两个发病高峰。
3. 【单项选择题】鼻腔及副鼻窦恶性肿瘤最常见的病理类型为（ ）。
   参考答案：B
   【解析】鼻腔及副鼻窦恶性肿瘤常见的病理类型包括鳞状细胞癌、腺癌、恶性黑色素瘤和嗅神经母细胞瘤。其中鳞状细胞癌占绝大多数。

【选项】
A. 鼻塞
B. 面部肿胀
C. 颅面疼痛
D. 鼻出血
E. 眼球移位

1. 【单项选择题】上颌窦癌最早出现的症状是（ ）。
   参考答案：B
   【解析】上颔窦癌早期较少出现症状，面部肿胀常是患者最早出现的症状，其次为颅面疼痛、鼻塞，以上三个症状是上颌窦癌最常出现的症状。
2. 【单项选择题】鼻腔癌最常见的症状是（ ）。
   参考答案：A
   【解析】鼻腔癌较早期症状包括反复出现血性分泌物、鼻腔肿块和疼痛。鼻塞为最常见的症状。在肿瘤体积较大时出现，由于肿瘤压迫可继发鼻泪管阻塞而致流泪、鼻外形改变及眼球移位。

【选项】
A. 活检
B. 骨ECT
C. 磁共振成像
D. CT扫描
E. 免疫组化

1. 【单项选择题】鼻腔及副鼻窦恶性肿瘤的最准确的诊断手段是（ ）。
   参考答案：A
   【解析】活检可明确病理类型，诊断最准确，对治疗具有重要指导意义。
2. 【单项选择题】副鼻窦恶性肿瘤首选的诊断方法是（ ）。
   参考答案：D
   【解析】CT是副鼻窦病变的首选检查方法，可显示累及范围和鉴别炎症、良性肿瘤和恶性肿瘤。

【选项】
A. 活检
B. 磁共振成像
C. 骨ECT
D. CT扫描
E. 免疫组化

1. 【单项选择题】能准确显示副鼻窦肿瘤病变范围并可鉴别肿瘤和炎症的诊断方法是（ ）。
   参考答案：B
   【解析】磁共振成像能准确显示病变范围，还可以对一些病变作出鉴别，特别是肿瘤和炎症。
2. 【单项选择题】对确诊嗅神经母细胞瘤最有帮助的检查是（ ）。
   参考答案：E
   【解析】应用免疫组化方法可以帮助诊断嗅神经母细胞瘤。

【选项】
A. 免疫治疗
B. 放射治疗
C. 化学治疗
D. 外科手术
E. 中药治疗

1. 【单项选择题】颌骨纤维肉瘤治疗首选（ ）。
   参考答案：D
   【解析】根治性的外科切除一直是治疗颌骨骨肉瘤、软骨肉瘤、纤维肉瘤、中央性颌骨癌及恶性纤维组织细胞瘤等恶性肿瘤的最有效的方法。
2. 【单项选择题】颌骨尤文肉瘤治疗首选（ ）。
   参考答案：B
   【解析】放射治疗通常是颌骨尤文肉瘤的首选治疗。
3. 【单项选择题】颌骨浆细胞肉瘤治疗首选（ ）。
   参考答案：C
   【解析】化疗常作为颌骨浆细胞肉瘤的首选治疗。

【选项】
A. 淋巴瘤
B. 未分化癌
C. 黏液表皮样癌
D. 鳞癌
E. 腺样囊性癌

1. 【单项选择题】小涎腺癌最常见的是（ ）。
   参考答案：E
   【解析】小涎腺癌中以腺样囊性癌最常见。
2. 【单项选择题】颌下腺肿瘤最常见的是（ ）。
   参考答案：E
   【解析】领下腺肿瘤中腺样囊性癌最常见，黏液表皮样癌次之。

【选项】
A. 唇癌
B. 舌癌
C. 齿龈癌
D. 口底癌
E. 颊黏膜癌

1. 【单项选择题】上述口腔癌中发病率最低的是（ ）。
   参考答案：E
   【解析】常见口腔癌发病率由高到低依次为唇癌、舌癌、口底癌、齿龈癌及颊黏膜癌。
2. 【单项选择题】头颈部肿瘤中仅次于皮肤癌的最常见口腔肿瘤是（ ）。
   参考答案：A
   【解析】没有试题分析
3. 【单项选择题】在口腔癌中，最易出现颈部淋巴结转移的是（ ）。
   参考答案：B
   【解析】口腔癌总的淋巴结转移率约36%，并且与病变范围和病期有关，局部早期（T1～2）一般低于20%，局部晚期（T3～4）则高达50%。病变越远离中线其淋巴结转移率越低。由颈淋巴结转移率自高到低为舌、口底、下牙龈、颊黏膜、上牙龈、硬腭及唇。

【选项】
A. 单纯手术
B. 术前放射治疗＋根治性手术
C. 根治性放疗
D. 手术±术后放射治疗
E. 单纯化疗

1. 【单项选择题】早期口咽癌如为分化差的癌或未分化的癌，且需要尽可能保留器官功能，首选（ ）。
   参考答案：C
   【解析】早期口咽癌手术或放疗疗效相当，分化差的肿瘤对放疗相对敏感，如需尽可能保留器官功能，根治性放疗作为首选。
2. 【单项选择题】舌癌如为外生型较大肿瘤，无坏死溃疡、周围软组织浸润，首选（ ）。
   参考答案：B
   【解析】没有试题分析
3. 【单项选择题】早期舌根鳞癌经根治性放疗后半年出现局部复发，首选（ ）。
   参考答案：A
   【解析】手术可作为根治性放疗后局部复发口腔癌的解救治疗手段，仍可取得较好的局部控制率。

【选项】
A. 呼吸困难
B. 声嘶
C. 痰中带血
D. 咽部异物感
E. 颈部肿块

1. 【单项选择题】晚期声门下癌最常见的就诊症状是（ ）。
   参考答案：A
   【解析】声嘶以声门区病变为主，咽部不适（吞咽不适、咽部阻挡感、咽部异物感）多见声门上病变。声门下癌起病隐匿，发病时即晚期，多见呼吸困难。
2. 【单项选择题】声门上区癌的早期主要表现为（ ）。
   参考答案：D
   【解析】没有试题分析
3. 【单项选择题】声门癌较典型的临床表现是（ ）。
   参考答案：B
   【解析】没有试题分析

【选项】
A. 扁桃体癌
B. 声门上癌
C. 声门下癌
D. 声门癌
E. 下咽癌

1. 【单项选择题】具有沿黏膜或黏膜下扩散特点的肿瘤多见于（ ）。
   参考答案：E
   【解析】声带部位基本没有淋巴管，因此早期声带癌很少出现颈部淋巴结转移；扁桃体低分化鳞癌或未分化癌较多见，下咽癌和声门上癌相对分化程度也较差，声门癌和声门下癌分化程度较高；下咽癌不仅容易出现淋巴结转移，局部病变往往较广泛，易沿黏膜或黏膜下广泛浸润。
2. 【单项选择题】以低分化和未分化癌常见的头颈部肿瘤多见于（ ）。
   参考答案：A
   【解析】没有试题分析
3. 【单项选择题】较少出现颈淋巴结转移的是（ ）。
   参考答案：D
   【解析】没有试题分析

【选项】
A. 梨状窝区、韦氏环区、咽侧壁区、椎前区
B. 梨状窝区、环后区、咽后壁区
C. 前壁、顶壁、后壁、侧壁
D. 声门上区、声门区、声门下区
E. 舌会厌区、扁桃体、咽旁间隙、咽后间隙

1. 【单项选择题】临床上下咽按照解剖部位通常分为（ ）。
   参考答案：B
   【解析】没有试题分析
2. 【单项选择题】按照UICC（2002）分期标准，口咽的4个解剖分区为（ ）。
   参考答案：C
   【解析】本题包括口咽、下咽和喉的基本解剖分区，这些分区在一定程度上可帮助解释不同部位肿瘤生物学行为的差异性。
3. 【单项选择题】临床上喉癌按照解剖特点分为（ ）。
   参考答案：D
   【解析】没有试题分析

【选项】
A. 先眼外肌麻痹后视力障碍
B. 患侧眼球固定，微外突
C. 先视力障碍后眼外肌麻痹
D. 患眼上肌麻痹，眼球固定，后突眼性视力麻痹
E. 眼球突出

1. 【单项选择题】鼻咽癌出现眶尖综合征的临床表现是（ ）。
   参考答案：D
   【解析】没有试题分析
2. 【单项选择题】鼻咽癌出现眶上裂综合征的临床表现是（ ）。
   参考答案：B
   【解析】没有试题分析
3. 【单项选择题】鼻咽癌出现垂体蝶窦综合征的临床表现是（ ）。
   参考答案：C
   【解析】没有试题分析

**第2章 肺癌及胸膜间皮瘤（N=55）**

单项选择题

1. 下列方法不属于肺癌定性诊断方法的是（ ）。
A. 肺癌的内镜检查
B. 痰细胞学检查
C. 经皮细针肺穿刺细胞学检查
D. 胸腔积液癌细胞学检查
E. PET检查
参考答案：E
【解析】 肺癌的定性检查方法包括细胞学检查（痰细胞学、胸腔积液细胞学、经皮细针肺穿刺细胞学、淋巴结穿刺细胞学、皮下结节细胞学）和内镜检查（纤维支气管镜、纵隔镜、胸腔镜）。

2. 肺癌患者行X线检查时，必须同时行胸部正位片和胸部侧位片检查，加做胸部侧位片，可使肺癌的检出率增加（ ）。
A. 7%
B. 1%
C. 5%
D. 3%
E. 9%
参考答案：A
【解析】 与胸部正位片相比，侧位片可发现心后三角区、肺底的肿瘤，使肺癌的检出率增加。

3. 小细胞肺癌应当采取的综合治疗方式是（ ）。
A. 以分子靶向治疗为主
B. 以手术为主
C. 以放疗为主
D. 以化疗为主
E. 以化放疗为主
参考答案：E
【解析】 小细胞肺癌易出现血行转移，所以无论局限期和广泛期均应进行化学治疗。在化疗的同时，在病灶部位可行放疗。

4. 肺癌高危人群的吸烟指数（每天吸烟支数×吸烟年数）应大于（ ）。
A. 400
B. 100
C. 300
D. 200
E. 500
参考答案：A
【解析】 没有试题分析

5. 中央型肺癌发生部位是（ ）。
A. 细支气管开口以上
B. 主支气管开口以上
C. 段支气管开口以上
D. 叶支气管开口以上
E. 毛细支气管开口以上
参考答案：C
【解析】 中央型肺癌发生在段支气管开口以上的支气管，周围型肺癌发生在段支气管开口以下的支气管。

6. 肺癌最常见的症状是（ ）。
A. 胸痛
B. 咳嗽
C. 胸闷
D. 血痰
E. 气促
参考答案：B
【解析】 没有试题分析

7. 吉非替尼的有效率与下列因素不相关的是（ ）。
A. EGFR表达
B. 分期
C. 性别
D. 从不吸烟者
E. 腺癌
参考答案：B
【解析】 没有试题分析

8. 有明显症状的急性放射性肺炎的临床治疗不包括（ ）。
A. 抗生素
B. 吸氧
C. 肾上腺皮质激素
D. 支气管扩张剂
E. 氨磷汀
参考答案：E
【解析】 氨磷汀对肺具有放射防护作用，在放射治疗的同时合并使用具有保护肺组织的作用，但在已经发生了放射性肺炎的患者没有太大作用。

9. 小细胞肺癌局限期不包括（ ）。
A. 少量胸腔积液
B. 同侧纵隔淋巴结转移
C. 同侧膈肌侵犯
D. 同侧锁骨上淋巴结转移
E. 轻度上腔静脉压迫综合征
参考答案：C
【解析】 小细胞肺癌局限期定义为病变局限于一侧胸腔，可包含于单个可耐受的放射野内。目前国内常用的局限期定义为病变局限于一侧胸腔、纵隔、前斜角肌及锁骨上淋巴结，但不能有明显的上腔静脉压迫、声带麻痹和胸腔积液。因此，少量胸腔积液和轻度上腔静脉压迫综合征均属于小细胞肺癌局限期。

10. 小细胞肺癌最多见的病理类型是（ ）。
A. 中间细胞型
B. 大细胞型
C. 癌肉瘤
D. 燕麦细胞型
E. 混合细胞型
参考答案：A
【解析】 中间细胞型占小细胞肺癌的70%。

11. 针对非小细胞肺癌根治术后的放射治疗，下列表述正确的是（ ）。
A. 适用于T4N1M0的病例
B. 适用于术后有肿瘤残存的病例
C. 适用于T2N1M0的病例
D. 适用于T4N0M0的病例
E. 适用于T3N0M0的病例
参考答案：B
【解析】 非小细胞肺癌根治术后放射治疗的适应证是：①术后有肿瘤残存的病例；②N2以上淋巴结阳性的病例。因此，术后分期为T4N0M0的患者不需要接受术后放射治疗。

12. 肺癌患者放疗过程中出现急性放射性食管炎的时间是（ ）。
A. DT40～60Gy左右
B. DT10～30Gy左右
C. DT30～50Gy左右
D. DT20～40Gy左右
E. DT50～70Gy左右
参考答案：D
【解析】 放射性食管炎多数表现为吞咽疼痛和进食困难。发生时间多数为20～40Gy左右，主要原因为食管黏膜的充血、水肿、渗出及糜烂。轻度的放射性食管炎患者可以观察，重者则需要进行激素和抗生素治疗。

13. 目前在我国，肺癌的发病率和死亡率居城市恶性肿瘤的（ ）。
A. 第4位
B. 第1位
C. 第3位
D. 第2位
E. 第5位
参考答案：B
【解析】 没有试题分析

14. 关于非小细胞肺癌的治疗原则，不正确的表述是（ ）。
A. 一般情况好、无胸腔积液的ⅢA期患者行同步化放疗
B. ⅠA期以手术为主
C. ⅢA期患者先行手术，术后行放疗和化疗
D. ⅠB和Ⅱ期患者先行手术，术后可行放疗和（或）化疗
E. Ⅳ期患者应行以全身治疗为主的综合治疗
参考答案：C
【解析】 ⅢA期患者最好先行新辅助治疗，然后再手术，术后根据情况进行其他抗肿瘤治疗。

15. 恶性胸膜间皮瘤治疗中应用最多的方法是（ ）。
A. 分子靶向治疗
B. 手术
C. 放疗
D. 化疗
E. 姑息治疗
参考答案：D
【解析】 恶性胸膜间皮瘤治疗困难，其治疗方法包括手术治疗、放射治疗和化疗。目前胸膜间皮瘤的放射治疗疗效差，因此化疗更多地应用于胸膜间皮瘤的治疗中。

16. 恶性胸膜间皮瘤标准的根治术不包括的范围是（ ）。
A. 膈肌
B. 患侧胸膜
C. 心包
D. 全肺
E. 纵隔淋巴结
参考答案：E
【解析】 恶性胸膜间皮瘤标准的根治术常包括患侧胸膜、全肺、心包及膈肌切除。

17. 下列不属于恶性胸膜间皮瘤手术适应证的是（ ）。
A. 患者伴有难以忍受的胸部疼痛
B. 病变集中一侧胸腔，无远处转移
C. 患者相对年轻，能承受手术创伤，预期术后能接受辅助治疗和较好的生活质量
D. 弥漫性恶性胸膜间皮瘤
E. 反复难以控制的胸腔积液而其他治疗无效
参考答案：D
【解析】 恶性胸膜间皮瘤手术适应证是：①病变集中一侧胸腔，无远处转移；②患者相对年轻，能承受手术创伤；③患者伴有难以忍受的胸部疼痛，或反复难以控制的胸腔积液而其他治疗无效。

18. 下列哪项是恶性胸膜间皮瘤目前疗效最好的化疗方案？（ ）
A. 培美曲塞＋奥沙利铂
B. 雷替曲塞＋顺铂
C. 培美曲塞＋顺铂
D. 雷替曲塞＋奥沙利铂
E. 吉西他滨＋顺铂
参考答案：C
【解析】 多靶点抗叶酸药物培美曲塞和顺铂联合方案治疗恶性胸膜间皮瘤的缓解率达到45%，中位生存时间达到13.3月。

19. 目前恶性胸膜间皮瘤唯一可能获得根治的手段是（ ）。
A. 分子靶向治疗
B. 手术
C. 化疗
D. 放疗
E. 姑息治疗
参考答案：B
【解析】 没有试题分析

20. 患者男性，57岁，反复咳嗽咳痰月余，X线图像如下，最有可能是下列哪项诊断？（ ）
A. 外周型肺癌
B. 肺结核瘤
C. 中央型肺癌
D. 肺转移瘤
E. 胸腺瘤
参考答案：A
【解析】 右上肺可见单个6cm大小实质性肿块，位于肺的外侧部分，无肺不张，外周型肺癌。肺转移瘤一般多发，中央型肺癌有肺不张，肺结核瘤有卫星灶，胸腺瘤发生在纵隔。

21. 患者男性，64岁，肝癌术后一年余，现出现反复咳嗽咳痰，X线图像如下，最有可能的诊断是（ ）。
A. 肺结核
B. 中央型肺癌
C. 肺转移癌
D. 外周型肺癌
E. 肺炎
参考答案：C
【解析】 双肺可见多个大小不等的肿块，有肝癌病史，肺转移癌。中央型肺癌有肺不张，外周型肺癌一般单发。

22. 患者男性，54岁，出现反复咳嗽咳痰3月，CT线图像如下，最有可能的诊断是（ ）。
A. 肺结核
B. 中央型肺癌
C. 肺脓肿
D. 外周型肺癌
E. 肺炎
参考答案：D
【解析】 左下肺可见单个3cm大小实质性肿块，位于外周带，可见分叶，外周型肺癌。中央型肺癌有肺不张，肺脓肿病史不符合。

23. 患者，女性，39岁。因“双侧膝关节疼痛5月，干咳2月，咯血3天”入院。查体：浅表淋巴结未及肿大，杵状指。胸部CT示左肺门占位性病变，活检报告为低分化腺癌。X线见双股骨下段骨膜增生，此表现为（ ）。
A. 骨膜炎
B. 类癌综合征
C. 滑膜炎
D. 类风湿性关节炎
E. 肺性肥大性骨关节病
参考答案：E
【解析】 肺性肥大性骨关节病多见于肺腺癌患者，主要临床表现为大关节疼痛，杵状指、趾，X线见长骨骨膜增生。

24. 患者男性，67岁。因“体检发现左肺占位性病变”入院，行左上肺叶切除术，术后病理示：左上肺高分化鳞癌，淋巴结癌转移，残端可见癌组织。患者术后行NP方案化疗4周期，并在化疗结束后行放疗。患者在放疗已完成52Gy时突然出现咳嗽、胸闷。胸片示：左肺下叶间质密度增高，左侧少量胸腔积液。此时，应当考虑的诊断是（ ）。
A. 放射性肺炎
B. 大叶性肺炎
C. 细支气管肺泡炎
D. 小叶性肺炎
E. 胸膜炎
参考答案：A
【解析】 没有试题分析

共享题干题

【题干一】
患者女性，57岁，因“咳嗽、胸闷半月余，加重2天”入院。胸部CT示：右上肺叶外周带占位性病变，纵隔淋巴结肿大、增多，右侧中等量胸腔积液。胸水细胞学可见腺癌细胞。头颅MRI、腹部B超、骨ECT均未发现异常。

25. 若患者PS评分为1，那么首选的治疗方式是（ ）。
A. 分子靶向治疗
B. 手术
C. 放疗
D. 化疗
E. 最佳支持治疗
参考答案：D
【解析】 没有试题分析

26. 患者的诊断与分期属于（ ）。
A. 右肺腺癌cT4N2M0，ⅢA期
B. 右肺腺癌cT4N1M0，ⅢA期
C. 右肺腺癌cT3N2M0，ⅢB期
D. 右肺腺癌cT3N2M0，ⅢA期
E. 右肺腺癌cT4N2M0，ⅢB期
参考答案：E
【解析】 没有试题分析

27. 若患者PS评分为3，那么首选的治疗方式是（ ）。
A. 分子靶向治疗
B. 手术
C. 放疗
D. 化疗
E. 最佳支持治疗
参考答案：E
【解析】 根据NCCN非小细胞肺癌诊疗指南，胸水细胞学检查阳性的患者，若PS评分为0～2分，那么应当选择化疗；若PS评分为3～4分，那么应当选择最佳支持治疗。

【题干二】
患者男性，70岁，左声门高中分化鳞癌T2N0M0，行左声带切除术后15年再次出现声嘶，有咳嗽及咳痰情况。查体：喉部外形完整，双颈部未及肿大淋巴结。喉镜检查局部未见异常。既往有吸烟史30余年。

28. 下列检查对帮助诊断及后续治疗意义不大的是（ ）。
A. 颈部B超
B. 头颈部MRI
C. 痰细胞学检查
D. 喉镜
E. 胸部CT检查
参考答案：C
【解析】 没有试题分析

29. 经纤维支气管镜活检证实为小细胞肺癌，下一步治疗策略是（ ）。
A. 以化疗为主的综合治疗，包括手术及放疗
B. 行根治性手术
C. 单纯放疗
D. 分子靶向治疗
E. 单纯化疗
参考答案：A
【解析】 没有试题分析

30. 患者行胸部CT示右上肺可见一大小约4cm×2cm占位，周围有毛刺，纵隔淋巴结肿大明显。头颈部MR检查未见异常。查血NSE明显升高。考虑最有可能的诊断是（ ）。
A. 炎性假瘤
B. 喉癌术后复发肺转移
C. 肺结核
D. 第二原发肿瘤：肺癌可能
E. 原发灶不明肺转移性肿瘤
参考答案：D
【解析】 该患者15年前确诊为喉癌早期，目前出现声嘶及咳嗽、咳痰。影像学检查发现肺部单一占位并纵隔淋巴结肿大，而喉部无异常；另血NSE升高明显；既往有吸烟史。临床上应高度怀疑第二原发肿瘤（肺癌）可能。

【题干三】
患者男性，46岁。因“干咳6个月”入院。查体：浅表淋巴结未及肿大。胸部CT示右肺上叶占位性病变，纤维支气管镜在右上叶支气管开口处见新生物，活检报告为小细胞癌。

31. 患者初始化疗时不可选用的方案是（ ）。
A. VP-16＋IFO＋DDP
B. CBP＋VP-16
C. CTX＋ADM＋VCR
D. VP-16＋DDP
E. CTX＋ADM＋DDP
参考答案：A
【解析】 局限期和广泛期小细胞肺癌的初始治疗方案：CE（CBP＋VP-16），EP（VP-16＋DDP），CDA（CTX＋ADM＋VCR），CAP（CTX＋ADM＋DDP），TPT＋DDP。复发性小细胞肺癌的治疗方案：VIP（VP-16＋IFO＋DDP），CPT-11＋DDP。

32. 如果患者行头颅MRI腹部B超、骨ECT均未发现异常，那么患者应当接受的治疗是（ ）。
A. 手术＋化疗
B. 手术
C. 放疗
D. 化疗
E. 化疗＋放疗
参考答案：E
【解析】 目前，局限期SCLC的标准治疗方案是化疗加局部放射治疗的综合治疗。

33. 对帮助判断小细胞肺癌最有价值的肿瘤标记物是（ ）。
A. CA199
B. CEA
C. Cyfra211
D. NSE
E. CA125
参考答案：D
【解析】 肺癌标志物包括：CEA、NSE、Cy-fra211，其中NSE对小细胞肺癌具有提示意义。

34. 如果对患者进行预防性放疗，那么应当接受治疗的部位是（ ）。
A. 肝
B. 胸椎
C. 脑
D. 腰椎
E. 胰腺
参考答案：C
【解析】 脑是SCLC常见的转移部位，全脑预防照射（PCI）能够提高生存率及无病生存率。

【题干四】
患者男性，62岁。因为“体检发现左肺占位性病变2天”入院。既往有吸烟史35年。入院后行胸部CT示：左上肺叶直径约2cm的占位性病变，纵隔内未见肿大淋巴结。纤维支气管镜可见左上叶支气管新生物，活检示：高分化鳞状细胞癌。患者行头颅MRI、腹部B超、骨ECT均未发现异常。

35. 患者的诊断是（ ）。
A. 左上肺高分化鳞癌pT2N0M0
B. 左上肺高分化鳞癌cT1N0M0
C. 左上肺高分化鳞癌cT2N0M0
D. 左上肺高分化鳞癌pT1N0M0
E. 左上肺高分化鳞癌cT3N0M0
参考答案：B
【解析】 没有试题分析

36. 若患者在手术或放射治疗后3年，出现双肺占位性病变，患者拒行化疗，那么患者可以选择的最佳分子靶向治疗药物是（ ）。
A. 厄罗替尼
B. 索拉非尼
C. 吉非替尼
D. 甲磺酸伊马替尼
E. 拉帕替尼
参考答案：A
【解析】 吉非替尼和厄罗替尼均用于非小细胞肺癌的分子靶向治疗。本患者为男性、有吸烟史、病理类型为鳞癌，选用厄罗替尼较吉菲替尼更为合适。

37. 若患者因心脏原因不能手术，那么在现有设备条件下，患者可选择的最佳的放射治疗为（ ）。
A. 四维放射治疗
B. 常规放疗
C. 三维适形放疗
D. 立体定向放疗
E. 实时跟踪放疗
参考答案：C
【解析】 目前在普通医院，常用的放射治疗技术包括：常规照射、立体定向放射治疗和三维适形放射治疗技术。三者之中，三维适形放射治疗技术的疗效最佳，副作用较小。

38. 若患者经完善相关检查，无手术禁忌证，那么首选的术式是（ ）。
A. 肺叶切除术＋肺门纵隔淋巴结清扫
B. 肺局部切除术
C. 全肺切除术
D. 肺叶切除术
E. 扩大性肺切除术＋肺门纵隔淋巴结清扫
参考答案：A
【解析】 肺癌的手术术式以肺叶切除加肺门纵隔淋巴结清扫为首选术式。

【题干五】
患者男性，63岁。因“胸背部疼痛3月余，加重1天”入院。胸部CT示：右上肺占位性病变，纵隔淋巴结肿大、增多，T9、T10椎体骨质破坏。活检示：低分化腺癌。骨ECT示：T9、T10、L1、右侧第5肋骨转移性病变。

39. 患者在3个月后突发一侧肢体乏力状况，需要考虑进行的检查是（ ）。
A. 血生化检查
B. 头颅MRI，以了解有无脑转移
C. 腹部CT
D. 脊髓MRI
E. 暂时不需要检查，观察病情变化
参考答案：B
【解析】 肺癌患者在出现一侧肢体乏力症状时，需要高度警惕有无脑转移的发生。

40. 若患者目前身体活动正常，那么应当接受的治疗除外（ ）。
A. T9、T10椎体放疗
B. 镇痛治疗
C. 化疗
D. 手术
E. 双磷酸盐治疗
参考答案：D
【解析】 患者系右肺低分化腺癌Ⅳ期（骨转移），在没有出现病理性骨折、明显脊髓压迫症状的时候，应当接受镇痛治疗、化疗、椎体放疗和双磷酸盐治疗。

41. 若对T9、T10椎体进行放疗，那么镇痛作用维持时间最长的照射剂量与剂量分别是（ ）。
A. DT8Gy/1F
B. DT40Gy/20F
C. DT20Gy/5F
D. DT30Gy/10F
E. DT6Gy/1F
参考答案：D
【解析】 在骨转移瘤的放射治疗中，对于估计有较长生存期，一般状况好者，给予DT30Gy/10F或DT40Gy/20F，副作用较小，缓解疼痛效果较好。

42. 在第3问的基础上，如果出现脑转移，应该马上进行的后续治疗是（ ）。
A. 口服替莫唑胺化疗
B. 分子靶向治疗
C. 姑息性放疗辅助脱水治疗
D. 全身化疗
E. 最佳支持对症治疗
参考答案：C
【解析】 在出现脑转移时，应该对患者进行全脑放疗，并辅助以脱水治疗。

共享答案题

【选项】
A. 腺鳞癌
B. 鳞状细胞癌
C. 大细胞癌
D. 腺癌
E. 小细胞癌

43. 恶性程度高，容易发生转移的肺癌组织学类型是（ ）。
参考答案：E
【解析】 没有试题分析

44. 以中央型肺癌为主的组织学类型是（ ）。
参考答案：B
【解析】 没有试题分析

45. 肺癌最常见的组织学类型是（ ）。
参考答案：D
【解析】 没有试题分析

【选项】
A. 胸腔镜
B. 纤维支气管镜
C. 纵隔镜
D. 经皮肺穿刺活检
E. 剖胸探查

46. 是肺癌分期的重要手段，同时也可用于胸部疑难疾病的鉴别诊断的是（ ）。
参考答案：C
【解析】 纵隔镜检查在确定肺癌有无纵隔淋巴结转移上有重要作用，是肺癌分期的重要手段。

47. 对于非创伤检查执行之后仍然未能确诊的恶性胸腔积液患者，可以选用的定性诊断方法是（ ）。
参考答案：A
【解析】 胸腔镜检查的适应证主要是：胸膜病变；恶性胸腔积液；肺的弥漫性病变等。

48. 患者身体健康，无手术禁忌证，胸部CT提示右肺下叶外带孤立的结节性病变，此时应当选用的定性诊断方法是（ ）。
参考答案：E
【解析】 对于肺部孤立的结节性病变，如果没有手术禁忌证，应选择剖胸探查，诊断与治疗同步进行。

【选项】
A. Pancoast综合征
B. 类癌综合征
C. Horner综合征
D. 上腔静脉阻塞综合征
E. Cushing综合征

49. 肺癌组织中的嗜银细胞产生的生物活性胺类，可以导致（ ）。
参考答案：B
【解析】 没有试题分析

50. 肺癌或转移淋巴结累及第7颈椎至第1胸椎外侧旁的交感神经，可以导致（ ）。
参考答案：C
【解析】 没有试题分析

51. 肺癌或转移淋巴结破坏第1、2肋骨和臂丛神经，可以导致（ ）。
参考答案：A
【解析】 没有试题分析

【选项】
A. 50～60Gy
B. 40～50Gy
C. 20～30Gy
D. 30～40Gy
E. 60～70Gy

52. 局部晚期NSCLC的姑息性照射剂量为（ ）。
参考答案：A
【解析】 没有试题分析

53. 不能耐受手术或拒绝手术的早期NSCLC根治性放疗的照射剂量为（ ）。
参考答案：E
【解析】 没有试题分析

【选项】
A. 50～60Gy
B. 20～30Gy
C. 40～50Gy
D. 30～40Gy
E. 60～70Gy

54. 晚期NSCLC脑转移的照射剂量为（ ）。
参考答案：D
【解析】 没有试题分析

55. SCLC局部放射治疗的照射剂量为（ ）。
参考答案：A
【解析】 没有试题分析

**第3章 纵隔肿瘤（N=11）**

单项选择题

1. 原发纵隔淋巴瘤多位于（ ）。
A. 下后纵隔区
B. 前上纵隔
C. 下前纵隔区
D. 后上纵隔
E. 中纵隔区
参考答案：E
【解析】 纵隔不同部位好发不同的纵隔肿瘤：前上纵隔是胸内甲状腺肿的好发部位；前上纵隔下部多见胸腺肿瘤及囊肿；前下纵隔上部是畸胎类肿瘤与囊肿的好发部位；后纵隔以神经源性肿瘤最常见；中纵隔的肿瘤绝大多数为恶性肿瘤如淋巴瘤或纵隔淋巴结转移癌。

2. 下列哪项是上纵隔区和下纵隔区的分界线？（ ）
A. 胸骨角与第四胸椎间盘连线
B. 胸骨柄与第三胸椎间盘连线
C. 胸骨角与第三胸椎间盘连线
D. 胸骨柄与第四胸椎间盘连线
E. 胸骨角与第五胸椎间盘连线
参考答案：A
【解析】 没有试题分析

3. 下列哪项是胸腺瘤致死的主要原因？（ ）
A. 脑转移
B. 局部侵犯纵隔重要脏器
C. 肝转移
D. 重症肌无力
E. 骨转移
参考答案：B
【解析】 胸腺瘤的扩散方式以胸内进展为主，侵及胸膜及心包时出现胸腔积液、心包积液，并直接侵犯周围组织及器官，严重者可出现呼吸困难、上腔静脉阻塞综合征等。淋巴结转移和血行转移发生率较低。

4. 下列有关胸腺瘤伴重症肌无力的说法错误的是（ ）。
A. 男性多于女性
B. 有的未经治疗可以自愈
C. 发生率为4.3%～54%
D. 有的治愈后又可复发
E. 40岁以上成人重症肌无力患者常伴胸腺瘤
参考答案：A
【解析】 胸腺瘤伴重症肌无力的发生率为4.3%～54%，部分病例可自愈或复发。重症肌无力常见于青春期与年轻成人，女性多于男性，40岁以上成人常伴胸腺瘤。

5. 下列有关纵隔畸胎瘤的表述错误的是（ ）。
A. 治疗以外科手术为主
B. 为良性肿瘤
C. X线典型表现为前下纵隔向一侧生长的圆形或椭圆形阴影，有时呈分叶状；多数边缘清晰，常可见囊壁钙化或不规则骨骼影
D. 常见的症状有胸闷、胸痛、咳嗽、气促及心悸等
E. 易发生继发感染
参考答案：B
【解析】 大多数畸胎类肿瘤位于前纵隔近心包底部，分为表皮样囊肿、皮样囊肿和畸胎瘤。约10%的纵隔畸胎瘤为恶性。

6. 良性神经源性肿瘤属于（ ）。
A. 恶性神经鞘瘤
B. 神经节细胞瘤
C. 节神经母细胞瘤
D. 神经母细胞瘤
E. 神经纤维肉瘤
参考答案：B
【解析】 没有试题分析

7. 有关纵隔神经源性肿瘤的外科治疗的陈述不正确的是（ ）。
A. 肿瘤位于胸前者应避免损伤胸3～4、交感神经而导致颈交感神经麻痹综合征
B. 一经诊断，原则上应早期手术切除
C. 多数肿瘤与肋间神经或交感神经有联系
D. 大都有完整的包膜，易于完整摘除
E. 来源于迷走神经者要注意勿损伤喉返神经
参考答案：A
【解析】 纵隔神经源性肿瘤一经诊断应早期手术切除。肿瘤多与肋间神经或交感神经有联系，有时呈哑铃状伸入椎间孔。手术应注意避免损伤脊髓、胸1～2交感神经（导致颈交感神经麻痹综合征）及喉返神经。

8. 患者，男，32岁。因“头面部肿胀半月”入院。胸部CT示：前上纵隔下部占位性病变。最有可能的诊断是（ ）。
A. 神经源性肿瘤
B. 胸内甲状腺肿
C. 畸胎类肿瘤
D. 胸腺肿瘤
E. 恶性淋巴瘤
参考答案：D
【解析】 前上纵隔下部多见的病变是胸腺肿瘤及囊肿。

9. 患者，女性，26岁。因“顽固性呃逆2周”入院，查体无阳性体征。胸部CT示：前下纵隔占位性病变，部分膈肌侵犯。此占位性病变可能侵犯的神经是（ ）。
A. 喉返神经
B. 胸交感神经节
C. 臂丛
D. 肋间神经
E. 膈神经
参考答案：E
【解析】 纵隔肿瘤可出现神经刺激症状，如膈神经受侵犯可出现顽固性呃逆。

10. 患者，女性，19岁。因“干咳5月，呼吸困难1天”入院。患者入院后咳出毛发样物。在诊断时应当考虑的肿瘤是（ ）。
A. 支气管囊肿
B. 胸腺肿瘤
C. 神经源性肿瘤
D. 畸胎瘤
E. 胃肠囊肿
参考答案：D
【解析】 没有试题分析

11. 患者，女性，47岁。因“进行性吞咽困难3月余”入院。患者伴有刺激性咳嗽，在仰卧位时加重。胸部CT示：上纵隔内可见轮廓清晰的占位性病变，呈分叶状，向双侧突出，食管部分受压。131I扫描可见上纵隔内热结节。此患者可能的诊断是（ ）。
A. 胸腺肿瘤
B. 胸内甲状腺肿
C. 胸腺囊肿
D. 胸内甲状腺囊肿
E. 支气管囊肿
参考答案：B
【解析】 胸内甲状腺肿多为颈部甲状腺肿或腺瘤向胸骨后延伸，压迫食管或气管时可引起吞咽困难、咳嗽等症状，仰卧位时加重。131I扫描可表现为热结节，CT有助于明确肿瘤范围及性质。

**第4章 消化系统肿瘤（N=94）**

单项选择题

1. 下列不是早期食管癌的临床表现的是（ ）。
A. 进食时胸骨后不适或疼痛
B. 食管内异物感
C. 进行性吞咽困难
D. 食物停滞感
E. 进食时胸骨后烧灼感
参考答案：C
【解析】 早期食管癌的症状有食物通过缓慢并有滞留，食管内有异物感，或者进食时胸骨后疼痛或烧灼感等，而进行性的吞咽困难则是中晚期食管癌的症状。

2. 下列不是晚期食管癌的临床表现的是（ ）。
A. 持续性胸背痛
B. 声音嘶哑
C. 胸骨后烧灼感
D. 进食时呛咳
E. 进行性吞咽困难
参考答案：C
【解析】 晚期食管癌的症状有胸背部疼痛；进行性吞咽困难；压迫交感神经后可引起声音嘶哑、进食时呛咳等，而胸骨后烧灼感在食管癌早期即可出现。

3. 食管癌主要发生于下列哪种组织？（ ）
A. 食管黏膜下结缔组织
B. 食管黏膜上皮细胞
C. 食管黏膜下腺体
D. 食管旁淋巴组织
E. 食管肌层
参考答案：B
【解析】 食管癌主要是食管黏膜上皮细胞发生癌变引起。

4. 下列是食管癌的癌前病变的是（ ）。
A. Barrett食管
B. 食管裂孔疝
C. 食管乳头状瘤
D. 食管息肉
E. 食管憩室
参考答案：A
【解析】 目前认为，食管癌前病变有Barrett食管、食管上皮增生和食管黏膜炎症。

5. 恶性程度较高的胃癌可以超越常规所属淋巴结转移方式，而直接侵犯远处淋巴结，其中最常见为（ ）。
A. 锁骨上淋巴结
B. 脾门淋巴结
C. 腹腔动脉周围淋巴结
D. 胃小弯淋巴结
E. 胃左动脉淋巴结
参考答案：A
【解析】 胃癌可直接侵犯更远处的淋巴结，如锁骨上淋巴结。

6. 提高胃癌治愈的关键在于（ ）。
A. 早期化疗
B. 早期诊断
C. 积极放疗
D. 彻底手术
E. 综合治疗
参考答案：B
【解析】 提高胃癌治愈的关键在于提高早期诊断率。

7. 胃癌的癌前期状态除外（ ）。
A. 胃酸缺乏症
B. 胃息肉
C. 慢性萎缩性胃炎
D. 胃平滑肌瘤
E. 恶性贫血
参考答案：D
【解析】 胃癌的癌前期状态包括胃息肉、慢性萎缩性胃炎、胃酸缺乏症、恶性贫血等，而胃平滑肌瘤只是胃的良性肿瘤，一般不发生恶变。

8. 从病理学上分期，下列属于早期胃癌概念的是（ ）。
A. 无淋巴结转移
B. 局限于胃窦内
C. 直径在2cm以内
D. 局限于黏膜及黏膜下层
E. 尚未侵及浆膜层
参考答案：D
【解析】 早期胃癌是指局限在黏膜和黏膜下层的胃癌，而无论其是否伴有淋巴结转移。

9. 关于胃癌，下列叙述错误的是（ ）。
A. 胃大弯和前壁一般不发生胃癌
B. 胃癌占我国消化道肿瘤的第一位
C. 胃癌发生部多在胃窦，其次是胃小弯
D. 胃癌发病年龄以40～60岁多见
E. 贲门区的胃癌比胃大弯更常见
参考答案：A
【解析】 胃癌可发生于胃的各个部位，最多在胃窦，其次是胃小弯。

10. 下列属于结肠癌最重要诊断方法的是（ ）。
A. 腹部B超
B. 钡剂灌肠X线检查
C. 纤维结肠镜检查＋组织活检
D. CEA测定
E. 直肠指诊
参考答案：C
【解析】 纤维结肠镜检查＋组织活检是最直接也是最重要的一种诊断方法。

11. 关于结肠癌，下列不正确的是（ ）。
A. 结肠癌的血行转移多转移至肝
B. 结肠癌可伴有贫血和发热
C. 左半结肠癌以全身中毒症状为主
D. 根据肿瘤形态，可分为肿块型，浸润型，溃疡型
E. 结肠癌淋巴转移首先转移到结肠旁淋巴结
参考答案：C
【解析】 左半结肠癌以肠梗阻表现多见，而右半结肠癌多伴有贫血、消瘦、发热、无力等全身中毒症状。

12. 左半结肠癌急性梗阻时，下列措施中不恰当的是（ ）。
A. 胃肠减压后，待梗阻解除便作左半结肠切除术
B. 注意纠正水电解质及酸碱失衡
C. 稍准备好便手术，先作横结肠造口术，二期再作左半结肠切除吻合术
D. 注意腹部情况，预计病变严重程度，早插胃管，进行胃肠减压
E. 急诊作心电图、血生化等检查，应初步纠正酸中毒后手术
参考答案：A
【解析】 左半结肠癌多伴有急性肠梗阻，属于急诊手术适应证，应及早插胃管，进行胃肠减压，纠正水电解质及酸碱失衡后行手术治疗。

13. 下列因素与原发性肝癌发病最密切的是（ ）。
A. 饮用水污染
B. 肝硬化
C. 放射线辐射
D. 病毒性肝炎
E. 寄生虫
参考答案：D
【解析】 据统计，80%～90%左右的肝癌患者常有急性肝炎、慢性肝炎、肝硬化、肝癌的病史，提示病毒性肝炎与肝癌可能有因果关系。

14. 原发性肝癌经淋巴转移最常见于下列哪个部位？（ ）
A. 肝门淋巴结
B. 锁骨上淋巴结
C. 胰腺旁淋巴结
D. 主动脉旁淋巴结
E. 脾门淋巴结
参考答案：A
【解析】 原发性肝癌局部转移到肝门淋巴结最常见（占12.6%）。

15. 原发性肝癌肝外血行转移最常见于下列哪个部位？（ ）
A. 肾上腺
B. 脑
C. 肺
D. 骨髓
E. 肾
参考答案：C
【解析】 原发性肝癌血行转移：以肺转移率最高，占43.5%。

16. 原发性肝癌按大体形态分型下述错误的是（ ）。
A. 结节型易发生癌结节破裂出血
B. 巨块型癌直径＞10cm
C. 巨块型易发生坏死引起肝破裂
D. 孤立的直径＜5cm的癌结节称为小肝癌
E. 弥漫型往往因肝功能衰竭而死亡
参考答案：A
【解析】 巨块型肝癌是指肿块直径大于10cm，巨块型易发生破裂出血。

17. 原发性肝癌的临床特点错误的是（ ）。
A. 癌块侵犯肝门胆管引起黄疸
B. 肿块增长迅速，肝包膜被牵拉引起疼痛
C. 门脉外癌栓可以产生血管杂音
D. 门脉内癌栓可导致门脉高压
E. 肿瘤生长缓慢，可以无痛
参考答案：C
【解析】 由于肿瘤压迫肝内大血管或肿瘤本身血管丰富可以产生肝区血管杂音。

18. 原发性肝细胞癌的AFP阳性率（ ）。
A. 60%～70%
B. 100%
C. 70%～90%
D. 80%～90%
E. 50%～60%
参考答案：A
【解析】 没有试题分析

19. 下列属于原发性肝细胞癌最敏感肿瘤标志的是（ ）。
A. AP
B. 肝脏B超
C. AFP
D. 肝CT
E. r-GT
参考答案：C
【解析】 AFP是原发性肝细胞肝癌的最灵敏、最特异的肿瘤标志。

20. 除原发性肝癌，哪种疾病能引起AFP＞500μg/L？（ ）
A. 肾脏胚胎瘤
B. 多囊肝
C. 生殖腺胚胎瘤
D. 慢性肝炎
E. 肝硬化
参考答案：C
【解析】 生殖腺胚胎性肿瘤可引起AFP明显升高＞500μg/L。

21. AFP＞200μg/L排除活动性肝病诊断肝癌的标准为（ ）。
A. ALT正常，AFP＞200μg/L持续8周
B. ALT同步升降持续一个月
C. ALT正常，AFP＞200μg/L持续一个月
D. ALT同步升降持续二个月
E. ALT正常，AFP＞200μg/L持续8个月
参考答案：A
【解析】 在排除活动性肝病、妊娠和生殖腺胚胎瘤的基础上，AFP检查诊断肝细胞癌的标准为：①AFP大于500μg/L持续4周；②AFP由低浓度逐渐升高不降；③AFP在200μg/L以上的中等水平持续8周。

22. 下列有助于鉴别肝癌和良性活动性肝病的是（ ）。
A. 肝功能明显损害
B. HBsAg阳性
C. AFP阴性
D. AFP阳性
E. AFP和ALT动态曲线
参考答案：E
【解析】 肝癌和活动性肝病AFP都可为阳性。肝炎患者血清AFP升高通常为“一过性”，且往往伴有转氨酶显著升高，而肝癌患者血清AFP持续上升，往往超过500ng/ml，此时与转氨酶下降呈曲线分离现象。

23. 肝癌的一线诊断方法是（ ）。
A. 肝动脉造影
B. B超
C. MRI
D. CT
E. B超＋AFP定量测定
参考答案：E
【解析】 B超检查＋AFP定量测定作为肝癌的一线诊断方法。

24. 原发性肝癌首选和最有效的方法是（ ）。
A. 中医治疗
B. 化学抗癌药物治疗
C. 放射治疗
D. 手术切除治疗
E. 生物和免疫治疗
参考答案：D
【解析】 对于可以切除的肝癌，首选手术治疗。

25. 不可行根治性肝切除的情况是（ ）。
A. 肝功能异常属Child C级
B. 单发的微小肝癌
C. 单发的向肝外生长的大肝癌，表面光滑，边界清楚，正常肝组织超过70%
D. 单发的小肝癌
E. 小于3个的多发结节，局限在一个肝叶内
参考答案：A
【解析】 肝功能异常达到Child C级是肝癌手术的禁忌证。

26. 胰腺癌最好发的部位是（ ）。
A. 全胰腺
B. 胰腺头部
C. 胰腺尾部
D. 胰腺体部
E. 异位胰腺
参考答案：B
【解析】 胰腺癌以胰腺头部最常见，约占胰腺癌的60%～70%。

27. 胰腺癌最常见的首发症状是（ ）。
A. 消瘦和乏力
B. 上腹痛和上腹饱胀不适
C. 消化道症状
D. 黄疸
E. 发热
参考答案：B
【解析】 上腹部不适及隐痛是胰腺癌最常见的首发症状。

28. 胰腺癌首选的无创性检查方法是（ ）。
A. MRI
B. 超声检查
C. CT
D. X线钡餐造影
E. 肿瘤标记物CA199
参考答案：B
【解析】 超声检查是胰腺癌的首选无创性检查。B超对胰腺癌的诊断阳性率可达90%。

29. 胰腺癌同步放化疗的放疗剂量推荐为DT（ ）。
A. 66Gy/33F/7W
B. 60Gy/30F/6W
C. 40Gy/20F/4W
D. 50Gy/25F/5W
E. 44Gy/22F/5W
参考答案：D
【解析】 没有试题分析

30. NCCN指南推荐晚期胰腺癌的一线标准治疗药物是（ ）。
A. 多西紫杉醇
B. 吉西他滨
C. 紫杉醇
D. 长春瑞滨
E. 培美曲塞
参考答案：B
【解析】 没有试题分析

31. 患者男性，58岁，吞咽困难2月余，X线图像如下，最有可能的诊断是（ ）。
A. 慢性食管炎
B. 食管癌
C. 食管间质瘤
D. 食管静脉曲张
E. 食管结核
参考答案：B
【解析】 男性，可见食管中段狭窄，可见充盈缺损，黏膜破坏，局部蠕动消失，食管癌。

32. 患者男性，69岁，进行性腹痛腹胀月余，X线图像如下，最有可能的诊断是（ ）。
A. 胃间质瘤
B. 慢性胃炎
C. 十二指肠癌
D. 胃溃疡
E. 胃癌
参考答案：E
【解析】 老年男性，可见胃窦狭窄，可见胃壁侵犯，黏膜破坏，局部蠕动消失，典型胃癌。

33. 患者女性，65岁，反复腹痛，腹泻3月余，X线图像如下，最有可能的诊断是（ ）。
A. 肠梗阻
B. 肠结核
C. 结肠息肉
D. 结肠癌
E. 节段性肠炎
参考答案：D
【解析】 可见横结肠中段局部狭窄，可见黏膜破坏，局部蠕动消失，典型结肠癌。

34. 患者男性，49岁，反复腹痛，进行性黄疸月余，CT图像如下，最有可能的诊断是（ ）。
A. 肾癌
B. 胆管癌
C. 胰头癌
D. 肝癌
E. 淋巴瘤
参考答案：C
【解析】 可见胰头4cm大小肿块，胰管和胆管扩张，进行性黄疸，典型胰头癌。

35. 患者男性，49岁，发现乙肝十余年，反复腹痛，腹胀黄疸月余，CT图像如下，最有可能的诊断是（ ）。
A. 肝肉瘤
B. 肝血管瘤
C. 肝癌
D. 肝再生结节
E. 肝腺瘤
参考答案：C
【解析】 可见肝脏超过20cm巨大肿块，平扫低密度，增强不均匀强化，乙肝十余年，反复腹痛，腹胀黄疸，典型肝癌。

36. 患者男性，54岁，体检发现肝脏占位，MR图像如下，最有可能的诊断是（ ）。
A. 肝癌
B. 肝肉瘤
C. 肝血管瘤
D. 肝腺瘤
E. 肝再生结节
参考答案：A
【解析】 肝脏右叶7cm肿块，可见子灶，T1WI低信号，T2WI等信号，动脉期增强早期强化，静脉期不均匀强化，延时低信号，典型肝癌。

37. 男，62岁，吞咽梗噎感2个月，人渐消瘦，临床高度怀疑食管癌。确诊的方法是（ ）。
A. 锁骨上淋巴结活检
B. CT
C. 胸部MRI检查
D. 食管吞钡X线检查
E. 食管镜检查及组织活检
参考答案：E
【解析】 食管镜检查及组织活检是明确病理诊断的最佳方法。

38. 男，63岁，进行性吞咽困难3个月，近1周来出现声音嘶哑。下列检查对患者适合的是（ ）。
A. 食道吞钡＋食管镜＋间接喉镜
B. 胸部X线片＋心电图＋胸部CT
C. 食管镜＋胸部MRI＋纤维支气管镜
D. 腹部B超＋痰细胞学检查＋间接喉镜
E. 癌胚抗原＋同位素32P扫描＋胃液分析
参考答案：A
【解析】 进行性吞咽困难是食管癌的比较典型的症状，患者合并有声嘶症状，初步分析可能是纵隔淋巴结肿大压迫喉返神经所致，可行间接喉镜了解声带运动情况。

39. 男，60岁，吞咽困难3个月，食管镜检报告中段食管鳞癌，病变长度约3.5cm，外侵不明显，无远处转移，下述处理最合适的是（ ）。
A. 食管大部分切除，胃代食管术
B. 食管大部分切除，空肠代食管术
C. 食管大部分切除，结肠代食管术
D. 食管病灶切除，食管端端吻合术
E. 放疗＋抗癌药物治疗
参考答案：A
【解析】 因该患者为中段食管癌，且病变相对局限，故手术方式为食管大部分切除＋胃代食管术。

40. 男，50岁，嗜酒吸烟，近3周来出现吞咽时胸骨后烧灼痛，门诊吞钡照片未发现明显异常。为进一步明确诊断，应作的检查是（ ）。
A. 大便潜血试验
B. 食管镜检查或食管拉网细胞检查
C. 胸部CT
D. 胸部X线摄片
E. 继续观察
参考答案：B
【解析】 该患者出现吞咽时胸骨后烧灼痛，有可能是早期食管癌的症状，由于吞钡检查未发现明显异常，故可行食管镜检查或食管拉网细胞检查以进一步明确诊断。

41. 男性，42岁，上腹烧灼样痛半年，近一周排柏油便，为确诊，宜首选的检查方法是（ ）。
A. B型超声检查
B. X线胃肠钡餐透视
C. 大便潜血检查
D. X线钡灌肠透视检查
E. 胃镜检查
参考答案：E
【解析】 该患者上腹部烧灼样痛，并有上消化道出血情况，应高度怀疑胃的疾病，因此胃镜是作为明确诊断的首选。

42. 男，48岁，因胃癌而行剖腹探查时，发现肿块已浸润至横结肠，但较局限，该患者宜（ ）。
A. 行全胃切除术
B. 禁忌手术
C. 行胃大部切除术
D. 行胃癌根治术＋横结肠切除术
E. 行横结肠切除术
参考答案：D
【解析】 该患者剖腹探查发现胃癌已浸润至横结肠，如果范围较局限，仍然可以选择行根治术。

43. 女，40岁，上腹隐痛不适，近2个月来加剧，服胃痛片后有所缓解，食欲尚可，大便隐血试验(++)，胃肠道钡餐检查见胃窦部小弯侧黏膜纹理紊乱，胃壁僵直不规则。首先应考虑的诊断是（ ）。
A. 胃黏膜脱垂
B. 慢性胃窦炎
C. 胃癌
D. 胃溃疡
E. 萎缩性胃炎
参考答案：C
【解析】 临床上如遇到年龄40岁以上，伴有上腹痛、消瘦、便血、食欲减退等症状就要考虑到胃癌的可能性，需进一步行胃镜或者X线检查。胃癌典型的X线表现为：胃腔狭窄、胃壁僵硬、蠕动消失、黏膜异常等。

44. 男，28岁，近半年来腹泻与便秘交替发生，近3个月来腹部隐痛，近2天解鲜血便，腹部触诊和直肠指诊未发现肿块，钡灌肠示降结肠肠壁僵硬，可见充盈缺损。应诊断为（ ）。
A. 溃疡性结肠炎（增殖型）
B. 乙状结肠癌
C. 肠结核
D. 直肠壶腹癌
E. 降结肠癌
参考答案：E
【解析】 根据患者病史、症状及体征，符合肠道肿瘤的判断，再根据钡剂灌肠的结果，确定病变部位在降结肠。

45. 男，72岁，因乏力，消瘦近一年而来院就诊，查体：贫血貌、消瘦，右下腹可扪及一4cm×3cm大小的肿块，界清，质硬，无明显压痛，纤维结肠镜检查提示为盲肠癌，对该患者行根治性右半结肠切除术应包括下述范围，除外（ ）。
A. 左半横结肠
B. 右半横结肠
C. 盲肠
D. 升结肠
E. 长约15～20cm的回肠末段
参考答案：A
【解析】 盲肠癌的手术范围按右半结肠癌根治术的要求进行，应包括盲肠、升结肠、右半横结肠及部分末段回肠，而不需要切除左半横结肠。

46. 男，60岁，因下腹部隐痛月余就诊，低热，纳差，腹泻，有过结核病史。体查：贫血貌，右下腹可触及包块，压痛明显。实验室检查：WBC10×10⁹/L，N0.75，ESR25mm/h。腹部B超示右下腹肿块并显示出液性平段，钡灌肠示盲肠充盈缺损，黏膜破坏，阑尾未充填。应诊断是（ ）。
A. 盲肠憩室炎
B. 阑尾周围脓肿
C. 回盲部肿瘤
D. 回盲部结核
E. 克隆病
参考答案：C
【解析】 根据该患者的症状、体征和既往史，不能准确区分结核、炎症或者肿瘤，但钡灌肠的表现强烈提示了肿瘤的诊断。

47. 男性，36岁，单位健康体检发现AFP升高＞500μg/L，肝功能正常，HBsAg（＋）HBeAg（＋）HBcAb（＋），最可能的诊断是（ ）。
A. 肝癌二期
B. 生殖腺胚胎瘤
C. 肝硬化晚期
D. 慢性活动性肝炎
E. 亚临床肝癌
参考答案：E
【解析】 中年男性，肝炎病史，AFP＞500μg/L，肝功能正常，患者无症状，考虑亚临床肝癌。

48. 男，40岁，有慢性肝炎史15年，近来腹胀消瘦，体格检查：巩膜黄染，上腹部触到拳头大质硬、表面不光滑包块，最可能的诊断是（ ）。
A. 肝脓肿
B. 原发性肝癌
C. 慢性肝炎
D. 转移性肝癌
E. 肝硬化
参考答案：B
【解析】 中年男性，肝炎病史，症状：腹胀、消瘦，体征：上腹部肿块，黄疸，考虑为原发性肝癌。

49. 男38岁，HBsAg（＋）20年，近期乏力，肝区不适做B超检查，发现肝右叶有一3cm×3cm实质性暗区有声晕，最可能的诊断是（ ）。
A. 肝囊肿
B. 肝硬化（结节性）
C. 转移性肝癌
D. 原发性肝癌
E. 肝血管瘤
参考答案：D
【解析】 中年男性，乙肝病史，症状：乏力、肝区不适，B超：肝内结节，考虑原发性肝癌。

50. 45岁，男，右肋痛3个月，微热，巩膜轻度黄染，肝于吸气时肋下1.0cm质中等，右膈外侧抬高，B超肝内大小不等的结节样回声，边缘不整齐，HBsAg（＋），甲胎蛋白为100μg/L，最有可能的诊断是（ ）。
A. 原发性肝癌
B. 乙型肝炎
C. 阿米巴肝脓肿
D. 肝硬化
E. 淤胆性肝炎
参考答案：A
【解析】 没有试题分析

51. 女性，50岁，黄疸，B超检查显示肝内胆管直径约1cm，应进一步选择的检查是（ ）。
A. 十二指肠低张造影
B. 静脉胆道造影
C. 经皮肝穿刺胆道造影
D. 核素扫描
E. 腹腔动脉造影
参考答案：C
【解析】 肝内胆管扩张应行经皮肝穿刺胆道造影明确病因。

52. 男性，65岁，进行性黄疸3个月，伴中上腹持续性胀感，夜间平卧时加重，消瘦显著。查体：慢性消耗性面容。皮肤、巩膜黄染。腹平坦，脐右上方深压痛，未及块物。Courvoisier征阳性。首先考虑的诊断是（ ）。
A. 胃癌
B. 慢性胆囊炎
C. 原发性肝癌
D. 胆石症
E. 胰头癌
参考答案：E
【解析】 老年男性，进行性黄疸，消耗体征，Courvoisier征阳性，应诊断为胰头癌。

53. 男性，35岁，黄疸1个月，右上腹轻微胀痛，食欲减退，经内科治疗无效。查体：肝大，胆囊增大，血胆红素17μmo1/L，AST70U/L，AKP45U/L，AFP＞5ng/ml，可能诊断是（ ）。
A. 肝硬化晚期
B. 黄疸性肝炎
C. 肝癌
D. 胆总管结石梗阻
E. 壶腹周围癌
参考答案：E
【解析】 中年男性，症状：黄疸、腹胀、食欲差，体征：肝、胆囊大，提示梗阻，且梗阻部位在胆总管以下，AFP不高，肝功能异常，考虑壶腹周围癌。

54. 男性，53岁。剧烈腹痛，黄疸。B超显示：肝内回声均匀，血管纹理清，肝内管壁回声增强，胰头处见50mm×32mm不规则低回声团块，边界不清，胰管4mm。考虑的诊断是（ ）。
A. 胆囊癌
B. 肝癌
C. 胰管扩张
D. 胰头癌
E. 胆管结石
参考答案：D
【解析】 症状：腹痛、黄疸，影像学B超提示胰头处不规则低回声团块，考虑为胰头癌。

55. 男性，40岁，持续黄疸9个月，伴皮肤瘙痒，查体：体温39℃，肝肋下5cm，中等硬度，表面稍不平，压痛(+)，甲胎蛋白（一），为明确诊断，下列检查最有价值的是（ ）。
A. 放射性核素扫描
B. 白细胞计数及分类
C. 胸部透视
D. 血γ-谷氨酰转肽酶测定
E. 腹部B超检查
参考答案：E
【解析】 B超检查在这几个选项中是最好的选择，可了解肝、胆、胰腺等的情况。

56. 女性，60岁，发现腹部肿物半月。查体：腹部软，触诊可探及肿物7cm×6cm×6cm，可活动，无压痛。腹部B超示：腹腔内实性结节，结节间有融合征象。免疫组织化学染色示肿瘤细胞：CD117＋，CD34＋，SMA-，Desmin－，S－100＋。考虑诊断（ ）。
A. 肉瘤
B. 肠道肿瘤
C. 平滑肌瘤
D. 间质瘤
E. 黑色素瘤
参考答案：D
【解析】 老年女性，腹腔实性肿块，CD117、CD34阳性，诊断为间质瘤。

共享题干题

【题干一】
患者男性，48岁，因吞咽梗噎感半年来院就诊，目前仅能进半流质食物。查体：稍消瘦，锁骨上未扪及肿大淋巴结。食管吞钡X线片示食管中下段4cm长之局限性管壁僵硬，黏膜部分中断，钡剂尚能通过。

57. 首先考虑的诊断是（ ）。
A. 贲门失弛缓症
B. 食管炎
C. 食管良性肿瘤
D. 食管癌
E. 食管憩室
参考答案：D
【解析】 没有试题分析

58. 确诊后应选择的治疗措施是（ ）。
A. 免疫疗法
B. 手术治疗
C. 化学疗法
D. 放射疗法
E. 中医中药治疗
参考答案：B
【解析】 该患者临床分期较早，未发现远处转移征象，故首选手术治疗。

59. 进一步的检查应是（ ）。
A. 胸腔镜检查
B. 胸部CT
C. 食管镜检查及组织活检
D. 胸部MRI
E. 纵隔镜检查
参考答案：C
【解析】 在不进行手术的情况下，食管镜检查及组织活检是下一步必须完成的检查。

【题干二】
患者男性，71岁，腹部胀痛3天，伴反酸、嗳气、食欲下降，查体：剑突下轻压痛，无反跳痛，肝脾肋下未及，移动性浊音阴性。胃镜检查示胃窦部巨大溃疡。

60. 如果HP检查为阴性，下一步治疗（ ）。
A. 化疗＋放疗
B. 手术
C. 放疗
D. 化疗
E. 抗HP治疗
参考答案：C
【解析】 黏膜相关组织淋巴瘤，对于早期患者，HP阴性患者首选放疗。

61. 如果HP检查为阳性，下一步治疗（ ）。
A. 化疗＋放疗
B. 手术
C. 放疗
D. 化疗
E. 抗HP治疗
参考答案：E
【解析】 黏膜相关组织淋巴瘤，HP阳性首选抗HP治疗。

62. 下列检查对诊断最有价值的是（ ）。
A. 腹部X片
B. CT
C. MRI
D. B超
E. 胃组织活检
参考答案：E
【解析】 没有试题分析

63. 如果胃镜活检结果为胃黏膜相关组织淋巴瘤，下一步应进行（ ）。
A. 胸部平片
B. HP检查
C. 腹部CT
D. 骨髓穿刺
E. 结肠镜检查
参考答案：B
【解析】 没有试题分析

【题干三】
患者男性，60岁，胃溃疡病史8年，近3个月腹胀、食欲减退、消瘦明显、粪隐血持续阳性，应用抗酸剂治疗胃痛无效。

64. 按照2008年版美国NCCN指南推荐，该患者具有同步放化疗指征，指南推荐的同步化疗药物是（ ）。
A. 5-Fu
B. 奥沙利铂
C. 紫杉醇
D. 顺铂
E. VP-16
参考答案：A
【解析】 没有试题分析

65. 首选的治疗方法是（ ）。
A. 迷走神经切断术
B. 胃大部切除术
C. 全胃切除术
D. 胃癌根治术
E. 给予黏膜保护剂等继续内科治疗
参考答案：D
【解析】 根据题目提供的信息，应高度怀疑为胃溃疡恶变，胃癌的治疗如无远处转移，应首选手术。

66. 该患者最有可能的诊断是（ ）。
A. 胃泌素瘤
B. 复合溃疡
C. 顽固性溃疡
D. 穿透性胃溃疡
E. 胃溃疡恶变
参考答案：E
【解析】 没有试题分析

67. 应首选的检查方法是（ ）。
A. 腹部X线平片
B. CT扫描
C. MRI检查
D. B超
E. 纤维胃镜活检
参考答案：E
【解析】 没有试题分析

68. 患者术后的病理报告是胃窦低分化腺癌，穿透浆膜层，未累及周围组织，伴8/17枚淋巴结转移，则根据2002年版的AJCC分期患者术后病理分期为（ ）。
A. T2N2M0
B. T2N1M0
C. T3N2M0
D. T3N4M0
E. T3N1M0
参考答案：C
【解析】 2002年版AJCC分期将肿瘤穿透浆膜层但未累及周围组织定义为T3，周围淋巴结7～15个转移定义为N2，因此该患者分期为T3N2M0。

69. 该患者进行术后放疗的推荐剂量是（ ）。
A. 60Gy
B. 45Gy
C. 30Gy
D. 55Gy
E. 56Gy
参考答案：B
【解析】 没有试题分析

【题干四】
患者男性，58岁，进行性贫血，消瘦、乏力半年，有时右腹隐痛，无腹泻。查体：右中腹部扪及肿块，肠鸣音活跃。

70. 如果需要手术治疗，下列术前准备最重要的是（ ）。
A. 肝肾功能检查
B. 纠正营养
C. 心肺功能检查
D. 肠道准备
E. 心理准备
参考答案：D
【解析】 由于下消化道寄生菌多，如肠道清洁不好，手术后易并发感染、腹膜炎甚至感染性休克。

71. 下列检查可明确诊断的是（ ）。
A. B超
B. 纤维结肠镜
C. CT
D. CEA
E. X线钡餐检查
参考答案：B
【解析】 纤维结肠镜及组织活检是明确诊断的最佳选择。

72. 如果B超发现肝脏多个转移灶，患者一般情况可，PS评分为1，肝功能正常，这时的治疗应首选（ ）。
A. 介入治疗
B. 放疗
C. 化疗
D. 手术
E. 中药
参考答案：C
【解析】 如此时发现的其他重要脏器出现转移，且为多个转移灶，往往提示已失去手术机会，应考虑全身治疗为主。

【题干五】
患者女性，65岁，皮肤、巩膜黄染30天，伴皮肤瘙痒、厌食、乏力、消瘦、小便黄、大便发白等；既往史无特殊。体检：皮肤、巩膜黄染，腹部无压痛反跳痛，肝区无叩击痛，可及肿大的胆囊，腹水征阴性。实验室检查：TBil：25mg/dl、DBil：20mg/dl、CA199：12u/ml、CA50：8u/ml；影像学检查：B超：低位胆道梗阻、肝内外胆管扩张，胰头不大；CT：低位胆道梗阻、肝内外胆管扩张、胰管扩张（双管征），胰腺未见明显占位；十二指肠低张造影：十二指肠曲不大、降部黏膜紊乱，未见明确的充盈缺损。

73. 患者行胸部CT提示双肺广泛转移，行ERCP后血胆红素降至接近正常，下列关于后续治疗的选择，合理的是（ ）。
A. 该患者总的治疗原则为姑息治疗，以局部治疗为主
B. 仅行壶腹部放疗
C. 如患者一般情况差（PS＞2），可考虑分子靶向治疗，首选易瑞沙
D. 如患者一般情况可，可以全身化疗为主，配合局部姑息性放疗
E. 该患者总的治疗原则以最大限度杀灭肿瘤，争取达到PR或CR
参考答案：D
【解析】 晚期胰腺癌预后极差，中位生存时间约3～6月，放化疗疗效较差，因此治疗上以姑息治疗为原则。

74. 如果明确诊断并排除远处转移，最积极的治疗手段是（ ）。
A. 胆总管切除
B. 胰十二指肠切除
C. 胆囊切除
D. 剖腹探查
E. 肝癌根治术
参考答案：B
【解析】 没有试题分析

75. 下列检查项目对帮助明确诊断最有价值的是（ ）。
A. ERCP
B. 腹部平片
C. MRI
D. 大便常规＋隐血
E. PET
参考答案：A
【解析】 没有试题分析

76. 如果该患者接受手术治疗，那么术后可能出现的并发症是（ ）。
A. 肝炎
B. 脂肪泻
C. 胰瘘
D. 肠梗阻
E. 胆总管狭窄
参考答案：C
【解析】 没有试题分析

77. 对于该患者的初步诊断是（ ）。
A. 胆总管癌
B. 壶腹癌
C. 胆囊癌
D. 肝癌
E. 十二指肠癌
参考答案：B
【解析】 没有试题分析

【题干六】
患者男性，44岁，右上腹疼痛半年，加重伴上腹部包块一月，伴腹胀、纳差、恶心，无呕吐、腹泻，大小便正常，体重下降5kg。既往有乙肝病史多年。查体：T36.7℃，P78次/分，R18次/分，BP110/70mmHg，全身皮肤无黄染，巩膜轻度黄染。腹平软，右上腹饱满，右上腹压痛，无肌紧张，肝脏肿大肋下5cm，边缘钝，质韧，有触痛，脾未及，腹部叩诊鼓音，无移动性浊音，肝上界叩诊在第五肋间，肝区叩痛，听诊肠鸣音8次/分。辅助检查：Hb89g/L，WBC5.6×10⁹/L，ALT84U/L，AST78U/L，TBIL30μmo1/L，DBIL10μmo1/L，ALP188U/L，GGT64U/L，AFP880ng/ml，CEA24mg/ml。B超：肝右叶实质性占位性病变8cm，肝内外胆管不扩张。

78. 如果排除远处转移，最积极的治疗手段是（ ）。
A. 肝癌根治术
B. 化疗
C. 介入
D. 放疗
E. 射频
参考答案：A
【解析】 没有试题分析

79. 下列哪项是对于该患者的初步诊断？（ ）
A. 肝血管瘤
B. 原发性肝癌
C. 胰腺癌
D. 转移性肝癌
E. 肝囊肿
参考答案：B
【解析】 没有试题分析

80. 如果需要明确该患者的组织类型，必须进行的检查项目是（ ）。
A. 消化道造影
B. CT
C. 肝穿刺活检
D. MRI
E. ERCP
参考答案：C
【解析】 为明确病理组织学类型，必须行肝穿刺活检。

81. 如果术后复查发现肝左叶约3cm肿块，AFP持续升高，无远处转移，肝功能基本正常，患者拒绝手术，下列治疗最合适的是（ ）。
A. 全肝照射
B. 化疗
C. 中药治疗
D. 肝左叶肿块X刀治疗
E. 干扰素
参考答案：D
【解析】 对于原发性肝癌复发的患者，如果复发病灶局限在肝脏，且肝功能正常、肝脏代偿能力可，可考虑再次手术治疗或精确放射治疗。

共享答案题

【选项一】
A. AFP＞200μg/L持续8周
B. AFP＜20μg/L
C. AFP＞200μg/L持续6周
D. AFP＞100μg/L
E. AFP＞500μg/L持续4周以上

82. 可以诊断肝细胞癌（ ）。
参考答案：E
【解析】 没有试题分析

83. 胆管细胞癌（ ）。
参考答案：B
【解析】 肝细胞癌AFP高，胆管细胞癌AFP不高。

【选项二】
A. 肝内胆管不扩张、肝管扩张、胆囊空虚
B. 肝内胆管扩张、肝管扩张、胆囊空虚
C. 肝内外胆管扩张、胆囊增大、胰管扩张（双管征）
D. 肝内外胆管扩张、胆囊增大
E. 肝内胆管不扩张、肝管不扩张、胆囊空虚

84. 梗阻部位在胆总管（ ）。
参考答案：D
【解析】 没有试题分析

85. 梗阻部位在肝总管或以上（ ）。
参考答案：B
【解析】 没有试题分析

86. 梗阻部位在壶腹部（ ）。
参考答案：C
【解析】 根据解剖结构，当梗阻部位在壶腹部时，除肝内胆管和胆囊扩张外，胰管也扩张。

【选项三】
A. 化学疗法
B. 食管癌切除加食管胃吻合术
C. 放射疗法
D. 胃造瘘术
E. 中医中药治疗

87. 男，50岁，进食后吞咽困难3个月余，目前不能进流质食物，人渐消瘦，体查：一般情况差，左锁骨上可扪及拇指头大小质硬淋巴结，食管镜活检报告为鳞癌Ⅲ级。应行（ ）。
参考答案：C
【解析】 对于晚期有远处转移的患者则应行化疗；如位于食管上段，则一般不行手术，而考虑放疗。

88. 男，50岁，进食后吞咽困难3个月余，食管吞钡X线片示中段食管有3cm长之环状狭窄，食管镜活检报告鳞癌Ⅱ级，锁骨上无肿大淋巴结，无声嘶，胸部X线片正常。应行（ ）。
参考答案：B
【解析】 没有试题分析

【选项四】
A. 气促、心悸、咯血
B. 进行性吞咽困难，多见40岁以上者
C. 声音嘶哑伴吞咽困难
D. 间歇性吞咽困难，多见于青壮年
E. 四肢周期麻痹

89. 食管癌晚期出现的症状是（ ）。
参考答案：C
【解析】 中期和晚期食管癌的主要临床表现略有不同，其中中期主要为进行性吞咽困难，而晚期除吞咽困难之外，往往伴随肿瘤其他组织器官的症状。

90. 食管癌中期出现的症状是（ ）。
参考答案：B
【解析】 没有试题分析

【选项五】
A. 全胃切除术
B. 食管下端和胃近端切除术
C. 胃癌根治切除术
D. 胃空肠吻合术
E. 放弃作根治术

91. 剖腹探查发现胃癌已广泛转移应行（ ）。
参考答案：E
【解析】 如果已出现广泛转移，则不宜行手术治疗。

92. 有幽门梗阻的晚期胃癌应行（ ）。
参考答案：D
【解析】 如伴有幽门梗阻的症状，可行胃空肠吻合术。

93. 胃体小弯部胃癌已侵犯胃底部应行（ ）。
参考答案：A
【解析】 如果肿瘤侵犯全胃或大部分胃组织，则可行全胃切除术。

94. 小弯高位胃癌和贲门癌应行（ ）。
参考答案：B
【解析】 如肿瘤位于胃底部，可行食管下端和胃近端切除术。

**第5章 乳腺癌（N=19）**

单项选择题

1. 乳腺癌主要发生于女性，男性乳腺癌约占（ ）。
A. 2%
B. 0.5%
C. 1.5%
D. 1%
E. 2.5%
参考答案：D
【解析】 乳腺癌是女性常见的恶性肿瘤之一，在男性中并不多见，还不到整个乳腺癌病例的1%。

2. 下列有关乳腺癌的危险因素的叙述错误的有（ ）。
A. 哺乳时间长
B. 月经初潮年龄早
C. 绝经早
D. 初次足月产年龄早
E. 有乳腺癌家族史
参考答案：A
【解析】 乳腺癌的病因尚不清楚，可能为多种因素共同作用的结果，其中最危险的因素是家族史。哺乳时间过短或不哺乳可能增加罹患乳腺癌的风险。

3. 乳腺癌最常见的转移部位是（ ）。
A. 脑
B. 骨
C. 肺
D. 肝
E. 肾上腺
参考答案：C
【解析】 乳腺癌细胞可经淋巴途径进入静脉，也可直接侵入血循环而致远处转移。最常见的远处转移部位为肺，其次为骨、肝、软组织、脑、肾上腺等。

4. TAM的疗效与下列因素有关的是（ ）。
A. 淋巴结状况
B. Her2表达状况
C. 肿块大小
D. ER状况
E. 绝经状况
参考答案：D
【解析】 乳腺癌内分泌治疗的疗效与受体状况明显相关，ER与PR均阳性者有效率为60%～70%，ER或PR阳性为30%左右，两者均阴性有效率小于10%。

5. 下列不属于保乳手术后放疗的绝对禁忌证的是（ ）。
A. 肿瘤切缘持续阳性
B. 不同象限内两个或两个以上肿瘤
C. 大乳房
D. 既往乳腺区曾行过放疗
E. 妊娠期妇女
参考答案：C
【解析】 保乳手术后放疗的绝对禁忌证包括：既往有胸壁或乳腺区放疗史；切缘持续阳性；妊娠期妇女；乳房照相提示为显微钙化的弥散性恶性肿瘤或为多中心病灶。大乳房及下垂乳房为相对禁忌证。

6. Her-2/neu过度表达的乳腺癌占所有乳腺癌的比例是（ ）。
A. 40%以上
B. 10%
C. 20%～30%
D. 10%～20%
E. 50%以上
参考答案：C
【解析】 乳腺癌患者中有25%～30%存在Her-2过度表达的情况。Her-2阳性患者的生存率明显低于阴性患者。

7. 下列不属于保乳手术适应证的是（ ）。
A. 肿瘤位于乳晕区
B. 原发肿瘤小于4cm
C. 局灶性显微钙化
D. 肿瘤多发但局限于一个象限
E. 无胶原性疾病
参考答案：A
【解析】 保乳术的适应证包括：乳腺单发病灶，最大径小于或等于3cm；乳腺肿瘤位于乳晕区以外的部位；腋窝无肿大淋巴结或有单个可活动的肿大淋巴结；无胶原血管病病史等。当肿块位于乳晕区时，为保乳的禁忌证。

8. 乳腺癌根治术后锁骨上/腋顶野的放射治疗剂量为（ ）。
A. 60Gy/6周
B. 40Gy/4周
C. 56Gy/5～6周
D. 50Gy/5周
E. 以上都不是
参考答案：D
【解析】 乳腺癌术后放射治疗的剂量为50Gy/5周。

9. 乳腺癌辅助性化疗的适应证除外（ ）。
A. ER-/PR-
B. N+
C. T＞1cm/SBRⅢ
D. N-
E. Her-2/neu强阳性
参考答案：D
【解析】 对于腋窝淋巴结阴性（N-）的患者，是否行辅助性治疗应根据预后判断指标。一般认为，肿块直径大于1.0cm、ER阴性、组织学分级为Ⅲ级、脉管癌栓、Her-2阳性应考虑给予术后辅助化疗。

10. 希罗达对阿霉素及紫杉醇治疗无效的转移性乳腺癌的有效率为（ ）。
A. 30%
B. ＜10%
C. 20%
D. 15%
E. 35%
参考答案：C
【解析】 抗代谢药5-Fu的口服衍生物希罗达对阿霉素及紫杉醇治疗无效的转移性乳腺癌的有效率为20%。

11. 关于新辅助化疗的描述错误的是（ ）。
A. 可减少远处转移
B. 可消灭微小转移灶
C. 化疗后临床和病理上的反应可帮助术后治疗方案的选择
D. 可缩小肿瘤，便于手术
E. 对患者而言，除了化疗药物的不良反应外，没有其他风险
参考答案：E
【解析】 新辅助化疗在缩小肿瘤，消灭转移灶的同时，部分患者可能存在对新辅助化疗方案不敏感，导致肿块继续增大，延误手术治疗时间。

12. 患者女性，67岁，发现乳腺包块月余，伴乳头溢液，X线图像如下，最有可能的诊断是（ ）。
A. 乳腺囊肿
B. 乳腺增生
C. 乳腺纤维瘤
D. 乳腺癌
E. 乳腺炎
参考答案：D
【解析】 乳腺肿块，可见钙化，腺体破坏，乳腺癌。乳腺增生、乳腺纤维瘤和乳腺囊肿无钙化，同时乳腺炎病史不符合，也不表现为肿块。

13. 患者，女性，39岁，4年前曾行乳腺癌根治术，目前出现左髋区疼痛，X线示左髋骨和股骨头出现骨质破坏，在下列情况下不首选内分泌治疗的是（ ）。
A. 出现骨和软组织转移
B. 仅ER阳性
C. 仅PR阳性
D. 辅助治疗后DFS（无病生存期）＞2年
E. ER、PR均阴性
参考答案：E
【解析】 若患者ER、PR均阴性或内脏转移、或皮肤受侵伴淋巴管转移、或脑转移、DFS＜2年，既往内分泌治疗无效应首选化疗。

14. 女性，42岁，左乳房外上象限4cm×3cm肿块，同侧腋窝淋巴结多个肿大融合，病理检查结果提示为左乳浸润性导管癌，13枚腋窝淋巴结中4枚阳性示阳性，ER＋＋，PR－，Her-2/neu－，该患者的临床分期考虑为（ ）。
A. ⅢA期
B. Ⅰ期
C. ⅡB期
D. ⅡA期
E. ⅢB期
参考答案：A
【解析】 患者为T2N2M0，为ⅢA期。乳腺癌T2的定义：肿瘤的最大径大于2.0cm，小于等于5.0cm。N2的定义：4～9个腋窝淋巴结转移。

共享题干题

【题干】
患者女性，37岁，左乳房外上象限4cm×3cm肿块，距乳头距离5cm，可推动，但是患者双手叉腰时肿块活动度明显受限，左腋窝未扪及肿大淋巴结。

15. 该患者在接受根治术后3年出现头痛、恶心，MRI提示脑部多发转移灶，此时首选的治疗是（ ）。
A. 内分泌治疗
B. 手术治疗
C. 放射治疗
D. 化学治疗
E. 赫赛汀治疗
参考答案：C
【解析】 乳腺癌患者出现单发脑转移者，可考虑行局部手术，但对多发脑转移者，首选放射治疗。

16. 若该患者病理检查结果提示为左乳浸润性小叶癌，15枚腋窝淋巴结中2枚阳性示阳性，ER（＋＋），PR（－），Her-2/neu（＋＋＋），该患者的临床分期考虑为（ ）。
A. ⅢA期
B. Ⅰ期
C. ⅡB期
D. ⅡA期
E. ⅢB期
参考答案：C
【解析】 T2N1M0患者的临床分期为ⅡB期。

17. 下列哪项是该患者最佳的定性诊断方法（ ）。
A. 近红外线扫描
B. 粗针穿刺活检
C. 切取活检
D. 钼靶X线摄片
E. 细针穿刺细胞学检查
参考答案：C
【解析】 病理检查是乳腺癌诊断的金标准。对乳腺癌患者的诊断最可靠的方法是组织病理学。

共享答案题

【选项】
A. 50%
B. 小于10%
C. 30%
D. 20%
E. 60%～70%

18. 乳腺癌患者ER或PR阳性，内分泌治疗有效率为（ ）。
参考答案：C
【解析】 没有试题分析

19. 乳腺癌患者ER与PR均阴性，内分泌治疗有效率为（ ）。
参考答案：B
【解析】 内分泌治疗的疗效与受体状况明显相关。ER与PR均阳性者有效率为60%～70%，ER或PR为阳性为30%，两者均阴性有效率小于10%。

**第6章 泌尿系统和男性生殖系统肿瘤（N=70）**

单项选择题

1. 据统计肾癌发病占全身恶性肿瘤的（ ）。
A. 4%
B. 1%
C. 3%
D. 2%
E. 5%
参考答案：C
【解析】 没有试题分析

2. 在我国，泌尿系统最常见的肿瘤是（ ）。
A. 睾丸癌
B. 肾癌
C. 前列腺癌
D. 膀胱癌
E. 肾盂癌
参考答案：D
【解析】 在我国，最常见的泌尿系统肿瘤是膀胱癌，其次是肾癌、肾盂癌。

3. 最常见的膀胱恶性肿瘤的病理类型是（ ）。
A. 鳞癌
B. 尿路上皮癌
C. 黏液腺癌
D. 腺癌
E. 透明细胞癌
参考答案：B
【解析】 最常见的膀胱恶性肿瘤的病理类型是尿路上皮癌，占95%以上，且绝大多数是移行细胞乳头状肿瘤，鳞癌和腺癌各占2%～3%。

4. 膀胱癌最常见的首发症状是（ ）。
A. 无痛性血尿
B. 尿路刺激症状
C. 下腹肿块
D. 排尿困难
E. 下腹肿块
参考答案：A
【解析】 无痛性肉眼血尿和镜下血尿是最常见的症状。

5. 关于肾癌的流行病学统计，下列正确的是（ ）。
A. 与摄入脂肪过高无关
B. 美洲发病率最高
C. 高发年龄以50～60岁居多
D. 农村高于城市
E. 占全身恶性肿瘤的10%
参考答案：C
【解析】 肾癌的发病率城市高于农村，与摄入脂肪过高有关，占全身恶性肿瘤的3%。高发年龄50～60岁，在瑞典和冰岛发病率最高。

6. 下列疾病临床分期都为Ⅱ~Ⅲ期时预后最好的是（ ）。
A. 睾丸精原细胞瘤
B. 肝癌
C. 食管癌
D. 胃癌
E. 肺癌
参考答案：A
【解析】 Ⅰ期睾丸精原细胞瘤中90%以上可治愈，因此其预后最好，即使是Ⅱ~Ⅲ期其5年生存率也明显高于其他肿瘤。

7. 睾丸肿瘤中最常见的病理类型是（ ）。
A. 卵黄囊瘤
B. 畸胎瘤
C. 精原细胞瘤
D. 胚胎癌
E. 绒毛膜上皮癌
参考答案：C
【解析】 睾丸肿瘤中生殖细胞肿瘤占95%，而生殖细胞肿瘤中，精原细胞瘤及非精原细胞瘤约各占一半。精原细胞瘤是最常见的类型。

8. 晚期睾丸肿瘤最常见的转移部位是（ ）。
A. 骨
B. 肝脏
C. 肺
D. 肾脏
E. 脑
参考答案：C
【解析】 睾丸肿瘤最常见的转移部位是肺，其次为肝、脑、骨、肾等。

9. Ⅰ期睾丸肿瘤首选的治疗方式是（ ）。
A. 分子靶向治疗
B. 手术
C. 化疗
D. 放疗
E. 内分泌治疗
参考答案：B
【解析】 第一步治疗应该行经腹股沟根治性睾丸切除术，既是治疗同时也取得病理诊断。

10. 睾丸精原细胞瘤1期患者术后放疗靶区是（ ）。
A. 纵隔淋巴结
B. 对侧睾丸
C. 腹主动脉旁淋巴结
D. 腹股沟淋巴结
E. 髂血管旁淋巴结
参考答案：C
【解析】 没有试题分析

11. 睾丸肿瘤腹主动脉旁照射野的上界为（ ）。
A. T11下缘
B. T8下缘
C. T10下缘
D. T9下缘
E. T12下缘
参考答案：C
【解析】 没有试题分析

12. 睾丸肿瘤腹主动脉旁照射野的下界为（ ）。
A. L4下缘
B. L1下缘
C. L3下缘
D. L2下缘
E. L5下缘
参考答案：E
【解析】 没有试题分析

13. 前列腺癌发病率最高的地区是（ ）。
A. 东南亚
B. 中国
C. 非洲
D. 北美
E. 澳大利亚
参考答案：D
【解析】 前列腺癌在北美和西欧发病率最高，非洲和亚洲最低。

14. 下列属于前列腺癌最佳初筛方法的是（ ）。
A. 前列腺穿刺活检和血清PSA检测
B. CT和MRI
C. 直肠指诊和血清PSA检测
D. X线检查和前列腺穿刺活检
E. PET-CT
参考答案：C
【解析】 直肠指诊可发现80%的前列腺癌，而血清PSA的特异性较好，两者结合应用，既简便易行，又成本低廉，是前列腺癌的最佳初筛方法。

15. 下列最常出现骨转移的疾病是（ ）。
A. 宫颈癌
B. 胃癌
C. 食管癌
D. 前列腺癌
E. 胃肠道间质瘤
参考答案：D
【解析】 各肿瘤的骨转移发生率：胃癌13%，前列腺癌65%～75%，食管癌5%～7%，宫颈癌5%，而胃肠道间质瘤较少出现腹腔外转移。

16. 前列腺癌行外照射放疗的合适体位是（ ）。
A. 侧卧位
B. 仰卧位
C. 截石位
D. 俯卧位
E. 端坐位
参考答案：B
【解析】 没有试题分析

17. 下列哪项是激素不敏感型前列腺癌的标准化疗方案？（ ）
A. 含雌二醇氮芥方案
B. 含米托蒽醌方案
C. 含多西他赛方案
D. 含阿霉素方案
E. 以上都不是
参考答案：C
【解析】 没有试题分析

18. 前列腺癌发生骨转移时最常见于下列哪种类型？（ ）
A. 病理性骨折
B. 溶骨型
C. 混合型
D. 成骨型
E. 骨髓纤维化
参考答案：D
【解析】 前列腺癌的骨转移以成骨型为主，约占80%。

19. 患者男性，74岁，进行性排尿困难4月，MR图像如下，最有可能的诊断是（ ）。
A. 膀胱癌
B. 前列腺增生
C. 前列腺肉瘤
D. 前列腺癌
E. 慢性前列腺炎
参考答案：D
【解析】 前列腺8cm肿块，可见分叶。T1WI稍低信号，T2WI稍高信号，增强不均匀强化，侵犯膀胱，典型前列腺癌。

20. 患者男性，66岁，反复无痛性肉眼血尿3周，CT图像如下，最有可能的诊断是（ ）。
A. 肾盂癌
B. 肾囊肿
C. 肾癌
D. 肾错构瘤
E. 肾脓肿
参考答案：C
【解析】 反复无痛性肉眼血尿，右肾4cm肿块，平扫等密度，动脉期明显强化，静脉期不均匀强化，典型肾癌。

21. 患者男性，76岁，反复无痛性肉眼血尿3月，MR图像如下，最有可能的诊断是（ ）。
A. 膀胱癌
B. 前列腺癌
C. 膀胱息肉
D. 膀胱炎
E. 精囊腺癌
参考答案：A
【解析】 膀胱5cm肿块，T1WI稍低信号，T2WI等信号，增强不均匀强化，典型膀胱癌。

22. 患者男性，55岁，反复发热，出现肉眼血尿，伴腰痛，查体肾区叩痛（－），未扪及肿块，小便示：红细胞2＋，行肾脏彩超和CT提示：左肾实质性包块。最有可能的诊断是（ ）。
A. 肾盂癌
B. 肾癌
C. 肾母细胞瘤
D. 肾囊肿
E. 肾转移瘤
参考答案：B
【解析】 没有试题分析

23. 患者男性，45岁，因"反复肉眼血尿，发现左肾区占位"，完善术前检查后于全麻下行"根治性肾切除术+肿大淋巴结清扫术"，术中见肿瘤大小约3cm×5cm，侵及肾上腺，肾周淋巴结1/3枚阳性，术后病检提示：肾透明细胞癌。该患者疾病分期是（ ）。
A. T4N1M0
B. T1N0M0
C. T3N1M0
D. T2N1M0
E. T2N2M0
参考答案：C
【解析】 没有试题分析

24. 患者女性，56岁，因肉眼血尿就诊，行肾脏彩超及CT发现右肾实质性占位，大小约3cm，左肾结石，轻度肾盂积水。该患者适合的手术方式是（ ）。
A. 保留肾单位手术＋淋巴结清扫术
B. 单纯全肾切除术
C. 根治性肾切除术
D. 保留肾单位手术
E. 部分肾切除术
参考答案：D
【解析】 没有试题分析

25. 患者男性，60岁，无痛性全程肉眼血尿，伴尿频症状，尿脱落细胞学见：癌细胞。行膀胱镜示：膀胱颈、三角区见褐色团块状结节，表面有溃疡，边缘隆起水肿。该患者最适合的手术方式是（ ）。
A. 膀胱部分切除术＋盆腔淋巴结清扫术
B. 经尿道膀胱肿瘤切除术
C. 根治性膀胱全切除术
D. 膀胱部分切除术
E. 膀胱切开肿瘤切除术
参考答案：C
【解析】 没有试题分析

26. 患者男性，50岁，无痛性全程肉眼血尿，行膀胱镜示：膀胱顶壁见一单发、粉红色、蒂细长物，似水草在水中漂荡。该患者最适合的手术方式是（ ）。
A. 膀胱部分切除术＋盆腔淋巴结清扫术
B. 经尿道膀胱肿瘤切除术
C. 根治性膀胱全切除术
D. 膀胱部分切除术
E. 膀胱切开肿瘤切除术
参考答案：B
【解析】 没有试题分析

27. 患者男性，60岁，一年来进行性排尿困难，近一月右侧腰痛，查体浅表淋巴结无肿大，右腰区叩击痛(+)，直肠指诊触及前列腺呈结节状，质硬。对疾病定性有重要意义的检测指标是（ ）。
A. 血CEA
B. 血Bun
C. 血清PSA
D. 血Cr
E. 血AFP
参考答案：C
【解析】 没有试题分析

共享题干题

【题干一】
患者男性，60岁，无痛性全程肉眼血尿，伴腰痛，消瘦，体重下降；查体：腹软，未扪及明显包块，肾区叩痛（－）。全血细胞计数白细胞：13×10⁹/L，中性粒细胞85%，肾功能肌酐60μmo1/L，尿素氮6.1mg/L，血沉20mm/h，尿常规：红细胞（＋＋），尿细胞学（－）。

28. 为帮助诊断首先考虑要做的检查是（ ）。
A. 静脉尿路造影
B. 膀胱镜
C. 腹部CT
D. 泌尿系统彩超
E. PET
参考答案：D
【解析】 没有试题分析

29. 该患者最有可能的诊断是（ ）。
A. 肾母细胞瘤
B. 肾癌
C. 膀胱癌
D. 肾盂癌
E. 肾囊肿
参考答案：B
【解析】 没有试题分析

30. 若该患者准备行手术治疗，根治性手术应包括的范围除外（ ）。
A. 肾蒂淋巴结
B. 肾周筋膜
C. 肾和肾上腺
D. 肾周脂肪
E. 输尿管
参考答案：E
【解析】 没有试题分析

【题干二】
患者男性，45岁，无痛性全程肉眼血尿，伴尿频，尿痛，下腹疼痛，查体：腹软，未扪及明显包块，肾区叩痛（－）。全血细胞计数白细胞：16×10⁹/L，中性粒细胞90%，肾功能肌酐55μmo1/L，尿素氮4.5mg/L，血沉5mm/h，尿常规：红细胞（＋＋＋），尿细胞学（－）。

31. 目前对于该疾病化疗的有效率应为（ ）。
A. 40%～50%
B. 10%～20%
C. 30%～40%
D. 20%～30%
E. 50%～60%
参考答案：A
【解析】 没有试题分析

32. 若患者需要做全膀胱切除术，手术适应证不包括（ ）。
A. 多发性的非浸润膀胱癌
B. 位于膀胱颈，三角区的浸润性癌
C. 巨大浸润性癌
D. 膀胱鳞癌
E. 高级别肿瘤伴发原位癌
参考答案：A
【解析】 没有试题分析

33. 该患者可以做下列检查以明确诊断，除外（ ）。
A. CT
B. 膀胱镜
C. 逆行尿路造影
D. 静脉尿路造影
E. 核素肾图
参考答案：E
【解析】 没有试题分析

【题干三】
患者男性，60岁，因"进行性排尿困难半年"入院，直肠指诊触及前列腺呈结节状，质硬，血清PSA升高，前列腺穿刺活检示前列腺癌，盆腔CT示前列腺癌，局限于左叶内，盆腔淋巴结无肿大，其余检查未发现异常。

34. 该患者的临床分期属于（ ）。
A. T4N0M0
B. T1N0M0
C. T3N0M0
D. T2N0M0
E. T1N1M0
参考答案：D
【解析】 该患者肿块局限于前列腺内，未发现淋巴结转移及远处转移，分期应为T2N0M0。

35. 该患者适合的治疗是（ ）。
A. 化疗
B. 观察
C. 前列腺切除术
D. 去势
E. 前列腺电切术
参考答案：C
【解析】 没有试题分析

36. 若患者拒绝上述治疗，可考虑的治疗是（ ）。
A. 化疗
B. 观察
C. 根治性放疗
D. 去势
E. 前列腺切除术
参考答案：C
【解析】 前列腺癌分期为T1～2N0M者，可选择前列腺切除术或根治性放疗。

【题干四】
患者男性，25岁，1个月前出现左睾丸疼痛，并可触及左侧睾丸肿块约2cm×1cm×2cm大小，浅表淋巴结未触及肿大，行睾丸切除术病检为精原细胞瘤。

37. 在检测肿瘤标志物时做法错误的是（ ）。
A. 需检测PSA
B. 动态观察对了解治疗效果有益
C. 需检测AFP
D. 需检测β-hCG
E. 需检测LDH
参考答案：A
【解析】 睾丸肿瘤患者常规行β-hCG、AFP及LDH检测，PSA对前列腺癌意义较大，精原细胞瘤无需检测PSA。

38. 该类患者五年生存率约为（ ）。
A. 80%
B. 15%
C. 60%
D. 45%
E. 95%
参考答案：A
【解析】 睾丸精原细胞瘤ⅡB期5年生存率约为75%～90%。

39. 进一步检查发现腹膜后淋巴结肿大约3cm×2cm×2cm，胸腔未见异常，按Royal Marsden医院分期为（ ）。
A. ⅡC期
B. Ⅰ期
C. ⅡB期
D. ⅡA期
E. Ⅲ期
参考答案：C
【解析】 膈下淋巴结转移，病灶直径2～5cm，分期为ⅡB。

40. 术后合适的辅助治疗是（ ）。
A. 观察
B. 化疗
C. 内分泌治疗
D. 放疗
E. 免疫治疗
参考答案：D
【解析】 睾丸精原细胞瘤患者术后分期Ⅰ、ⅡA、ⅡB者应行辅助放疗。

【题干五】
患者男性，65岁，无痛性肉眼血尿，肾脏彩超示：左肾实质性占位，腹部CT示：左肾近皮质缘有一2cm×3cm大小低密度灶，强化不明显，向肾外突出，腹主动脉旁见一个软组织密度结节影。

41. 该患者手术病检示：透明细胞癌。术中肿瘤大小为3cm×3cm，肾周脂肪组织，腹主动脉旁淋巴结1枚，活检发现癌组织。该患者的临床分期属于（ ）。
A. T3aN1M0
B. T1aN1M0
C. T2N1M0
D. T1bN1M0
E. T3bN1M0
参考答案：A
【解析】 没有试题分析

42. 该患者下一步首选的治疗策略是（ ）。
A. 根治性肾切除术＋放疗
B. 根治性肾切除术
C. 保留肾单位术
D. 根治性肾切除术＋肿大淋巴结切除术
E. 化疗
参考答案：D
【解析】 没有试题分析

43. 若患者行术前检查时发现右肺单发结节，下一步最佳的治疗方案是（ ）。
A. 分子靶向治疗
B. 根治性肾切除术＋转移灶切除术
C. 免疫治疗
D. 全身化疗
E. 放疗
参考答案：B
【解析】 没有试题分析

【题干六】
患者男性，52岁，无痛性肉眼血尿伴尿频，尿急症状，尿常规示：白细胞（＋＋＋），红细胞(+++)，尿蛋白（－），尿细胞学发现癌细胞。查体：肾区叩痛（－），双合诊未发现明显异常。

44. 患者下一步要做的检查是（ ）。
A. 尿路平片
B. IVP
C. 膀胱镜
D. 泌尿系统B超
E. 腹部盆部CT
参考答案：C
【解析】 没有试题分析

45. 患者行手术治疗，术中见肿瘤位于膀胱颈、三角区，褐色团块状结节，表面有溃疡。术后病检示：肿瘤侵犯深肌层，淋巴结（－）。患者的分期属于（ ）。
A. T3aN0M0
B. T1aN0M0
C. T2bN0M0
D. T2aN0M0
E. T3bN0M0
参考答案：C
【解析】 没有试题分析

46. 若该患者准备行放疗，下面关于放疗不正确的是（ ）。
A. 局部剂量为65Gy
B. 设野上界位于第4、5腰椎之间
C. 设野两侧达真骨盆外1～2cm
D. 设野下缘位于闭孔下线
E. 盆腔野剂量40～50Gy
参考答案：B
【解析】 没有试题分析

【题干七】
患者男性，60岁，因"进行性排尿困难半年，背痛1月"入院，直肠指诊触及前列腺呈结节状，质硬，血清PSA升高，前列腺穿刺活检示前列腺癌，盆腔CT示前列腺癌侵犯直肠，盆腔淋巴结肿大，骨ECT示胸椎多个椎体放射性浓聚，余检查未发现异常。

47. 为明确背痛原因需要进行的最佳检查是（ ）。
A. 肺部CT
B. 胸椎MRI
C. PET-CT
D. 胸椎CT
E. 以上均可
参考答案：B
【解析】 前列腺癌易出现骨转移。MRI是确诊骨转移性肿瘤的首选方法。

48. 若患者确诊多个胸椎转移，分期属于（ ）。
A. T4N1M1
B. T1N0M0
C. T3N0M1
D. T2N1M0
E. T3N1M1
参考答案：A
【解析】 没有试题分析

49. 需要立即进行的治疗是（ ）。
A. 内分泌治疗
B. 手术内固定
C. 放疗
D. 全身化疗
E. 同步放化疗
参考答案：C
【解析】 胸椎为承重骨，发生转移时容易出现椎体骨折、神经根压迫等骨相关事件，多个椎体转移首选椎体放疗预防骨相关事件。

【题干八】
患者男性，27岁，因左侧睾丸疼痛发现睾丸肿块，行睾丸切除术，病检为非精原细胞瘤，进一步行胸部CT检查发现右肺转移。

50. 首选的化疗方案是（ ）。
A. CVP
B. BEP
C. GP
D. VIP
E. NP
参考答案：B
【解析】 BEP方案用于睾丸非精原细胞瘤的一线化疗时，无瘤生存率为83%，已取代PVB成为睾丸非精原细胞瘤首选化疗方案。

51. 化疗后复查胸CT仍有病灶残留，下一步治疗是（ ）。
A. 换用VIP方案化疗
B. 继续原方案化疗
C. 局部放疗
D. 肺叶切除术
E. 观察
参考答案：A
【解析】 常规联合化疗方案治疗失败时，换用VIP方案作为补救治疗仍可使30%患者获得无瘤生存。

52. 经上述治疗达CR，半年后再次发现肺内复发，可选的化疗方案是（ ）。
A. 高剂量CBP＋VP16
B. VIP
C. EP
D. BEP
E. BVP
参考答案：A
【解析】 三线化疗可考虑高剂量CBP＋VP16化疗或联合自体造血干细胞移植。

共享答案题

【选项一】
A. 根治性膀胱切除术
B. 经尿道膀胱肿瘤切除术
C. 全膀胱切除术
D. 膀胱部分切除术
E. 膀胱切开肿瘤切除术

53. 侵犯膀胱颈、三角区的肿瘤应选择的术式是（ ）。
参考答案：C
【解析】 没有试题分析

54. Ta的表浅膀胱肿瘤应选择的术式是（ ）。
参考答案：B
【解析】 没有试题分析

55. 膀胱侧壁、顶部、底部单个局限的浸润性肿瘤应选择的术式是（ ）。
参考答案：D
【解析】 没有试题分析

【选项二】
A. 肿块、疼痛、膀胱刺激症状
B. 血尿、疼痛、肿块
C. 血尿、肿块
D. 血尿、膀胱刺激症状
E. 肿块、膀胱刺激症状

56. 上述哪项为膀胱癌的典型症状？（ ）
参考答案：D
【解析】 没有试题分析

57. 上述哪项为肾癌的三联征？（ ）
参考答案：B
【解析】 没有试题分析

【选项三】
A. 膀胱容量缩小，肿瘤附近黏膜皱缩、增厚、水肿、充血
B. 单发或多发粉红色、蒂细长、似水草在水中漂浮
C. 暗红色有浸润的团块状结节，呈暗红色，短蒂，表面覆有灰白色坏死组织，肿物活动性小
D. 膀胱表面黏膜上有突起的红色区域，外观与充血和增生的黏膜相似
E. 无蒂的、境界不清的褐色团块，坏死处呈溃疡，边缘形成水肿，有钙质沉着

58. 上述哪项为膀胱镜下乳头状癌的表现？（ ）
参考答案：B
【解析】 没有试题分析

59. 上述哪项为膀胱镜下原位癌的表现？（ ）
参考答案：D
【解析】 没有试题分析

60. 上述哪项为膀胱镜下浸润性癌的表现？（ ）
参考答案：A
【解析】 没有试题分析

61. 上述哪项为膀胱镜下T2期癌的表现？（ ）
参考答案：C
【解析】 没有试题分析

【选项四】
A. 40～45Gy
B. 20～30Gy
C. 35～40Gy
D. 30～35Gy
E. 45～50Gy

62. 睾丸精原细胞瘤Ⅰ期患者术后放疗剂量为（ ）。
参考答案：B
【解析】 没有试题分析

63. 小肠耐受剂量为（ ）。
参考答案：E
【解析】 没有试题分析

64. 睾丸精原细胞瘤Ⅱ期患者术后放疗剂量为（ ）。
参考答案：C
【解析】 没有试题分析

【选项五】
A. CEA
B. BHCG
C. CA125
D. AFP
E. 以上都不是

65. 睾丸非精原细胞瘤患者不表达而精原细胞瘤患者表达（ ）。
参考答案：E
【解析】 βHCG在精原细胞瘤和非精原细胞瘤都可能升高。

66. 睾丸精原细胞瘤和非精原细胞瘤患者都表达（ ）。
参考答案：B
【解析】 没有试题分析

67. 睾丸精原细胞瘤患者不表达而非精原细胞瘤患者表达（ ）。
参考答案：D
【解析】 没有试题分析

【选项六】
A. 6500～7000cGy
B. 3500～4000cGy
C. 5500～6000cGy
D. 4500～5000cGy
E. 7000～7600cGy

68. 直肠耐受剂量（ ）。
参考答案：C
【解析】 没有试题分析

69. 膀胱耐受剂量（ ）。
参考答案：C
【解析】 没有试题分析

70. 前列腺癌根治性放疗剂量（ ）。
参考答案：E
【解析】 没有试题分析

**第7章 恶性淋巴瘤（N=54）**

**单项选择题**

1. 我国霍奇金淋巴瘤的发病特点，下列错误的是（ ）。
   A. 发病年龄呈曲线双峰
   B. 发病率低于欧美国家
   C. 发病年龄曲线呈单峰
   D. 男性高于女性
   E. 随年龄增加逐渐升高
   **参考答案：A**
   **解析：** 霍奇金淋巴瘤高发区为北美、西欧，我国与欧美相比，发病率较低，男性高于女性，随年龄增长发病率逐渐升高，发病年龄曲线为单峰状态。
2. Burkitt淋巴瘤与下列感染有关的是（ ）。
   A. HP
   B. HTLV-1
   C. EBV
   D. HHV-8
   E. 衣原体
   **参考答案：C**
   **解析：** EBV感染与Burkitt淋巴瘤相关；HTLV-1感染与成人T细胞淋巴瘤相关；HHV-8感染是Kaposi肉瘤的病因，体腔淋巴瘤和胸腔积液淋巴瘤中常见该病毒感染，常见于HIV感染患者；HP与胃黏膜相关组织淋巴瘤相关；鹦鹉衣原体与眼附属器淋巴瘤相关。
3. 下列不属于经典型霍奇金淋巴瘤的是（ ）。
   A. 富于淋巴细胞的经典霍奇金淋巴瘤
   B. 结节硬化型
   C. 结节性淋巴细胞为主型霍奇金淋巴瘤
   D. 淋巴细胞衰减型
   E. 混合细胞型
   **参考答案：C**
   **解析：** 2000年WHO将霍奇金淋巴瘤分为结节性淋巴细胞为主型霍奇金淋巴瘤和经典型霍奇金淋巴瘤，后者分为：①结节硬化型；②混合细胞型；③淋巴细胞衰减型；④富于淋巴细胞的经典霍奇金淋巴瘤。
4. 富于淋巴细胞的经典型霍奇金淋巴瘤的RS细胞特点（ ）。
   A. 周围的淋巴细胞为反应T淋巴细胞
   B. CD30+
   C. CD20-
   D. CD15+
   E. 以上都正确
   **参考答案：E**
   **解析：** 富于淋巴细胞的经典型霍奇金淋巴瘤的RS细胞形态学与结节性淋巴细胞为主型霍奇金淋巴瘤相似，但有经典霍奇金淋巴瘤的免疫表型：CD30+，CD15+，CD20-，周围的淋巴细胞为反应T淋巴细胞。
5. 淋巴瘤患者进行脑脊液检查见于下列哪种情况？（ ）
   A. 睾丸受侵
   B. IV期患者
   C. 中枢神经系统受侵
   D. 骨髓侵犯
   E. 以上都正确
   **参考答案：E**
   **解析：** IV期患者和骨髓、睾丸、中枢神经系统受侵，应行脑脊液细胞学检查。
6. 霍奇金淋巴瘤预后不良因素下列正确的是（ ）。
   A. 血清白蛋白＞40g/L
   B. 年龄≤45岁
   C. HB＜105g/L
   D. 女性
   E. WBC≥10×10⁹/L
   **参考答案：C**
   **解析：** 霍奇金淋巴瘤预后不良因素：年龄≥45岁、男性、IV期、HB＜105g/L、血清白蛋白＜40g/L、WBC≥15×10⁹/L、淋巴细胞计数＜0.6×10⁹/L或白细胞分类淋巴细胞＜0.08。
7. 非霍奇金淋巴瘤国际预后指数IPI得分为3，应为（ ）。
   A. 高危组
   B. 低危组
   C. 中高危组
   D. 中低危组
   E. 极高危组
   **参考答案：C**
   **解析：** NHL的国际预后指数IPI根据危险程度分4组：低危组：0～1分，中低危组：2分，中高危组：3分，高危组：4～5分。
8. 淋巴瘤的B症状（AJCC第6版）下列正确的是（ ）。
   A. 体重减轻＜10%
   B. 皮肤瘙痒
   C. 乏力
   D. 出汗
   E. 无法解释的发热，体温超过38℃
   **参考答案：E**
   **解析：** AJCC第6版中规定B症状：无法解释的发热，体温超过38℃；需要更换床单和被罩的大汗；诊断前6个月无法解释的体重减轻，超过平时体重的10%。
9. 早期预后良好型霍奇金淋巴瘤的最佳治疗方式（ ）。
   A. 联合化疗＋受累野放疗
   B. 单纯放疗
   C. 手术
   D. 单纯化疗
   E. 手术＋受累野放疗
   **参考答案：A**
   **解析：** 综合目前研究结果，NCCN建议早期预后良好型霍奇金淋巴瘤的最佳治疗模式为联合化疗（ABVD方案4周期）联合受累野放疗（20～36Gy）。
10. NHL淋巴结受侵下列检查最可靠的是（ ）。
    A. 淋巴结穿刺
    B. CT
    C. 临床体检
    D. X线片
    E. 淋巴结活检
    **参考答案：E**
    **解析：** 淋巴结活检是诊断的金标准。
11. 早期预后不良型霍奇金淋巴瘤（伴巨大纵隔肿块）的最佳治疗方法是（ ）。
    A. 6周期化疗＋受累野放疗
    B. 扩大野单纯放疗
    C. 4周期化疗＋扩大野放疗
    D. 4周期化疗＋受累野放疗
    E. 6周期化疗＋扩大野放疗
    **参考答案：A**
    **解析：** 目前文献表明6周期ABVD方案或Stanford V方案联合受累野放疗（20～36Gy）是较为理想的选择。
12. 放疗技术中，斗篷野加锄形野照射属于（ ）。
    A. 扩大野照射
    B. 全淋巴结照射
    C. 受累野照射
    D. 次全淋巴结照射
    E. 区域野照射
    **参考答案：D**
    **解析：** 淋巴结照射方式包括扩大野照射、区域野照射、受累野照射，扩大野照射包括全淋巴结照射：斗篷野＋倒Y野（锄形野＋盆腔野）；次全淋巴结照射：斗篷野＋锄形野。区域野包括受累淋巴结区域和相邻的淋巴结区域，受累野包括受累的淋巴结区域。
13. 复发和难治性霍奇金淋巴瘤影响化疗的不良预后因素包括（ ）。
    A. 原发耐药
    B. B症状
    C. 老年患者
    D. 结外侵犯
    E. 以上都正确
    **参考答案：E**
14. 对于原发耐药的难治性霍奇金淋巴瘤应采取的治疗是（ ）。
    A. HDC/AHSCT
    B. 手术
    C. 解救化疗
    D. 放疗
    E. 放疗＋化疗
    **参考答案：A**
    **解析：** 原发耐药患者预后差，中位生存期＜1.5年，常规化疗无效，大剂量化疗/自体干细胞移植（HDC/AHSCT）是最佳选择。
15. I期前体淋巴母细胞淋巴瘤治疗方案首选（ ）。
    A. 手术
    B. Hyper-CVAD方案化疗
    C. 受累野放疗
    D. CHOP方案化疗
    E. 手术＋放疗
    **参考答案：B**
    **解析：** 无论I期还是IV期前体淋巴母细胞淋巴瘤，均应按全身性疾病治疗，治疗方案首选Hyper-CVAD方案化疗。
16. 针对I~II期弥漫大B细胞淋巴瘤，治疗方案首选（ ）。
    A. CHOP方案化疗3～4周期联合受累野放疗
    B. R-CHOP方案化疗3～4周期
    C. CHOP方案化疗3～4周期
    D. R-CHOP方案化疗3～4周期联合受累野放疗
    E. R-CHOP方案化疗3～4周期联合扩大野放疗
    **参考答案：D**
    **解析：** R-CHOP方案化疗3～4周期联合受累野放疗是治疗首选，若不能耐受放疗，可行R-CHOP方案化疗6～8周期；若不能进行利妥昔单抗治疗，可用CHOP方案代替R-CHOP方案化疗。
17. 下列哪项是III~IV期无症状滤泡性淋巴瘤治疗原则？（ ）
    A. 氟达拉滨
    B. 观察和等待
    C. CVP方案化疗
    D. CHOP方案化疗
    E. 氟达拉滨＋利妥昔单抗
    **参考答案：B**
    **解析：** 无症状FL患者采取观察和等待原则，对于肿瘤进展，可选择以上选项化疗。
18. 下列哪项是局限IE期鼻腔和鼻型NK/T细胞淋巴瘤治疗原则？（ ）
    A. 手术
    B. 单纯化疗
    C. 单纯放疗
    D. 化疗加受累野放疗
    E. 手术＋放疗
    **参考答案：C**
    **解析：** 鼻腔和鼻型NK/T细胞淋巴瘤对放疗敏感，但对化疗相对抗拒。多项研究表明，化疗加放疗未改善早期鼻腔和鼻型NK/T细胞淋巴瘤的生存率。放射治疗是早期鼻腔和鼻型NK/T细胞淋巴瘤的主要治疗手段。
19. I期胃黏膜相关组织淋巴瘤幽门螺杆菌（HP）阳性患者，首选下列哪项治疗？（ ）
    A. 手术＋化疗
    B. 抗HP治疗
    C. 手术
    D. 放疗
    E. 手术＋放疗
    **参考答案：B**
    **解析：** I期胃黏膜相关组织淋巴瘤，HP阳性患者可首选抗HP治疗，HP阴性患者也可先试用抗HP治疗，或者首选放疗。
20. 下列不属于缓慢进展性淋巴瘤的是（ ）。
    A. 滤泡性淋巴瘤
    B. 小淋巴细胞淋巴瘤
    C. 淋巴浆细胞淋巴瘤
    D. 边缘带B细胞淋巴瘤
    E. 前体淋巴母细胞淋巴瘤
    **参考答案：E**
    **解析：** 前体淋巴母细胞淋巴瘤属高度进展性淋巴瘤。
21. EORTC定义预后不良早期霍奇金淋巴瘤除外（ ）。
    A. 无B症状但ESR＜50
    B. 年龄≥50岁
    C. ≥4个部位受侵
    D. 大肿块或大纵隔
    E. 有B症状且ESR＞30
    **参考答案：A**
    **解析：** 预后不良早期霍奇金淋巴瘤包括年龄≥50岁、大肿块或大纵隔、≥4个部位受侵、无B症状但ESR＞50或有B症状且ESR＞30。
22. 下列哪项是淋巴瘤患者往往合并贫血的原因？（ ）
    A. 溶血
    B. 骨髓侵犯
    C. 脾功能亢进
    D. 以上均正确
    E. A+B+C不完全正确
    **参考答案：D**
23. 霍奇金淋巴瘤预后极好型有（ ）。
    A. 病理为淋巴细胞为主型或结节硬化型
    B. 女性
    C. 年龄＜40岁
    D. 临床IA期
    E. 以上均正确
    **参考答案：E**
24. 晚期霍奇金淋巴瘤可采用下列的化疗方案是（ ）。
    A. HDC/AHSCT
    B. ABVD
    C. BEACOPP
    D. Stanford V
    E. 以上均正确
    **参考答案：E**
25. 以下器官受侵不属于霍奇金淋巴瘤IV期的是（ ）。
    A. 皮肤
    B. 肝
    C. 骨髓
    D. 肺
    E. 以上都不对
    **参考答案：A**
    **解析：** 肝、肺、骨髓侵犯均属IV期，皮肤受侵属IE期。
26. 女，26岁，右颈单一无痛性淋巴结肿大为2.5cm×3.0cm，活动欠佳。活体组织检查发现包膜完整，无出血及坏死。镜下见其结构已破坏，大量的束状纤维组织增生及散在一些大细胞。其胞质丰富、透明、核大，有多个核仁，并与周围形成透明的空隙。同时还可见嗜酸性粒细胞、浆细胞及少量的中性粒细胞。该病最可能的诊断是（ ）。
    A. 淋巴结反应性增生
    B. 淋巴结转移性癌
    C. 非霍奇金淋巴瘤
    D. 淋巴结炎
    E. 霍奇金病，结节硬化型
    **参考答案：E**
27. 患者男性，15岁，胸闷胸痛2月，伴低热月余，患者行MR扫描图像如下，最有可能的诊断是（ ）。
    A. 纵隔畸胎瘤
    B. 淋巴瘤
    C. 中央型肺癌
    D. 纵隔脓肿
    E. 神经纤维瘤
    **参考答案：B**
    **解析：** 前中纵隔淋巴结肿块融合成片，T1WI稍低信号，T2WI高信号，淋巴瘤。纵隔脓肿可见高热和脓腔。中央型肺癌伴肺不张，纵隔畸胎瘤可见脂肪和其他的不均匀信号，而神经纤维瘤是位于后纵隔的结块。
28. 患者，女性，28岁，高热伴右颈包块1月余，CT检查发现颈部、纵隔及腹膜后淋巴结多发肿大，肝脾多发占位性病变，边缘强化，考虑为转移瘤。右颈淋巴结活检示弥漫大B细胞淋巴瘤，正确的诊断分期属于（ ）。
    A. IVA期
    B. II期
    C. IIIB期
    D. IIIA期
    E. IVB期
    **参考答案：E**
    **解析：** 患者，全身多发淋巴结肿大，位于横膈两侧，同时伴肝脏受侵，属于IV期淋巴瘤，此外高热1月余，为B症状。因此诊断为非霍奇金淋巴瘤（DLBCL）IVB期。
29. 患者，男性，65岁，因腹痛3月余入院，入院查体：ECOG评分1级，双颈可及多个肿大淋巴结，心肺无异常，腹软，肝肋下5指可及，脾胁下3指可及，移动性浊音阴性。B超检查示腹膜后多发淋巴结肿大，肝脏多发性占位病变，结合病史考虑转移瘤。血清LDH正常。颈淋巴结穿刺细胞学示"非霍奇金淋巴瘤"。该患者根据IPI应分为（ ）。
    A. 高危组
    B. 低危组
    C. 中高危组
    D. 中低危组
    E. 极高危组
    **参考答案：C**
    **解析：** NHL的IPI包括：年龄≥60岁，LDH升高，一般状况（ECOG≥2），临床分期III~IV期，淋巴结受侵区域＞1个，每项预后因素记1分，低危=0～1，中低危=2分，中高危=3分，高危=4～5分。患者年龄>60岁，临床分期IV期，受侵淋巴结区域＞1处，为3项危险因素，3分为中高危组。
30. 患者，男性，58岁，因右腋窝肿块2周入院，手术后切除病检示：外周T细胞淋巴瘤（非特异型）。完善相关检查，胸部、腹部CT均未见异常，骨髓细胞学未见淋巴瘤侵犯。下一步治疗方案应选择（ ）。
    A. CHOP方案化疗3周期＋右腋窝放疗
    B. CHOP方案化疗6周期
    C. CHOP方案化疗3周期
    D. CHOP方案化疗6周期＋右腋窝放疗
    E. 右腋窝放疗
    **参考答案：D**
    **解析：** 外周T细胞淋巴瘤（非特异型）对放化疗敏感，尽管该患者分期属于I期，但属于侵袭性淋巴瘤，仍应进行CHOP方案化疗6～8周期，再进行受累野（右腋窝）放疗。
31. 患者男性，21岁，因"胸闷、气促1周"入院，胸部CT示纵隔巨大肿块，纵隔镜活检示"霍奇金淋巴瘤"，诊断为霍奇金淋巴瘤IA期，行6周期ABVD方案化疗后，患者胸闷症状消失，复查胸部CT示纵隔肿块较前缩小。下一步治疗方案选择（ ）。
    A. 手术
    B. 继续ABVD方案化疗2周期
    C. 更换化疗方案
    D. 纵隔放疗
    E. 自体干细胞移植
    **参考答案：D**
    **解析：** 纵隔霍奇金淋巴瘤化疗6周期后仍有残留，可对纵隔行放疗，争取达到完全缓解。此时更换化疗方案和自体干细胞移植都不恰当。

**共享题干题**

【题干】患者女性，65岁，咽痛1月余，查体：右侧扁桃体肿大，鼻咽部未见异常，双肺呼吸音清晰，心脏听诊无异常。腹软，无压痛，未触及腹部包块，肝脾肋下未及，移动性浊音阴性。双下肢无浮肿。扁桃体活检示弥漫大B细胞淋巴瘤，胸部CT及腹部CT未见异常。

1. 化疗后进行放疗，则恰当的放疗剂量选择（ ）。
   A. 40～45Gy
   B. 25～30Gy
   C. 35～40Gy
   D. 30～35Gy
   E. 45～50Gy
   **参考答案：A**
   **解析：** 该患者属于弥漫大B细胞淋巴瘤，治疗原则为全身化疗联合受累野放疗，该类型淋巴瘤往往CD20阳性，化疗方案以CHOP方案为主，联合利妥昔单抗可进一步增高疗效。所以该患者选择R-CHOP方案，因患者年龄＞60岁，存在不良预后因素，化疗周期6～8周期为宜，若无不良预后因素，化疗3～4周期即可行放疗。因扁桃体属韦氏环淋巴瘤，放疗靶区需包括韦氏环（鼻咽、舌根、扁桃体），同时全颈也应包括在设野之内。放疗剂量在化疗基础上以40～45Gy为宜。
2. 该患者下一步如果进行化疗，最佳方案选择（ ）。
   A. R-CHOP
   B. MINE
   C. BACOP
   D. CHOP
   E. EPOCH
   **参考答案：A**
3. 若患者禁忌放疗，化疗以多少周期为宜？（ ）
   A. 8～12周期
   B. 3周期
   C. 6～8周期
   D. 4周期
   E. 12周期以上
   **参考答案：C**
4. 患者化疗后，若进行放疗，比较合理的设野方式是（ ）。
   A. 鼻前野
   B. 面颈联合野
   C. 面颈联合野＋下颈切线野
   D. 耳前野
   E. 全颈切线野
   **参考答案：C**

**共享答案题**

【选项】
A. 蕈样霉菌病
B. 胃黏膜相关组织淋巴瘤
C. Burkitt's淋巴瘤
D. 弥漫大B细胞淋巴瘤
E. 鼻腔NK/T细胞淋巴瘤

1. 以侵犯皮肤为主的淋巴瘤是（ ）。
   **参考答案：A**
2. 可以不用放化疗就能治愈的淋巴瘤是（ ）。
   **参考答案：B**
   **解析：** 胃黏膜相关组织淋巴瘤可通过抗幽门螺杆菌治愈；鼻腔NK/T细胞淋巴瘤以放射治疗为主，对化疗抗拒；Burkitt's淋巴瘤多见于非洲儿童，与EB病毒感染有关；蕈样霉菌病是原发于皮肤的低度恶性淋巴瘤；弥漫大B细胞淋巴瘤在我国属最常见的非霍奇金淋巴瘤，大多表达CD20，抗CD20的利妥昔单抗联用化疗可提高10%～20%的有效率。

【选项】
A. 蕈样霉菌病
B. 胃黏膜相关组织淋巴瘤
C. Burkitt's淋巴瘤
D. 鼻腔NK/T细胞淋巴瘤
E. 弥漫大B细胞淋巴瘤

1. 对放疗敏感，对化疗相对抗拒的侵袭性淋巴瘤是（ ）。
   **参考答案：D**
   **解析：** 胃黏膜相关组织淋巴瘤可通过抗幽门螺杆菌治愈；鼻腔NK/T细胞淋巴瘤以放射治疗为主，对化疗抗拒；Burkitt's淋巴瘤多见于非洲儿童，与EB病毒感染有关；蕈样霉菌病是原发于皮肤的低度恶性淋巴瘤；弥漫大B细胞淋巴瘤在我国属最常见的非霍奇金淋巴瘤，大多表达CD20，抗CD20的利妥昔单抗联用化疗可提高10%～20%的有效率。
2. 发生于非洲儿童的高度进展型淋巴瘤是（ ）。
   **参考答案：C**
3. 可使用利妥昔单抗治疗的进展型淋巴瘤是（ ）。
   **参考答案：E**

【选项】
A. EBV
B. 幽门螺杆菌
C. HHV-8
D. HTLV-1
E. 衣原体

1. 在胸腔积液淋巴瘤和体腔淋巴瘤中，常见感染是（ ）。
   **参考答案：C**
2. 成人T细胞淋巴瘤患者中可分离出（ ）。
   **参考答案：D**
   **解析：** EBV感染与Burkitt's淋巴瘤相关；HTLV-1感染与成人T细胞淋巴瘤相关；HHV-8感染是Kaposi肉瘤的病因，体腔淋巴瘤和胸腔积液淋巴瘤中常见该病毒感染，常见于HIV感染患者；幽门螺杆菌与胃黏膜相关组织淋巴瘤相关；鹦鹉衣原体与眼附属器淋巴瘤相关。

【选项】
A. 幽门螺杆菌
B. EBV
C. HHV-8
D. HTLV-1
E. 衣原体

1. 胃黏膜相关淋巴瘤与之有关（ ）。
   **参考答案：A**
2. 非洲儿童Burkitt's淋巴瘤常伴感染的病原体是（ ）。
   **参考答案：B**
   **解析：** EBV感染与Burkitt's淋巴瘤相关；HTLV-1感染与成人T细胞淋巴瘤相关；HHV-8感染是Kaposi肉瘤的病因，体腔淋巴瘤和胸腔积液淋巴瘤中常见该病毒感染，常见于HIV感染患者；幽门螺杆菌与胃黏膜相关组织淋巴瘤相关；鹦鹉衣原体与眼附属器淋巴瘤相关。
3. 与眼附属器淋巴瘤有关（ ）。
   **参考答案：E**

【选项】
A. 混合细胞型
B. 结节性淋巴细胞为主型
C. 结节硬化型
D. 淋巴细胞消减型
E. 淋巴细胞为主型

1. 镜下细胞呈"爆米花"型，通常找不到典型的RS细胞是（ ）。
   **参考答案：B**
2. 欧美等发达国家最常见的HD亚型，特征是腔隙型RS细胞（ ）。
   **参考答案：C**
   **解析：** 在组织病理学方面，目前公认的是"WHO霍奇金淋巴瘤分类（2000）"。与1994年欧美修订的REAL分类法相比，该分类系统将结节性淋巴细胞为主型霍奇金淋巴瘤（NLPHL）作为一种新的病理类型从淋巴细胞为主型中分离出来，而其他四种归为一个类型，即经典型（cHL）。cHL的标志是巨大的RS细胞，占受累组织浸润细胞的＜1%，通常散在于由T、B细胞，中性粒细胞，巨噬细胞及其他类型细胞组成的肿瘤微环境中，其来源仍不十分清楚。NLPHL与cHL明显不同，它的肿瘤细胞称作淋巴和组织细胞（L&H），具有特征性折痕，核仁不明显，细胞核呈扭曲、分叶状，即"爆米花（popcorn）样"特征，而典型的RS细胞罕见。欧美国家结节硬化型cHL常见，具有特征是腔隙型RS细胞；淋巴细胞为主型cHL镜下淋巴细胞占主导地位，CD30+，CD15+，CD20+/-；混合细胞型cHL镜下见典型的RS细胞，病变呈弥漫性；淋巴细胞消减型cHL镜下呈弥漫纤维化，含大量RS细胞和"肉瘤样"变异细胞。

【选项】
A. 混合细胞型
B. 结节性淋巴细胞为主型
C. 结节硬化型
D. 淋巴细胞为主型
E. 淋巴细胞消减型

1. 镜下呈弥漫纤维化，含大量RS细胞和"肉瘤样"变异细胞（ ）。
   **参考答案：E**
   **解析：** 在组织病理学方面，目前公认的是"WHO霍奇金淋巴瘤分类（2000）"。与1994年欧美修订的REAL分类法相比，该分类系统将结节性淋巴细胞为主型霍奇金淋巴瘤（NLPHL）作为一种新的病理类型从淋巴细胞为主型中分离出来，而其他四种归为一个类型，即经典型（cHL）。cHL的标志是巨大的RS细胞，占受累组织浸润细胞的＜1%，通常散在于由T、B细胞，中性粒细胞，巨噬细胞及其他类型细胞组成的肿瘤微环境中，其来源仍不十分清楚。NLPHL与cHL明显不同，它的肿瘤细胞称作淋巴和组织细胞（L&H），具有特征性折痕，核仁不明显，细胞核呈扭曲、分叶状，即"爆米花（popcorn）样"特征，而典型的RS细胞罕见。欧美国家结节硬化型cHL常见，具有特征是腔隙型RS细胞；淋巴细胞为主型cHL镜下淋巴细胞占主导地位，CD30+，CD15+，CD20+/-；混合细胞型cHL镜下见典型的RS细胞，病变呈弥漫性；淋巴细胞消减型cHL镜下呈弥漫纤维化，含大量RS细胞和"肉瘤样"变异细胞。
2. 镜下见典型的RS细胞，病变呈弥漫性（ ）。
   **参考答案：A**
3. 镜下淋巴细胞占主导地位，CD30+，CD15+，CD20+/-（ ）。
   **参考答案：D**

【选项】
A. 40～45Gy
B. 20～36Gy
C. 50～55Gy
D. 30～35Gy
E. 60Gy

1. 霍奇金淋巴瘤化疗后受累野放疗剂量（ ）。
   **参考答案：B**
   **解析：** 目前研究表明，在全身化疗基础上，霍奇金淋巴瘤和非霍奇金淋巴瘤局部放疗剂量均有降低趋势。霍奇金淋巴瘤化疗后，受累野放疗剂量可降至20～36Gy。非霍奇金淋巴瘤的最适剂量，不像霍奇金淋巴瘤那样明确，不同类型的非霍奇金淋巴瘤剂量亦不统一。研究发现，局部黏膜相关性淋巴样组织（MALT）淋巴瘤对于低剂量放疗有极好的反应。胃MALT淋巴瘤对放疗敏感，IE~IIE期胃MALT淋巴瘤放疗后5年总生存率和无病生存率分别超过90%和80%。多数侵袭性淋巴瘤，如最具代表性的弥漫性大B细胞淋巴瘤（DLBCL），不仅要通过全身化疗有效控制远处组织器官的亚临床转移，还需要通过放疗有效控制局部病变，化疗后放疗剂量可降至40～45Gy。鼻腔NK/T细胞淋巴瘤尽管是侵袭性淋巴瘤，但对化疗抗拒，临床I~II期患者，以化疗为首程治疗时，化疗后的完全缓解（CR）率仅为0%～59%，大部分低于40%，而以放疗为首程治疗的CR率达到52%～100%，放疗根治剂量为50～55Gy。
2. 早期胃黏膜相关组织淋巴瘤放疗剂量（ ）。
   **参考答案：D**

【选项】
A. 50～55Gy
B. 20～36Gy
C. 40～45Gy
D. 30～35Gy
E. 60Gy

1. 鼻腔NK/T细胞淋巴瘤放疗剂量（ ）。
   **参考答案：A**
   **解析：** 目前研究表明，在全身化疗基础上，霍奇金淋巴瘤和非霍奇金淋巴瘤局部放疗剂量均有降低趋势。霍奇金淋巴瘤化疗后，受累野放疗剂量可降至20～36Gy。非霍奇金淋巴瘤的最适剂量，不像霍奇金淋巴瘤那样明确，不同类型的非霍奇金淋巴瘤剂量亦不统一。研究发现，局部黏膜相关性淋巴样组织（MALT）淋巴瘤对于低剂量放疗有极好的反应。胃MALT淋巴瘤对放疗敏感，IE~IIE期胃MALT淋巴瘤放疗后5年总生存率和无病生存率分别超过90%和80%。多数侵袭性淋巴瘤，如最具代表性的弥漫性大B细胞淋巴瘤（DLBCL），不仅要通过全身化疗有效控制远处组织器官的亚临床转移，还需要通过放疗有效控制局部病变，化疗后放疗剂量可降至40～45Gy。鼻腔NK/T细胞淋巴瘤尽管是侵袭性淋巴瘤，但对化疗抗拒，临床I~II期患者，以化疗为首程治疗时，化疗后的完全缓解（CR）率仅为0%～59%，大部分低于40%，而以放疗为首程治疗的CR率达到52%～100%，放疗根治剂量为50～55Gy。
2. 弥漫大B细胞淋巴瘤化疗后受累野放疗剂量（ ）。
   **参考答案：C**

**第8章 骨与软组织肿瘤（N=81）**

**单项选择题**

1. 原发性骨肿瘤的发生率为（ ）。
   A. 2%～3%
   B. 10%～15%
   C. 1%～2%
   D. 5%～10%
   E. 3%～5%
   **参考答案：A**
2. 对于怀疑骨松质的病变，以下影像学检查最具优势的是（ ）。
   A. 骨扫描
   B. X线片
   C. MRI
   D. CT
   E. 以上均可
   **参考答案：C**
   **解析：** MRI具有组织分辨率高，多平面成像的特点，可以准确显示骨肿瘤在骨内和软组织的范围，以及骺软骨、关节软骨的破坏情况，并可早期发现邻近的跳跃转移灶。对于发现脊柱等骨松质的早期病变，MRI比X线片和CT更具有优势。
3. 下列属于诊断骨肿瘤最基本和首要影像学检查方法的是（ ）。
   A. 骨扫描
   B. X线片
   C. MRI
   D. CT
   E. 以上均可
   **参考答案：B**
4. 对于大多数原发性骨肿瘤，诊断依靠（ ）。
   A. 术中所见
   B. 临床表现
   C. 病理
   D. 影像学检查
   E. 临床表现、影像、病理结合
   **参考答案：E**
   **解析：** 明确诊断是合理治疗的前提，对原发骨肿瘤的诊断一般按如下程序进行：首先要判断病变是否为肿瘤，再判断其良恶性，最后确定其具体的病理类型。对于大多数原发性骨肿瘤，诊断必须坚持临床、影像和病理三结合的原则，违背此原则，单纯依靠其中任何一项都可能导致严重的误诊。
5. 了解骨肿瘤对软组织、软骨的破坏情况，最好选择的检查是（ ）。
   A. 骨扫描
   B. X线片
   C. MRI
   D. CT
   E. 以上均可
   **参考答案：C**
6. 以下关于原发性骨肿瘤活检术错误的是（ ）。
   A. 操作轻柔，严密止血
   B. 临床诊断为良性的骨肿瘤可以不行活检
   C. 活检通道应位于下次手术时可以连同肿瘤整块切除的部位
   D. 多采用套管针活检和切取式活检
   E. 必要时行免疫组化检查
   **参考答案：B**
   **解析：** 对所有怀疑为恶性以及很多怀疑为良性的原发性骨肿瘤，均需进行术前活检以获得病理学诊断，因为病理分型对制定治疗方案有重要的指导作用。
7. 软组织肿瘤除外以下哪种来源的肿瘤？（ ）
   A. 皮肤及附属器
   B. 纤维组织
   C. 滑膜组织
   D. 脂肪组织
   E. 脉管组织
   **参考答案：A**
   **解析：** 软组织是身体骨外的非上皮结缔组织，软组织位于表皮与实质脏器之间，它包括运动器官（肌肉及肌腱）及各种支持组织结构，如纤维组织、脂肪组织、滑膜组织以及营养这些结构的脉管组织。
8. 良性与恶性软组织肿瘤之比超过（ ）。
   A. 5：1
   B. 100：1
   C. 10：1
   D. 50：1
   E. 1：1
   **参考答案：B**
   **解析：** 绝大多数软组织肿瘤为良性，而软组织肉瘤则较为罕见。良性与恶性软组织肿瘤之比超过100：1。
9. 最多见的良性软组织肿瘤是（ ）。
   A. 脂肪瘤
   B. 神经鞘瘤
   C. 纤维组织细胞性和纤维性肿瘤
   D. 血管性肿瘤
   E. 平滑肌瘤
   **参考答案：A**
   **解析：** 良性软组织肿瘤中，至少1/3为脂肪瘤，1/3为纤维组织细胞性和纤维性肿瘤，10%为血管性肿瘤，5%为神经鞘瘤。
10. 总体来说软组织肉瘤最好发于下列哪个部位？（ ）
    A. 小腿
    B. 躯干
    C. 前臂
    D. 大腿和骨盆带
    E. 上臂和肩胛带
    **参考答案：D**
    **解析：** 软组织肉瘤大约半数涉及大腿和骨盆带，四分之一涉及上臂和肩胛带，其余的涉及前臂、小腿和躯干，很少累及手和足（例外情况多见于滑膜肉瘤、上皮样肉瘤和透明细胞肉瘤）。
11. 软组织肉瘤最常见下列哪种转移部位？（ ）
    A. 脑
    B. 肺
    C. 骨
    D. 肝
    E. 淋巴结
    **参考答案：B**
    **解析：** 软组织肉瘤远地转移主要发生于肺，转移至其他器官通常发生于疾病的晚期。
12. 恶性软组织肿瘤的常见特点除外（ ）。
    A. 持续性疼痛或压痛
    B. 质硬，固定
    C. 常位于深筋膜浅层
    D. 肿块持续增大
    E. 肿块常较大
    **参考答案：C**
    **解析：** 恶性软组织肿瘤多数位于深筋膜深层。
13. 对于大多数软组织肿瘤来说，首选的治疗手段是（ ）。
    A. 生物治疗
    B. 放疗
    C. 手术
    D. 化疗
    E. 热疗
    **参考答案：C**
    **解析：** 恰当的外科治疗至今仍是软组织肿瘤最主要和有效的治疗手段，是整体治疗决策的基础。
14. 下列哪种软组织肿瘤化疗有肯定价值？（ ）
    A. 血管肉瘤
    B. 脂肪肉瘤
    C. 滑膜肉瘤
    D. 平滑肌肉瘤
    E. 尤文肉瘤
    **参考答案：E**
    **解析：** 全身化疗的价值取决于肿瘤的类型。对于尤文肉瘤，应该进行新辅助化疗和辅助化疗。而其他组织类型的软组织肉瘤，全身化疗的价值仍有争议。
15. 肢体软组织肉瘤术后肿瘤瘤床的放疗剂量至少需要达到（1.8～2.0Gy/F）（ ）。
    A. 60～65Gy
    B. 45～50Gy
    C. 55～60Gy
    D. 50～55Gy
    E. 65～70Gy
    **参考答案：A**
    **解析：** 软组织肉瘤术后放疗初始阶段应该根据不同的临床情况控制在肿瘤边缘外5～10cm范围，剂量应达到DT50Gy，肿瘤外2cm边缘区内照射量一般不应低于60～62Gy，高危复发危险区域剂量可达65～70Gy。
16. 肢体软组织肉瘤放疗描述错误的是（ ）。
    A. 包括整个长骨
    B. 尽量避免跨关节照射
    C. 保留一侧正常皮肤、皮下组织
    D. 包括整个受侵肌肉起始点
    E. 注意保护重要的器官功能
    **参考答案：A**
    **解析：** 软组织肉瘤放疗照射野的设计既要考虑肿瘤的控制，又要考虑到使正常组织得到更好的保护。尽量避免整个长骨和跨关节的照射，以降低关节僵直和骨折的风险，保持良好的功能。放射野不应横贯肢体横径，至少应保留有2～3cm的条形区不受照射，以利于体液的回流。
17. 下列哪种骨肿瘤化疗不作为主要治疗手段？（ ）
    A. 骨髓瘤
    B. 尤文肉瘤
    C. 软骨母细胞瘤
    D. 恶性纤维组织细胞瘤
    E. 骨肉瘤
    **参考答案：C**
    **解析：** 软骨母细胞瘤为起源于幼稚软骨细胞（软骨母细胞）的骨良性肿瘤，以手术治疗为主。
18. 具有良性组织学表现，却有少数患者出现肺转移的骨肿瘤是（ ）。
    A. 骨髓瘤
    B. 尤文肉瘤
    C. 软骨母细胞瘤
    D. 恶性纤维组织细胞瘤
    E. 骨巨细胞瘤
    **参考答案：E**
    **解析：** 骨巨细胞瘤性质在骨肿瘤中很独特，在组织学完全为良性者，却可以发生肺转移（占总数的1%～6%），而肺转移的组织学表现与原发瘤相同，仍为典型的骨巨细胞瘤组织像，此即所谓的"良性肺转移"。
19. 临床上作出骨肉瘤诊断时，已发生肺的微小转移的比例是（ ）。
    A. 60%～70%
    B. 30%～40%
    C. 50%～60%
    D. 40%～50%
    E. 80%以上
    **参考答案：E**
    **解析：** 骨肉瘤最突出的特点是转移极早，这也是单纯截肢术不能提高生存率的原因，目前对骨肉瘤的治疗强调手术、化疗及放疗配合的综合治疗。
20. 以下药物不属于骨肉瘤化疗的主要有效药物的是（ ）。
    A. 足叶乙甙
    B. 多柔比星
    C. 甲氨喋呤
    D. 异环磷酰胺
    E. 顺铂
    **参考答案：A**
21. 发生骨转移最多见于下列哪种恶性肿瘤？（ ）
    A. 甲状腺癌
    B. 肺癌
    C. 肾癌
    D. 前列腺癌
    E. 乳腺癌
    **参考答案：E**
    **解析：** 发生骨转移最多见的恶性肿瘤依次是乳腺癌、肺癌、前列腺癌、甲状腺癌、肾癌，总体约占80%。
22. 骨转移最常发生于下列哪个部位？（ ）
    A. 脊柱和骨盆
    B. 股骨近端
    C. 肋骨
    D. 肩胛骨
    E. 颅骨
    **参考答案：A**
    **解析：** 骨转移最常发生的部位是脊柱和骨盆，其次是股骨近端，肋骨和肩胛骨。
23. 骨肉瘤综合治疗5年生存率可达（ ）。
    A. 40%～50%
    B. 75%左右
    C. 50%～60%
    D. 60%～70%
    E. 30%
    **参考答案：B**
    **解析：** 1970年以前，骨肉瘤的治疗原则是高位截肢术，5年生存率在15%以下，至70年代后期治疗规范化，强调联合用药、新辅助化疗和剂量强度原则，5年生存率可达75%左右。
24. 有关恶性骨肿瘤保肢手术的描述错误的是（ ）。
    A. 必须保证各个部位均达到广泛或根治的切除边界
    B. 将肿瘤骨和宿主骨一起整块切除
    C. 重建后的肢体功能应不低于假肢
    D. 肢体重建包括骨与关节重建和软组织的重建
    E. 已替代截肢手术
    **参考答案：E**
    **解析：** 恶性骨肿瘤的手术方式可以分为保肢手术和截肢手术两种，具有各自的适应证。病理分级为G1或G2、单发病变、MRI显示无局部软组织侵犯的患者可以考虑保肢治疗，而且目前在联合化疗及放疗的前提下，保肢手术已经得到广泛应用。但是对于肿瘤有局部软组织侵犯、血管受侵等情况下，保肢治疗要谨慎。截肢术适用于就诊较晚，破坏广泛和对其他治疗无效的恶性骨或软组织肿瘤。
25. 下列哪种骨肿瘤对放疗较为敏感？（ ）
    A. 骨恶性纤维组织细胞瘤
    B. 尤文肉瘤
    C. 骨肉瘤
    D. 软骨肉瘤
    E. 软骨母细胞瘤
    **参考答案：B**
26. 下列哪种骨肿瘤对放化疗均不敏感？（ ）
    A. 骨恶性纤维组织细胞瘤
    B. 尤文肉瘤
    C. 骨肉瘤
    D. 软骨肉瘤
    E. 以上均不是
    **参考答案：D**
    **解析：** 软骨肉瘤是恶性的成软骨性肿瘤，化疗和放疗疗效差，治疗以手术为主。
27. 恶性骨肿瘤的预后因素有（ ）。
    A. 转移部位
    B. 原发肿瘤大小
    C. 原发肿瘤位置
    D. 组织学分级
    E. 以上均是
    **参考答案：E**
    **解析：** 恶性骨肿瘤已知的预后因素如下：①T1期肿瘤的预后比T2期好；②组织学病理分级低级（G1、G2）好；③原发肿瘤的位置是一个预后因素，在解剖上能切除部位的肿瘤比不能切除部位的肿瘤预后好。脊柱比骨盆的肿瘤预后差；④对于骨肉瘤和尤文肉瘤来说，原发肿瘤的大小也是一个预后因素，肿瘤最大径≤8cm的尤文肉瘤比＞8cm者预后好，最大径≤9cm的骨肉瘤比＞9cm者好；⑤某些特定部位的转移预后差，骨和肝转移比肺转移预后差，多发肺转移比单发肺转移预后差。
28. 骨转移癌进行手术的目的主要是（ ）。
    A. 固定病理骨折和解除脊髓压迫
    B. 减少肿瘤负荷
    C. 减轻疼痛
    D. 根治性手术
    E. 取得组织学诊断
    **参考答案：A**
    **解析：** 骨转移癌的治疗应与原发癌及其他并发症一同考虑，治疗目的是减轻症状、提高生活质量。外科手术治疗的适应证主要是针对承重骨或关节破坏明显，影响患者活动，包括对即将发生病理性骨折部位的预防性固定和已经发生的病理性骨折部位的固定处理等。
29. 软组织肉瘤最常见的转移方式是（ ）。
    A. 直接侵犯
    B. 血道转移
    C. 种植转移
    D. 淋巴道转移
    E. 以上均是
    **参考答案：B**
30. 有关横纹肌肉瘤描述正确的是（ ）。
    A. 对于胚胎型横纹肌肉瘤，单纯放疗可以根治
    B. 主要有两种类型：黏液型和胚胎型
    C. 主要转移部位是肺和骨
    D. 高度恶性，生长迅速
    E. 淋巴结转移罕见
    **参考答案：D**
    **解析：** 横纹肌肉瘤有两种主要的类型：成年型（多形性型）和儿童型（胚胎型）。恶性程度高，术后易出现局部复发和远处转移，5年生存率为12%～23%，主要转移部位是肺，淋巴结转移也可能出现，转移至骨者罕见。治疗需要综合治疗，放疗多数针对术后残留或局部复发灶，单纯放疗仅限于有手术禁忌或拒绝手术、多次复发不能手术、肿瘤巨大或已有淋巴结转移的姑息性放疗。
31. 淋巴结转移率最高的软组织肉瘤是（ ）。
    A. 滑膜肉瘤
    B. 脂肪肉瘤
    C. 平滑肌肉瘤
    D. 横纹肌肉瘤
    E. 纤维肉瘤
    **参考答案：A**
    **解析：** 大约10%的软组织肉瘤患者会出现区域淋巴结转移，而滑膜肉瘤的淋巴结转移率为25%。
32. 有关Enneking外科分期系统描述正确的是（ ）。
    A. 良性肿瘤外科分级为G1
    B. 基于外科分级（G）、解剖定位（T）和有无转移（M）
    C. 良性肿瘤均为1期
    D. 淋巴结转移属于T分期
    E. A表示原发肿瘤位于间室外
    **参考答案：B**
    **解析：** Enneking外科分期系统中，无区域淋巴结和远处器官转移者为M0，出现其一者为M1；良性肿瘤外科分级为G0，分期1期为潜伏性，2期为活动性，3期为侵袭性；间室指宿主对于肿瘤的生长有天然屏障，在软组织为关节囊、大的筋膜间隙、韧带、肌腱等致密组织，在骨为皮质骨和关节软骨，这些屏障未成的可限制肿瘤局部延伸的解剖区域，称为间室，A表示原发肿瘤位于间室内。
33. 化疗已作为某些恶性骨肿瘤的重要治疗手段，其中包括（ ）。
    A. 骨髓瘤
    B. 尤文肉瘤
    C. 骨肉瘤
    D. 骨恶性纤维组织细胞瘤
    E. 以上均是
    **参考答案：E**
34. 有关骨巨细胞瘤描述错误的是（ ）。
    A. X线表现为溶骨性破坏
    B. 可能来自髓腔内未分化的间叶组织细胞
    C. 好发于长管状骨已闭合的骺板处
    D. 好发年龄为20～40岁之间
    E. 因为系潜在恶性肿瘤，手术多选择广泛性或根治性切除
    **参考答案：E**
    **解析：** 骨巨细胞瘤是以手术治疗为主的肿瘤，尽管肿瘤性质为潜在恶性肿瘤，但目前主张彻底的刮除术。
35. 关于MTS分期系统描述正确的是（ ）。
    A. 危险因素包括病变数目、大小和出现转移的时间
    B. 强调提供有用的预后信息
    C. 每一期分别根据是否存在危险因素分为A期和B期
    D. 分为Ⅰ~Ⅳ期
    E. 以上都不对
    **参考答案：A**
    **解析：** 骨骼肌肉肿瘤学会（MTS）采用的三期系统，强调的目的是指导选择恰当的手术方法和（或）辅助治疗方法。分为Ⅰ、Ⅱ、Ⅲ期，Ⅰ、Ⅱ期再根据病变的部位细分为间室内（A）和间室外（B），Ⅲ期病变根据出现危险因素的多少分为ⅢA和ⅢB，这些危险因素包括：①一个以上的病变；②病变直径大于2cm；③原发病变经局部控制后，18个月内出现转移。
36. 好发于青少年的骨肿瘤除外（ ）。
    A. 尤文肉瘤
    B. 软骨母细胞瘤
    C. 骨肉瘤
    D. 骨样骨瘤
    E. 软骨肉瘤
    **参考答案：E**
    **解析：** 骨肉瘤发病年龄有两个高峰，第一个高峰在青少年（在此时期，长骨的生长发育迅速），第二高峰在老年时期（此时期与Paget病和既往放疗史相关的第二原发肿瘤发病率升高有关）。软骨肉瘤多发生于中老年人，患者年龄多在30～60岁之间。
37. 目前认为需要常规进行术后化疗的肿瘤是（ ）。
    A. 脂肪肉瘤
    B. 滑膜肉瘤
    C. 尤文肉瘤
    D. 软组织恶性纤维组织细胞瘤
    E. 软骨肉瘤
    **参考答案：C**
38. 黑色素瘤多发生于白种人，尤其是有日晒史的人群，而我国发病率较低，约为（ ）。
    A. 5～6/10万
    B. 0.4～0.5/10万
    C. 10～15/10万
    D. 0.2/10万
    E. 16/10万
    **参考答案：B**
    **解析：** A为大洋洲的死亡率，C为美国的发病率，D是我国黑色素瘤的死亡率，E为澳大利亚昆士兰邦的发病率。
39. 恶性黑色素瘤最常发生于下列哪种部位？（ ）
    A. 女性四肢的皮肤
    B. 眼球的虹膜
    C. 消化道黏膜
    D. 脑膜的脉络丛
    E. 眼球的睫状体
    **参考答案：A**
    **解析：** 恶性黑色素瘤可发生于身体任何部位，最常见于女性的四肢和男性的躯干，多发生于皮肤和邻近皮肤的黏膜。
40. 最常见的皮肤恶性黑色素瘤类型是（ ）。
    A. 雀斑型黑色素瘤
    B. 结节型黑色素瘤
    C. 乳头状黑色素瘤
    D. 肢端雀斑型黑色素瘤
    E. 表浅转移型黑色素瘤
    **参考答案：E**
    **解析：** 结节型黑色素瘤占所有皮肤恶性黑色素瘤的15%～30%，雀斑型及肢端雀斑型所占比例均不超过10%，而表浅转移型黑色素瘤占70%。
41. 下列不是早期皮肤恶性黑色素瘤皮损表现的是（ ）。
    A. 表面不规则隆起、粗糙、脱屑和渗液等
    B. 颜色改变，尤其以蓝黑色、灰色、棕色和杂色最为重要
    C. 周边参差不齐，呈锯齿状
    D. 可扪及明显增大、质硬、不规则肿块，压痛明显
    E. 皮损迅速增大、持续瘙痒、结痂或出现卫星结节等
    **参考答案：D**
    **解析：** 皮肤恶性黑色素瘤与含色素的皮损密切相关，早期的变化主要为皮肤颜色改变，损伤区域的外形、面积和性质异常改变，不会出现明显的包块。
42. 下列无色素性黑色素瘤的鉴别对象不包括（ ）。
    A. 软组织肉瘤
    B. 低分化腺癌
    C. 淋巴瘤
    D. 未分化癌
    E. 生殖细胞癌
    **参考答案：A**
    **解析：** 无色素性黑色素瘤较少见，多发生予中年女性，部位多位于皮肤和黏膜的交界处，镜下多为小细胞性恶性肿瘤，往往与低分化腺癌、未分化癌、淋巴瘤难以区分，部分上皮样瘤细胞还需与鳞癌或生殖细胞癌鉴别。
43. 下列关于恶性黑色素瘤TNM分期原则的描述中不正确的是（ ）。
    A. 原发肿瘤发生溃疡时，所有Ⅰ、Ⅱ、Ⅲ期患者的分期均应升高一期
    B. T分期的确定根据黑色素瘤的厚度和溃疡形成，而不是侵犯范围（T1期除外）
    C. M分期根据远处转移部位和血清乳酸脱氢酶的水平
    D. N分期根据转移淋巴结的数目而不是大小
    E. 出现卫星灶转移或第一站淋巴结之前的淋巴转移均应归入ⅢB期
    **参考答案：E**
    **解析：** 目前最常用的AJCC2002年修订的第六版TNM分期方法中明确指出出现卫星灶转移或第一站淋巴结之前的淋巴转移均应归入ⅢC期。
44. 下列关于恶性黑色素瘤TNM分期中区域淋巴结分期描述中不正确的是（ ）。
    A. 4个以上淋巴结转移，或融合的淋巴结转移，或局部淋巴结转移伴有卫星灶转移或第一站淋巴结之前的淋巴转移为N3
    B. 2～5个区域淋巴结转移或存在淋巴内转移为N2
    C. 无局部淋巴结转移但有卫星灶转移或第一站淋巴结之前的淋巴转移为N2c
    D. 一个淋巴结转移且临床隐性转移（镜下转移）为Nla
    E. 区域淋巴结无法评价为Nx
    **参考答案：B**
    **解析：** 根据目前最常用的AJCC2002年修订的第六版TNM分期中区域淋巴结分期描述中2～3个区域淋巴结转移或存在淋巴内转移为N2。
45. 下列关于恶性黑色素瘤病理分期中ⅢC期的正确描述是（ ）。
    A. T1～4aN2bM0
    B. T1～4bN2aM0
    C. T1～4bN2bM0
    D. T1～4aNlbM0
    E. T1～4α/βN2cM0
    **参考答案：C**
    **解析：** 根据目前最常用的AJCC2002年修订的第六版TNM分期中关于恶性黑色素瘤病理分期中ⅢC期的正确描述是T1～4bN2bM0。
46. 下列关于恶性黑色素瘤的治疗原则中不正确的是（ ）。
    A. 对于Ⅳ期患者，以化疗和生物治疗为主，还可以考虑局部放疗、减症手术和姑息手术
    B. 对于原位恶性黑色素瘤，推荐的切除边缘距病灶或活检瘢痕0.5～1cm
    C. 对于ⅡBⅡC术后患者和Ⅲ期已行淋巴结清扫术的患者，术后辅助生物治疗有助于降低复发和转移的风险
    D. 对于未行活检但临床怀疑淋巴结受侵的患者，须切除原发灶但无需进行区域淋巴结清扫
    E. 对于仅有皮肤或淋巴结转移的患者，有时也可以考虑局部放疗
    **参考答案：D**
    **解析：** 对于临床怀疑淋巴结受侵或者活检证实淋巴结受侵的患者，均建议切除原发灶的同时进行区域淋巴结清扫，以降低转移或复发的几率。
47. 对于ⅡBⅡC术后患者和Ⅲ期已行淋巴结清扫术的患者，术后辅助治疗有助于降低复发和转移的风险，其中主要使用的是（ ）。
    A. 白介素－2
    B. 丝裂霉素
    C. 干扰素α-2b
    D. 白介素－11
    E. 甲氨蝶呤
    **参考答案：C**
    **解析：** 对于ⅡBⅡC术后患者和Ⅲ期已行淋巴结清扫术的患者，术后全身辅助治疗主要使用的是干扰素α-2b，它能够提高20%～30%的无复发生存率和30%的总生存率。
48. 晚期恶性黑色素瘤患者的治疗主要以化疗配合生物治疗，在生物治疗方式（用药）中除外（ ）。
    A. 白介素－2
    B. LAK细胞
    C. 干扰素α-2b
    D. 肿瘤坏死因子
    E. 疫苗
    **参考答案：D**
    **解析：** 晚期恶性黑色素瘤患者的治疗推荐用大剂量干扰素α-2b，但由于毒性大，耐受性不佳，临床实际应用采用中低剂量，但同时往往合并使用其他生物治疗如白介素－2、LAK细胞或疫苗，但不包括肿瘤坏死因子。
49. 下列哪种药物是晚期恶性黑色素瘤的化疗主要用药？（ ）
    A. 达卡巴嗪
    B. 阿霉素
    C. 紫杉醇
    D. 吉西他滨
    E. 顺铂
    **参考答案：A**
    **解析：** 目前美国FDA批准用于晚期黑色素瘤治疗的药物为达卡巴嗪，含有达卡巴嗪的化疗方案客观有效率约为15%～20%。
50. 下列关于恶性黑色素瘤手术治疗原则描述不正确的是（ ）。
    A. 对于厚度超过4mm的病灶，建议争取手术切除边缘距病灶或活检疤痕3cm
    B. 对于厚度小于1mm的病灶，手术切除边缘距病灶或活检疤痕0.5～1cm
    C. 对于厚度2～4mm的病灶，建议手术切除边缘距病灶或活检疤痕2～3cm
    D. 对于厚度1～2mm的病灶。尽可能争取手术切除边缘距病灶或活检疤痕2cm
    E. 厚度小于1mm的病灶，建议行区域淋巴结清扫
    **参考答案：E**
    **解析：** 厚度小于1mm的病灶，很少存在淋巴结转移，不必行区域淋巴结清扫。其余厚度超过1mm的病灶，可以考虑选择性区域淋巴结清扫。
51. 根据AJCC调查Ⅰ期恶性黑色素瘤患者5年生存率约为（ ）。
    A. 65%
    B. 90%
    C. 50%
    D. 75%
    E. 43%
    **参考答案：B**
    **解析：** 恶性黑色素瘤各期患者间生存率存在显著差别，其五年生存率在Ⅰ期患者约为90%，Ⅱ期约为70%，Ⅲ期约为50%，Ⅳ期约为10%。
52. Clark根据恶性黑色素瘤肿瘤浸润深度对病变进行了组织学分类，以下描述不正确的是（ ）。
    A. Ⅳ度为病变局限于网状层
    B. Ⅰ度为病变局限于表皮内
    C. Ⅲ度为侵犯至真皮乳头与网状层交界处
    D. Ⅱ度为侵犯至真皮乳头
    E. Ⅴ度为病变达皮下组织
    **参考答案：A**
    **解析：** 恶性黑色素瘤的皮肤浸润深度对预后有明显影响，故了解其分类较重要，Ⅳ度应为病变已穿透网状层。
53. 恶性黑色素瘤远处转移最常见下列哪个部位？（ ）
    A. 脑
    B. 肝脏
    C. 骨
    D. 肺
    E. 腹膜后
    **参考答案：D**
    **解析：** 恶性黑色素瘤远处转移最常见的部位是肺，约为70%～87%，其次为肝脑54%～77%，第三为脑36%～54%，骨、腹膜后均较少见。
54. 下列对恶性黑色素瘤放射治疗的描述正确的是（ ）。
    A. 浸润性病变厚度小于1mm者射野边界在瘤外1cm
    B. 不需要非常高的分次剂量
    C. 射野边界在原位癌为瘤外0.5cm
    D. 总剂量为50～60Gy，原位癌为50Gy为宜
    E. 浸润性病变厚度1～4mm或大于4mm者射野边界在瘤外2cm
    **参考答案：B**
    **解析：** 早期认为大分割剂量放疗对恶性黑色素瘤效果较好，但近期研究认为小于或等于3Gy/次的有效率更高。放疗总剂量应为70～80Gy，而原位癌为70Gy为宜。其余选项射野范围均偏小，A应为2cm，C应为1cm，E应为3cm。
55. 下列对恶性黑色素瘤的放疗适应证的描述不正确的是（ ）。
    A. 60岁以上，病变厚度大于1mm者，不适合放疗
    B. 恶性黑色素瘤对放射治疗相对不敏感，通常非第一选择治疗手段
    C. 可用于治疗病变位于面部且病变变厚或年龄小，因大手术可极大影响面容而拒绝手术的患者
    D. 对于头皮部病变可考虑放射治疗
    E. 对不能手术局部晚期、转移或复发的恶性黑色素瘤病变也可行放疗
    **参考答案：A**
    **解析：** 在60岁以上、病变厚度大于1mm的患者，放射治疗可取得与手术相近的疗效。
56. 关于恶性黑色素瘤脑转移的治疗描述不正确的是（ ）。
    A. 外科治疗除解除症状外，还可延长脑部无瘤时间
    B. 激素治疗常用于减轻水肿而减轻症状，达到姑息治疗的目的
    C. 在单个的转移的患者，手术后中位生存期为10个月，先前对免疫治疗反应的患者疗效更好
    D. 如果病变为孤立有症状者，可给予X刀或手术治疗，治疗后无明显神经损伤
    E. 对于多发脑转移者，给予全脑放疗后疗效较好
    **参考答案：E**
    **解析：** 对于多发脑转移者，应给予全脑预防照射，而后根据病情给予局部推量放射治疗，可解除症状和延长生存期，但疗效差。
57. 患者男性，17岁，下肢肿痛，行走不便2月余，X线图像如下，最有可能的诊断是（ ）。
    A. 骨肉瘤
    B. 骨巨细胞瘤
    C. 骨结核
    D. 骨软骨瘤
    E. 骨转移瘤
    **参考答案：A**
    **解析：** 青年男性，可见胫骨上端广泛骨质破坏，骨膜破坏，软组织侵犯，骨肉瘤。骨巨细胞瘤、骨软骨瘤、骨结核和骨转移瘤没有骨膜破坏、软组织侵犯。
58. 患者男性，21岁，右股骨下端骨肿瘤，活检示尤文肉瘤，肿瘤最大径4cm，未发现淋巴结及远处转移，AJCC分期为（ ）。
    A. ⅡB期
    B. Ⅰ期
    C. ⅡA期
    D. ⅠB期
    E. Ⅲ期
    **参考答案：C**
    **解析：** 尤文肉瘤组织学分级为高级，G4，故此患者分期为T1N0M0G4，ⅡA期。
59. 患者女性，65岁，发现肩背部肿块5年，生长缓慢，现约5cm×4cm，质软，边界不清，无压痛，表面皮肤无红肿，无静脉曲张，最有可能的诊断是（ ）。
    A. 皮脂腺囊肿
    B. 平滑肌瘤
    C. 神经纤维瘤
    D. 皮肤癌
    E. 脂肪瘤
    **参考答案：E**
    **解析：** 皮肤癌一般伴有局部皮损，皮脂腺囊肿是由于皮脂腺分泌不畅导致局部皮脂腺瘀积而形成的，一般较小，不会达到5cm左右。平滑肌瘤质地偏中，边界清晰。神经纤维瘤多为多发，质地中等偏硬。患者的临床表现符合脂肪瘤的特点，因此对该患者首先考虑的可能诊断是脂肪瘤。
60. 患者女性，18岁，右大腿下段肿痛2约余，X线片见股骨下段境界不清的骨质破坏区，骨膜增生及放射状阴影，两端可见骨膜三角，最有可能的诊断是（ ）。
    A. 骨巨细胞瘤
    B. 骨髓炎
    C. 骨转移癌
    D. 骨结核
    E. 骨肉瘤
    **参考答案：E**
    **解析：** 骨髓炎在X线片上可见到明显的骨修复，骨膜的新生骨增厚，并同骨皮质融合，呈分层状，外缘呈花边状，在未痊愈的骨髓炎中可以同时看到骨质破坏和死骨。长骨干结核主要变现为骨质局限性破坏，很少骨质增生，可侵及骨皮质，并且可能伴有轻微骨膜增生，死骨少见。骨囊肿的表现为圆形或卵圆形、边界清楚的透明区，有时呈多囊状，但病变内无骨隔，只有横行的骨嵴，囊肿沿长轴发展，骨皮质变为薄层骨壳。骨巨细胞瘤多数在破坏区内可有数量不等、比较纤细的骨嵴，有大小不一的小房征，也有的患者表现为溶骨性改变，肿瘤内无钙化或骨化致密影，无反应性骨增生，如没有骨折也不会伴有骨膜增生。该患者的临床X线表现符合骨肉瘤的典型临床表现。
61. 患者男性，21岁，左大腿下段肿痛3月，X线片示左股骨下端骨肿瘤，骨肉瘤可能性大。活检示骨肉瘤，下一步合适的处理是（ ）。
    A. 大剂量放疗，后行保肢或截肢手术
    B. 立即进行截肢
    C. 保肢或截肢手术，术后化疗
    D. 大剂量放疗后行保肢手术
    E. 化疗后行保肢或截肢手术，术后化疗，考虑术后放疗
    **参考答案：E**
    **解析：** 骨肉瘤的治疗是综合治疗获得明显疗效的典型例证，化疗、放疗及手术的综合治疗提高了保肢率、生存率，对患者生活质量有明显改善。目前术前新辅助化疗、后行保肢或截肢手术，术后病理检查了解局部肿瘤细胞坏死率，术后考虑辅助化疗或放疗等成为骨肉瘤标准的治疗方式。

**共享题干题**

【题干】患者男性，15岁，左大腿疼痛半月，X线提示左股骨中段溶骨性破坏，周围软组织肿胀。活检示尤文肉瘤。

1. 【单项选择题】此病最常见下列哪项远处转移部位？（ ）
   A. 肺、脑
   B. 肝、骨
   C. 肺、骨
   D. 肺、肝
   E. 肝、脑
   **参考答案：C**
2. 【单项选择题】尤文肉瘤通过综合治疗，总体5年生存率达（ ）。
   A. 60%～70%
   B. 30%～40%
   C. 50%～60%
   D. 40%～50%
   E. 70%以上
   **参考答案：E**
   **解析：** 尤文肉瘤分化程度差，恶性程度高，大约四分之一的患者在确诊时已经发现远处转移，最常见的转移部位是肺和骨。所有尤文肉瘤患者的治疗均应遵循以下原则：①G－CSF支持下的多药联合化疗维持12～24周；②局部治疗（包括病灶广泛切除±术前放疗；根治性放疗＋化疗；截肢术）；③辅助化疗±放疗。包括新辅助化疗在内，总体化疗时间应达到36周。
3. 【单项选择题】如病变局限，首选下列哪项治疗方式？（ ）
   A. 新辅助化疗＋广泛性或根治性手术＋辅助化疗
   B. 广泛性或根治性手术
   C. 手术＋术后放疗
   D. 手术＋术后化疗
   E. 化疗＋放疗
   **参考答案：A**

【题干】患者男性，16岁，左小腿上段肿胀疼痛3月余，夜间痛明显，查体：左胫骨上段肿胀明显，有压痛，可扪及约6cm×7cm质硬包块，固定，X片显示左胫骨上段虫蚀状溶骨性破坏，骨膜反应明显，可见Codman三角，局部软组织受侵、肿胀。

1. 【单项选择题】应常规进行的检查是（ ）。
   A. 胸部X线检查
   B. 淋巴管造影
   C. 头颅CT
   D. 胃肠道钡餐检查
   E. 骨髓穿刺
   **参考答案：A**
2. 【单项选择题】临床诊断考虑为（ ）。
   A. 骨巨细胞瘤
   B. 骨髓炎
   C. 骨肉瘤
   D. 骨髓瘤
   E. 骨囊肿
   **参考答案：C**
3. 【单项选择题】最佳治疗方案是（ ）。
   A. 保肢/截肢术，术前化疗，术后放疗
   B. 单纯化疗
   C. 刮除术＋骨水泥填充术，术前后化疗
   D. 单纯放疗
   E. 保肢/截肢术，术前后化疗
   **参考答案：E**
   **解析：** 良性骨肿瘤通常无骨膜反应，无软组织影，本病例根据发病年龄、部位、X线特点考虑骨肉瘤可能性大。骨肉瘤是儿童和青少年最常见的原发性骨恶性肿瘤，中位发病年龄20岁。好发于股骨远端和胫骨近端干骺部。疼痛和肿胀是最常见的早期症状。骨肉瘤通过血道播散，肺是最常见的转移部位，所以胸部X线检查是常规检查。新辅助化疗和辅助化疗是有效的治疗手段，化疗的病理缓解程度是重要的预后指标。化疗药物应该至少包括以下药物之中的两种：多柔比星、顺铂、异环磷酰胺和大剂量甲氨蝶呤。辅助化疗后的放疗推荐用于未能完全切除的高分级的骨肉瘤。

【题干】患者女性，39岁，右股骨上段疼痛1个月。查体：右股骨上段压痛，右髋关节活动受限，X线片，右股骨颈及转子下溶骨性骨破坏。3年前曾患乳腺癌，行乳癌根治术，术后未作辅助治疗。

1. 【单项选择题】最有可能是的诊断是（ ）。
   A. 骨巨细胞瘤
   B. 骨肉瘤
   C. 软骨肉瘤
   D. 骨髓瘤
   E. 乳癌复发（骨转移）
   **参考答案：E**
2. 【单项选择题】了解其他部位的骨骼有无并存的病灶，首选的检查是（ ）。
   A. X线断层摄片
   B. CT
   C. 骨ECT
   D. MRI
   E. 骨髓穿刺
   **参考答案：C**
   **解析：** 骨转移癌的发生率远远超过原发性恶性骨肿瘤的发生率，大约有1/4的恶性肿瘤患者发生骨转移，至少50%的癌症患者尸检后证实骨转移，乳腺癌骨转移发生率为28%～69%。骨转移癌好发于脊柱、骨盆和长骨干骺端，躯干骨多于四肢骨，下肢骨多于上肢骨。本病例患者有乳腺癌病史，未作规范治疗，高度怀疑乳癌复发。

【题干】某女性乳腺癌患者，手术放化疗综合治疗3年后，主诉腰背部疼痛不适2月余，且因为疼痛夜间睡眠较差，但X线平片腰椎检查没有异常发现。

1. 【单项选择题】为尽早判断有无骨转移，应该再进行（ ）。
   A. 碱性磷酸酶水平检测
   B. 骨扫描检查
   C. CT检查
   D. MRI检查
   E. 活检穿刺
   **参考答案：B**
   **解析：** 与MRI、CT及X线平片相比，骨扫描可以较早发现骨转移性病变（可提前3个月左右）。
2. 【单项选择题】该患者2个月后疼痛加重，MRI检查证实腰3、4椎体破坏，应该进行下列哪项姑息性治疗？（ ）
   A. 放射性核素治疗
   B. 姑息性化疗
   C. 内分泌治疗
   D. 姑息性放疗
   E. 分子靶向治疗
   **参考答案：D**
   **解析：** 脊柱出现骨转移时，为了避免出现压缩性骨折引起截瘫等严重并发症，应该行姑息性放疗。此外如果是股骨、髋骨等负重部位的骨转移，也应做类似的处理。如果病理性骨折已经出现，还需要考虑外科手术治疗，以改善患者生活质量。
3. 【单项选择题】为了控制患者疼痛，在初期进行止痛药物剂量滴定时，应改选用下列哪种制剂？（ ）
   A. 曲马朵
   B. 芬太尼透皮贴剂
   C. 即释吗啡片
   D. 长效吗啡控释剂
   E. 度冷丁
   **参考答案：C**
   **解析：** 度冷丁不适用于慢性癌痛患者的止痛治疗。由于即释吗啡片作用时间短，在初期对患者进行止痛药物剂量滴定时，常用即释吗啡片，便于进行剂量调整。

**共享答案题**

【选项】
A. 40～60岁
B. 10岁以下
C. 20～40岁
D. 5～15岁
E. 60岁以上

1. 【单项选择题】尤文肉瘤的好发年龄（ ）。
   **参考答案：D**
2. 【单项选择题】骨巨细胞瘤的好发年龄（ ）。
   **参考答案：C**
3. 【单项选择题】软骨肉瘤的好发年龄（ ）。
   **参考答案：A**

【选项】
A. 术前放疗＋手术
B. 术前化疗＋手术＋术后化疗
C. 手术＋术后化疗
D. 手术
E. 放疗

1. 【单项选择题】尤文肉瘤的主要治疗方式为（ ）。
   **参考答案：B**
2. 【单项选择题】软骨肉瘤的主要治疗方式为（ ）。
   **参考答案：D**
   **解析：** 联合化疗在恶性骨肿瘤综合治疗中的地位目前已得到广泛的公认。骨肉瘤和尤文肉瘤治疗原则相似，术前讲行薪辅助化疗，目的是提高全身控制率和保存肢体，使手术容易进行。术后标本进行病理组织学观察，根据化疗后组织坏死程度分为四级：Ⅰ级，几乎没有肿瘤坏死；Ⅱ级，肿瘤坏死率＜90%；Ⅲ级，肿瘤坏死率＞90%；Ⅳ级，肿瘤全部坏死，未见活的肿瘤细胞。参考坏死程度选择术后化疗方案，Ⅰ、Ⅱ级沿用术前化疗方案，Ⅲ、Ⅳ级更改方案。术前化疗周期数2～6个，术后2～4周期。软骨肉瘤对化疗不敏感，治疗以手术治疗为主，切缘阳性者还要考虑术后放疗。
3. 【单项选择题】骨肉瘤的主要治疗方式为（ ）。
   **参考答案：B**

【选项】
A. 平滑肌肉瘤
B. 恶性纤维组织细胞瘤
C. 滑膜肉瘤
D. 横纹肌肉瘤
E. 脂肪肉瘤

1. 【单项选择题】手足附近最常见的软组织肉瘤是（ ）。
   **参考答案：C**
2. 【单项选择题】最常见于腹腔和腹膜后区的软组织肉瘤是（ ）。
   **参考答案：A**
3. 【单项选择题】下肢的病变得到控制后，仍有30%～50%的几率在腹膜后出现第二个病变，这种软组织肉瘤是（ ）。
   **参考答案：E**
