# Supplementary material for: Knowledge localization is associated with higher performance of domestic large language models in a Chinese radiation oncology examination
Source: Front Oncol. 2026 Jun 17;16:1808714. doi: 10.3389/fonc.2026.1808714 (PMC13318762; doi:10.3389/fonc.2026.1808714)
Supplement: Supplementary file 2 [file Table2.docx]

# Chapter 1: Head and Neck Tumors (N=178)

## Standard Multiple-Choice Questions

1. In China, the incidence of malignant head and neck tumors in males ranks first in:

A. Thyroid cancer

B. Nasopharyngeal carcinoma

C. Oral cancer

D. Laryngeal cancer

E. Maxillary sinus cancer

1. In China, the incidence of malignant head and neck tumors in females ranks first in:

A. Thyroid cancer

B. Nasopharyngeal carcinoma

C. Oral cancer

D. Laryngeal cancer

E. Maxillary sinus cancer

1. Commonly referred to head and neck tumors exclude:

A. Thyroid cancer

B. Nasopharyngeal carcinoma

C. Brain glioma

D. Oral cancer

E. Squamous cell carcinoma of the external auditory canal

1. In China, the number of head and neck malignant tumor cases accounts for approximately what percentage of all systemic malignant tumors?

A. 15%–20%

B. 1%–5%

C. 10%–15%

D. 5%–10%

E. More than 20%

1. In the United States, the number of head and neck malignant tumor cases accounts for approximately what percentage of all systemic malignant tumors?

A. 15%–20%

B. Less than 5%

C. 10%–15%

D. 5%–10%

E. More than 20%

1. The most common pathological type of head and neck malignant tumors is:

A. Lymphoma

B. Squamous cell carcinoma

C. Undifferentiated carcinoma

D. Adenocarcinoma

E. Soft tissue sarcoma

1. If a head and neck malignant tumor undergoes preoperative radiotherapy first, the optimal timing for surgery after the end of radiotherapy is:

A. 4–6 weeks after the end of radiotherapy

B. Within 1 week after the end of radiotherapy

C. 2–4 weeks after the end of radiotherapy

D. 1–2 weeks after the end of radiotherapy

E. 6–8 weeks after the end of radiotherapy

1. Among the following head and neck malignant tumors, the one with the poorest treatment outcome is:

A. Nasopharyngeal carcinoma

B. Thyroid cancer

C. Laryngeal cancer

D. Parotid cancer

E. Hypopharyngeal cancer

1. The overall cure rate for head and neck malignant tumors is in the range of:

A. 40%–70%

B. 10%–20%

C. 30%–40%

D. 20%–30%

E. 70%–100%

1. Which of the following statements regarding the treatment and prognosis of head and neck tumors is correct?

A. Concurrent chemoradiotherapy cannot be used for preoperative treatment of head and neck tumors

B. Most head and neck tumors have superficial lesions, which is conducive to early detection and diagnosis, so the cure rate is relatively high

C. Cervical lymph node metastasis does not affect the patient's survival rate

D. Hypopharyngeal cancer has better treatment outcomes, while parotid cancer and laryngeal cancer have poorer outcomes

E. Preoperative radiotherapy dose for head and neck tumors is 60–70 Gy

1. Which of the following chemotherapy drugs does not belong to the commonly used drugs for head and neck tumors?

A. Docetaxel

B. Cisplatin

C. Paclitaxel

D. Fluorouracil

E. Irinotecan

1. Regarding the application of concurrent chemoradiotherapy in the treatment of head and neck tumors, the incorrect description is:

A. Concurrent chemoradiotherapy has severe adverse reactions and patients cannot tolerate it

B. The application of concurrent chemoradiotherapy can improve the local control rate of head and neck tumors

C. Drugs currently commonly used for concurrent chemoradiotherapy of head and neck tumors include cisplatin, fluorouracil, paclitaxel, etc.

D. Chemotherapy drugs have a radiosensitizing effect

E. Concurrent chemoradiotherapy can also be performed before surgery

1. Which of the following belongs to the optimal treatment mode for malignant tumors of the nasal cavity and paranasal sinuses?

A. Radiotherapy combined with chemotherapy

B. Surgery alone

C. Radiotherapy combined with surgery

D. Radiotherapy alone

E. Surgery combined with chemotherapy

1. Regarding the treatment of malignant tumors of the nasal cavity and paranasal sinuses, which of the following statements is incorrect?

A. Malignant melanoma should adopt a comprehensive treatment mode combining radiotherapy, surgery, chemotherapy, and biotherapy

B. Undifferentiated carcinoma of the maxillary sinus only requires simple radiotherapy

C. Clinical Stage A esthesioneuroblastoma can be treated with simple surgery or simple radiotherapy

D. If the roof of the maxillary sinus and orbital soft tissue are invaded, the orbital contents can be preserved, and postoperative radiotherapy can be supplemented

E. Radical neck lymph node dissection should be performed when there is cervical lymph node metastasis

1. The most common pathological type of malignant tumors of the jaw is:

A. Fibrosarcoma

B. Osteosarcoma

C. Malignant fibrous histiocytoma

D. Chondrosarcoma

E. Lymphoma

1. The most common tissue origin of malignant tumors of the jaw is:

A. Non-odontogenic

B. Metastatic cancer

C. Odontogenic

D. Neurogenic

E. Soft tissue origin

1. Which of the following statements regarding malignant salivary gland tumors is incorrect?

A. The natural course is generally short

B. Malignant parotid tumors are the most common

C. Malignant sublingual gland tumors are the least common

D. Minor salivary gland cancer occurs frequently in the palate

E. Pathological types are complex and diverse

1. The most common clinical manifestation of malignant parotid tumors is:

A. Difficulty opening the mouth

B. Facial nerve paralysis

C. Hoarseness

D. Pre-auricular mass

E. Facial numbness

1. The most common clinical manifestation of malignant submandibular gland tumors is:

A. Skin ulceration

B. Submandibular mass

C. Local pain

D. Ipsilateral tongue numbness

E. Restricted mouth opening

1. The most common pathological type of submandibular gland and minor salivary gland carcinomas is:

A. Adenoid cystic carcinoma

B. Undifferentiated carcinoma

C. Mucoepidermoid carcinoma

D. Squamous cell carcinoma

E. Lymphoma

1. In which of the following situations should total parotidectomy be performed?

A. Squamous cell carcinoma

B. Poorly differentiated adenocarcinoma

C. Undifferentiated carcinoma

D. Malignant mixed tumor

E. All of the above

1. Which of the following principles regarding neck lymph node management for malignant parotid tumors is incorrect?

A. Lymph node dissection is generally not performed for adenoid cystic carcinoma

B. Parotid cancer with cervical lymph node metastasis should undergo neck lymph node dissection

C. Lymph node dissection is not performed for squamous cell carcinoma and poorly differentiated mucoepidermoid carcinoma without lymph node metastasis

D. Clinical N0 undifferentiated carcinoma and poorly differentiated adenocarcinoma can undergo elective lymph node dissection

E. Malignant mixed tumors accompanied by facial nerve paralysis should undergo elective lymph node dissection

1. Indications for postoperative radiotherapy for malignant parotid tumors exclude:

A. Well-differentiated mucoepidermoid carcinoma

B. Positive pathological margins or macroscopic residue

C. Accompanied by lymph node metastasis

D. Accompanied by facial nerve paralysis

E. Recurrent patients

1. Regarding the surgical principles for malignant parotid tumors, which is incorrect?

A. If the tumor invades surrounding tissues, the invaded and adjacent tissues should be resected

B. For adenoid cystic carcinoma that has already metastasized to the lung, resection of the primary lesion is meaningless

C. For acinic cell carcinoma located in the superficial lobe of the parotid gland, smaller, and without external invasion, superficial parotidectomy preserving the facial nerve can be performed

D. For deep lobe parotid cancer without facial nerve invasion, total parotidectomy preserving the facial nerve can be performed

E. Adenoid cystic carcinoma with nerve invasion should be resected until the margin is negative

1. Which of the following does not belong to the clinical manifestations of ethmoid sinus cancer?

A. Cranio-orbital pain

B. Facial numbness

C. Diplopia

D. Nasal congestion

E. Eyeball displacement

1. The main diagnostic means for malignant tumors of the nasal cavity and paranasal sinuses exclude:

A. Biopsy

B. Magnetic resonance imaging (MRI)

C. Bone ECT

D. CT scan

E. Immunohistochemistry

1. Which of the following does not belong to the functions of preoperative radiotherapy in the treatment of malignant tumors of the nasal cavity and paranasal sinuses?

A. Reduce bleeding

B. Improve local control rate

C. Eliminate subclinical lesions

D. Shrink the tumor

E. Reduce intraoperative dissemination

1. Which of the following descriptions of the anatomical structure of the larynx is inappropriate?

A. Mainly composed of bone, mucous membrane, and muscle

B. Anatomically divided into three regions: supraglottic region, glottic region, and subglottic region

C. Located in the center of the anterior neck, corresponding to the level of the 4th to 6th cervical vertebrae in adults

D. Includes two spaces: pre-styloid space and paraglottic space

E. It continues with the oropharynx above and communicates with the trachea below

1. Regarding the anatomical characteristics of the oropharynx, the following statement is correct:

A. The oropharynx does not include the posterior 1/3 of the tongue

B. The oropharynx is between the level of the hard palate and the hyoid bone

C. The anterior wall of the oropharynx is the tongue-epiglottis area, including the tongue base and vallecula; the roof is the soft palate and uvula; the posterior wall is the anterior margin of the cervical vertebrae; the lateral walls include the tonsils, tonsillar fossa, faucial pillars, and glossotonsillar sulcus

D. The upper boundary of the oropharynx connects with the nasopharynx, and the lower boundary reaches the vallecula adjoining the larynx

E. There is very little lymphoid tissue in the oropharynx, so tumors in this area rarely show lymph node metastasis regardless of early or late stage

1. Which of the following regarding the anatomy of the larynx is incorrect?

A. The pre-epiglottic space is shaped like an inverted cone, wide at the top and narrow at the bottom, located anterior to the epiglottis

B. Both the suprahyoid epiglottis and infrahyoid epiglottis belong to the supraglottic region

C. The subglottic region refers to the area from below the glottic region to the level of the lower border of the cricoid cartilage, about 2cm long

D. The glottic region includes the area between the vocal cords, the anterior commissure, and the posterior commissure

E. The paraglottic space is located between the inner perichondrium of the thyroid cartilage and the thyroarytenoid muscle

1. Which of the following descriptions regarding the lymphatic distribution of the larynx is correct?

A. The superficial lymphatic vessels in the larynx have septa, and the supraglottic and subglottic regions do not communicate

B. The lymphatic vessels in the supraglottic region are thin and sparse, distributed in a single layer

C. The superficial lymphatic vessels in the larynx have septa, and the left and right larynx do not communicate

D. The lymphatic vessels in the subglottic region are thick and dense, distributed in multiple layers

E. The blood vessels and lymphatic vessels in the subglottic cricoid part communicate circumferentially

1. The most common neck lymph node metastasis regions for oropharyngeal tumors are:

A. Levels I~VI

B. Level I and Level II

C. Level IV and Level V

D. Level II and Level III

E. Levels I~V

1. Regarding the lymphatic drainage of the larynx, the following statement is correct:

A. The true vocal cords basically have no capillary lymphatic vessels

B. The lymphatic drainage of the supraglottic and subglottic regions drains to the same lymph node groups bounded by the vocal cords

C. The lymphatic vessels in the subglottic region are rich, mainly draining to the pre-laryngeal, pre-tracheal, and paratracheal lymph nodes

D. The lymphatic vessels in the supraglottic region are small, collecting and draining to the upper or middle deep cervical lymph nodes

E. Early vocal cord cancer is prone to lymph node metastasis

1. Which of the following descriptions regarding laryngeal cancer is incorrect?

A. More than 90% of laryngeal cancers can be detected by indirect laryngoscopy

B. The pathological type of laryngeal cancer is mostly squamous cell carcinoma

C. Cervical lymph node metastasis in glottic cancer is common and appears early

D. Supraglottic cancer is more common in women than in men

E. The most common distant metastasis of laryngeal cancer is to the lung

1. The common invasion pathway of supraglottic laryngeal cancer is:

A. Via the paraglottic space to the paraglottic space and developing into the glottic region

B. Via the paraglottic space to the pre-glottic space and then invading the glottic region

C. Via the supraglottic space to the pre-glottic space and developing into the glottic region

D. Via the supraglottic space to the paraglottic space and finally invading the glottic region

E. Via the pre-epiglottic space to the paraglottic space and developing into the glottic region

1. Regarding the description of glottic cancer, which is correct?

A. Tumors mostly occur in the posterior 1/3 of the vocal cords

B. It ranks second in the incidence of laryngeal cancer, accounting for about 40%

C. The pathological type is mostly poorly differentiated squamous cell carcinoma

D. Compared with supraglottic cancer, cervical lymph node metastasis is more common

E. The main clinical manifestation is hoarseness

1. Regarding oral cancer, the following statement is correct:

A. The anatomical structures included in oral cancer are: upper and lower gingiva, hard palate, floor of mouth, buccal mucosa (five parts)

B. The further the site of oral tumor is from the lip and closer to the oropharynx, the better the tumor differentiation

C. Oral cancer is incurable

D. The pathological type of oral tumors is mainly adenocarcinoma

E. Simple high-energy X-ray irradiation and high-energy X-ray plus interstitial brachytherapy are the most commonly used treatment techniques in radiotherapy for oral cancer

1. The following description regarding lip cancer is correct:

A. Upper lip cancer is mainly transitional cell carcinoma

B. It occurs frequently in black people and is rare in light-skinned races

C. Most lower lip cancers are moderately differentiated or well-differentiated squamous cell carcinomas

D. About 90% of lip cancers occur on the upper lip

E. Primary skin cancer invading the lip should also be diagnosed as lip cancer

1. Which of the following statements regarding tonsillar cancer is correct?

A. In the initial stage of treatment for late-stage tonsillar cancer, unilateral tonsillar region irradiation technique is generally adopted

B. More than 95% of tumors originating from the tonsil are squamous cell carcinoma and sarcoma

C. The common symptom of tonsillar cancer is bilateral throat pain combined with hoarseness, which can radiate to the root of the ear and is aggravated when eating or drinking

D. Tonsillar cancer is mostly poorly differentiated, with the pathological type mainly being poorly differentiated or undifferentiated carcinoma, and is prone to spread to adjacent structures

E. Tonsillar cancer has a low rate of lymph node metastasis, so the lower neck and supraclavicular regions are not routinely irradiated prophylactically

1. Considering treatment efficacy and preservation of organ function, the preferred treatment method for early-stage head and neck tumors is:

A. Biotherapy

B. Surgical treatment

C. Chemotherapy

D. Radiotherapy

E. Hyperthermia

1. The reasonable treatment method for early glottic laryngeal cancer (T1N0M0) is:

A. Preoperative radiotherapy + surgical resection + postoperative concurrent chemoradiotherapy

B. Radical radiotherapy

C. Total laryngectomy + radical radiotherapy

D. Vocal cord resection only

E. Surgery + postoperative chemotherapy

1. For tonsillar cancer cT2N1M0 undergoing conventional radical radiotherapy, the correct design of the radiation field is:

A. Bilateral tonsillar region face-neck field

B. Ipsilateral tonsillar region small field

C. Ipsilateral tonsillar region face-neck field + middle/lower neck and supraclavicular field

D. Ipsilateral tonsillar region face-neck field

E. Bilateral tonsillar region face-neck field + bilateral middle/lower neck and supraclavicular field

1. At the beginning of radiotherapy for head and neck tumors, obvious parotid swelling and pain accompanied by low-grade fever appear, and blood tests do not suggest infection. The reasonable management method is:

A. Apply hot compress treatment only

B. Only simple antipyretic treatment is needed

C. Hold Vitamin C in mouth only

D. Keep the mouth as clean as possible, use mouthwash treatment, add antibiotics if necessary, and pause radiotherapy

E. Terminate radiotherapy and give systemic anti-inflammatory treatment

1. For well-differentiated squamous cell carcinoma of the buccal mucosa cT2N0M0, if radiotherapy is performed, the correct management of the neck lymphatic drainage area is:

A. Only need to prophylactically irradiate the ipsilateral submandibular, upper deep cervical, digastric, and submental lymph node regions

B. Neck lymph node regions do not need prophylactic irradiation, observe regularly

C. Prophylactic irradiation of the neck, followed by lymph node dissection after radiotherapy

D. Prophylactic irradiation of the neck

E. Need to prophylactically irradiate bilateral submandibular, upper deep cervical, digastric, and submental lymph node regions

1. In the 2002 UICC staging of oral cancer, the diagnostic criterion for N3 is:

A. Bilateral cervical lymph node metastasis <3cm but fixed

B. Contralateral cervical lymph node metastasis but <3cm

C. Ipsilateral cervical lymph node metastasis >3cm but not exceeding 6cm

D. Bilateral cervical lymph node metastasis but <3cm

E. Cervical lymph node metastasis >6cm

1. The radiation source selection for conventional external beam radiotherapy of early squamous cell carcinoma of the mobile tongue (cT1N0M0) primary lesion is:

A. Electron beam

B. Low energy X-ray + Electron beam

C. Fast neutron

D. High energy X-ray + Electron beam

E. High energy X-ray + Fast neutron

1. Regarding the surgical treatment principles of laryngeal cancer, which of the following statements is incorrect?

A. Generally, neck lymph node dissection is required, especially for those with cervical lymph node metastasis

B. Locally advanced patients (cT3~4N1~3) can undergo total laryngectomy or preserve laryngeal function according to the situation after preoperative radiotherapy

C. When accompanied by severe laryngeal obstruction, surgical resection can be performed first, and radiotherapy or other treatments can be considered after surgery

D. Patients with local recurrence within a short period after radical radiotherapy can undergo salvage surgery

E. Early glottic well-differentiated squamous cell carcinoma (cT1~2) requires total laryngectomy + neck lymph node dissection + postoperative adjuvant radiotherapy

1. Which of the following statements regarding the principles of radiotherapy for laryngeal cancer is incorrect?

A. For patients with lesions invading the subglottic region undergoing postoperative radiotherapy, the tracheostoma should be avoided

B. Early supraglottic poorly differentiated carcinoma or undifferentiated carcinoma can choose radiotherapy as the first choice

C. Patients with cartilage invasion but no lymph node metastasis still need postoperative radiotherapy after surgical resection

D. T3 and T4 laryngeal cancer requires postoperative radiotherapy after surgical resection

E. Patients with lesions severely obstructing the airway accompanied by dyspnea are relative contraindications for radiotherapy

1. Which of the following descriptions regarding conventional radiotherapy for laryngeal cancer is correct?

A. Second primary tumor is a common acute phase reaction of radiotherapy

B. For glottic cancer radiotherapy, 60Co or 4MV linear accelerator is the first choice

C. The fractional dose of radiotherapy should be lower than 2Gy, i.e., 1.8Gy/fraction

D. Laryngeal cancer usually adopts split-course irradiation to reduce upper respiratory tract and upper digestive tract complications

E. The choice of ray energy for supraglottic laryngeal cancer has a greater impact on efficacy

1. Which of the following statements regarding the indications for radiotherapy of lip cancer is incorrect?

A. Patients with local recurrence after surgery who cannot or are unwilling to undergo surgical treatment again can undergo radiotherapy

B. Superficial lesions occupying only 1/3 of the lower lip or T1 lesions, hoping to obtain better cosmetic results

C. For infiltrative lesions (e.g., infiltration depth exceeding 3cm) where surgical treatment is difficult to obtain satisfactory functional and cosmetic results, radiotherapy can be performed first

D. Lesions involving the commissure or simultaneously involving the upper and lower lips can choose radiotherapy as the first choice

E. Early lip cancer (cT1~2N0M0) requires routine supplementary local radiotherapy after surgery

1. Which of the following is incorrect regarding the management of complications of radiotherapy for laryngeal cancer?

A. If laryngeal edema persists for 3 months after radical radiotherapy accompanied by vocal cord fixation, the possibility of uncontrolled tumor should be considered; once confirmed, salvage laryngectomy can be performed

B. When mild laryngeal edema occurs, ultrasonic nebulization treatment should be given in time

C. Avoiding excessive voice use during radiotherapy can reduce acute radiation reactions

D. For laryngeal cartilage necrosis, anti-inflammatory conservative treatment should be the first choice

E. Quitting smoking before or during radiotherapy can reduce the occurrence of complications

1. Factors affecting the local control rate of radiotherapy for laryngeal cancer to be excluded are:

A. Duration of symptoms

B. KPS score status

C. High or low Hb (hemoglobin) before treatment

D. Second primary tumor combined after radiotherapy

E. Size and growth pattern of the tumor

1. Which of the following is correct regarding the target volume delineation design principles for late-stage laryngeal cancer?

A. Target volume delineation for cT3~4N0 glottic cancer only needs to include bilateral level I~II cervical lymph nodes

B. The CTV of the primary tumor is the GTV delineated by imaging plus a 3mm margin

C. The CTV of the lymphatic drainage area for N0 supraglottic cancer includes bilateral upper and middle cervical lymph nodes

D. The PTV is the CTV of the primary tumor plus a 2cm margin

E. The CTV of the lymphatic drainage area for cT3~4N2c supraglottic lesions includes bilateral level II~V cervical lymph nodes + retropharyngeal lymph node region

1. Which of the following is correct regarding the radiotherapy principles for soft palate cancer?

A. Soft palate cancer of minor salivary gland origin has high radiosensitivity, and the radical dose of radiotherapy is DT 60Gy

B. Well-differentiated squamous cell carcinoma without upper neck lymph node metastasis requires prophylactic irradiation of the middle and lower neck lymph nodes

C. For poorly differentiated or undifferentiated carcinoma, regardless of whether there is lymph node metastasis in the upper neck, prophylactic irradiation is required for the bilateral middle and lower neck and supraclavicular regions

D. For well-differentiated squamous cell carcinoma with unilateral upper neck lymph node metastasis, prophylactic irradiation is required for the contralateral middle and lower neck regions

E. Soft palate cancer is conventionally irradiated with large fields to DT 36Gy then avoids the spinal cord, and at DT 40Gy the field is shrunk to the soft palate area

1. Which of the following is incorrect regarding the conventional radiotherapy design for base of tongue cancer?

A. The isocenter position of the primary lesion horizontal field is conventionally located at the intersection of the middle-posterior 1/3 of the tongue and the body midline

B. Generally, the primary lesion and upper neck isocenter level use opposed fields, and the lower neck and supraclavicular fields use anterior tangential field irradiation technique

C. The posterior border of the primary lesion should reach the anterior margin of the vertebral body, avoiding the jugular chain lymph node area

D. The superior border of the primary lesion is 1.5~2cm above the surface of the tongue base when the mouth bite block presses the tongue to the floor of the mouth; if the tumor invades the nasopharynx, the superior border should be raised accordingly

E. The target area included in the isocenter horizontal irradiation field is the primary lesion, bilateral retropharyngeal lymph nodes, upper deep cervical lymph nodes, subdigastric lymph nodes, and posterior cervical lymph nodes

1. Which of the following statements regarding conventional radiotherapy for gingival cancer is incorrect?

A. The radiation field for upper gingival cancer should include part of the maxillary sinus in addition to the primary lesion

B. Radical radiotherapy is the first choice for the treatment of early gingival cancer

C. The radiation field for lower gingival cancer should include the ipsilateral mandible

D. The conventional radiotherapy method uses ipsilateral orthogonal wedge field irradiation

E. The anterior border of the conventional radiation field should reach the anterior margin of the mandible and avoid the upper and lower lips as much as possible

1. Anatomically, the parapharyngeal space of the nasopharynx can be divided into:

A. 5 spaces

B. 2 spaces

C. 4 spaces

D. 3 spaces

E. 6 spaces

1. How many walls constitute the nasopharyngeal cavity?

A. 6 walls

B. 3 walls

C. 5 walls

D. 4 walls

E. 7 walls

1. The anatomical location of the hypopharynx corresponds to the plane between which two cervical vertebrae?

A. C4 to C7

B. C1 to C4

C. C3 to C6

D. C2 to C5

E. C5 to C1

1. The nasopharyngeal cavity is located at:

A. Posterior 1/3 of the nasal cavity

B. Upper pharynx

C. Lower pharynx

D. Middle pharynx

E. Between the upper pharynx and middle pharynx

1. Which pairs of cranial nerves pass through the cavernous sinus and its surroundings?

A. 4, 5, 6, 7

B. 1, 2, 3, 4

C. 3, 4, 5, 6

D. 2, 3, 4, 5

E. 5, 6, 7, 8

1. Among the 12 pairs of cranial nerves, the one with the longest course in the middle cranial fossa is:

A. Abducens nerve

B. Optic nerve

C. Trigeminal nerve

D. Oculomotor nerve

E. Facial nerve

1. The cranial nerve passing through the pre-styloid space of the nasopharynx is:

A. V3 cranial nerve

B. II, III cranial nerves

C. IV, V cranial nerves

D. III, IV cranial nerves

E. VI cranial nerve

1. The sentinel lymph nodes of nasopharyngeal carcinoma are generally considered to be composed of which two groups of lymph nodes?

A. Upper posterior cervical and upper deep cervical lymph nodes

B. Upper deep cervical and upper posterior cervical lymph nodes

C. Retropharyngeal and upper deep cervical lymph nodes

D. Upper deep cervical and subdigastric lymph nodes

E. Retropharyngeal and upper posterior cervical lymph nodes

1. Which of the following is a predilection site for nasopharyngeal carcinoma?

A. Torus tubarius

B. Roof of nasopharynx

C. Posterior wall of laryngeal nasopharynx

D. Pharyngeal recess (Fossa of Rosenmuller)

E. Posterior margin of vomer

1. Well-differentiated squamous cell carcinoma accounts for approximately what percentage of the total number of nasopharyngeal carcinomas?

A. 70%–80%

B. Less than 10%

C. 50%–65%

D. 30%–45%

E. 85%–90%

1. The anatomical position of the larynx is between which two vertebral body planes?

A. C4 to C6

B. C1 to C3

C. C3 to C5

D. C2 to C4

E. C5 to C7

1. The lymphatic drainage of the maxillary sinus mainly goes to:

A. Submental lymph nodes

B. Upper deep cervical lymph nodes

C. Submandibular lymph nodes

D. Posterior digastric group lymph nodes

E. Upper deep cervical + submandibular + submental lymph nodes

1. Which pathological type is most common in nasal cavity and paranasal sinus cancers?

A. Sarcoma

B. Squamous cell carcinoma

C. Lymphoma

D. Adenocarcinoma

E. Transitional cell carcinoma

1. The most common site among malignant tumors of the nasal cavity and paranasal sinuses is:

A. Sphenoid sinus cancer

B. External nose cancer

C. Maxillary sinus cancer

D. Nasal cavity cancer

E. Frontal sinus cancer

1. Which pathological type of thyroid cancer has the highest degree of malignancy?

A. Medullary carcinoma

B. Papillary carcinoma

C. Undifferentiated carcinoma (Anaplastic)

D. Follicular adenocarcinoma

E. None of the above answers are correct

1. Factors not affecting the prognosis of maxillary sinus cancer include:

A. Treatment technique

B. Presence or absence of lymph node metastasis

C. Pathological type

D. Involvement of each wall

E. Patient's gender

1. In which region of China does nasopharyngeal carcinoma frequently occur?

A. Northwest region

B. East China region

C. North China region

D. South China region

E. Northeast region

1. Which of the following belongs to the most common initial physical sign of nasopharyngeal carcinoma?

A. Shallowing of nasolabial fold

B. Nasopharyngeal mass

C. Eye movement disorder

D. Horner's sign

E. Neck mass

1. Symptoms of nasopharyngeal carcinoma do not include:

A. Headache

B. Nasal congestion

C. Tinnitus

D. Vertigo

E. Facial numbness

1. Difficulty in opening the mouth (trismus) in patients with nasopharyngeal carcinoma indicates that the tumor has most likely invaded:

A. Medial pterygoid muscle, lateral pterygoid muscle

B. Digastric muscle

C. Tensor veli palatini, levator veli palatini

D. Temporalis muscle

E. Sternocleidomastoid muscle

1. Hypoglossal nerve paralysis in a patient with nasopharyngeal carcinoma indicates that the tumor has invaded:

A. Retropharyngeal space

B. Nasal cavity

C. Post-styloid space

D. Oropharynx

E. Anterior cranial fossa

1. When nasopharyngeal carcinoma causes destruction of the skull base bone and invades the cavernous sinus, the cranial nerve most easily affected is:

A. Oculomotor nerve

B. Abducens nerve

C. Trochlear nerve

D. Trigeminal nerve

E. Optic nerve

1. In nasopharyngeal carcinoma, paralysis of cranial nerves III, IV, VI and the first branch of V on one side is called:

A. Petrosphenoidal syndrome

B. Orbital apex syndrome

C. Pituitary-sphenoid syndrome

D. Superior orbital fissure syndrome

E. Jugular foramen syndrome

1. Soft palate paralysis in a patient with nasopharyngeal carcinoma indicates that the tumor has most likely invaded which nerve?

A. Medial pterygoid muscle, lateral pterygoid muscle

B. Hypoglossal nerve

C. Facial nerve

D. Accessory nerve

E. Tensor veli palatini, levator veli palatini

1. The probability of upper neck lymph node metastasis at the time of diagnosis of nasopharyngeal carcinoma is approximately:

A. 60%–80%

B. Around 10%

C. Around 40%

D. Around 30%

E. 100%

1. Facial numbness in nasopharyngeal carcinoma usually indicates:

A. Trigeminal nerve invasion

B. Nasal cavity invasion

C. Orbit invasion

D. Ethmoid sinus invasion

E. Intracranial invasion

1. Clinical appearance of Horner's sign in a patient with nasopharyngeal carcinoma is due to paralysis of which nerve?

A. XI

B. IX

C. Cervical sympathetic nerve

D. X

E. XII

1. The most common site of cervical lymph node metastasis in nasopharyngeal carcinoma is:

A. Submandibular lymph nodes

B. Posterior cervical lymph nodes

C. Retropharyngeal lymph nodes

D. Upper deep cervical lymph nodes

E. Pre-auricular lymph nodes

1. Which of the following is the possible cause of sudden paroxysmal syncope in a patient with nasopharyngeal carcinoma?

A. Submandibular lymph node metastasis

B. Upper posterior cervical lymph node metastasis

C. Upper deep cervical lymph node metastasis

D. Middle posterior cervical lymph node metastasis

E. Subdigastric lymph node metastasis

1. Manifestations of maxillary sinus tumor invading the posterior wall include:

A. Eye distension and pain, diplopia, etc.

B. Bloody nasal discharge, nasal congestion, etc.

C. Facial pain, etc.

D. Toothache, loosening of teeth, etc.

E. Temporal pain, trismus (difficulty opening mouth), etc.

1. Which of the following examinations is necessary for the confirmation of nasopharyngeal carcinoma?

A. Nasopharyngeal biopsy

B. Nasopharyngoscopy

C. Fine needle aspiration cytology of neck mass

D. CT scan

E. EB virus serology

1. Which of the following is the indispensable and most basic examination for diagnosing nasopharyngeal carcinoma?

A. EB virus serology

B. CT

C. Nasopharyngoscopy plus biopsy

D. MRI

E. Plain X-ray examination

1. The preferred treatment for nasopharyngeal carcinoma without distant organ metastasis is:

A. Traditional Chinese Medicine treatment

B. Surgical treatment

C. Radiotherapy

D. Chemotherapy

E. Chemotherapy + Surgery

1. Which of the following is the main reason for the failure of radiotherapy for maxillary sinus cancer?

A. Local uncontrol or local recurrence

B. Local recurrence + lymph node metastasis

C. Lymph node metastasis + distant metastasis

D. Local recurrence + distant metastasis

E. Local uncontrol + distant metastasis

1. For nasopharyngeal carcinoma with clinical stage T2~4N+M0, the better method for field arrangement in the first phase of conventional radiotherapy is:

A. Anterior facial "Pin" character field (anterior + two lateral fields) + retroauricular field

B. Pre-auricular field + whole neck tangential field

C. Facio-cervical combined field + bilateral lower neck and supraclavicular tangential fields

D. Pre-auricular field + anterior facial "Pin" character field + whole neck tangential field

E. Anterior facial "Pin" character field + retroauricular field + whole neck tangential field

1. Nasopharyngeal carcinoma is mostly poorly differentiated carcinoma. The selected dose for conventional fractionation radiotherapy is:

A. 2.5 Gy/fraction

B. 1.5 Gy/fraction

C. 2.0 Gy/fraction

D. 1.7 Gy/fraction

E. 3.0 Gy/fraction

1. The cause of transient lightning sensation in the waist, sacrum, and lower limbs when lowering the head after radiotherapy for nasopharyngeal carcinoma is:

A. Early radiation pituitary reaction

B. Early radiation temporal lobe reaction

C. Early radiation cervical spinal cord reaction

D. Early radiation cerebellar reaction

E. Radiation vertebral decalcification

1. Indications for brachytherapy (intracavitary treatment) of nasopharyngeal carcinoma include:

A. Oropharyngeal invasion

B. Lesions confined to the nasopharyngeal wall, superficial lesions

C. Obvious invasion of the parapharyngeal space

D. Nasal cavity invasion

E. Skull base bone destruction

1. In Intensity-Modulated Radiation Therapy (IMRT) for nasopharyngeal carcinoma, CTV1 is generally defined as:

A. Primary tumor area

B. Lymph node drainage area

C. High-risk area

D. Low-risk area

E. Planning target volume

1. When high dose rate (HDR) intracavitary brachytherapy is used in synergy with external beam radiotherapy for nasopharyngeal carcinoma, it should be:

A. When external beam radiotherapy reaches DT 50–60 Gy, perform brachytherapy boost for residual superficial, small lesions

B. Performed simultaneously with external beam radiotherapy

C. Add brachytherapy after external beam radiotherapy DT 30 Gy

D. Add brachytherapy after external beam radiotherapy DT 10 Gy

E. Add high-dose brachytherapy after external beam radiotherapy reaches radical dose or above

1. Indications for surgery of the primary lesion of nasopharyngeal carcinoma include:

A. Accompanied by intracranial cavernous sinus invasion

B. Early nasopharyngeal carcinoma

C. Accompanied by sphenoid or ethmoid sinus invasion

D. Accompanied by nasal cavity or maxillary sinus invasion

E. Residual or recurrent nasopharyngeal cavity tumor after radiotherapy, without skull base destruction or post-styloid invasion

1. For a nasopharyngeal carcinoma extending to the oropharynx with metastatic lymph nodes in the upper neck, the best initial radiation field is:

A. Bilateral pre-auricular fields + anterior facial "Pin" character field + neck tangential field

B. Bilateral pre-auricular fields + bilateral neck vertical fields

C. Facio-cervical combined field + lower neck and supraclavicular tangential fields

D. Bilateral pre-auricular fields + neck tangential field

E. Bilateral pre-auricular fields + bilateral retroauricular fields + neck tangential field

1. The radical dose for the primary lesion of nasopharyngeal carcinoma in radiotherapy should generally be:

A. 70 Gy

B. 30 Gy

C. 50 Gy

D. 40 Gy

E. 80 Gy

1. The treatment for metastatic cervical lymph nodes of nasopharyngeal carcinoma should be:

A. High dose rate interstitial brachytherapy

B. Simple high-energy ray external irradiation

C. Simple low-energy ray external irradiation

D. Combination of high-energy X-rays and low-energy X-rays or electron beams

E. Fast neutron irradiation

1. When positioning the pre-auricular field for nasopharyngeal carcinoma, which of the following parts does not need lead shielding?

A. Skull base

B. Eyes

C. Brainstem

D. Larynx

E. Spinal cord

1. The most common complication of radical radiotherapy for nasopharyngeal carcinoma is:

A. Lower cranial nerve injury

B. Skin pigmentation

C. Hearing loss

D. Dry mouth (Xerostomia)

E. Excessive nasal discharge

1. The optimal treatment mode for clinical stage IIA pleomorphic T-cell non-Hodgkin lymphoma of the nasopharynx is:

A. Immunotherapy

B. Surgery

C. Simple chemotherapy

D. Comprehensive treatment with chemoradiotherapy

E. Gene therapy

1. In recent years, the treatment principle usually adopted for maxillary sinus cancer is:

A. Surgery + Chemotherapy

B. Surgery + Radiotherapy

C. Chemotherapy + Surgery

D. Radiotherapy + Surgery

E. Arterial chemotherapy + Fenestration and curettage of tumor

1. A more reasonable method for field arrangement for localized maxillary sinus cancer is:

A. Bilateral opposed fields plus ipsilateral anterior facial field

B. Single ipsilateral anterior facial field

C. Ipsilateral anterior facial and lateral fields (two wedged fields at an angle)

D. Single ipsilateral lateral field

E. Bilateral opposed fields

1. The preoperative radiotherapy dose for maxillary sinus cancer with destruction of the orbital floor or posterior wall is:

A. 8000 cGy

B. 3000 cGy

C. 6000 cGy

D. 4000 cGy

E. 9000 cGy

1. The main reason why the facio-cervical combined field for nasopharyngeal carcinoma radiotherapy needs to be split when reaching 36–40 Gy is:

A. To avoid excessive oral reactions

B. To protect the parotid gland

C. To protect the spinal cord

D. To protect the temporomandibular joint

E. To protect the glottis

1. The correct management when obvious parotid swelling and pain occur during radiotherapy for head and neck tumors is:

A. Apply hot compress only

B. No need to stop radiotherapy or special treatment

C. Hold Vitamin C in mouth only

D. Keep oral cavity clean, give low-dose corticosteroids

E. Besides oral hygiene, give systemic anti-inflammatory treatment and pause radiotherapy

1. The most reasonable method for handling the gap width between total central nervous system (CNS) radiotherapy fields is:

A. Calculate the gap width based on SSD, field length, and lesion depth

B. Do not set a gap to prevent missing lesions

C. Gap of 1cm, move the junction up or down once for every 1000 cGy irradiated

D. Do not set a gap but move the junction up or down once for every 1000 cGy irradiated

E. Gap of 1cm or more to prevent spinal cord injury caused by overlapping irradiation areas

1. The preferred treatment method for early hypopharyngeal cancer is:

A. Hyperthermia

B. Surgical treatment

C. Chemotherapy

D. Radiotherapy

E. Molecular targeted therapy

1. Male patient, 64 years old, recurrent epistaxis for 3 months, accompanied by headache and tinnitus for over a month. Patient's MR image is shown (described as: 6cm mass in nasopharynx, low signal on T1WI, iso-signal on T2WI, obvious extensive cervical lymphadenopathy). The most likely diagnosis is:

A. Nasopharyngeal angiofibroma

B. Malignant lymphoma

C. Nasopharyngeal carcinoma

D. Craniopharyngioma

E. Chronic nasopharyngitis

1. Male patient, 56 years old, presented with "recurrent nasal bloody discharge with nasal congestion for over 6 months". Physical examination revealed a neoplasm in the left nasal cavity. CT showed a space-occupying lesion in the left nasal cavity, with invasion of the nasal septum and the medial wall of the left maxillary sinus. The most likely diagnosis for this patient is:

A. Esthesioneuroblastoma

B. Nasal lymphoma

C. Nasal cavity cancer

D. Maxillary sinus cancer

E. Nasal adenoid cystic carcinoma

1. Female patient, 42 years old, felt right facial swelling 3 months ago. In the recent 2 weeks, she developed right facial pain, right nasal congestion, accompanied by epistaxis and right ear hearing loss. The correct diagnosis for this patient is:

A. Nasopharyngeal carcinoma

B. Nasal lymphoma

C. Nasal cavity cancer

D. Maxillary sinus cancer

E. Parotid cancer

1. Male patient, 59 years old, unilateral throat pain for half a year, radiating to the ear, pain aggravated when eating or drinking, trismus for two days. Physical examination revealed a neoplasm in the right tonsillar region. The first consideration is possibly:

A. Tongue cancer

B. Tonsillar cancer

C. Hard palate cancer

D. Buccal mucosa cancer

E. Floor of mouth cancer

1. Female patient, 35 years old, developed local recurrence half a year after radical radiotherapy for tonsillar cancer. The first treatment method to consider at this time is:

A. Immunotherapy

B. Re-irradiation

C. Chemotherapy

D. Surgical treatment

E. Laser therapy

1. Male patient, 50 years old, discovered an ulcer on the right lateral border of the tongue for half a year. Right ear pain appeared in the past week, accompanied by enlarged cervical lymph nodes. After completing examinations, the clinical diagnosis was tongue cancer cT3N1M0. The correct management principle is:

A. Palliative radiotherapy

B. Surgery alone

C. Preoperative concurrent chemoradiotherapy + Surgery

D. Radical radiotherapy

E. Chemotherapy

1. Male, 67 years old. Diagnosed with vocal cord cancer 10 years ago due to hoarseness and underwent radical radiotherapy. The patient developed hoarseness again in the past 2 months, accompanied by chest tightness, cough, and blood-stained sputum. History of smoking for 40 years. Physical exam: a mass about 1.5cm x 1cm palpable in the right supraclavicular fossa, fixed and non-tender. Direct laryngoscopy revealed a neoplasm on the left vocal cord, pathology indicated well-differentiated squamous cell carcinoma. Chest CT indicated a 5cm x 5cm mass in the right lower lung with spiculated margins, obvious enlargement of right hilar and mediastinal lymph nodes. Fiberoptic bronchoscopy biopsy confirmed poorly differentiated adenocarcinoma. Which of the following is the more comprehensive diagnosis for this patient?

A. Local recurrence of vocal cord cancer with lung metastasis

B. Local recurrence of vocal cord cancer

C. Local recurrence of vocal cord cancer combined with second primary tumor (lung cancer)

D. Lung cancer Stage IV (metastasis to left vocal cord)

E. Metastatic cancer of unknown primary (left vocal cord, lung, lymph node)

1. Male, 62 years old. Discovered a superficial ulcer about 0.5cm x 0.5cm on the left lateral border of the tongue half a year ago, which progressively enlarged, gradually hardened, became fixed, and enlarged lymph nodes were palpable in the neck. Biopsy of the tongue showed moderately differentiated squamous cell carcinoma. Oropharynx + Neck MR indicated a mass of about 3cm on the left lateral border of the tongue invading the genioglossus muscle; multiple significantly enlarged lymph nodes visible in the left upper neck, with the largest diameter about 3.5cm. Other auxiliary examinations showed no abnormalities. The current clinical stage of the patient belongs to:

A. cT4N2M0

B. cT1N1M0

C. cT3N2M0

D. cT2N1M0

E. cT4N3M0

1. Male, 69 years old, supraglottic poorly differentiated squamous cell carcinoma T2N0M0. Sore throat appeared again 4 months after radical radiotherapy. The currently appropriate management method is:

A. Vocal cord stripping

B. Antibiotic treatment + corticosteroids

C. Systemic chemotherapy

D. Immediate laryngectomy

E. Laser therapy

1. Female, 43 years old, teacher. Carcinoma in situ of the middle 1/3 of the left vocal cord underwent vocal cord stripping. Hoarseness reappeared 2 years post-surgery. Laryngoscopy showed slight elevation of the left vocal cord mucosa. Biopsy showed well-differentiated squamous cell carcinoma invading the mucosa, without submucosal infiltration. The preferred next treatment is:

A. Radical radiotherapy

B. Repeat vocal cord stripping

C. Cordectomy

D. Laser therapy

E. Systemic chemotherapy

1. Male, 57 years old, hoarseness accompanied by throat discomfort for half a year. Physical exam: a hard fixed mass palpable in the left neck, non-tender. Most likely is:

A. Glottic cancer

B. Nasopharyngeal carcinoma

C. Tonsillar cancer

D. Vocal cord polyp

E. Laryngeal tuberculosis

1. Female, 54 years old, underwent laryngoscopy due to swallowing pain and found a neoplasm in the pyriform sinus. Part of the neoplasm was taken for examination. Which of the following primary tumor histological types is impossible to exist in the biopsy?

A. Melanoma

B. Squamous cell carcinoma

C. Soft tissue sarcoma

D. Malignant lymphoma

E. Dysgerminoma (Seminoma)

1. Male, 60 years old, progressive hoarseness for half a year. Diagnosed with late-stage glottic cancer after comprehensive examination upon admission. Which of the following symptoms generally does not appear in late-stage glottic cancer?

A. Diplopia

B. Blood in sputum

C. Disappearance of laryngeal crepitus

D. Dyspnea

E. Hard, fixed enlarged lymph node in the left neck

1. A nasopharyngeal carcinoma patient developed massive nasopharyngeal bleeding during radiotherapy. At this time, the best emergency hemostatic measure is:

A. Endotracheal intubation to prevent asphyxia

B. Increase radiotherapy dose to stop bleeding

C. Nasopharyngeal packing/tamponade

D. Use potent hemostatic drugs

E. Combine with high-dose chemotherapy

1. Male, 33 years old, bloody nasal discharge on suction for half a year. CT showed thickening of the right wall of the nasopharynx, disappearance of the pharyngeal recess, enlargement of bilateral retropharyngeal and bilateral deep cervical lymph nodes, with signs of marginal enhancement and internal necrosis in the lymph nodes. The most likely diagnosis is:

A. Chordoma

B. Nasopharyngeal carcinoma

C. Lymphoma

D. Nasopharyngeal adenoidal hypertrophy

E. Malignant fibrous histiocytoma

1. Male, 32 years old, right neck mass for 1 month. CT scan showed a soft tissue mass in the right oropharynx and nasopharynx, uniform density, multiple enlarged lymph nodes in bilateral neck and mediastinum, with clear boundaries. The most likely diagnosis is:

A. Tuberculosis

B. Nasopharyngeal carcinoma invading oropharynx with lymph node metastasis

C. Fibrosarcoma invading nasopharynx and oropharynx with lymph node metastasis

D. Malignant fibrous histiocytoma invading nasopharynx and oropharynx with lymph node metastasis

E. Lymphoma

1. Male patient, 51 years old, presented with "nasal congestion accompanied by headache for half a year". Nasopharyngoscopy found obvious mucosal elevation in the right nasopharynx. Biopsy confirmed nasopharyngeal adenoid cystic carcinoma. Which of the following statements is correct?

A. Because the total dose required for radiotherapy of this type is high, generally reaching about 80 Gy, choosing IMRT is more conducive to protecting important tissues and organs, while avoiding cranial nerves and their pathways as much as possible in the radiation field

B. Nasopharyngeal adenoid cystic carcinoma tends to have local infiltrative growth and is more prone to local lymph node metastasis than poorly differentiated squamous cell carcinoma of the nasopharynx

C. The patient should undergo further relevant examinations (such as CT/MR) to clarify the stage. If the local lesion is extensive, radiotherapy is still the main treatment method

D. This type of nasopharyngeal carcinoma is mostly insensitive to radiotherapy, so the patient should be treated with surgery + chemotherapy mode

E. The prognosis of adenoid cystic carcinoma is extremely poor, and most patients die within 1 year after diagnosis

## Questions with Shared Clinical Vignettes

**[Case] Male patient, 27 years old, admitted for "swelling and pain in the left mandible for over 3 months". Physical exam: swelling of the left mandibular angle, distention of superficial veins on the left face, elevated skin surface temperature; X-ray showed bone destruction of the left mandible, elevated blood alkaline phosphatase.**

1. Which of the following is correct regarding this disease?

A. Skin ulceration is rare

B. Occurs frequently in middle-aged patients

C. Prone to lymph node metastasis

D. Incidence is similar in men and women

E. The higher the alkaline phosphatase, the better the prognosis

1. The most likely diagnosis for this patient is:

A. Osteomyelitis of the mandible

B. Osteosarcoma of the mandible

C. Fibrosarcoma of the mandible

D. Chondrosarcoma of the mandible

E. Tuberculosis of the mandible

1. The best treatment for this patient is:

A. Immunotherapy

B. Radiotherapy

C. Chemotherapy

D. Surgical surgery

E. Traditional Chinese medicine treatment

**[Case] Male patient, 60 years old, presented with "hoarseness for over half a month accompanied by sore throat". Physical exam found a palpable enlarged lymph node of 2cm x 4cm in the neck. Neck MRI indicated: 1) neoplasm in the right pyriform sinus, invading the right vocal cord, partial invasion of the cricoid cartilage; 2) multiple enlarged lymph nodes in the bilateral neck, maximum diameter < 6cm.**

1. The patient was confirmed to have poorly differentiated squamous cell carcinoma of the pyriform sinus by biopsy. Other auxiliary examinations showed no distant metastasis. The stage is considered to be:

A. cT4N2M0

B. cT1N1M0

C. cT3N2M0

D. cT2N1M0

E. cT4N3M0

1. Which of the following examinations is most significant for further confirmation?

A. Sputum cytology

B. Neck lymph node puncture cytology

C. Neck B-ultrasound

D. Fiberoptic laryngoscopy + biopsy

E. Neck CT

1. Which of the following treatment plan choices do you consider unreasonable?

A. Preoperative induction chemotherapy + Surgery + Postoperative radiotherapy

B. Surgery + Postoperative radiotherapy

C. Preoperative concurrent chemoradiotherapy + Surgery

D. Preoperative radiotherapy + Surgery

E. Simple chemotherapy

1. Regarding the conventional preoperative radiotherapy for this patient, which of the following statements is incorrect?

A. When conventional irradiation reaches 36Gy–40Gy, the posterior border of the horizontal field should be moved forward appropriately to avoid the spinal cord, and electron beams can be used to boost the posterior neck lymphatic drainage area

B. The upper border is generally at the level of the skull base, the lower border reaches the esophageal inlet (equivalent to the level of the lower border of the cricoid cartilage), and the tumor bed area includes the nasopharynx, oropharynx, parapharyngeal space, hypopharynx, larynx, and cervical esophageal inlet

C. The dose for simple preoperative radiotherapy is generally 50 Gy. If concurrent chemoradiotherapy is performed, the dose can be reduced to about 45 Gy

D. Regional lymph node irradiation includes bilateral cervical Ib~V lymph drainage areas and bilateral retropharyngeal lymph nodes; prophylactic irradiation is performed for the lower neck and supraclavicular lymph drainage areas. Therefore, the posterior border of the horizontal radiation field for this patient is placed at the spinous process of the cervical vertebrae

E. For the junction of the facio-cervical combined horizontal field and the lower neck tangential field, half-field junction or regular adjustment of the junction site can be selected to reduce dose hot spots or cold spots

**[Case] Male patient, 59 years old, hoarseness accompanied by throat discomfort for half a year, blood in sputum for 1 month. Physical exam: expansion of thyroid cartilage, a tough fixed mass about 2cm x 2.5cm palpable in the right upper neck (Level II), non-tender. Denies history of tuberculosis. Patient's neck MRI indicated the tumor is located in the supraglottic region, invading the pre-epiglottic space, left pharyngeal wall, epiglottis, and thyroid cartilage; left vocal cord is fixed; no invasion of oropharynx and parapharyngeal space; multiple enlarged lymph nodes visible in the upper neck. Direct laryngoscopy biopsy indicated supraglottic moderately differentiated squamous cell carcinoma. No metastasis seen in other auxiliary examinations.**

1. If the patient undergoes preoperative radiotherapy using conformal radiotherapy or IMRT, which statement regarding the lymph node irradiation range is correct?

A. Irradiation range includes right cervical levels I~V lymph node regions + retropharyngeal lymph nodes

B. Patient's ipsilateral Level II lymph nodes are positive, CTV does not need to include ipsilateral submandibular lymph nodes (i.e., Level I)

C. Irradiation range includes bilateral cervical levels I~V lymph node regions + retropharyngeal lymph nodes

D. Retropharyngeal lymph nodes do not undergo prophylactic irradiation

E. Superior mediastinal lymph nodes also need prophylactic irradiation

1. If conventional preoperative radiotherapy is performed, which of the following statements is incorrect?

A. Re-examine at DT 40–50 Gy, if tumor regression is satisfactory, switch to radical radiotherapy

B. The irradiation target area should include the primary tumor area + regional lymph node drainage area

C. Upper and middle cervical lymph node drainage areas must be irradiated, while lower neck and supraclavicular areas do not undergo prophylactic irradiation

D. The upper border of the conventional bilateral horizontal field is at the level of the first cervical vertebra, the lower border is the lower border of the cricoid cartilage, the anterior border is the anterior margin of the neck, and the posterior border should be the posterior margin of the spinous process

E. When the irradiation dose approaches 36–40 Gy, the cervical spinal cord should be avoided, and the posterior neck area supplemented with electron beam irradiation

1. According to relevant examinations, the patient's clinical stage belongs to:

A. cT4N2M0

B. cT1N1M0

C. cT3N2M0

D. cT2N1M0

E. cT4N3M0

**[Case] Male patient, 45 years old, smoker for 20 years, hoarseness for over 1 month, accompanied by laryngeal foreign body sensation and throat pain. Physical exam: PS=1, no superficial lymph node enlargement in the neck, tonsils not enlarged, obvious congestion of the posterior pharyngeal wall. Indirect laryngoscopy revealed restricted movement of the right vocal cord, neoplasm seemingly visible locally.**

1. The patient requests to restore voice as much as possible due to work reasons. The reasonable treatment method to choose is:

A. Vertical hemilaryngectomy

B. Surgery + Postoperative radiotherapy

C. Preoperative radiotherapy + Surgery + Postoperative adjuvant chemotherapy

D. Simple chemotherapy

E. Radical radiotherapy ± Concurrent chemotherapy

1. Direct laryngoscopy revealed the disappearance of the original shape of the patient's right vocal cord, restricted movement, a 2cm x 2cm mass visible locally involving the anterior commissure, restricted movement of the left vocal cord, no tumor invasion in the supraglottic and subglottic regions. Biopsy showed (laryngeal) squamous cell carcinoma. Auxiliary exams: Neck MRI indicated tumor confined to the right vocal cord, no cervical lymph node enlargement. Chest X-ray and abdominal B-ultrasound showed no abnormalities. The clinical stage is considered to be:

A. cT4N0M0

B. cT1N0M0

C. cT3N0M0

D. cT2N0M0

E. Cannot be determined

1. Combining clinical symptoms, the first possible tumor diagnosis to consider is:

A. Hypopharyngeal cancer

B. Esophageal cancer

C. Lung cancer

D. Laryngeal cancer

E. Tonsillar cancer

**[Case] Male patient, 40 years old, nasal congestion and bloody nasal discharge on suction for over 3 months, discovered left neck mass for over 2 weeks. Physical exam: a 3cm x 4cm mass in the upper left neck, hard texture, clear boundaries, poor mobility.**

1. The most likely diagnosis for this patient among the following is:

A. Maxillary sinus cancer

B. Esthesioneuroblastoma

C. Nasopharyngeal lymphoma

D. Nasopharyngeal carcinoma

E. Nasopharyngeal tuberculosis

1. To clarify the diagnosis, the first examination required for this patient is:

A. B-ultrasound

B. CT

C. MRI

D. Electronic nasopharyngoscopy and biopsy

E. EB virus antibody detection

**[Case] Female patient, 26 years old, 37 weeks pregnant, presented with "nasal congestion for 2 months, intermittent bloody nasal discharge for 3 days". Physical exam found multiple enlarged lymph nodes in bilateral upper neck, max diameter about 3cm. Direct nasopharyngoscopy found disappearance of the right pharyngeal recess (Fossa of Rosenmuller), local cauliflower-like neoplasm visible. Biopsy confirmed nasopharyngeal poorly differentiated squamous cell carcinoma.**

1. Patient completed other examinations with no distant metastasis found. Nasopharynx and Neck MRI indicated local tumor invasion of the pterygopalatine fossa, invasion of the medial pterygoid muscle, multiple enlarged lymph nodes visible in the upper neck. Currently, the diagnostic stage is considered to be:

A. cT4N2M0

B. cT1N1M0

C. cT3N2M0

D. cT2N1M0

E. cT3N1M0

1. The site of action of this drug in the cell is:

A. RAF gene expression product

B. VEGR receptor

C. Extracellular domain of EGFR receptor

D. Intracellular tyrosine kinase site of EGFR

E. Intracellular tyrosine kinase site of VEGFR

1. The patient complained that the neck mass has increased significantly in recent days, and nasal congestion symptoms have aggravated. Regarding the next step of treatment, the reasonable statement is:

A. If local progression is rapid, surgical resection can be considered first to avoid the impact of chemoradiotherapy on the fetus, and radiotherapy can be performed according to the surgical situation

B. Local radiotherapy has no effect on the fetus and can be performed directly according to routine radical radiotherapy

C. Since nasopharyngeal carcinoma during pregnancy progresses rapidly, obstetrics opinion can be sought to terminate pregnancy and start chemoradiotherapy as soon as possible

D. Suggest waiting until after the patient gives birth before conducting anti-tumor treatment

E. The prognosis of nasopharyngeal carcinoma in pregnancy is relatively good, and it often regresses spontaneously after termination of pregnancy, suggesting close observation

1. If the patient chooses molecular targeted drugs for concurrent radiotherapy sensitization, which drug has been approved by the US FDA for head and neck sensitization treatment?

A. Bevacizumab

B. Iressa (Gefitinib)

C. Sorafenib

D. Tarceva (Erlotinib)

E. Erbitux (Cetuximab)

## Questions with Shared Options

[Options]

A. Esthesioneuroblastoma

B. Squamous cell carcinoma

C. Malignant melanoma

D. Adenocarcinoma

E. Lymphoma

1. Which pathological type of malignant tumor of the nasal cavity and paranasal sinuses is often accompanied by surrounding satellite lesions and cervical lymph node metastasis?
2. Which pathological type of malignant tumor of the nasal cavity and paranasal sinuses has two peak ages of onset?
3. The most common pathological type of malignant tumor of the nasal cavity and paranasal sinuses is:

[Options]

A. Nasal congestion

B. Facial swelling

C. Cranio-orbital pain

D. Epistaxis

E. Eyeball displacement

1. The earliest symptom of maxillary sinus cancer is:
2. The most common symptom of nasal cavity cancer is:

[Options]

A. Biopsy

B. Bone ECT

C. Magnetic Resonance Imaging (MRI)

D. CT scan

E. Immunohistochemistry

1. The most accurate diagnostic means for malignant tumors of the nasal cavity and paranasal sinuses is:
2. The preferred diagnostic method for malignant tumors of the paranasal sinuses is:

[Options]

A. Biopsy

B. Magnetic Resonance Imaging (MRI)

C. Bone ECT

D. CT scan

E. Immunohistochemistry

1. The diagnostic method that can accurately display the extent of paranasal sinus tumor lesions and distinguish between tumor and inflammation is:
2. The examination most helpful for the confirmed diagnosis of esthesioneuroblastoma is:

[Options]

A. Immunotherapy

B. Radiotherapy

C. Chemotherapy

D. Surgical surgery

E. Traditional Chinese medicine treatment

1. The preferred treatment for jaw fibrosarcoma is:
2. The preferred treatment for jaw Ewing's sarcoma is:
3. The preferred treatment for jaw plasmacytoma (plasma cell sarcoma) is:

[Options]

A. Lymphoma

B. Undifferentiated carcinoma

C. Mucoepidermoid carcinoma

D. Squamous cell carcinoma

E. Adenoid cystic carcinoma

1. The most common minor salivary gland cancer is:
2. The most common submandibular gland tumor is:

[Options]

A. Lip cancer

B. Tongue cancer

C. Gingival cancer

D. Floor of mouth cancer

E. Buccal mucosa cancer

1. Among the above oral cancers, the one with the lowest incidence is:
2. The most common oral tumor in head and neck tumors, second only to skin cancer, is:
3. Among oral cancers, the one most prone to cervical lymph node metastasis is:

[Options]

A. Surgery alone

B. Preoperative radiotherapy + Radical surgery

C. Radical radiotherapy

D. Surgery ± Postoperative radiotherapy

E. Simple chemotherapy

1. For early oropharyngeal cancer, if it is poorly differentiated or undifferentiated carcinoma and requires preservation of organ function as much as possible, the first choice is:
2. For tongue cancer, if it is a large exophytic tumor without necrosis/ulceration or surrounding soft tissue infiltration, the first choice is:
3. For early tongue base squamous cell carcinoma appearing with local recurrence half a year after radical radiotherapy, the first choice is:

[Options]

A. Dyspnea

B. Hoarseness

C. Blood in sputum

D. Pharyngeal foreign body sensation

E. Neck mass

1. The most common presenting symptom of late subglottic cancer is:
2. The early main manifestation of supraglottic cancer is:
3. The relatively typical clinical manifestation of glottic cancer is:

[Options]

A. Tonsillar cancer

B. Supraglottic cancer

C. Subglottic cancer

D. Glottic cancer

E. Hypopharyngeal cancer

1. Tumors characterized by spread along the mucosa or submucosa are more common in:
2. Head and neck tumors where poorly differentiated and undifferentiated carcinomas are common are seen in:
3. Cervical lymph node metastasis is less common in:

[Options]

A. Pyriform sinus region, Waldeyer's ring region, lateral pharyngeal wall region, prevertebral region

B. Pyriform sinus region, post-cricoid region, posterior pharyngeal wall region

C. Anterior wall, roof, posterior wall, lateral wall

D. Supraglottic region, glottic region, subglottic region

E. Tongue-epiglottis region, tonsil, parapharyngeal space, retropharyngeal space

1. Clinically, the hypopharynx is usually divided according to anatomical sites into:
2. According to the UICC (2002) staging standard, the 4 anatomical subdivisions of the oropharynx are:
3. Clinically, laryngeal cancer is divided according to anatomical characteristics into:

[Options]

A. Ophthalmoplegia first, then visual impairment

B. Affected eye fixed, slightly protruding outward

C. Visual impairment first, then ophthalmoplegia

D. Superior muscle paralysis of the affected eye, eyeball fixed, then exophthalmic visual paralysis

E. Exophthalmos

1. The clinical manifestation of orbital apex syndrome in nasopharyngeal carcinoma is:
2. The clinical manifestation of superior orbital fissure syndrome in nasopharyngeal carcinoma is:
3. The clinical manifestation of pituitary-sphenoid syndrome in nasopharyngeal carcinoma is:

# Chapter 2: Lung Cancer and Pleural Mesothelioma (N=55)

## Standard Multiple-Choice Questions

1. Which of the following methods is not a qualitative diagnostic method for lung cancer?

A. Endoscopic examination of lung cancer

B. Sputum cytology

C. Percutaneous fine-needle lung aspiration cytology

D. Pleural effusion cytology

E. PET scan

2. When a lung cancer patient undergoes X-ray examination, both posteroanterior (PA) and lateral chest radiographs must be performed. Adding a lateral chest radiograph can increase the detection rate of lung cancer by:

A. 7%

B. 1%

C. 5%

D. 3%

E. 9%

3. The comprehensive treatment modality that should be adopted for small cell lung cancer is:

A. Primarily molecular targeted therapy

B. Primarily surgery

C. Primarily radiotherapy

D. Primarily chemotherapy

E. Primarily chemoradiotherapy

4. The smoking index (cigarettes smoked per day × years of smoking) for the high-risk population of lung cancer should be greater than:

A. 400

B. 100

C. 300

D. 200

E. 500

5. The site of occurrence for central lung cancer is:

A. Proximal to the opening of bronchioles

B. Proximal to the opening of main bronchi

C. Proximal to the opening of segmental bronchi

D. Proximal to the opening of lobar bronchi

E. Proximal to the opening of terminal bronchioles

6. The most common symptom of lung cancer is:

A. Chest pain

B. Cough

C. Chest tightness

D. Bloody sputum

E. Shortness of breath

7. The efficacy of Gefitinib is not correlated with which of the following factors?

A. EGFR expression

B. Stage

C. Gender

D. Never-smokers

E. Adenocarcinoma

8. Clinical treatment for acute radiation pneumonitis with obvious symptoms does not include:

A. Antibiotics

B. Oxygen inhalation

C. Corticosteroids

D. Bronchodilators

E. Amifostine

9. Limited stage small cell lung cancer does not include:

A. Small amount of pleural effusion

B. Ipsilateral mediastinal lymph node metastasis

C. Ipsilateral diaphragm invasion

D. Ipsilateral supraclavicular lymph node metastasis

E. Mild superior vena cava syndrome

10. The most common pathological type of small cell lung cancer is:

A. Intermediate cell type

B. Large cell type

C. Carcinosarcoma

D. Oat cell type

E. Mixed cell type

11. Regarding radiotherapy after radical resection for non-small cell lung cancer, which of the following statements is correct?

A. Applicable to T4N1M0 cases

B. Applicable to cases with residual tumor after surgery

C. Applicable to T2N1M0 cases

D. Applicable to T4N0M0 cases

E. Applicable to T3N0M0 cases

12. The time (dose) when acute radiation esophagitis appears during radiotherapy for lung cancer patients is:

A. Around DT 40–60 Gy

B. Around DT 10–30 Gy

C. Around DT 30–50 Gy

D. Around DT 20–40 Gy

E. Around DT 50–70 Gy

13. Currently in China, the incidence and mortality rates of lung cancer rank where among urban malignant tumors?

A. 4th

B. 1st

C. 3rd

D. 2nd

E. 5th

14. Regarding the treatment principles for non-small cell lung cancer, which statement is incorrect?

A. Stage IIIA patients with good general condition and no pleural effusion should undergo concurrent chemoradiotherapy

B. Stage IA is treated primarily with surgery

C. Stage IIIA patients undergo surgery first, followed by radiotherapy and chemotherapy

D. Stage IB and II patients undergo surgery first, followed by radiotherapy and/or chemotherapy

E. Stage IV patients should undergo comprehensive treatment based primarily on systemic therapy

15. The method most frequently applied in the treatment of malignant pleural mesothelioma is:

A. Molecular targeted therapy

B. Surgery

C. Radiotherapy

D. Chemotherapy

E. Palliative treatment

16. The scope of standard radical surgery for malignant pleural mesothelioma does not include:

A. Diaphragm

B. Ipsilateral pleura

C. Pericardium

D. Whole lung

E. Mediastinal lymph nodes

17. Which of the following is not an indication for surgery in malignant pleural mesothelioma?

A. Patient accompanied by unbearable chest pain

B. Lesion concentrated in one side of the thoracic cavity, no distant metastasis

C. Patient is relatively young, can withstand surgical trauma, and is expected to receive adjuvant therapy and have good quality of life postoperatively

D. Diffuse malignant pleural mesothelioma

E. Recurrent pleural effusion difficult to control and ineffective with other treatments

18. Which of the following is currently the chemotherapy regimen with the best efficacy for malignant pleural mesothelioma?

A. Pemetrexed + Oxaliplatin

B. Raltitrexed + Cisplatin

C. Pemetrexed + Cisplatin

D. Raltitrexed + Oxaliplatin

E. Gemcitabine + Cisplatin

19. Currently, the only means by which malignant pleural mesothelioma might potentially be cured is:

A. Molecular targeted therapy

B. Surgery

C. Chemotherapy

D. Radiotherapy

E. Palliative treatment

20. Male patient, 57 years old, recurrent cough and expectoration for over a month. X-ray image follows (below). What is the most likely diagnosis?

A. Peripheral lung cancer

B. Pulmonary tuberculoma

C. Central lung cancer

D. Pulmonary metastasis

E. Thymoma

21. Male patient, 64 years old, more than a year after liver cancer surgery, now presents with recurrent cough and expectoration. X-ray image follows (below). What is the most likely diagnosis?

A. Pulmonary tuberculosis

B. Central lung cancer

C. Pulmonary metastatic carcinoma

D. Peripheral lung cancer

E. Pneumonia

22. Male patient, 54 years old, recurrent cough and expectoration for 3 months. CT image follows (below). What is the most likely diagnosis?

A. Pulmonary tuberculosis

B. Central lung cancer

C. Lung abscess

D. Peripheral lung cancer

E. Pneumonia

23. Female patient, 39 years old. Admitted for "bilateral knee pain for 5 months, dry cough for 2 months, hemoptysis for 3 days". Physical exam: superficial lymph nodes not palpable, clubbing of fingers present. Chest CT shows a space-occupying lesion in the left hilum, biopsy reported poorly differentiated adenocarcinoma. X-ray shows periosteal proliferation of the lower segment of both femurs. This manifestation is:

A. Periostitis

B. Carcinoid syndrome

C. Synovitis

D. Rheumatoid arthritis

E. Hypertrophic pulmonary osteoarthropathy

24. Male patient, 67 years old. Admitted for "left lung space-occupying lesion found during physical exam". Underwent left upper lobectomy. Postoperative pathology: left upper lung well-differentiated squamous cell carcinoma, lymph node metastasis, cancer tissue visible at the residual end. Patient underwent 4 cycles of NP regimen chemotherapy postoperatively, followed by radiotherapy. When radiotherapy reached 52 Gy, the patient suddenly developed cough and chest tightness. Chest X-ray: increased interstitial density in the left lower lobe, small amount of left-sided pleural effusion. At this time, the diagnosis to consider is:

A. Radiation pneumonitis

B. Lobar pneumonia

C. Bronchiolitis obliterans organizing pneumonia

D. Lobular pneumonia

E. Pleurisy

## Questions with Shared Clinical Vignettes

**[Case 1] Female patient, 57 years old, admitted for "cough and chest tightness for over half a month, aggravated for 2 days". Chest CT shows: space-occupying lesion in the peripheral zone of the right upper lung lobe, enlarged and increased mediastinal lymph nodes, moderate right-sided pleural effusion. Pleural fluid cytology shows adenocarcinoma cells. Head MRI, abdominal B-ultrasound, and bone ECT show no abnormalities.**

25. If the patient's PS score is 1, the preferred treatment method is:

A. Molecular targeted therapy

B. Surgery

C. Radiotherapy

D. Chemotherapy

E. Best supportive care

26. The patient's diagnosis and stage belong to:

A. Right lung adenocarcinoma cT4N2M0, Stage IIIA

B. Right lung adenocarcinoma cT4N1M0, Stage IIIA

C. Right lung adenocarcinoma cT3N2M0, Stage IIIB

D. Right lung adenocarcinoma cT3N2M0, Stage IIIA

E. Right lung adenocarcinoma cT4N2M0, Stage IIIB

27. If the patient's PS score is 3, the preferred treatment method is:

A. Molecular targeted therapy

B. Surgery

C. Radiotherapy

D. Chemotherapy

E. Best supportive care

**[Case 2] Male patient, 70 years old, left glottic moderately differentiated squamous cell carcinoma T2N0M0. 15 years after left cordectomy, he presents again with hoarseness, cough, and expectoration. Physical exam: laryngeal contour intact, no enlarged lymph nodes in both sides of the neck. Laryngoscopy shows no local abnormalities. History of smoking for over 30 years.**

28. Which of the following examinations is of little significance for helping diagnosis and subsequent treatment?

A. Neck B-ultrasound

B. Head and neck MRI

C. Sputum cytology

D. Laryngoscopy

E. Chest CT examination

29. Fiberoptic bronchoscopy biopsy confirmed small cell lung cancer. The next treatment strategy is:

A. Comprehensive treatment based mainly on chemotherapy, including surgery and radiotherapy

B. Radical surgery

C. Radiotherapy alone

D. Molecular targeted therapy

E. Chemotherapy alone

30. Patient's chest CT shows a space-occupying lesion approx. 4cm × 2cm in the right upper lung with surrounding spiculation, and obvious mediastinal lymph node enlargement. Head and neck MR examination shows no abnormalities. Blood NSE is significantly elevated. The most likely diagnosis to consider is:

A. Inflammatory pseudotumor

B. Lung metastasis from postoperative recurrence of laryngeal cancer

C. Pulmonary tuberculosis

D. Second primary tumor: Lung cancer probable

E. Metastatic lung tumor of unknown primary origin

**[Case 3] Male patient, 46 years old. Admitted for "dry cough for 6 months". Physical exam: superficial lymph nodes not palpable. Chest CT shows space-occupying lesion in the right upper lobe. Fiberoptic bronchoscopy shows neoplasm at the opening of the right upper lobe bronchus, biopsy reported small cell carcinoma.**

31. The scheme that should not be chosen for the patient's initial chemotherapy is:

A. VP-16 + IFO + DDP

B. CBP + VP-16

C. CTX + ADM + VCR

D. VP-16 + DDP

E. CTX + ADM + DDP

32. If the patient undergoes head MRI, abdominal B-ultrasound, and bone ECT and no abnormalities are found, the treatment the patient should receive is:

A. Surgery + Chemotherapy

B. Surgery

C. Radiotherapy

D. Chemotherapy

E. Chemotherapy + Radiotherapy

33. The tumor marker most valuable for helping judge small cell lung cancer is:

A. CA199

B. CEA

C. Cyfra211

D. NSE

E. CA125

34. If prophylactic radiotherapy is performed for the patient, the site that should receive treatment is:

A. Liver

B. Thoracic spine

C. Brain

D. Lumbar spine

E. Pancreas

**[Case 4] Male patient, 62 years old. Admitted because "left lung space-occupying lesion found during physical exam 2 days ago". History of smoking for 35 years. Admission chest CT shows: space-occupying lesion approx. 2cm in diameter in the left upper lung lobe, no enlarged lymph nodes in the mediastinum. Fiberoptic bronchoscopy shows neoplasm in the left upper lobe bronchus, biopsy shows: well-differentiated squamous cell carcinoma. Patient's head MRI, abdominal B-ultrasound, and bone ECT showed no abnormalities.**

35. The patient's diagnosis is:

A. Left upper lung well-differentiated squamous cell carcinoma pT2N0M0

B. Left upper lung well-differentiated squamous cell carcinoma cT1N0M0

C. Left upper lung well-differentiated squamous cell carcinoma cT2N0M0

D. Left upper lung well-differentiated squamous cell carcinoma pT1N0M0

E. Left upper lung well-differentiated squamous cell carcinoma cT3N0M0

36. If 3 years after surgery or radiotherapy, the patient presents with bilateral lung space-occupying lesions and refuses chemotherapy, the best molecular targeted therapy drug the patient can choose is:

A. Erlotinib

B. Sorafenib

C. Gefitinib

D. Imatinib mesylate

E. Lapatinib

37. If the patient cannot undergo surgery due to cardiac reasons, the best radiotherapy technique the patient can choose under existing equipment conditions is:

A. Four-dimensional radiotherapy

B. Conventional radiotherapy

C. Three-dimensional conformal radiotherapy

D. Stereotactic radiotherapy

E. Real-time tracking radiotherapy

38. If the patient completes relevant examinations and has no surgical contraindications, the preferred surgical method is:

A. Lobectomy + Hilar and mediastinal lymph node dissection

B. Local lung resection

C. Pneumonectomy

D. Lobectomy

E. Extended lung resection + Hilar and mediastinal lymph node dissection

**[Case 5] Male patient, 63 years old. Admitted for "chest and back pain for over 3 months, aggravated for 1 day". Chest CT shows: space-occupying lesion in the right upper lung, enlarged and increased mediastinal lymph nodes, bone destruction of T9 and T10 vertebrae. Biopsy shows: poorly differentiated adenocarcinoma. Bone ECT shows: metastatic lesions in T9, T10, L1, and right 5th rib.**

39. 3 months later, the patient suddenly develops weakness in one limb. The examination that needs to be considered is:

A. Blood biochemistry

B. Head MRI, to understand if there is brain metastasis

C. Abdominal CT

D. Spinal cord MRI

E. No examination needed for now, observe condition changes

40. If the patient currently has normal physical activity, the treatments he should receive exclude:

A. Radiotherapy for T9 and T10 vertebrae

B. Analgesic treatment

C. Chemotherapy

D. Surgery

E. Bisphosphonate treatment

41. If radiotherapy is performed on T9 and T10 vertebrae, the irradiation dose and fractionation that maintain the analgesic effect for the longest time are:

A. DT 8 Gy / 1 F

B. DT 40 Gy / 20 F

C. DT 20 Gy / 5 F

D. DT 30 Gy / 10 F

E. DT 6 Gy / 1 F

42. Based on question 3, if brain metastasis appears, the immediate subsequent treatment should be:

A. Oral Temozolomide chemotherapy

B. Molecular targeted therapy

C. Palliative radiotherapy assisted by dehydration therapy

D. Systemic chemotherapy

E. Best supportive symptomatic treatment

## Questions with Shared Options

[Options]

A. Adenosquamous carcinoma

B. Squamous cell carcinoma

C. Large cell carcinoma

D. Adenocarcinoma

E. Small cell carcinoma

**43. The histological type of lung cancer with high malignancy and easy metastasis is:**

**44. The histological type that is predominantly central lung cancer is:**

**45. The most common histological type of lung cancer is:**

[Options]

A. Thoracoscopy

B. Fiberoptic bronchoscopy

C. Mediastinoscopy

D. Percutaneous lung biopsy

E. Thoracotomy

**46. Is an important means for lung cancer staging and can also be used for the differential diagnosis of difficult chest diseases:**

**47. For patients with malignant pleural effusion who cannot be diagnosed after non-invasive examinations, the qualitative diagnostic method that can be selected is:**

**48. Patient is healthy with no surgical contraindications. Chest CT suggests an isolated nodular lesion in the outer zone of the right lower lung. The qualitative diagnostic method that should be selected at this time is:**

[Options]

A. Pancoast syndrome

B. Carcinoid syndrome

C. Horner syndrome

D. Superior vena cava obstruction syndrome

E. Cushing syndrome

**49. Biologically active amines produced by argyrophil cells in lung cancer tissue can cause:**

**50. Lung cancer or metastatic lymph nodes involving the sympathetic nerves lateral to the 7th cervical vertebra to the 1st thoracic vertebra can cause:**

**51. Lung cancer or metastatic lymph nodes destroying the 1st and 2nd ribs and brachial plexus can cause:**

[Options]

A. 50–60 Gy

B. 40–50 Gy

C. 20–30 Gy

D. 30–40 Gy

E. 60–70 Gy

**52. The palliative irradiation dose for locally advanced NSCLC is:**

**53. The radical radiotherapy irradiation dose for early-stage NSCLC patients who cannot tolerate surgery or refuse surgery is:**

[Options]

A. 50–60 Gy

B. 20–30 Gy

C. 40–50 Gy

D. 30–40 Gy

E. 60–70 Gy

**54. The irradiation dose for brain metastasis of advanced NSCLC is:**

**55. The local radiotherapy irradiation dose for SCLC is:**

# Chapter 3: Mediastinal Tumors (N=11)

## Standard Multiple-Choice Questions

1. Primary mediastinal lymphoma is mostly located in:

A. Inferior posterior mediastinum

B. Anterior superior mediastinum

C. Inferior anterior mediastinum

D. Posterior superior mediastinum

E. Middle mediastinum

2. Which of the following is the dividing line between the superior mediastinum and the inferior mediastinum?

A. Line connecting the sternal angle and the 4th thoracic intervertebral disc

B. Line connecting the manubrium and the 3rd thoracic intervertebral disc

C. Line connecting the sternal angle and the 3rd thoracic intervertebral disc

D. Line connecting the manubrium and the 4th thoracic intervertebral disc

E. Line connecting the sternal angle and the 5th thoracic intervertebral disc

3. Which of the following is the main cause of death in thymoma?

A. Brain metastasis

B. Local invasion of important mediastinal organs

C. Liver metastasis

D. Myasthenia gravis

E. Bone metastasis

4. Which of the following statements regarding thymoma accompanied by myasthenia gravis is incorrect?

A. More common in males than females

B. Some can heal spontaneously without treatment

C. Incidence rate is 4.3%–54%

D. Some can recur after cure

E. Adults over 40 with myasthenia gravis often have accompanying thymoma

5. Which of the following statements regarding mediastinal teratoma is incorrect?

A. Treatment is primarily surgical

B. It is a benign tumor

C. Typical X-ray shows a round or oval shadow growing to one side in the anterior inferior mediastinum, sometimes lobulated; mostly clear edges, often with visible calcification of the cyst wall or irregular bone shadows

D. Common symptoms include chest tightness, chest pain, cough, shortness of breath, and palpitations

E. Prone to secondary infection

6. Benign neurogenic tumors belong to:

A. Malignant schwannoma

B. Ganglioneuroma

C. Ganglioneuroblastoma

D. Neuroblastoma

E. Neurofibrosarcoma

7. Which statement regarding surgical treatment of mediastinal neurogenic tumors is incorrect?

A. For tumors located anterior to the chest, damage to T3-T4 sympathetic nerves should be avoided to prevent cervical sympathetic paralysis syndrome

B. Once diagnosed, early surgical resection should be performed in principle

C. Most tumors are connected to intercostal nerves or sympathetic nerves

D. Most have intact capsules and are easy to remove completely

E. For those originating from the vagus nerve, care must be taken not to damage the recurrent laryngeal nerve

8. Male patient, 32 years old. Admitted for "head and facial swelling for half a month". Chest CT shows: space-occupying lesion in the lower part of the anterior superior mediastinum. The most likely diagnosis is:

A. Neurogenic tumor

B. Intrathoracic goiter

C. Teratoma

D. Thymic tumor

E. Malignant lymphoma

9. Female patient, 26 years old. Admitted for "intractable hiccups for 2 weeks", physical exam showed no positive signs. Chest CT shows: space-occupying lesion in the anterior inferior mediastinum, partial diaphragm invasion. The nerve likely invaded by this lesion is:

A. Recurrent laryngeal nerve

B. Thoracic sympathetic ganglion

C. Brachial plexus

D. Intercostal nerve

E. Phrenic nerve

10. Female patient, 19 years old. Admitted for "dry cough for 5 months, difficulty breathing for 1 day". The patient coughed up hair-like material after admission. The tumor to consider in diagnosis is:

A. Bronchial cyst

B. Thymic tumor

C. Neurogenic tumor

D. Teratoma

E. Gastroenteric cyst

11. Female patient, 47 years old. Admitted for "progressive dysphagia for over 3 months". Patient has irritating cough, aggravated in supine position. Chest CT shows: clearly outlined space-occupying lesion in the superior mediastinum, lobulated, protruding to both sides, esophagus partially compressed. I-131 scan shows hot nodule in the superior mediastinum. The possible diagnosis for this patient is:

A. Thymic tumor

B. Intrathoracic goiter

C. Thymic cyst

D. Intrathoracic thyroid cyst

E. Bronchial cyst

# Chapter 4: Digestive System Tumors (N=94)

## Standard Multiple-Choice Questions

1. Which of the following is not a clinical manifestation of early esophageal cancer?

A. Retrosternal discomfort or pain while eating

B. Sensation of a foreign body in the esophagus

C. Progressive dysphagia

D. Sensation of food stagnation

E. Retrosternal burning sensation while eating

2. Which of the following is not a clinical manifestation of late esophageal cancer?

A. Persistent chest and back pain

B. Hoarseness

C. Retrosternal burning sensation

D. Choking while eating

E. Progressive dysphagia

3. Esophageal cancer mainly occurs in which of the following tissues?

A. Esophageal submucosal connective tissue

B. Esophageal mucosal epithelial cells

C. Esophageal submucosal glands

D. Para-esophageal lymphoid tissue

E. Esophageal muscle layer

4. Which of the following is a precancerous lesion of esophageal cancer?

A. Barrett's esophagus

B. Hiatal hernia

C. Esophageal papilloma

D. Esophageal polyp

E. Esophageal diverticulum

5. High-grade gastric cancer can skip the routinely associated lymph nodes and directly invade distant lymph nodes. The most common site is:

A. Supraclavicular lymph nodes

B. Splenic hilar lymph nodes

C. Celiac lymph nodes

D. Lesser curvature lymph nodes

E. Left gastric artery lymph nodes

6. The key to improving the cure rate of gastric cancer lies in:

A. Early chemotherapy

B. Early diagnosis

C. Aggressive radiotherapy

D. Radical surgery

E. Comprehensive treatment

7. Precancerous states of gastric cancer exclude:

A. Achlorhydria

B. Gastric polyps

C. Chronic atrophic gastritis

D. Gastric leiomyoma

E. Pernicious anemia

8. Pathologically staging, which of the following falls under the concept of early gastric cancer?

A. No lymph node metastasis

B. Confined to the gastric antrum

C. Diameter within 2cm

D. Confined to the mucosa and submucosa

E. Has not yet invaded the serosal layer

9. Regarding gastric cancer, which of the following statements is incorrect?

A. Gastric cancer generally does not occur in the greater curvature and anterior wall

B. Gastric cancer ranks first among digestive tract tumors in China

C. The most common site of gastric cancer is the antrum, followed by the lesser curvature

D. The age of onset for gastric cancer is most common between 40 and 60 years old

E. Gastric cancer in the cardia region is more common than in the greater curvature

10. Which of the following is the most important diagnostic method for colon cancer?

A. Abdominal B-ultrasound

B. Barium enema X-ray examination

C. Fiberoptic colonoscopy + tissue biopsy

D. CEA determination

E. Digital rectal examination

11. Regarding colon cancer, which of the following is incorrect?

A. Hematogenous metastasis of colon cancer most commonly goes to the liver

B. Colon cancer can be accompanied by anemia and fever

C. Left-sided colon cancer is dominated by systemic toxic symptoms

D. According to tumor morphology, it can be divided into mass type, infiltrating type, and ulcerative type

E. Lymphatic metastasis of colon cancer first spreads to the paracolic lymph nodes

12. In acute obstruction of left-sided colon cancer, which of the following measures is inappropriate?

A. Perform left hemicolectomy immediately after gastrointestinal decompression and obstruction relief

B. Pay attention to correcting water, electrolyte, and acid-base imbalances

C. Operate after brief preparation, perform transverse colostomy first, and perform left hemicolectomy and anastomosis in a second stage

D. Monitor abdominal conditions, estimate the severity of the lesion, insert a gastric tube early, and perform gastrointestinal decompression

E. Perform emergency ECG and blood biochemistry tests, and operate after preliminary correction of acidosis

13. Which of the following factors is most closely related to the pathogenesis of primary liver cancer?

A. Drinking water pollution

B. Liver cirrhosis

C. Radiation exposure

D. Viral hepatitis

E. Parasites

14. Primary liver cancer lymphatic metastasis is most common to which of the following sites?

A. Hilar lymph nodes

B. Supraclavicular lymph nodes

C. Peripancreatic lymph nodes

D. Para-aortic lymph nodes

E. Splenic hilar lymph nodes

15. Extrahepatic hematogenous metastasis of primary liver cancer is most common to which of the following sites?

A. Adrenal gland

B. Brain

C. Lung

D. Bone marrow

E. Kidney

16. Regarding the gross morphological classification of primary liver cancer, which of the following is incorrect?

A. Nodular type is prone to cancer nodule rupture and hemorrhage

B. Massive type cancer diameter > 10cm

C. Massive type is prone to necrosis causing liver rupture

D. Solitary cancer nodules with diameter < 5cm are called small liver cancer

E. Diffuse type often leads to death due to liver failure

17. Which of the following clinical features of primary liver cancer is incorrect?

A. Cancer mass invades hilar bile ducts causing jaundice

B. Rapid growth of the mass pulls the liver capsule causing pain

C. Extra-portal tumor thrombus can produce vascular murmurs

D. Intra-portal tumor thrombus can lead to portal hypertension

E. Tumor grows slowly and can be painless

18. The AFP positivity rate of primary hepatocellular carcinoma is:

A. 60%–70%

B. 100%

C. 70%–90%

D. 80%–90%

E. 50%–60%

19. Which of the following is the most sensitive tumor marker for primary hepatocellular carcinoma?

A. AP

B. Liver B-ultrasound

C. AFP

D. Liver CT

E. r-GT

20. Besides primary liver cancer, which disease can cause AFP > 500 μg/L?

A. Renal embryonal tumor

B. Polycystic liver disease

C. Gonadal embryonal tumor

D. Chronic hepatitis

E. Liver cirrhosis

21. The standard for diagnosing liver cancer with AFP > 200 μg/L after excluding active liver disease is:

A. Normal ALT, AFP > 200 μg/L lasting 8 weeks

B. ALT rises and falls synchronously lasting one month

C. Normal ALT, AFP > 200 μg/L lasting one month

D. ALT rises and falls synchronously lasting two months

E. Normal ALT, AFP > 200 μg/L lasting 8 months

22. Which of the following helps differentiate liver cancer from benign active liver disease?

A. Significant impairment of liver function

B. HBsAg positive

C. AFP negative

D. AFP positive

E. Dynamic curves of AFP and ALT

23. The first-line diagnostic method for liver cancer is:

A. Hepatic angiography

B. B-ultrasound

C. MRI

D. CT

E. B-ultrasound + AFP quantitative determination

24. The preferred and most effective method for primary liver cancer is:

A. Traditional Chinese Medicine treatment

B. Chemical anticancer drug treatment

C. Radiotherapy

D. Surgical resection treatment

E. Biological and immunotherapy

25. The situation where radical hepatectomy is not feasible is:

A. Liver function abnormality classified as Child C

B. Solitary micro liver cancer

C. Solitary large liver cancer growing extrahepatically, with smooth surface, clear boundaries, and more than 70% normal liver tissue

D. Solitary small liver cancer

E. Fewer than 3 multiple nodules localized in one liver lobe

26. The most common site for pancreatic cancer is:

A. Whole pancreas

B. Pancreatic head

C. Pancreatic tail

D. Pancreatic body

E. Ectopic pancreas

27. The most common initial symptom of pancreatic cancer is:

A. Weight loss and fatigue

B. Epigastric pain and epigastric fullness/discomfort

C. Digestive tract symptoms

D. Jaundice

E. Fever

28. The preferred non-invasive examination method for pancreatic cancer is:

A. MRI

B. Ultrasound examination

C. CT

D. X-ray barium meal

E. Tumor marker CA199

29. The recommended radiotherapy dose (DT) for concurrent chemoradiotherapy in pancreatic cancer is:

A. 66Gy/33F/7W

B. 60Gy/30F/6W

C. 40Gy/20F/4W

D. 50Gy/25F/5W

E. 44Gy/22F/5W

30. The first-line standard treatment drug for advanced pancreatic cancer recommended by NCCN guidelines is:

A. Docetaxel

B. Gemcitabine

C. Paclitaxel

D. Vinorelbine

E. Pemetrexed

31. Male patient, 58 years old, dysphagia for more than 2 months, X-ray image as follows. The most likely diagnosis is:

A. Chronic esophagitis

B. Esophageal cancer

C. Esophageal stromal tumor

D. Esophageal varices

E. Esophageal tuberculosis

32. Male patient, 69 years old, progressive abdominal pain and bloating for over a month, X-ray image as follows. The most likely diagnosis is:

A. Gastric stromal tumor

B. Chronic gastritis

C. Duodenal cancer

D. Gastric ulcer

E. Gastric cancer

33. Female patient, 65 years old, recurrent abdominal pain and diarrhea for over 3 months, X-ray image as follows. The most likely diagnosis is:

A. Intestinal obstruction

B. Intestinal tuberculosis

C. Colonic polyp

D. Colon cancer

E. Segmental enteritis (Crohn's disease)

34. Male patient, 49 years old, recurrent abdominal pain and progressive jaundice for over a month, CT image as follows. The most likely diagnosis is:

A. Renal cancer

B. Cholangiocarcinoma

C. Pancreatic head cancer

D. Liver cancer

E. Lymphoma

35. Male patient, 49 years old, history of Hepatitis B for over ten years, recurrent abdominal pain, bloating and jaundice for over a month, CT image as follows. The most likely diagnosis is:

A. Hepatic sarcoma

B. Hepatic hemangioma

C. Liver cancer

D. Regenerative nodule of liver

E. Hepatic adenoma

36. Male patient, 54 years old, liver space-occupying lesion found during physical examination, MR image as follows. The most likely diagnosis is:

A. Liver cancer

B. Hepatic sarcoma

C. Hepatic hemangioma

D. Hepatic adenoma

E. Regenerative nodule of liver

37. Male, 62 years old, sensation of choking/obstruction while swallowing for 2 months, gradual weight loss, clinically highly suspected of esophageal cancer. The method for confirmed diagnosis is:

A. Supraclavicular lymph node biopsy

B. CT

C. Chest MRI examination

D. Esophageal barium swallow X-ray examination

E. Esophagoscopy and tissue biopsy

38. Male, 63 years old, progressive dysphagia for 3 months, hoarseness for the past week. The suitable examination for the patient is:

A. Barium swallow + Esophagoscopy + Indirect laryngoscopy

B. Chest X-ray + ECG + Chest CT

C. Esophagoscopy + Chest MRI + Fiberoptic bronchoscopy

D. Abdominal B-ultrasound + Sputum cytology + Indirect laryngoscopy

E. CEA + Isotope 32P scan + Gastric fluid analysis

39. Male, 60 years old, dysphagia for 3 months, esophagoscopy reported mid-esophageal squamous cell carcinoma, lesion length about 3.5cm, no obvious external invasion, no distant metastasis. The most appropriate management is:

A. Subtotal esophagectomy with gastric interposition

B. Subtotal esophagectomy with jejunal interposition

C. Subtotal esophagectomy with colonic interposition

D. Resection of esophageal lesion with end-to-end anastomosis

E. Radiotherapy + Anticancer drug therapy

40. Male, 50 years old, history of alcohol and tobacco use, retrosternal burning pain while swallowing for the past 3 weeks, outpatient barium swallow showed no obvious abnormalities. To further clarify the diagnosis, the examination to be performed is:

A. Fecal occult blood test

B. Esophagoscopy or esophageal balloon cytology

C. Chest CT

D. Chest X-ray

E. Continue observation

41. Male, 42 years old, epigastric burning pain for half a year, tarry stools for the past week. To confirm the diagnosis, the preferred examination method is:

A. B-mode ultrasound examination

B. X-ray gastrointestinal barium meal fluoroscopy

C. Fecal occult blood test

D. X-ray barium enema fluoroscopy

E. Gastroscopy

42. Male, 48 years old, during exploratory laparotomy for gastric cancer, the mass was found to have invaded the transverse colon but was relatively localized. This patient should:

A. Undergo total gastrectomy

B. Surgery is contraindicated

C. Undergo subtotal gastrectomy

D. Undergo radical gastrectomy + transverse colectomy

E. Undergo transverse colectomy

43. Female, 40 years old, vague epigastric pain and discomfort, exacerbated over the past 2 months, somewhat relieved by stomach tablets, appetite fair, fecal occult blood test (++), gastrointestinal barium meal exam showed disordered mucosal texture on the lesser curvature of the antrum, stiff and irregular gastric wall. The first diagnosis to consider is:

A. Gastric mucosal prolapse

B. Chronic antral gastritis

C. Gastric cancer

D. Gastric ulcer

E. Atrophic gastritis

44. Male, 28 years old, alternating diarrhea and constipation for the past half year, vague abdominal pain for the past 3 months, passing fresh blood in stool for the past 2 days. No mass found on abdominal palpation and digital rectal examination. Barium enema showed stiffness of the descending colon wall and visible filling defect. The diagnosis should be:

A. Ulcerative colitis (proliferative type)

B. Sigmoid colon cancer

C. Intestinal tuberculosis

D. Rectal ampulla cancer

E. Descending colon cancer

45. Male, 72 years old, presented due to fatigue and weight loss for nearly a year. Physical exam: anemic appearance, emaciated, a 4cm x 3cm mass palpable in the right lower abdomen, clear boundaries, hard, no obvious tenderness. Fiberoptic colonoscopy indicated cecal cancer. Radical right hemicolectomy for this patient should include the following areas, except:

A. Left half of the transverse colon

B. Right half of the transverse colon

C. Cecum

D. Ascending colon

E. Terminal ileum approx. 15-20cm long

46. Male, 60 years old, presented due to vague lower abdominal pain for over a month, low-grade fever, anorexia, diarrhea, history of tuberculosis. Physical exam: anemic appearance, palpable mass in the right lower abdomen, obvious tenderness. Laboratory tests: WBC 10x10⁹/L, N 0.75, ESR 25mm/h. Abdominal B-ultrasound showed right lower abdominal mass with liquid dark area. Barium enema showed filling defect in the cecum, mucosal destruction, appendix not filled. The diagnosis should be:

A. Cecal diverticulitis

B. Periappendiceal abscess

C. Ileocecal tumor

D. Ileocecal tuberculosis

E. Crohn's disease

47. Male, 36 years old, AFP elevated > 500 μg/L found during unit health check-up, normal liver function, HBsAg (+), HBeAg (+), HBcAb (+). The most likely diagnosis is:

A. Stage II liver cancer

B. Gonadal embryonal tumor

C. Late stage liver cirrhosis

D. Chronic active hepatitis

E. Subclinical liver cancer

48. Male, 40 years old, history of chronic hepatitis for 15 years, abdominal distension and weight loss recently. Physical examination: scleral icterus, fist-sized hard mass with uneven surface palpable in the upper abdomen. The most likely diagnosis is:

A. Liver abscess

B. Primary liver cancer

C. Chronic hepatitis

D. Metastatic liver cancer

E. Liver cirrhosis

49. Male, 38 years old, HBsAg (+) for 20 years, recently fatigued, liver discomfort. B-ultrasound showed a 3cm x 3cm substantial dark area with acoustic halo in the right lobe of the liver. The most likely diagnosis is:

A. Hepatic cyst

B. Liver cirrhosis (nodular)

C. Metastatic liver cancer

D. Primary liver cancer

E. Hepatic hemangioma

50. 45 years old, male, right rib pain for 3 months, slight fever, mild scleral icterus, liver 1.0cm below costal margin on inspiration, medium texture, elevation of the lateral side of the right diaphragm, B-ultrasound showed unequal nodular echoes in the liver, irregular edges, HBsAg (+), alpha-fetoprotein 100 μg/L. The most likely diagnosis is:

A. Primary liver cancer

B. Hepatitis B

C. Amoebic liver abscess

D. Liver cirrhosis

E. Cholestatic hepatitis

51. Female, 50 years old, jaundice, B-ultrasound showed intrahepatic bile duct diameter about 1cm. The further examination to choose is:

A. Hypotonic duodenography

B. Intravenous cholangiography

C. Percutaneous transhepatic cholangiography (PTC)

D. Radionuclide scan

E. Celiac angiography

52. Male, 65 years old, progressive jaundice for 3 months, accompanied by persistent distension in the middle and upper abdomen, aggravated when lying flat at night, significant weight loss. Physical exam: chronic wasting face. Skin and scleral icterus. Flat abdomen, deep tenderness in the upper right of the umbilicus, no mass palpable. Courvoisier sign positive. The first diagnosis to consider is:

A. Gastric cancer

B. Chronic cholecystitis

C. Primary liver cancer

D. Cholelithiasis

E. Pancreatic head cancer

53. Male, 35 years old, jaundice for 1 month, slight distending pain in the right upper abdomen, loss of appetite, ineffective medical treatment. Physical exam: hepatomegaly, gallbladder enlargement, serum bilirubin 17 μmol/L, AST 70 U/L, AKP 45 U/L, AFP > 5 ng/ml. The possible diagnosis is:

A. Late stage liver cirrhosis

B. Icteric hepatitis

C. Liver cancer

D. Common bile duct stone obstruction

E. Periampullary carcinoma

54. Male, 53 years old. Severe abdominal pain, jaundice. B-ultrasound showed: uniform intrahepatic echoes, clear vascular texture, enhanced intrahepatic duct wall echoes, 50mm x 32mm irregular hypoechoic mass at the pancreatic head, unclear boundaries, pancreatic duct 4mm. Diagnosis considered is:

A. Gallbladder cancer

B. Liver cancer

C. Pancreatic duct dilation

D. Pancreatic head cancer

E. Bile duct stones

55. Male, 40 years old, persistent jaundice for 9 months, accompanied by skin pruritus. Physical exam: temperature 39°C, liver 5cm below ribs, medium hardness, slightly uneven surface, tenderness (+), alpha-fetoprotein (-). To clarify the diagnosis, the most valuable examination among the following is:

A. Radionuclide scan

B. White blood cell count and differential

C. Chest fluoroscopy

D. Serum gamma-glutamyl transpeptidase determination

E. Abdominal B-ultrasound examination

56. Female, 60 years old, abdominal mass discovered half a month ago. Physical exam: abdomen soft, mass 7cm x 6cm x 6cm palpable, mobile, no tenderness. Abdominal B-ultrasound showed: solid nodule in abdominal cavity, signs of fusion between nodules. Immunohistochemical staining showed tumor cells: CD117+, CD34+, SMA-, Desmin-, S-100+. Diagnosis considered:

A. Sarcoma

B. Intestinal tumor

C. Leiomyoma

D. Gastrointestinal stromal tumor (GIST)

E. Melanoma

## Questions with Shared Clinical Vignettes

**[Case 1] Male patient, 48 years old, came for consultation due to a sensation of choking/obstruction while swallowing for half a year, currently can only ingest semi-liquid food. Physical exam: slightly emaciated, no enlarged lymph nodes palpable in the supraclavicular fossa. Esophageal barium swallow X-ray showed a 4cm long localized stiffness of the vessel wall in the middle and lower segment of the esophagus, mucosal interruption in parts, barium could still pass through.**

57. The diagnosis to consider first is:

A. Achalasia

B. Esophagitis

C. Benign esophageal tumor

D. Esophageal cancer

E. Esophageal diverticulum

58. After confirmed diagnosis, the treatment measure to choose is:

A. Immunotherapy

B. Surgical treatment

C. Chemotherapy

D. Radiotherapy

E. Traditional Chinese Medicine treatment

59. The further examination should be:

A. Thoracoscopy

B. Chest CT

C. Esophagoscopy and tissue biopsy

D. Chest MRI

E. Mediastinoscopy

**[Case 2] Male patient, 71 years old, abdominal distending pain for 3 days, accompanied by acid reflux, belching, decreased appetite. Physical exam: mild tenderness under the xiphoid process, no rebound tenderness, liver and spleen not palpable under ribs, shifting dullness negative. Gastroscopy showed a huge ulcer in the gastric antrum.**

60. If HP test is negative, the next treatment is:

A. Chemotherapy + Radiotherapy

B. Surgery

C. Radiotherapy

D. Chemotherapy

E. Anti-HP therapy

61. If HP test is positive, the next treatment is:

A. Chemotherapy + Radiotherapy

B. Surgery

C. Radiotherapy

D. Chemotherapy

E. Anti-HP therapy

62. Which of the following examinations is most valuable for diagnosis:

A. Abdominal X-ray

B. CT

C. MRI

D. B-ultrasound

E. Gastric tissue biopsy

63. If gastroscopy biopsy results indicate gastric mucosa-associated lymphoid tissue (MALT) lymphoma, the next step should be:

A. Chest plain film

B. HP test

C. Abdominal CT

D. Bone marrow aspiration

E. Colonoscopy

**[Case 3] Male patient, 60 years old, history of gastric ulcer for 8 years, abdominal distension, loss of appetite, significant weight loss, persistently positive fecal occult blood for the past 3 months, ineffective treatment of gastric pain with antacids.**

64. According to the 2008 NCCN guidelines, this patient has indications for concurrent chemoradiotherapy. The guideline-recommended concurrent chemotherapy drug is:

A. 5-Fu

B. Oxaliplatin

C. Paclitaxel

D. Cisplatin

E. VP-16

65. The preferred treatment method is:

A. Vagotomy

B. Subtotal gastrectomy

C. Total gastrectomy

D. Radical gastrectomy

E. Administer mucosal protective agents and continue medical treatment

66. The most likely diagnosis for this patient is:

A. Gastrinoma

B. Compound ulcer

C. Intractable ulcer

D. Penetrating gastric ulcer

E. Malignant transformation of gastric ulcer

67. The preferred examination method is:

A. Abdominal plain X-ray

B. CT scan

C. MRI examination

D. B-ultrasound

E. Fiberoptic gastroscopy biopsy

68. The postoperative pathology report of the patient is poorly differentiated adenocarcinoma of the gastric antrum, penetrating the serosal layer, not involving surrounding tissues, with 8/17 lymph node metastases. According to the 2002 AJCC staging, the patient's postoperative pathological stage is:

A. T2N2M0

B. T2N1M0

C. T3N2M0

D. T3N4M0

E. T3N1M0

69. The recommended dose for postoperative radiotherapy for this patient is:

A. 60Gy

B. 45Gy

C. 30Gy

D. 55Gy

E. 56Gy

**[Case 4] Male patient, 58 years old, progressive anemia, emaciation, fatigue for half a year, occasional right abdominal vague pain, no diarrhea. Physical exam: mass palpable in the right middle abdomen, active bowel sounds.**

70. If surgical treatment is required, which of the following preoperative preparations is most important:

A. Liver and kidney function tests

B. Correct nutrition

C. Cardiopulmonary function tests

D. Bowel preparation

E. Psychological preparation

71. Which of the following examinations can clarify the diagnosis:

A. B-ultrasound

B. Fiberoptic colonoscopy

C. CT

D. CEA

E. X-ray barium meal examination

72. If B-ultrasound reveals multiple liver metastases, the patient's general condition is acceptable, PS score is 1, liver function is normal, the treatment at this time should be:

A. Interventional therapy

B. Radiotherapy

C. Chemotherapy

D. Surgery

E. Traditional Chinese Medicine

**[Case 5] Female patient, 65 years old, skin and scleral icterus for 30 days, accompanied by skin pruritus, anorexia, fatigue, weight loss, dark urine, pale stool, etc.; no special past history. Physical exam: skin and scleral icterus, no tenderness or rebound tenderness in abdomen, no percussion pain in liver area, palpable enlarged gallbladder, negative for ascites. Laboratory tests: TBil: 25mg/dl, DBil: 20mg/dl, CA199: 12u/ml, CA50: 8u/ml; Imaging: B-ultrasound: low-level biliary obstruction, intrahepatic and extrahepatic bile duct dilation, pancreatic head not enlarged; CT: low-level biliary obstruction, intrahepatic and extrahepatic bile duct dilation, pancreatic duct dilation (double duct sign), no obvious mass in pancreas; Hypotonic duodenography: duodenal curve not enlarged, disordered mucosa in descending part, no definite filling defect.**

73. Chest CT indicated extensive bilateral lung metastases. After ERCP, blood bilirubin dropped to near normal. Regarding the choice of subsequent treatment, which is reasonable:

A. The general treatment principle for this patient is palliative care, mainly local treatment

B. Radiotherapy only to the ampulla

C. If the patient's general condition is poor (PS > 2), molecular targeted therapy can be considered, preferably Iressa

D. If the patient's general condition is acceptable, systemic chemotherapy should be the mainstay, combined with local palliative radiotherapy

E. The general treatment principle for this patient is to maximize tumor killing and strive for PR or CR

74. If diagnosis is confirmed and distant metastasis is excluded, the most aggressive treatment method is:

A. Common bile duct resection

B. Pancreaticoduodenectomy (Whipple procedure)

C. Cholecystectomy

D. Exploratory laparotomy

E. Radical hepatectomy

75. Which of the following examination items is most valuable for helping to clarify the diagnosis:

A. ERCP

B. Abdominal plain film

C. MRI

D. Stool routine + Occult blood

E. PET

76. If this patient undergoes surgical treatment, a possible postoperative complication is:

A. Hepatitis

B. Steatorrhea

C. Pancreatic fistula

D. Intestinal obstruction

E. Common bile duct stenosis

77. The preliminary diagnosis for this patient is:

A. Common bile duct cancer

B. Ampullary cancer

C. Gallbladder cancer

D. Liver cancer

E. Duodenal cancer

**[Case 6] Male patient, 44 years old, right upper quadrant pain for half a year, aggravated with upper abdominal mass for one month, accompanied by bloating, anorexia, nausea, no vomiting or diarrhea, normal urination and defecation, weight loss of 5kg. History of Hepatitis B for many years. Physical exam: T 36.7°C, P 78 bpm, R 18 bpm, BP 110/70 mmHg, no skin jaundice, mild scleral icterus. Abdomen flat and soft, right upper quadrant full, tenderness in right upper quadrant, no muscle guarding, hepatomegaly 5cm below ribs, blunt edge, tough texture, tender to touch, spleen not palpable, tympany on abdominal percussion, no shifting dullness, upper liver border percussion at 5th intercostal space, liver area percussion pain, bowel sounds 8 times/min. Auxiliary exams: Hb 89g/L, WBC 5.6x10⁹/L, ALT 84U/L, AST 78U/L, TBIL 30μmol/L, DBIL 10μmol/L, ALP 188U/L, GGT 64U/L, AFP 880ng/ml, CEA 24mg/ml. B-ultrasound: solid space-occupying lesion in right liver lobe 8cm, no dilation of intra/extrahepatic bile ducts.**

78. If distant metastasis is excluded, the most aggressive treatment means is:

A. Radical hepatectomy

B. Chemotherapy

C. Interventional therapy

D. Radiotherapy

E. Radiofrequency ablation

79. Which of the following is the preliminary diagnosis for this patient?

A. Hepatic hemangioma

B. Primary liver cancer

C. Pancreatic cancer

D. Metastatic liver cancer

E. Hepatic cyst

80. If the histological type of this patient needs to be clarified, the examination item that must be performed is:

A. Gastrointestinal series

B. CT

C. Liver needle biopsy

D. MRI

E. ERCP

81. If a 3cm mass in the left liver lobe is found during postoperative follow-up, AFP continues to rise, no distant metastasis, liver function is basically normal, and the patient refuses surgery, the most appropriate treatment is:

A. Whole liver irradiation

B. Chemotherapy

C. Traditional Chinese Medicine treatment

D. X-knife treatment (Stereotactic Radiotherapy) for left liver lobe mass

E. Interferon

## Questions with Shared Options

[Options 1]

A. AFP > 200μg/L lasting 8 weeks

B. AFP < 20μg/L

C. AFP > 200μg/L lasting 6 weeks

D. AFP > 100μg/L

E. AFP > 500μg/L lasting more than 4 weeks

**82. Diagnostic of hepatocellular carcinoma:**

**83. Cholangiocarcinoma:**

[Options 2]

A. Intrahepatic bile ducts not dilated, hepatic duct dilated, gallbladder empty

B. Intrahepatic bile ducts dilated, hepatic duct dilated, gallbladder empty

C. Intrahepatic and extrahepatic bile ducts dilated, gallbladder enlarged, pancreatic duct dilated (double duct sign)

D. Intrahepatic and extrahepatic bile ducts dilated, gallbladder enlarged

E. Intrahepatic bile ducts not dilated, hepatic duct not dilated, gallbladder empty

**84. Obstruction site is in the common bile duct:**

**85. Obstruction site is in the common hepatic duct or above:**

**86. Obstruction site is in the ampulla:**

[Options 3]

A. Chemotherapy

B. Esophagectomy + Esophagogastrostomy

C. Radiotherapy

D. Gastrostomy

E. Traditional Chinese Medicine treatment

**87. Male, 50 years old, dysphagia after eating for more than 3 months, currently cannot ingest liquid food, gradually emaciated. Physical exam: general condition poor, a hard lymph node the size of a thumb palpable in the left supraclavicular fossa. Esophagoscopy biopsy reported squamous cell carcinoma grade III. Should undergo:**

**88. Male, 50 years old, dysphagia after eating for more than 3 months. Esophageal barium swallow X-ray showed a 3cm long annular stenosis in the middle segment of the esophagus. Esophagoscopy biopsy reported squamous cell carcinoma grade II. No enlarged supraclavicular lymph nodes, no hoarseness, chest X-ray normal. Should undergo:**

[Options 4]

A. Shortness of breath, palpitations, hemoptysis

B. Progressive dysphagia, more common in those over 40 years old

C. Hoarseness accompanied by dysphagia

D. Intermittent dysphagia, more common in young adults

E. Periodic paralysis of limbs

**89. Symptoms appearing in late stage esophageal cancer:**

**90. Symptoms appearing in middle stage esophageal cancer:**

[Options 5]

A. Total gastrectomy

B. Resection of lower esophagus and proximal stomach

C. Radical gastrectomy

D. Gastrojejunostomy

E. Abandon radical surgery

**91. Exploratory laparotomy reveals widespread metastasis of gastric cancer, should perform:**

**92. Late stage gastric cancer with pyloric obstruction should perform:**

**93. Gastric cancer of the lesser curvature of the body invading the fundus should perform:**

**94. High gastric cancer of the lesser curvature and cardia cancer should perform:**

# Chapter 5: Breast Cancer (N=19)

## Standard Multiple-Choice Questions

1. Breast cancer primarily occurs in women. Male breast cancer accounts for approximately:

A. 2%

B. 0.5%

C. 1.5%

D. 1%

E. 2.5%

2. Which of the following statements regarding risk factors for breast cancer is incorrect?

A. Long duration of breastfeeding

B. Early age of menarche

C. Early menopause

D. Early age of first full-term pregnancy

E. Family history of breast cancer

3. The most common site of metastasis for breast cancer is:

A. Brain

B. Bone

C. Lung

D. Liver

E. Adrenal gland

4. The efficacy of Tamoxifen (TAM) is related to which of the following factors?

A. Lymph node status

B. Her2 expression status

C. Tumor size

D. ER status

E. Menopausal status

5. Which of the following is not an absolute contraindication for radiotherapy after breast-conserving surgery?

A. Persistently positive tumor margins

B. Two or more tumors in different quadrants

C. Large breasts

D. Previous history of radiotherapy to the breast region

E. Pregnant women

6. The proportion of breast cancers with Her-2/neu overexpression is:

A. More than 40%

B. 10%

C. 20%–30%

D. 10%–20%

E. More than 50%

7. Which of the following is not an indication for breast-conserving surgery?

A. Tumor located in the areola region

B. Primary tumor smaller than 4cm

C. Focal microcalcifications

D. Multiple tumors but limited to one quadrant

E. No collagen vascular disease

8. The radiotherapy dose for the supraclavicular/apical axillary field after radical mastectomy is:

A. 60 Gy / 6 weeks

B. 40 Gy / 4 weeks

C. 56 Gy / 5–6 weeks

D. 50 Gy / 5 weeks

E. None of the above

9. Indications for adjuvant chemotherapy in breast cancer exclude:

A. ER-/PR-

B. N+

C. T > 1cm / SBR Grade III

D. N-

E. Strong positive Her-2/neu

10. The response rate of Xeloda (Capecitabine) for metastatic breast cancer resistant to Doxorubicin and Paclitaxel is:

A. 30%

B. < 10%

C. 20%

D. 15%

E. 35%

11. Which description regarding neoadjuvant chemotherapy is incorrect?

A. Can reduce distant metastasis

B. Can eliminate micrometastases

C. Clinical and pathological response after chemotherapy can help in the selection of postoperative treatment regimens

D. Can shrink the tumor, facilitating surgery

E. For patients, apart from the side effects of chemotherapy drugs, there are no other risks

12. Female patient, 67 years old, discovered a breast mass for over a month, accompanied by nipple discharge. The X-ray image is shown below. The most likely diagnosis is:

A. Breast cyst

B. Breast hyperplasia

C. Breast fibroma

D. Breast cancer

E. Mastitis

13. Female patient, 39 years old, underwent radical mastectomy for breast cancer 4 years ago. Currently presents with left hip pain. X-ray shows bone destruction in the left hip bone and femoral head. Endocrine therapy is not the first choice in which of the following situations?

A. Presence of bone and soft tissue metastases

B. Only ER positive

C. Only PR positive

D. DFS (Disease-Free Survival) > 2 years after adjuvant therapy

E. Both ER and PR negative

14. Female, 42 years old, 4cm × 3cm mass in the upper outer quadrant of the left breast, multiple matted enlarged lymph nodes in the ipsilateral axilla. Pathology results indicate left breast invasive ductal carcinoma. Among 13 axillary lymph nodes, 4 are positive. ER++, PR-, Her-2/neu-. The clinical stage of this patient is considered to be:

A. Stage IIIA

B. Stage I

C. Stage IIB

D. Stage IIA

E. Stage IIIB

## Questions with Shared Clinical Vignettes

**[Case] Female patient, 37 years old, 4cm × 3cm mass in the upper outer quadrant of the left breast, 5cm from the nipple, mobile, but mobility significantly restricted when the patient places hands on hips. No enlarged lymph nodes palpable in the left axilla.**

15. Three years after radical mastectomy, the patient developed headache and nausea. MRI indicated multiple brain metastases. The preferred treatment at this time is:

A. Endocrine therapy

B. Surgical treatment

C. Radiotherapy

D. Chemotherapy

E. Herceptin therapy

16. If the pathology result indicated left breast invasive lobular carcinoma, and 2 out of 15 axillary lymph nodes were positive, ER(++), PR(-), Her-2/neu(+++), the clinical stage of this patient would be considered:

A. Stage IIIA

B. Stage I

C. Stage IIB

D. Stage IIA

E. Stage IIIB

17. Which of the following is the best method for qualitative diagnosis in this patient?

A. Near-infrared scanning

B. Core needle biopsy

C. Incisional biopsy

D. Mammography (Molybdenum target X-ray)

E. Fine needle aspiration cytology

## Questions with Shared Options

[Options]

A. 50%

B. Less than 10%

C. 30%

D. 20%

E. 60%–70%

**18. For breast cancer patients who are ER or PR positive, the response rate to endocrine therapy is:**

**19. For breast cancer patients who are both ER and PR negative, the response rate to endocrine therapy is:**

# Chapter 6: Urinary and Male Reproductive System Tumors (N=70)

## Standard Multiple-Choice Questions

1. According to statistics, the incidence of renal cancer accounts for what percentage of systemic malignancies?

A. 4%

B. 1%

C. 3%

D. 2%

E. 5%

2. In China, the most common tumor of the urinary system is:

A. Testicular cancer

B. Renal cancer

C. Prostate cancer

D. Bladder cancer

E. Renal pelvic cancer

3. The most common pathological type of malignant bladder tumor is:

A. Squamous cell carcinoma

B. Urothelial carcinoma

C. Mucinous adenocarcinoma

D. Adenocarcinoma

E. Clear cell carcinoma

4. The most common initial symptom of bladder cancer is:

A. Painless hematuria

B. Urinary tract irritation symptoms

C. Lower abdominal mass

D. Dysuria

E. Lower abdominal mass

5. Which of the following is correct regarding the epidemiological statistics of renal cancer?

A. Unrelated to high fat intake

B. Incidence is highest in the Americas

C. Peak age is mostly 50–60 years old

D. Rural areas higher than urban areas

E. Accounts for 10% of systemic malignancies

6. Among the following diseases, which has the best prognosis when the clinical stage is Stage II~III?

A. Testicular seminoma

B. Liver cancer

C. Esophageal cancer

D. Gastric cancer

E. Lung cancer

7. The most common pathological type of testicular tumor is:

A. Yolk sac tumor

B. Teratoma

C. Seminoma

D. Embryonal carcinoma

E. Choriocarcinoma

8. The most common site of metastasis for advanced testicular tumors is:

A. Bone

B. Liver

C. Lung

D. Kidney

E. Brain

9. The preferred treatment method for Stage I testicular tumor is:

A. Molecular targeted therapy

B. Surgery

C. Chemotherapy

D. Radiotherapy

E. Endocrine therapy

10. The radiotherapy target area for Stage I testicular seminoma patients after surgery is:

A. Mediastinal lymph nodes

B. Contralateral testis

C. Para-aortic lymph nodes

D. Inguinal lymph nodes

E. Iliac vascular lymph nodes

11. The upper border of the para-aortic irradiation field for testicular tumors is:

A. Lower edge of T11

B. Lower edge of T8

C. Lower edge of T10

D. Lower edge of T9

E. Lower edge of T12

12. The lower border of the para-aortic irradiation field for testicular tumors is:

A. Lower edge of L4

B. Lower edge of L1

C. Lower edge of L3

D. Lower edge of L2

E. Lower edge of L5

13. The region with the highest incidence of prostate cancer is:

A. Southeast Asia

B. China

C. Africa

D. North America

E. Australia

14. Which of the following is the best initial screening method for prostate cancer?

A. Prostate biopsy and serum PSA testing

B. CT and MRI

C. Digital rectal examination (DRE) and serum PSA testing

D. X-ray examination and prostate biopsy

E. PET-CT

15. Which of the following diseases most frequently causes bone metastases?

A. Cervical cancer

B. Gastric cancer

C. Esophageal cancer

D. Prostate cancer

E. Gastrointestinal stromal tumor (GIST)

16. The appropriate position for external beam radiotherapy for prostate cancer is:

A. Lateral decubitus position

B. Supine position

C. Lithotomy position

D. Prone position

E. Sitting position

17. Which of the following is the standard chemotherapy regimen for hormone-refractory prostate cancer?

A. Estramustine-containing regimen

B. Mitoxantrone-containing regimen

C. Docetaxel-containing regimen

D. Doxorubicin-containing regimen

E. None of the above

18. When prostate cancer metastasizes to bone, which type is most common?

A. Pathological fracture

B. Osteolytic type

C. Mixed type

D. Osteoblastic type

E. Myelofibrosis

19. Male patient, 74 years old, progressive dysuria for 4 months. MR image is shown below. The most likely diagnosis is:

A. Bladder cancer

B. Benign prostatic hyperplasia

C. Prostate sarcoma

D. Prostate cancer

E. Chronic prostatitis

20. Male patient, 66 years old, recurrent painless gross hematuria for 3 weeks. CT image is shown below. The most likely diagnosis is:

A. Renal pelvic carcinoma

B. Renal cyst

C. Renal cell carcinoma

D. Renal angiomyolipoma

E. Renal abscess

21. Male patient, 76 years old, recurrent painless gross hematuria for 3 months. MR image is shown below. The most likely diagnosis is:

A. Bladder cancer

B. Prostate cancer

C. Bladder polyp

D. Cystitis

E. Seminal vesicle adenocarcinoma

22. Male patient, 55 years old, recurrent fever, gross hematuria accompanied by back pain. Physical exam: negative renal percussion tenderness, no mass palpable. Urinalysis: RBC 2+. Renal ultrasound and CT indicated: left renal solid mass. The most likely diagnosis is:

A. Renal pelvic carcinoma

B. Renal cell carcinoma

C. Nephroblastoma (Wilms tumor)

D. Renal cyst

E. Renal metastasis

23. Male patient, 45 years old, due to "recurrent gross hematuria, left renal area space-occupying lesion found", underwent "radical nephrectomy + lymph node dissection" under general anesthesia after completing preoperative examinations. Intraoperatively, the tumor size was about 3cm × 5cm, invading the adrenal gland, renal hilar lymph nodes 1/3 positive. Postoperative pathology indicated: renal clear cell carcinoma. The disease stage for this patient is:

A. T4N1M0

B. T1N0M0

C. T3N1M0

D. T2N1M0

E. T2N2M0

24. Female patient, 56 years old, presented with gross hematuria. Renal ultrasound and CT revealed a solid space-occupying lesion in the right kidney, size approx. 3cm, left kidney stones, and mild hydronephrosis. The suitable surgical method for this patient is:

A. Nephron-sparing surgery + lymph node dissection

B. Simple total nephrectomy

C. Radical nephrectomy

D. Nephron-sparing surgery

E. Partial nephrectomy

25. Male patient, 60 years old, painless total gross hematuria accompanied by urinary frequency. Urinary exfoliative cytology: cancer cells found. Cystoscopy showed: brown nodular mass in the bladder neck and trigone, surface ulcerated, edges raised and edematous. The most suitable surgical method for this patient is:

A. Partial cystectomy + pelvic lymph node dissection

B. Transurethral resection of bladder tumor (TURBT)

C. Radical cystectomy

D. Partial cystectomy

E. Cystotomy and tumor resection

26. Male patient, 50 years old, painless total gross hematuria. Cystoscopy showed: a single, pink, long-stalked mass on the bladder dome, resembling water weeds floating in water. The most suitable surgical method for this patient is:

A. Partial cystectomy + pelvic lymph node dissection

B. Transurethral resection of bladder tumor (TURBT)

C. Radical cystectomy

D. Partial cystectomy

E. Cystotomy and tumor resection

27. Male patient, 60 years old, progressive dysuria for one year, right back pain for the last month. Physical exam: superficial lymph nodes not enlarged, right renal area percussion tenderness (+), DRE palpable nodular, hard prostate. The detection indicator with significant meaning for qualitative diagnosis of the disease is:

A. Blood CEA

B. Blood BUN

C. Serum PSA

D. Blood Cr

E. Blood AFP

## Questions with Shared Clinical Vignettes

**[Case 1] Male patient, 60 years old, painless total gross hematuria accompanied by back pain, emaciation, weight loss. Physical exam: abdomen soft, no obvious mass palpable, renal percussion tenderness (-). Complete blood count WBC: 13×10⁹/L, neutrophils 85%, renal function Creatinine 60 μmol/L, BUN 6.1 mg/L, ESR 20 mm/h, Urinalysis: RBC (++), Urinary cytology (-).**

28. To aid diagnosis, the first examination to consider is:

A. Intravenous urography (IVP)

B. Cystoscopy

C. Abdominal CT

D. Urinary system ultrasound

E. PET

29. The most likely diagnosis for this patient is:

A. Nephroblastoma

B. Renal cell carcinoma

C. Bladder cancer

D. Renal pelvic carcinoma

E. Renal cyst

30. If this patient is prepared for surgical treatment, the scope of radical surgery should exclude:

A. Renal hilar lymph nodes

B. Gerota's fascia

C. Kidney and adrenal gland

D. Perirenal fat

E. Ureter

**[Case 2] Male patient, 45 years old, painless total gross hematuria accompanied by frequency, urgency, lower abdominal pain. Physical exam: abdomen soft, no obvious mass palpable, renal percussion tenderness (-). CBC WBC: 16×10⁹/L, neutrophils 90%, Creatinine 55 μmol/L, BUN 4.5 mg/L, ESR 5 mm/h, Urinalysis: RBC (+++), Urinary cytology (-).**

31. The current efficacy rate of chemotherapy for this disease should be:

A. 40%–50%

B. 10%–20%

C. 30%–40%

D. 20%–30%

E. 50%–60%

32. If the patient requires total cystectomy, indications for surgery do not include:

A. Multiple non-invasive bladder cancers

B. Invasive carcinoma located in the bladder neck or trigone

C. Huge invasive carcinoma

D. Squamous cell carcinoma of the bladder

E. High-grade tumor accompanied by carcinoma in situ

33. This patient can undergo the following examinations to clarify the diagnosis, except:

A. CT

B. Cystoscopy

C. Retrograde urography

D. Intravenous urography

E. Radionuclide renogram

**[Case 3] Male patient, 60 years old, admitted for "progressive dysuria for half a year". DRE palpable nodular, hard prostate. Serum PSA elevated. Prostate biopsy showed prostate cancer. Pelvic CT showed prostate cancer confined to the left lobe, no pelvic lymph node enlargement, other examinations revealed no abnormalities.**

34. The clinical stage of this patient belongs to:

A. T4N0M0

B. T1N0M0

C. T3N0M0

D. T2N0M0

E. T1N1M0

35. The suitable treatment for this patient is:

A. Chemotherapy

B. Observation

C. Prostatectomy

D. Castration

E. Transurethral resection of the prostate (TURP)

36. If the patient refuses the above treatments, the treatment to consider is:

A. Chemotherapy

B. Observation

C. Radical radiotherapy

D. Castration

E. Prostatectomy

**[Case 4] Male patient, 25 years old, experienced left testicular pain 1 month ago, and a palpable left testicular mass approx. 2cm × 1cm × 2cm. Superficial lymph nodes not palpable. underwent orchiectomy, pathology showed seminoma.**

37. Which practice is wrong when detecting tumor markers?

A. Need to detect PSA

B. Dynamic observation is beneficial for understanding treatment effect

C. Need to detect AFP

D. Need to detect β-hCG

E. Need to detect LDH

38. The 5-year survival rate for this type of patient is approximately:

A. 80%

B. 15%

C. 60%

D. 45%

E. 95%

39. Further examination revealed retroperitoneal lymph node enlargement approx. 3cm × 2cm × 2cm, no abnormalities in the thoracic cavity. According to the Royal Marsden Hospital staging, it is:

A. Stage IIC

B. Stage I

C. Stage IIB

D. Stage IIA

E. Stage III

40. The suitable postoperative adjuvant treatment is:

A. Observation

B. Chemotherapy

C. Endocrine therapy

D. Radiotherapy

E. Immunotherapy

**[Case 5] Male patient, 65 years old, painless gross hematuria. Renal ultrasound showed: left renal solid space-occupying lesion. Abdominal CT showed: a 2cm × 3cm low-density lesion near the cortex of the left kidney, no obvious enhancement, protruding outside the kidney; a soft tissue density nodule seen beside the abdominal aorta.**

41. The patient underwent surgery and pathology showed: clear cell carcinoma. Intraoperatively, the tumor size was 3cm × 3cm, perirenal adipose tissue involved, 1 para-aortic lymph node biopsy found cancer tissue. The clinical stage of this patient belongs to:

A. T3aN1M0

B. T1aN1M0

C. T2N1M0

D. T1bN1M0

E. T3bN1M0

42. The preferred next treatment strategy for this patient is:

A. Radical nephrectomy + Radiotherapy

B. Radical nephrectomy

C. Nephron-sparing surgery

D. Radical nephrectomy + Enlarged lymph node resection

E. Chemotherapy

43. If a solitary nodule in the right lung is found during preoperative examination, the best next treatment plan is:

A. Molecular targeted therapy

B. Radical nephrectomy + Resection of metastatic lesion

C. Immunotherapy

D. Systemic chemotherapy

E. Radiotherapy

**[Case 6] Male patient, 52 years old, painless gross hematuria accompanied by frequency and urgency. Urinalysis: WBC (+++), RBC (+++), Protein (-), Urinary cytology found cancer cells. Physical exam: renal percussion tenderness (-), bimanual examination revealed no obvious abnormalities.**

44. The next examination the patient needs to do is:

A. KUB (Kidney, Ureter, Bladder X-ray)

B. IVP

C. Cystoscopy

D. Urinary system B-scan

E. Abdominal and pelvic CT

45. The patient underwent surgical treatment. Intraoperatively, the tumor was found located at the bladder neck and trigone, brown nodular mass, surface ulcerated. Postoperative pathology showed: tumor invasion of deep muscle layer, lymph nodes (-). The patient's stage belongs to:

A. T3aN0M0

B. T1aN0M0

C. T2bN0M0

D. T2aN0M0

E. T3bN0M0

46. If this patient is prepared for radiotherapy, which of the following regarding radiotherapy is incorrect?

A. Local dose is 65 Gy

B. Upper border of field is located between L4 and L5

C. Lateral borders of field reach 1–2 cm outside the true pelvis

D. Lower border of field is located at the lower line of the obturator foramen

E. Pelvic field dose 40–50 Gy

**[Case 7] Male patient, 60 years old, admitted for "progressive dysuria for half a year, back pain for 1 month". DRE palpable nodular, hard prostate. Serum PSA elevated. Prostate biopsy showed prostate cancer. Pelvic CT showed prostate cancer invading the rectum, pelvic lymph node enlargement. Bone ECT showed radioactive concentration in multiple thoracic vertebral bodies. Other examinations revealed no abnormalities.**

47. The best examination to clarify the cause of back pain is:

A. Chest CT

B. Thoracic MRI

C. PET-CT

D. Thoracic CT

E. Any of the above

48. If multiple thoracic metastases are confirmed, the stage belongs to:

A. T4N1M1

B. T1N0M0

C. T3N0M1

D. T2N1M0

E. T3N1M1

49. Treatment that needs to be performed immediately is:

A. Endocrine therapy

B. Surgical internal fixation

C. Radiotherapy

D. Systemic chemotherapy

E. Concurrent chemoradiotherapy

**[Case 8] Male patient, 27 years old, discovered a testicular mass due to left testicular pain. Underwent orchiectomy, pathology showed non-seminoma. Further chest CT examination revealed right lung metastasis.**

50. The preferred chemotherapy regimen is:

A. CVP

B. BEP

C. GP

D. VIP

E. NP

51. Follow-up chest CT after chemotherapy showed residual lesions. The next treatment is:

A. Switch to VIP regimen chemotherapy

B. Continue original regimen chemotherapy

C. Local radiotherapy

D. Lobectomy

E. Observation

52. After achieving CR with the above treatment, recurrence in the lung was found again half a year later. The optional chemotherapy regimen is:

A. High-dose CBP + VP16

B. VIP

C. EP

D. BEP

E. BVP

## Questions with Shared Options

[Options 1]

A. Radical cystectomy

B. Transurethral resection of bladder tumor (TURBT)

C. Total cystectomy

D. Partial cystectomy

E. Cystotomy and tumor resection

**53. The surgical method chosen for tumors invading the bladder neck and trigone is:**

**54. The surgical method chosen for Ta superficial bladder tumors is:**

**55. The surgical method chosen for a single localized invasive tumor on the lateral wall, dome, or base of the bladder is:**

[Options 2]

A. Mass, pain, bladder irritation symptoms

B. Hematuria, pain, mass

C. Hematuria, mass

D. Hematuria, bladder irritation symptoms

E. Mass, bladder irritation symptoms

**56. Which of the above is the typical symptom of bladder cancer?**

**57. Which of the above is the triad of renal cancer?**

[Options 3]

A. Bladder capacity reduced, mucosa near tumor shrunken, thickened, edematous, congested

B. Single or multiple pink, long-stalked, resembling water weeds floating in water

C. Dark red invasive nodular mass, short stalk, surface covered with grayish-white necrotic tissue, tumor mobility small

D. Raised red area on bladder surface mucosa, appearance similar to congested and hyperplastic mucosa

E. Sessile, ill-defined brown mass, ulcerated at necrosis, edges edematous, with calcification

**58. Which of the above is the cystoscopic appearance of papillary carcinoma?**

**59. Which of the above is the cystoscopic appearance of carcinoma in situ?**

**60. Which of the above is the cystoscopic appearance of invasive carcinoma?**

**61. Which of the above is the cystoscopic appearance of T2 stage carcinoma?**

[Options 4]

A. 40–45 Gy

B. 20–30 Gy

C. 35–40 Gy

D. 30–35 Gy

E. 45–50 Gy

**62. Postoperative radiotherapy dose for Stage I testicular seminoma patients is:**

**63. Tolerance dose of the small intestine is:**

**64. Postoperative radiotherapy dose for Stage II testicular seminoma patients is:**

[Options 5]

A. CEA

B. BHCG

C. CA125

D. AFP

E. None of the above

**65. Not expressed in testicular non-seminoma patients but expressed in seminoma patients (Note: Actually refers to markers usually NOT elevated in pure seminoma vs non-seminoma, or specific patterns):**

**66. Expressed in both testicular seminoma and non-seminoma patients:**

**67. Not expressed in testicular seminoma patients but expressed in non-seminoma patients:**

[Options 6]

A. 6500–7000 cGy

B. 3500–4000 cGy

C. 5500–6000 cGy

D. 4500–5000 cGy

E. 7000–7600 cGy

**68. Rectal tolerance dose:**

**69. Bladder tolerance dose:**

**70. Radical radiotherapy dose for prostate cancer:**

# Chapter 7: Malignant Lymphoma (N=54)

## Standard Multiple-Choice Questions

1. Which of the following statements regarding the epidemiological characteristics of Hodgkin lymphoma in China is incorrect?

A. The age of onset presents a bimodal curve

B. The incidence rate is lower than in European and American countries

C. The age of onset curve presents a single peak

D. Incidence is higher in males than in females

E. Incidence gradually increases with age

2. Burkitt lymphoma is associated with which of the following infections?

A. HP (Helicobacter pylori)

B. HTLV-1

C. EBV (Epstein-Barr Virus)

D. HHV-8

E. Chlamydia

3. Which of the following does not belong to classic Hodgkin lymphoma?

A. Lymphocyte-rich classic Hodgkin lymphoma

B. Nodular sclerosis

C. Nodular lymphocyte-predominant Hodgkin lymphoma (NLPHL)

D. Lymphocyte-depleted

E. Mixed cellularity

4. The characteristics of Reed-Sternberg (RS) cells in lymphocyte-rich classic Hodgkin lymphoma are:

A. Surrounding lymphocytes are reactive T lymphocytes

B. CD30+

C. CD20-

D. CD15+

E. All of the above are correct

5. In which of the following situations should a lymphoma patient undergo cerebrospinal fluid (CSF) examination?

A. Testicular involvement

B. Stage IV patients

C. Central nervous system involvement

D. Bone marrow invasion

E. All of the above are correct

6. Which of the following is a correct poor prognostic factor for Hodgkin lymphoma?

A. Serum albumin > 40g/L

B. Age ≤ 45 years

C. Hb < 105g/L

D. Female

E. WBC ≥ 10×10⁹/L

7. A Non-Hodgkin Lymphoma International Prognostic Index (IPI) score of 3 corresponds to:

A. High-risk group

B. Low-risk group

C. High-intermediate risk group

D. Low-intermediate risk group

E. Very high-risk group

8. Which of the following is correct regarding B symptoms in lymphoma (AJCC 6th Edition)?

A. Weight loss < 10%

B. Pruritus

C. Fatigue

D. Sweating

E. Unexplained fever with temperature exceeding 38°C

9. The optimal treatment for early-stage favorable Hodgkin lymphoma is:

A. Combination chemotherapy + Involved Field Radiotherapy (IFRT)

B. Radiotherapy alone

C. Surgery

D. Chemotherapy alone

E. Surgery + Involved Field Radiotherapy

10. Which of the following examinations is the most reliable for diagnosing lymph node involvement in NHL?

A. Lymph node aspiration

B. CT

C. Clinical physical examination

D. X-ray

E. Lymph node biopsy

11. The optimal treatment for early-stage unfavorable Hodgkin lymphoma (with bulky mediastinal mass) is:

A. 6 cycles of chemotherapy + Involved Field Radiotherapy

B. Extended field radiotherapy alone

C. 4 cycles of chemotherapy + Extended field radiotherapy

D. 4 cycles of chemotherapy + Involved Field Radiotherapy

E. 6 cycles of chemotherapy + Extended field radiotherapy

12. In radiotherapy techniques, a mantle field plus a spade field (inverted Y) irradiation belongs to:

A. Extended field irradiation

B. Total nodal irradiation

C. Involved field irradiation

D. Subtotal nodal irradiation

E. Regional field irradiation

13. Poor prognostic factors affecting chemotherapy in relapsed and refractory Hodgkin lymphoma include:

A. Primary drug resistance

B. B symptoms

C. Elderly patients

D. Extranodal involvement

E. All of the above are correct

14. The treatment that should be adopted for primary refractory Hodgkin lymphoma is:

A. HDC/AHSCT (High-Dose Chemotherapy / Autologous Hematopoietic Stem Cell Transplantation)

B. Surgery

C. Salvage chemotherapy

D. Radiotherapy

E. Radiotherapy + Chemotherapy

15. The preferred treatment regimen for Stage I precursor lymphoblastic lymphoma is:

A. Surgery

B. Hyper-CVAD regimen chemotherapy

C. Involved field radiotherapy

D. CHOP regimen chemotherapy

E. Surgery + Radiotherapy

16. For Stage I~II diffuse large B-cell lymphoma (DLBCL), the preferred treatment regimen is:

A. CHOP regimen chemotherapy for 3–4 cycles combined with involved field radiotherapy

B. R-CHOP regimen chemotherapy for 3–4 cycles

C. CHOP regimen chemotherapy for 3–4 cycles

D. R-CHOP regimen chemotherapy for 3–4 cycles combined with involved field radiotherapy

E. R-CHOP regimen chemotherapy for 3–4 cycles combined with extended field radiotherapy

17. Which of the following is the treatment principle for Stage III~IV asymptomatic follicular lymphoma?

A. Fludarabine

B. Watch and wait

C. CVP regimen chemotherapy

D. CHOP regimen chemotherapy

E. Fludarabine + Rituximab

18. Which of the following is the treatment principle for localized Stage IE nasal and nasal-type NK/T-cell lymphoma?

A. Surgery

B. Chemotherapy alone

C. Radiotherapy alone

D. Chemotherapy plus involved field radiotherapy

E. Surgery + Radiotherapy

19. For a patient with Stage I gastric mucosa-associated lymphoid tissue (MALT) lymphoma who is Helicobacter pylori (HP) positive, which treatment is preferred?

A. Surgery + Chemotherapy

B. Anti-HP therapy

C. Surgery

D. Radiotherapy

E. Surgery + Radiotherapy

20. Which of the following is not a slow-progressing (indolent) lymphoma?

A. Follicular lymphoma

B. Small lymphocytic lymphoma

C. Lymphoplasmacytic lymphoma

D. Marginal zone B-cell lymphoma

E. Precursor lymphoblastic lymphoma

21. The EORTC definition of unfavorable early-stage Hodgkin lymphoma excludes:

A. No B symptoms but ESR < 50

B. Age ≥ 50 years

C. ≥ 4 sites involved

D. Bulky mass or large mediastinum

E. Presence of B symptoms and ESR > 30

22. Which of the following is the reason why lymphoma patients often have concurrent anemia?

A. Hemolysis

B. Bone marrow invasion

C. Hypersplenism

D. All of the above are correct

E. A+B+C is not completely correct

23. Very favorable prognostic types of Hodgkin lymphoma include:

A. Pathology is lymphocyte-predominant or nodular sclerosis type

B. Female

C. Age < 40 years

D. Clinical Stage IA

E. All of the above are correct

24. The chemotherapy regimens that can be used for advanced Hodgkin lymphoma are:

A. HDC/AHSCT

B. ABVD

C. BEACOPP

D. Stanford V

E. All of the above are correct

25. Involvement of which of the following organs does not classify Hodgkin lymphoma as Stage IV?

A. Skin

B. Liver

C. Bone marrow

D. Lung

E. None of the above is correct

26. Female, 26 years old. A single painless enlarged lymph node in the right neck, 2.5cm × 3.0cm, with poor mobility. Biopsy revealed an intact capsule, no hemorrhage or necrosis. Under the microscope, the structure was destroyed, with massive proliferation of fascicular fibrous tissue and scattered large cells. The cells had abundant transparent cytoplasm, large nuclei, multiple nucleoli, forming a clear space with the surroundings (lacunar cells). Eosinophils, plasma cells, and a small amount of neutrophils were also visible. The most likely diagnosis is:

A. Reactive lymph node hyperplasia

B. Metastatic carcinoma in lymph node

C. Non-Hodgkin lymphoma

D. Lymphadenitis

E. Hodgkin disease, Nodular Sclerosis type

27. Male patient, 15 years old, chest tightness and chest pain for 2 months, accompanied by low-grade fever for over a month. The patient underwent an MRI scan (image described as anterior/middle mediastinal mass). The most likely diagnosis is:

A. Mediastinal teratoma

B. Lymphoma

C. Central lung cancer

D. Mediastinal abscess

E. Neurofibroma

28. Female patient, 28 years old, high fever accompanied by a right neck mass for over 1 month. CT examination revealed multiple enlarged lymph nodes in the neck, mediastinum, and retroperitoneum, as well as multiple space-occupying lesions in the liver and spleen with marginal enhancement, considered to be metastases. Right neck lymph node biopsy showed Diffuse Large B-Cell Lymphoma. The correct diagnostic stage belongs to:

A. Stage IVA

B. Stage II

C. Stage IIIB

D. Stage IIIA

E. Stage IVB

29. Male patient, 65 years old, admitted due to abdominal pain for over 3 months. Admission physical exam: ECOG score 1, multiple enlarged lymph nodes palpable in both sides of the neck, heart and lungs normal, abdomen soft, liver palpable 5 fingers below the ribs, spleen palpable 3 fingers below the ribs, shifting dullness negative. Ultrasound showed multiple enlarged retroperitoneal lymph nodes and multiple space-occupying lesions in the liver; combined with history, considered metastases. Serum LDH normal. Neck lymph node aspiration cytology indicated "Non-Hodgkin Lymphoma". According to the IPI, this patient should be classified into:

A. High-risk group

B. Low-risk group

C. High-intermediate risk group

D. Low-intermediate risk group

E. Very high-risk group

30. Male patient, 58 years old, admitted for a right axillary mass for 2 weeks. Postoperative pathology showed: Peripheral T-cell lymphoma (Not Otherwise Specified, PTCL-NOS). Relevant examinations were completed; chest and abdominal CT were normal, bone marrow cytology showed no lymphoma invasion. The next treatment plan should be:

A. CHOP regimen chemotherapy for 3 cycles + Right axillary radiotherapy

B. CHOP regimen chemotherapy for 6 cycles

C. CHOP regimen chemotherapy for 3 cycles

D. CHOP regimen chemotherapy for 6 cycles + Right axillary radiotherapy

E. Right axillary radiotherapy

31. Male patient, 21 years old, admitted for "chest tightness and shortness of breath for 1 week". Chest CT showed a huge mediastinal mass. Mediastinoscopy biopsy indicated "Hodgkin Lymphoma". Diagnosed as Hodgkin Lymphoma Stage IA. After 6 cycles of ABVD chemotherapy, the patient's chest tightness disappeared. Follow-up chest CT showed the mediastinal mass had shrunk compared to before (but residual exists). The next treatment plan choice is:

A. Surgery

B. Continue ABVD chemotherapy for 2 cycles

C. Change chemotherapy regimen

D. Mediastinal radiotherapy

E. Autologous stem cell transplantation

## Questions with Shared Clinical Vignettes

**[Case] Female patient, 65 years old, sore throat for over 1 month. Physical exam: Enlarged right tonsil, nasopharynx normal, clear breath sounds in both lungs, no cardiac abnormalities. Abdomen soft, no tenderness, no abdominal masses palpable, liver and spleen not palpable, shifting dullness negative. No edema in lower limbs. Tonsil biopsy showed Diffuse Large B-Cell Lymphoma (DLBCL). Chest CT and abdominal CT showed no abnormalities.**

32. If radiotherapy is performed after chemotherapy, the appropriate radiotherapy dose choice is:

A. 40–45 Gy

B. 25–30 Gy

C. 35–40 Gy

D. 30–35 Gy

E. 45–50 Gy

33. If this patient proceeds to chemotherapy next, the best regimen choice is:

A. R-CHOP

B. MINE

C. BACOP

D. CHOP

E. EPOCH

34. If the patient has a contraindication to radiotherapy, what is the appropriate number of chemotherapy cycles?

A. 8–12 cycles

B. 3 cycles

C. 6–8 cycles

D. 4 cycles

E. More than 12 cycles

35. If radiotherapy is performed after chemotherapy, the more reasonable field setup is:

A. Anterior nasal field

B. Combined face and neck field

C. Combined face and neck field + Lower neck tangential field

D. Pre-auricular field

E. Whole neck tangential field

## Questions with Shared Options

[Options]

A. Mycosis fungoides

B. Gastric mucosa-associated lymphoid tissue (MALT) lymphoma

C. Burkitt's lymphoma

D. Diffuse Large B-Cell Lymphoma

E. Nasal NK/T-cell lymphoma

**36. The lymphoma that primarily invades the skin is:**

**37. The lymphoma that can be cured without radiotherapy or chemotherapy is:**

**38. The aggressive lymphoma that is sensitive to radiotherapy but relatively resistant to chemotherapy is:**

**39. The highly aggressive lymphoma occurring in African children is:**

**40. The aggressive lymphoma that can be treated with Rituximab is:**

[Options]

A. EBV

B. Helicobacter pylori

C. HHV-8

D. HTLV-1

E. Chlamydia

**41. In pleural effusion lymphoma and body cavity lymphoma, the common infection is:**

**42. The pathogen that can be isolated from adult T-cell lymphoma patients is:**

**43. Gastric MALT lymphoma is associated with:**

**44. African childhood Burkitt's lymphoma is often accompanied by infection with:**

**45. Associated with ocular adnexal lymphoma:**

[Options]

A. Mixed cellularity type

B. Nodular lymphocyte-predominant type

C. Nodular sclerosis type

D. Lymphocyte-depleted type

E. Lymphocyte-rich type (Note: Option E in original text is "Lymphocyte-predominant" but refers to classic type in context of classification options)

**46. Microscopically, cells appear as "popcorn" type, and typical RS cells are usually not found:**

**47. The most common HD subtype in developed countries like Europe and America, characterized by lacunar RS cells:**

**48. Microscopically shows diffuse fibrosis, containing a large number of RS cells and "sarcomatoid" variant cells:**

**49. Microscopically shows typical RS cells, and the lesion is diffuse:**

**50. Microscopically lymphocytes are dominant, CD30+, CD15+, CD20+/-:**

[Options]

A. 40–45 Gy

B. 20–36 Gy

C. 50–55 Gy

D. 30–35 Gy

E. 60 Gy

**51. Involved field radiotherapy dose after chemotherapy for Hodgkin lymphoma:**

**52. Radiotherapy dose for early-stage gastric mucosa-associated lymphoid tissue (MALT) lymphoma:**

**53. Radiotherapy dose for nasal NK/T-cell lymphoma:**

**54. Involved field radiotherapy dose after chemotherapy for diffuse large B-cell lymphoma:**

# Chapter 8: Bone and Soft Tissue Tumors (N=81)

## Standard Multiple-Choice Questions

1. The incidence of primary bone tumors is:

A. 2%–3%

B. 10%–15%

C. 1%–2%

D. 5%–10%

E. 3%–5%

2. For suspected lesions in cancellous bone, which of the following imaging examinations is most advantageous?

A. Bone scan

B. X-ray

C. MRI

D. CT

E. All of the above

3. Which of the following is considered the most basic and primary imaging examination method for diagnosing bone tumors?

A. Bone scan

B. X-ray

C. MRI

D. CT

E. All of the above

4. For most primary bone tumors, diagnosis relies on:

A. Intraoperative findings

B. Clinical manifestations

C. Pathology

D. Imaging examinations

E. Combination of clinical manifestations, imaging, and pathology

5. To understand the destruction of soft tissue and cartilage by bone tumors, the best choice of examination is:

A. Bone scan

B. X-ray

C. MRI

D. CT

E. All of the above

6. Which of the following statements regarding biopsy for primary bone tumors is incorrect?

A. Manipulation should be gentle with strict hemostasis

B. Biopsy is not required for bone tumors clinically diagnosed as benign

C. The biopsy tract should be located in an area that can be resected en bloc with the tumor during the subsequent surgery

D. Trocar biopsy and incisional biopsy are commonly used

E. Immunohistochemical examination should be performed when necessary

7. Soft tissue tumors exclude tumors from which of the following origins?

A. Skin and appendages

B. Fibrous tissue

C. Synovial tissue

D. Adipose tissue

E. Vascular tissue

8. The ratio of benign to malignant soft tissue tumors exceeds:

A. 5:1

B. 100:1

C. 10:1

D. 50:1

E. 1:1

9. The most common benign soft tissue tumor is:

A. Lipoma

B. Schwannoma

C. Fibrohistiocytic and fibrous tumors

D. Vascular tumors

E. Leiomyoma

10. Generally speaking, soft tissue sarcomas most frequently occur in which of the following sites?

A. Lower leg

B. Trunk

C. Forearm

D. Thigh and pelvic girdle

E. Upper arm and shoulder girdle

11. What is the most common site of metastasis for soft tissue sarcomas?

A. Brain

B. Lung

C. Bone

D. Liver

E. Lymph nodes

12. Common characteristics of malignant soft tissue tumors exclude:

A. Persistent pain or tenderness

B. Hard texture, fixed

C. Usually located superficial to the deep fascia

D. Continuous enlargement of the mass

E. Mass is usually large

13. For most soft tissue tumors, the preferred treatment method is:

A. Biotherapy

B. Radiotherapy

C. Surgery

D. Chemotherapy

E. Hyperthermia

14. For which of the following soft tissue tumors does chemotherapy have definite value?

A. Angiosarcoma

B. Liposarcoma

C. Synovial sarcoma

D. Leiomyosarcoma

E. Ewing sarcoma

15. The radiotherapy dose for the tumor bed after surgery for soft tissue sarcoma of the extremities should reach at least (1.8–2.0 Gy/F):

A. 60–65 Gy

B. 45–50 Gy

C. 55–60 Gy

D. 50–55 Gy

E. 65–70 Gy

16. Which description regarding radiotherapy for soft tissue sarcoma of the extremities is incorrect?

A. Include the entire long bone

B. Try to avoid irradiation across joints

C. Preserve a strip of normal skin and subcutaneous tissue on one side

D. Include the entire origin of the involved muscle

E. Pay attention to protecting important organ functions

17. For which of the following bone tumors is chemotherapy not used as a major treatment modality?

A. Myeloma

B. Ewing sarcoma

C. Chondroblastoma

D. Malignant fibrous histiocytoma (MFH)

E. Osteosarcoma

18. Which bone tumor has benign histological features but can cause lung metastases in a few patients?

A. Myeloma

B. Ewing sarcoma

C. Chondroblastoma

D. Malignant fibrous histiocytoma

E. Giant cell tumor of bone

19. When a clinical diagnosis of osteosarcoma is made, what is the proportion of patients who already have pulmonary micrometastases?

A. 60%–70%

B. 30%–40%

C. 50%–60%

D. 40%–50%

E. More than 80%

20. Which of the following drugs is not considered one of the main effective drugs for osteosarcoma chemotherapy?

A. Etoposide (VP-16)

B. Doxorubicin

C. Methotrexate

D. Ifosfamide

E. Cisplatin

21. Bone metastasis is most frequently seen in which of the following malignant tumors?

A. Thyroid cancer

B. Lung cancer

C. Kidney cancer

D. Prostate cancer

E. Breast cancer

22. Bone metastasis most commonly occurs in which of the following sites?

A. Spine and pelvis

B. Proximal femur

C. Ribs

D. Scapula

E. Skull

23. The 5-year survival rate for osteosarcoma with comprehensive treatment can reach:

A. 40%–50%

B. Around 75%

C. 50%–60%

D. 60%–70%

E. 30%

24. Which description regarding limb-salvage surgery for malignant bone tumors is incorrect?

A. Must ensure that wide or radical resection margins are achieved in all areas

B. Resect the tumor bone and host bone en bloc

C. The function of the reconstructed limb should not be inferior to a prosthesis

D. Limb reconstruction includes bone and joint reconstruction as well as soft tissue reconstruction

E. It has replaced amputation

25. Which of the following bone tumors is relatively sensitive to radiotherapy?

A. Malignant fibrous histiocytoma of bone

B. Ewing sarcoma

C. Osteosarcoma

D. Chondrosarcoma

E. Chondroblastoma

26. Which of the following bone tumors is insensitive to both radiotherapy and chemotherapy?

A. Malignant fibrous histiocytoma of bone

B. Ewing sarcoma

C. Osteosarcoma

D. Chondrosarcoma

E. None of the above

27. Prognostic factors for malignant bone tumors include:

A. Site of metastasis

B. Size of primary tumor

C. Location of primary tumor

D. Histological grade

E. All of the above

28. The main purpose of surgery for bone metastases is:

A. Fixation of pathological fractures and relief of spinal cord compression

B. Reduction of tumor burden

C. Pain relief

D. Radical surgery

E. Obtaining histological diagnosis

29. The most common mode of metastasis for soft tissue sarcoma is:

A. Direct invasion

B. Hematogenous metastasis

C. Implantation metastasis

D. Lymphatic metastasis

E. All of the above

30. Which description regarding rhabdomyosarcoma is correct?

A. Embryonal rhabdomyosarcoma can be cured by radiotherapy alone

B. There are two main types: myxoid and embryonal

C. Main sites of metastasis are lung and bone

D. Highly malignant, rapid growth

E. Lymph node metastasis is rare

31. Which soft tissue sarcoma has the highest rate of lymph node metastasis?

A. Synovial sarcoma

B. Liposarcoma

C. Leiomyosarcoma

D. Rhabdomyosarcoma

E. Fibrosarcoma

32. Which description regarding the Enneking surgical staging system is correct?

A. Benign tumors are surgically graded as G1

B. Based on surgical grade (G), anatomical location (T), and presence of metastasis (M)

C. Benign tumors are all Stage 1

D. Lymph node metastasis belongs to T staging

E. "A" indicates the primary tumor is extracompartmental

33. Chemotherapy has become an important treatment modality for certain malignant bone tumors, including:

A. Myeloma

B. Ewing sarcoma

C. Osteosarcoma

D. Malignant fibrous histiocytoma of bone

E. All of the above

34. Which description regarding giant cell tumor of bone is incorrect?

A. X-ray shows osteolytic destruction

B. May originate from undifferentiated mesenchymal cells in the marrow cavity

C. Predilection for the epiphyseal plate region of long bones after closure

D. Peak age incidence is between 20 and 40 years

E. Because it is a potentially malignant tumor, wide or radical resection is mostly chosen for surgery

35. Which description regarding the MTS staging system is correct?

A. Risk factors include number of lesions, size, and time to metastasis

B. Emphasizes providing useful prognostic information

C. Each stage is divided into A and B based on the presence of risk factors

D. Divided into Stages I–IV

E. None of the above is correct

36. Bone tumors that commonly occur in adolescents exclude:

A. Ewing sarcoma

B. Chondroblastoma

C. Osteosarcoma

D. Osteoid osteoma

E. Chondrosarcoma

37. The tumor currently considered to require routine postoperative chemotherapy is:

A. Liposarcoma

B. Synovial sarcoma

C. Ewing sarcoma

D. Malignant fibrous histiocytoma of soft tissue

E. Chondrosarcoma

38. Melanoma occurs mostly in Caucasians, especially those with a history of sun exposure, while the incidence in China is lower, approximately:

A. 5–6/100,000

B. 0.4–0.5/100,000

C. 10–15/100,000

D. 0.2/100,000

E. 16/100,000

39. Malignant melanoma most commonly occurs in which of the following sites?

A. Skin of extremities in females

B. Iris of the eye

C. Mucosa of the digestive tract

D. Choroid plexus of the meninges

E. Ciliary body of the eye

40. The most common type of cutaneous malignant melanoma is:

A. Lentigo maligna melanoma

B. Nodular melanoma

C. Papillary melanoma

D. Acral lentiginous melanoma

E. Superficial spreading melanoma

41. Which of the following is not an early manifestation of cutaneous malignant melanoma lesions?

A. Irregular surface elevation, roughness, scaling, and exudation

B. Color change, especially blue-black, gray, brown, and variegated colors are most important

C. Irregular borders, serrated appearance

D. Palpable distinctly enlarged, hard, irregular mass with obvious tenderness

E. Rapid enlargement of lesion, persistent itching, crusting, or appearance of satellite nodules

42. The differential diagnosis for amelanotic melanoma does not include:

A. Soft tissue sarcoma

B. Poorly differentiated adenocarcinoma

C. Lymphoma

D. Undifferentiated carcinoma

E. Germ cell carcinoma

43. Which description regarding the TNM staging principles for malignant melanoma is incorrect?

A. When the primary tumor is ulcerated, the stage for all Stage I, II, and III patients should be increased by one stage

B. T staging is determined by melanoma thickness and ulceration, not by extent of invasion (except T1)

C. M staging depends on the site of distant metastasis and serum lactate dehydrogenase levels

D. N staging depends on the number of metastatic lymph nodes rather than size

E. Appearance of satellite metastases or in-transit metastases before the first station lymph nodes should be classified as IIIB

44. Which description regarding regional lymph node staging in the TNM staging of malignant melanoma is incorrect?

A. Metastasis to 4 or more lymph nodes, or matted lymph nodes, or local lymph node metastasis accompanied by satellite metastasis or in-transit metastasis is N3

B. Metastasis to 2–5 regional lymph nodes or presence of intralymphatic metastasis is N2

C. No local lymph node metastasis but presence of satellite metastasis or in-transit metastasis is N2c

D. Metastasis to one lymph node and clinically occult (microscopic metastasis) is N1a

E. Regional lymph nodes cannot be assessed is Nx

45. The correct description for pathological Stage IIIC in malignant melanoma is:

A. T1–4a N2b M0

B. T1–4b N2a M0

C. T1–4b N2b M0

D. T1–4a N1b M0

E. T1–4α/β N2c M0

46. Which of the following treatment principles for malignant melanoma is incorrect?

A. For Stage IV patients, chemotherapy and biotherapy are the mainstay; local radiotherapy, symptom-reducing surgery, and palliative surgery can also be considered

B. For carcinoma in situ, the recommended resection margin is 0.5–1 cm from the lesion or biopsy scar

C. For post-operative Stage IIB/IIC patients and Stage III patients who have undergone lymph node dissection, adjuvant biotherapy helps reduce the risk of recurrence and metastasis

D. For patients without biopsy but clinically suspected lymph node involvement, the primary lesion must be resected but regional lymph node dissection is not required

E. For patients with only skin or lymph node metastases, local radiotherapy can sometimes be considered

47. For post-operative Stage IIB/IIC patients and Stage III patients who have undergone lymph node dissection, post-operative adjuvant therapy helps reduce the risk of recurrence and metastasis. The primary agent used is:

A. Interleukin-2

B. Mitomycin

C. Interferon α-2b

D. Interleukin-11

E. Methotrexate

48. Treatment for advanced malignant melanoma patients primarily involves chemotherapy combined with biotherapy. Which of the following is excluded from biotherapy modalities (drugs)?

A. Interleukin-2

B. LAK cells

C. Interferon α-2b

D. Tumor Necrosis Factor (TNF)

E. Vaccines

49. Which of the following drugs is the main chemotherapy agent for advanced malignant melanoma?

A. Dacarbazine

B. Doxorubicin

C. Paclitaxel

D. Gemcitabine

E. Cisplatin

50. Which description regarding surgical treatment principles for malignant melanoma is incorrect?

A. For lesions with thickness >4mm, it is recommended to strive for a surgical resection margin of 3cm from the lesion or biopsy scar

B. For lesions with thickness <1mm, surgical resection margin is 0.5–1cm from the lesion or biopsy scar

C. For lesions with thickness 2–4mm, it is recommended that the surgical resection margin be 2–3cm from the lesion or biopsy scar

D. For lesions with thickness 1–2mm, strive for a surgical resection margin of 2cm from the lesion or biopsy scar

E. For lesions with thickness <1mm, regional lymph node dissection is recommended

51. According to AJCC surveys, the 5-year survival rate for Stage I malignant melanoma patients is approximately:

A. 65%

B. 90%

C. 50%

D. 75%

E. 43%

52. Clark histologically classified lesions based on the depth of tumor invasion in malignant melanoma. Which of the following descriptions is incorrect?

A. Level IV is lesion confined to the reticular dermis

B. Level I is lesion confined within the epidermis

C. Level III is invasion to the junction of the papillary and reticular dermis

D. Level II is invasion into the papillary dermis

E. Level V is lesion reaching the subcutaneous tissue

53. Distant metastasis of malignant melanoma is most common in which of the following sites?

A. Brain

B. Liver

C. Bone

D. Lung

E. Retroperitoneum

54. Which description regarding radiotherapy for malignant melanoma is correct?

A. For invasive lesions <1mm thick, radiation field margin is 1cm outside the tumor

B. Very high fractional doses are not required

C. Radiation field margin for carcinoma in situ is 0.5cm outside the tumor

D. Total dose is 50–60 Gy, with 50 Gy being appropriate for carcinoma in situ

E. For invasive lesions 1–4mm thick or >4mm, radiation field margin is 2cm outside the tumor

55. Which description regarding indications for radiotherapy in malignant melanoma is incorrect?

A. Patients over 60 years old with lesion thickness >1mm are not suitable for radiotherapy

B. Malignant melanoma is relatively insensitive to radiotherapy and it is usually not the first choice of treatment

C. Can be used for patients with facial lesions that are thickening, or young patients who refuse surgery because major surgery would greatly affect facial appearance

D. Radiotherapy can be considered for scalp lesions

E. Radiotherapy can also be performed for unresectable locally advanced, metastatic, or recurrent malignant melanoma lesions

56. Which description regarding the treatment of brain metastases from malignant melanoma is incorrect?

A. In addition to relieving symptoms, surgical treatment can prolong the brain disease-free interval

B. Corticosteroid therapy is often used to reduce edema and alleviate symptoms, achieving palliative goals

C. In patients with single metastasis, the median survival after surgery is 10 months; patients who previously responded to immunotherapy have better outcomes

D. If the lesion is solitary and symptomatic, X-knife or surgery can be given, with no obvious neurological damage after treatment

E. For patients with multiple brain metastases, whole-brain radiotherapy has good efficacy

57. Male patient, 17 years old, lower limb swelling and pain with difficulty walking for over 2 months. X-ray image follows (below). What is the most likely diagnosis?

A. Osteosarcoma

B. Giant cell tumor of bone

C. Tuberculosis of bone

D. Osteochondroma

E. Bone metastasis

58. Male patient, 21 years old, bone tumor in the lower end of the right femur. Biopsy showed Ewing sarcoma. Maximum tumor diameter is 4cm, no lymph node or distant metastasis found. The AJCC stage is:

A. Stage IIB

B. Stage I

C. Stage IIA

D. Stage IB

E. Stage III

59. Female patient, 65 years old, discovered a mass on the shoulder/back 5 years ago. It grows slowly, currently about 5cm x 4cm, soft texture, unclear borders, no tenderness, no redness or swelling of overlying skin, no varicose veins. The most likely diagnosis is:

A. Sebaceous cyst

B. Leiomyoma

C. Neurofibroma

D. Skin cancer

E. Lipoma

60. Female patient, 18 years old, swelling and pain in the lower segment of the right thigh for over 2 months. X-ray shows an area of bone destruction with unclear boundaries in the lower segment of the femur, periosteal proliferation and radial shadows, and Codman triangles visible at both ends. The most likely diagnosis is:

A. Giant cell tumor of bone

B. Osteomyelitis

C. Bone metastasis

D. Tuberculosis of bone

E. Osteosarcoma

61. Male patient, 21 years old, swelling and pain in the lower segment of the left thigh for 3 months. X-ray shows a bone tumor in the lower end of the left femur, osteosarcoma is likely. Biopsy confirms osteosarcoma. The appropriate next step is:

A. High-dose radiotherapy, followed by limb salvage or amputation

B. Immediate amputation

C. Limb salvage or amputation surgery, followed by postoperative chemotherapy

D. Limb salvage surgery after high-dose radiotherapy

E. Chemotherapy followed by limb salvage or amputation surgery, postoperative chemotherapy, consider postoperative radiotherapy

## Questions with Shared Clinical Vignettes

**[Case] Male patient, 15 years old, left thigh pain for half a month. X-ray indicates osteolytic destruction in the middle segment of the left femur with surrounding soft tissue swelling. Biopsy confirms Ewing sarcoma.**

62. [Single Choice] What is the most common site of distant metastasis for this disease?

A. Lung, Brain

B. Liver, Bone

C. Lung, Bone

D. Lung, Liver

E. Liver, Brain

63. [Single Choice] With comprehensive treatment, the overall 5-year survival rate for Ewing sarcoma reaches:

A. 60%–70%

B. 30%–40%

C. 50%–60%

D. 40%–50%

E. More than 70%

64. [Single Choice] If the lesion is localized, which of the following treatment methods is preferred?

A. Neoadjuvant chemotherapy + wide or radical surgery + adjuvant chemotherapy

B. Wide or radical surgery

C. Surgery + postoperative radiotherapy

D. Surgery + postoperative chemotherapy

E. Chemotherapy + radiotherapy

**[Case] Male patient, 16 years old, swelling and pain in the upper segment of the left lower leg for over 3 months, obvious night pain. Physical exam: obvious swelling of the upper segment of the left tibia, tenderness, a hard mass approx. 6cm x 7cm is palpable, fixed. X-ray shows moth-eaten osteolytic destruction in the upper segment of the left tibia, obvious periosteal reaction, Codman triangle visible, local soft tissue invasion and swelling.**

65. [Single Choice] The examination that should be routinely performed is:

A. Chest X-ray

B. Lymphangiography

C. Head CT

D. Gastrointestinal barium meal

E. Bone marrow aspiration

66. [Single Choice] The clinical diagnosis is considered to be:

A. Giant cell tumor of bone

B. Osteomyelitis

C. Osteosarcoma

D. Myeloma

E. Bone cyst

67. [Single Choice] The best treatment plan is:

A. Limb salvage/amputation, preoperative chemotherapy, postoperative radiotherapy

B. Chemotherapy alone

C. Curettage + bone cement filling, pre- and postoperative chemotherapy

D. Radiotherapy alone

E. Limb salvage/amputation, pre- and postoperative chemotherapy

**[Case] Female patient, 39 years old, right upper thigh pain for 1 month. Physical exam: tenderness in the upper segment of the right femur, limited movement of the right hip joint. X-ray: osteolytic bone destruction in the right femoral neck and subtrochanteric region. She had breast cancer 3 years ago and underwent radical mastectomy, but received no adjuvant treatment after surgery.**

68. [Single Choice] The most likely diagnosis is:

A. Giant cell tumor of bone

B. Osteosarcoma

C. Chondrosarcoma

D. Myeloma

E. Breast cancer recurrence (bone metastasis)

69. [Single Choice] To understand whether there are coexisting lesions in other skeletal sites, the preferred examination is:

A. X-ray tomography

B. CT

C. Bone ECT (Bone Scan)

D. MRI

E. Bone marrow aspiration

**[Case] A female breast cancer patient complained of lower back pain and discomfort for over 2 months, 3 years after comprehensive treatment including surgery, radiotherapy, and chemotherapy. Her sleep at night was poor due to pain, but plain X-ray of the lumbar spine showed no abnormalities.**

70. [Single Choice] To determine early on whether there is bone metastasis, she should further undergo:

A. Alkaline phosphatase level testing

B. Bone scan examination

C. CT examination

D. MRI examination

E. Biopsy puncture

71. [Single Choice] Two months later, the patient's pain worsened, and MRI confirmed destruction of the L3 and L4 vertebral bodies. Which of the following palliative treatments should be performed?

A. Radionuclide therapy

B. Palliative chemotherapy

C. Endocrine therapy

D. Palliative radiotherapy

E. Molecular targeted therapy

72. [Single Choice] To control the patient's pain, during the initial titration of analgesic dosage, which of the following formulations should be chosen?

A. Tramadol

B. Fentanyl transdermal patch

C. Immediate-release morphine tablets

D. Long-acting controlled-release morphine

E. Pethidine (Demerol)

## Questions with Shared Options

[Options]

A. 40–60 years old

B. Under 10 years old

C. 20–40 years old

D. 5–15 years old

E. Over 60 years old

**73. [Single Choice] Peak age for Ewing sarcoma:**

**74. [Single Choice] Peak age for Giant cell tumor of bone:**

**75. [Single Choice] Peak age for Chondrosarcoma:**

[Options]

A. Preoperative radiotherapy + surgery

B. Preoperative chemotherapy + surgery + postoperative chemotherapy

C. Surgery + postoperative chemotherapy

D. Surgery

E. Radiotherapy

**76. [Single Choice] Main treatment modality for Ewing sarcoma:**

**77. [Single Choice] Main treatment modality for Chondrosarcoma:**

**78. [Single Choice] Main treatment modality for Osteosarcoma:**

[Options]

A. Leiomyosarcoma

B. Malignant fibrous histiocytoma

C. Synovial sarcoma

D. Rhabdomyosarcoma

E. Liposarcoma

**79. [Single Choice] The most common soft tissue sarcoma near the hands and feet is:**

**80. [Single Choice] The soft tissue sarcoma most common in the abdominal cavity and retroperitoneal area is:**

**81. [Single Choice] After the lesion in the lower limb is controlled, there is still a 30%–50% chance of a second lesion appearing in the retroperitoneum. This soft tissue sarcoma is:**
